# Supplementary material for: A modular platform for bioluminescent RNA tracking
Source: Nat Commun. 2024 Nov 18;15:9992. doi: 10.1038/s41467-024-54263-5 (PMC11574019; doi:10.1038/s41467-024-54263-5)
Supplement: Supplementary file 1 — Supplementary Information [file 41467_2024_54263_MOESM1_ESM.pdf]

Supplementary Information

Supplementary Figs. 1 to 14

Supplementary Table 1

Supplementary Note 1

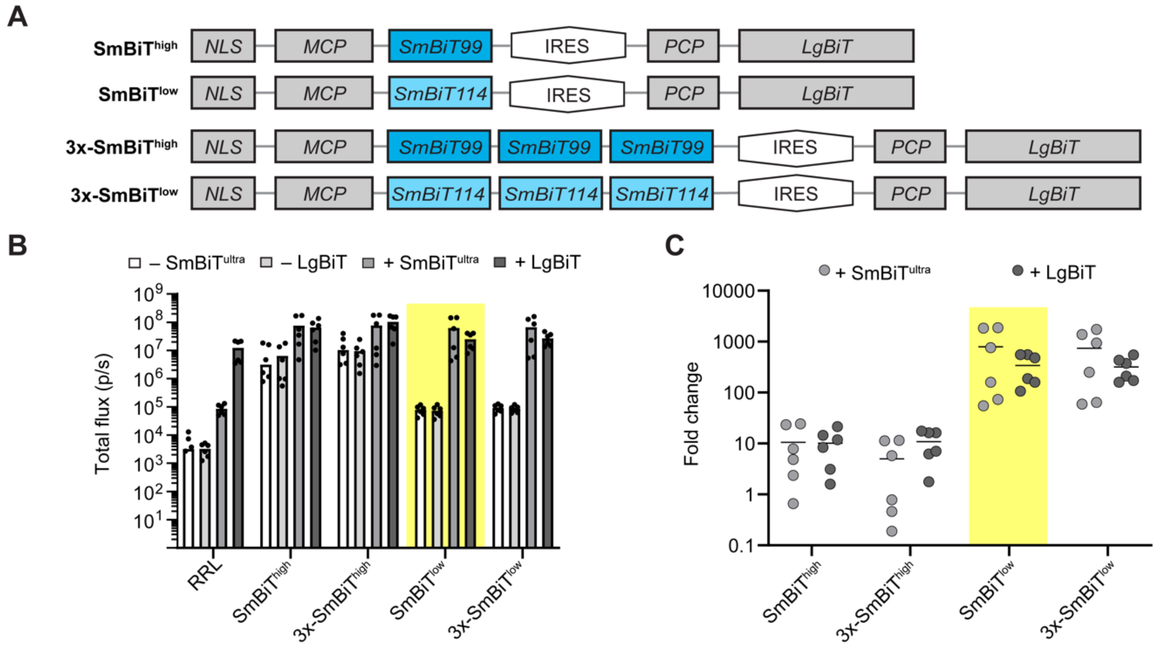

Supplementary Figure 1: Optimization of the split NanoLuc RNA lantern. (**A**) SmBiT^{high} (SmBiT99, dark blue) and SmBiT^{low} (SmBiT114, light blue) and their serial trimers were constructed as NLS-MCP fusions and co-expressed with IRES-driven PCP-LgBiT. (**B**) The lantern constructs were expressed in IVTT (rabbit reticulocyte lysate, RRL) and evaluated for RNA-independent photon production alone or supplemented with synthetic SmBiT^{ultra} (SmBiT86, 10 µM) or recombinant LgBiT (10 µM), as noted. The IVTT-expressed lanterns provide background photon measurements, whereas the supplemented experiments (+SmBiT^{ultra} or +LgBiT) represent the saturated complexes, reporting the maximum potential photon output from each construct. Only four of six samples are plotted in RRL for −SmBiT^{ultra} because two samples were below the limit of detection for the instrument. (**C**) Fold change in light output from (**B**). Lines represent the means of the populations for n = 6 replicates. Source data for B and C are provided as Source Data files.

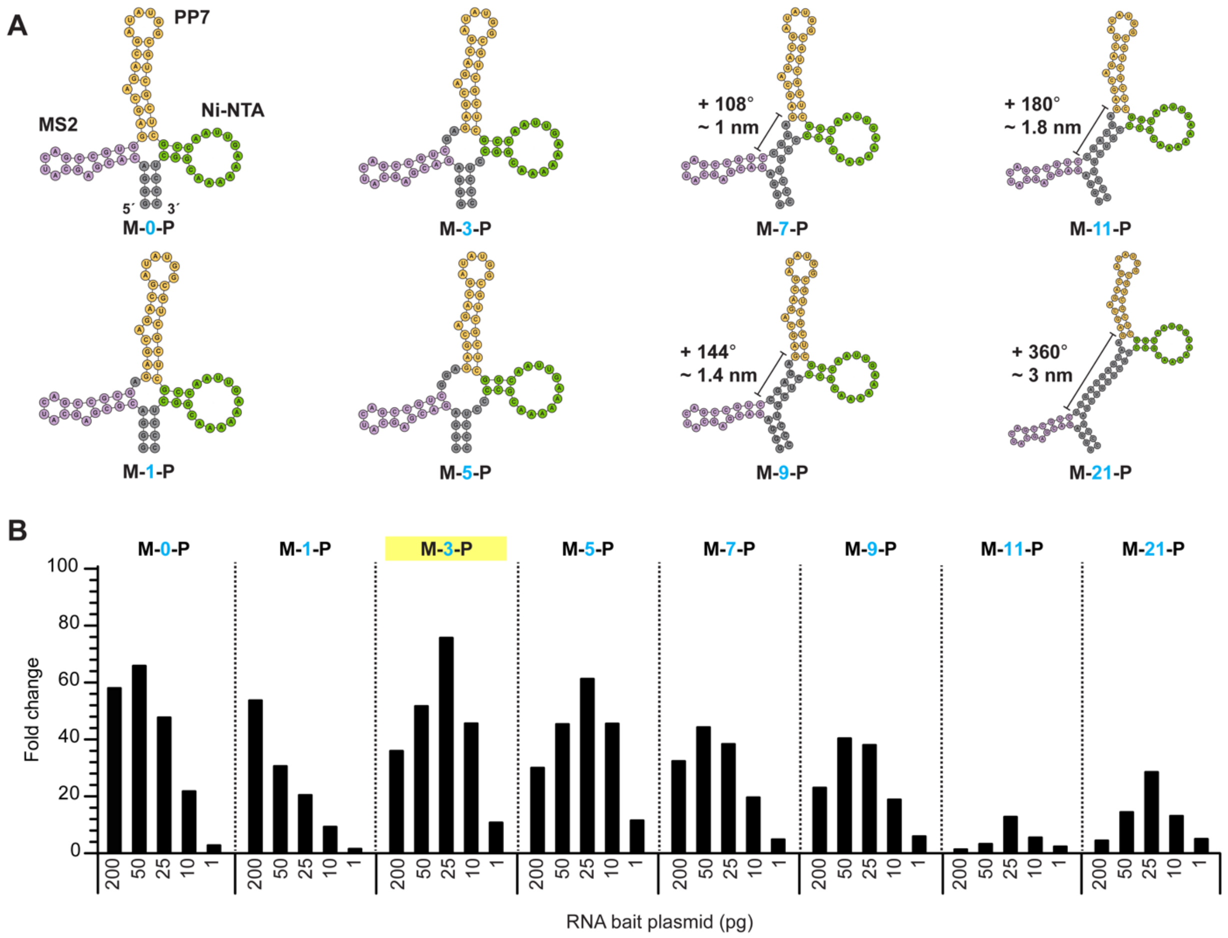

Supplementary Figure 2: Design of MCP/PCP baits. (**A**) RNA baits were designed with varying number of nucleotides between the MS2 (purple) and PP7 (orange) aptamers. A Ni-NTA aptamer (green) was also added for purification purposes and improved structural rigidity. (**B**) Luminescence readouts obtained with each RNA bait. Data are plotted as the fold change in signal from each RNA bait over a no-RNA control sample. Decreasing concentrations of RNA bait template were assessed with 1 ng of the lantern DNA plasmid. The mean fold change for each sample (n = 2) is shown. Source data for B are provided as a Source Data file.

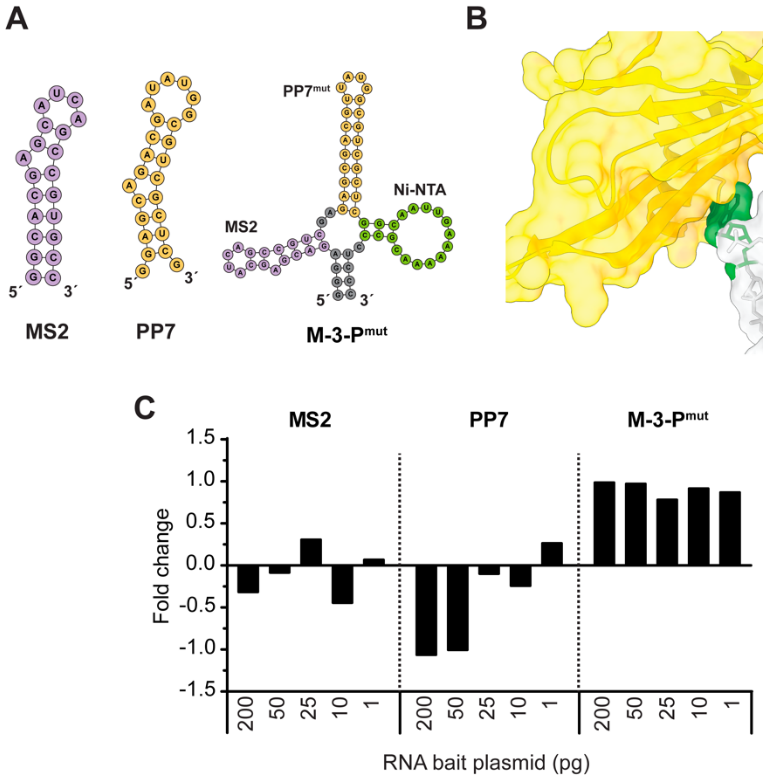

Supplementary Figure 3: Lack of luminescence observed with control RNAs. (**A**) Predicted secondary structures of control RNA baits comprising individual MS2 or PP7 aptamers or the M-3-P^{mut} construct containing two mutations (deletion of A6 and G10). (**B**) Location of the deleterious PP7 mutations in the structure of the unmutated aptamer-protein complex (PDB: 2QUX). (**C**) Fold change in signal observed with each control RNA bait over a no-RNA sample. Decreasing concentrations of RNA bait template were tested with 1 nM of the lantern DNA plasmid. The mean fold change for each sample (n = 2) is shown. Source data for C are provided as a Source Data file.

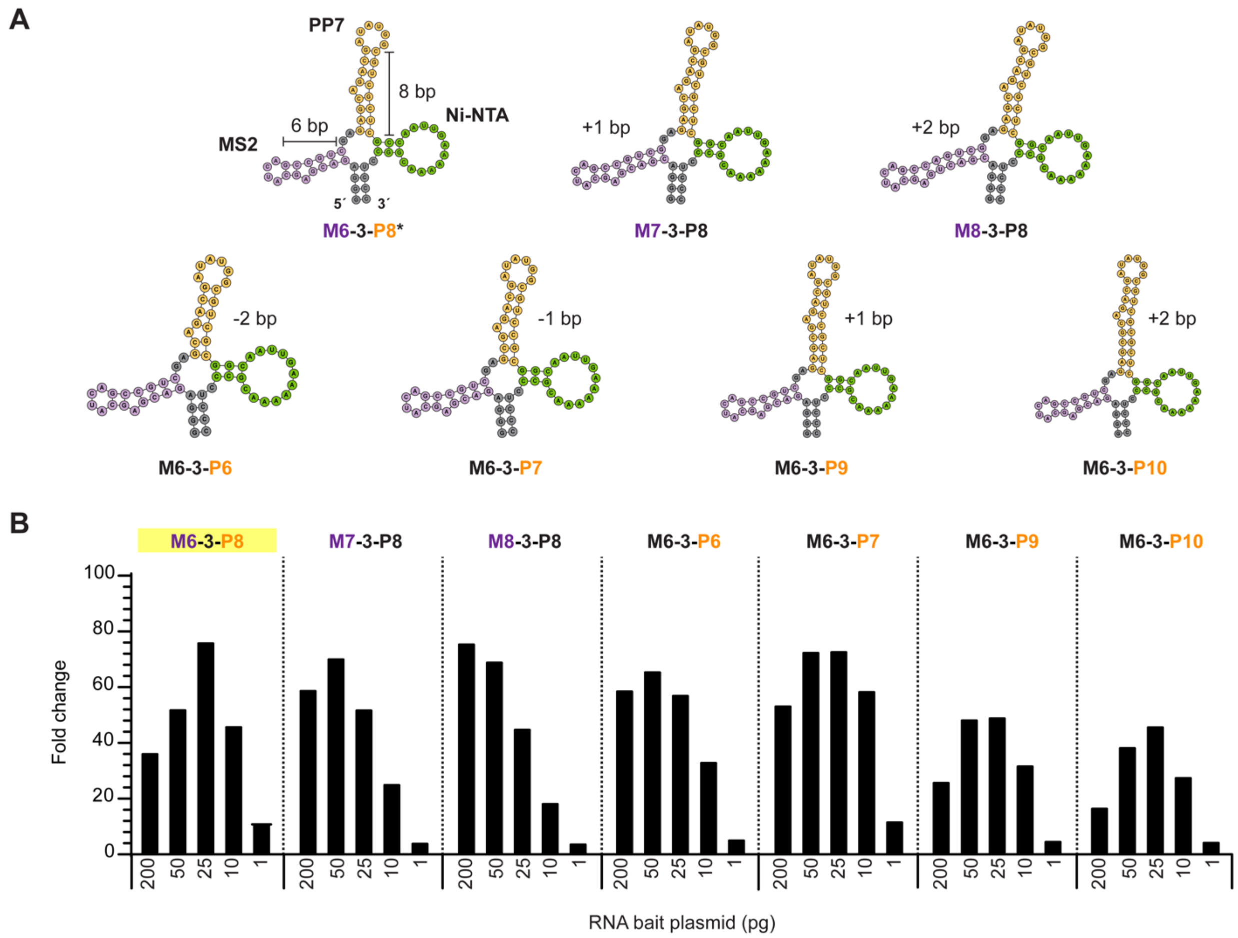

Supplementary Figure 4: Modulation of aptamer stem lengths. (**A**) The stem regions of the MS2 (purple) and PP7 (orange) aptamers were varied by changing the number of base pairs. The asterisk indicates RNA bait with unmodified MS2 and PP7 stems (Fig. 1E). (**B**) Fold change in signal observed with each RNA bait design over a no-RNA control. Decreasing concentrations of each RNA bait template were tested with 1 ng of the lantern DNA plasmid. The mean fold change for each sample (n = 2) is shown. Source data for B are provided as a Source Data file.

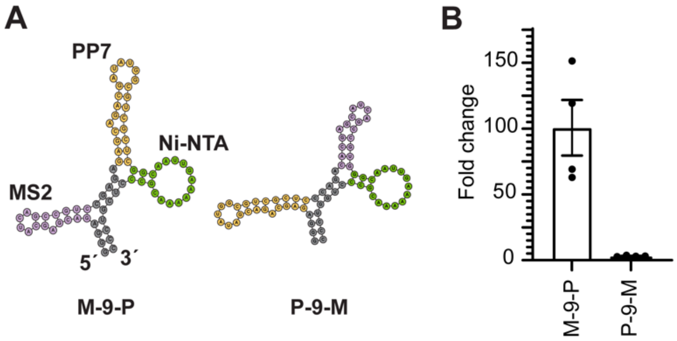

Supplementary Figure 5: Aptamer order is critical for lantern assembly. (**A**) The PP7 aptamer (orange) was placed either 5′ (right) or 3′ (left) of the MS2 aptamer (purple) to evaluate the effect of binding site orientation on lantern function. (**B**) RNA baits were assessed using IVTT and the fold changes in light output over samples without RNA bait are plotted. Error bars represent the standard error of the mean (SEM) for n = 4 replicates. Source data for B are provided as a Source Data file.

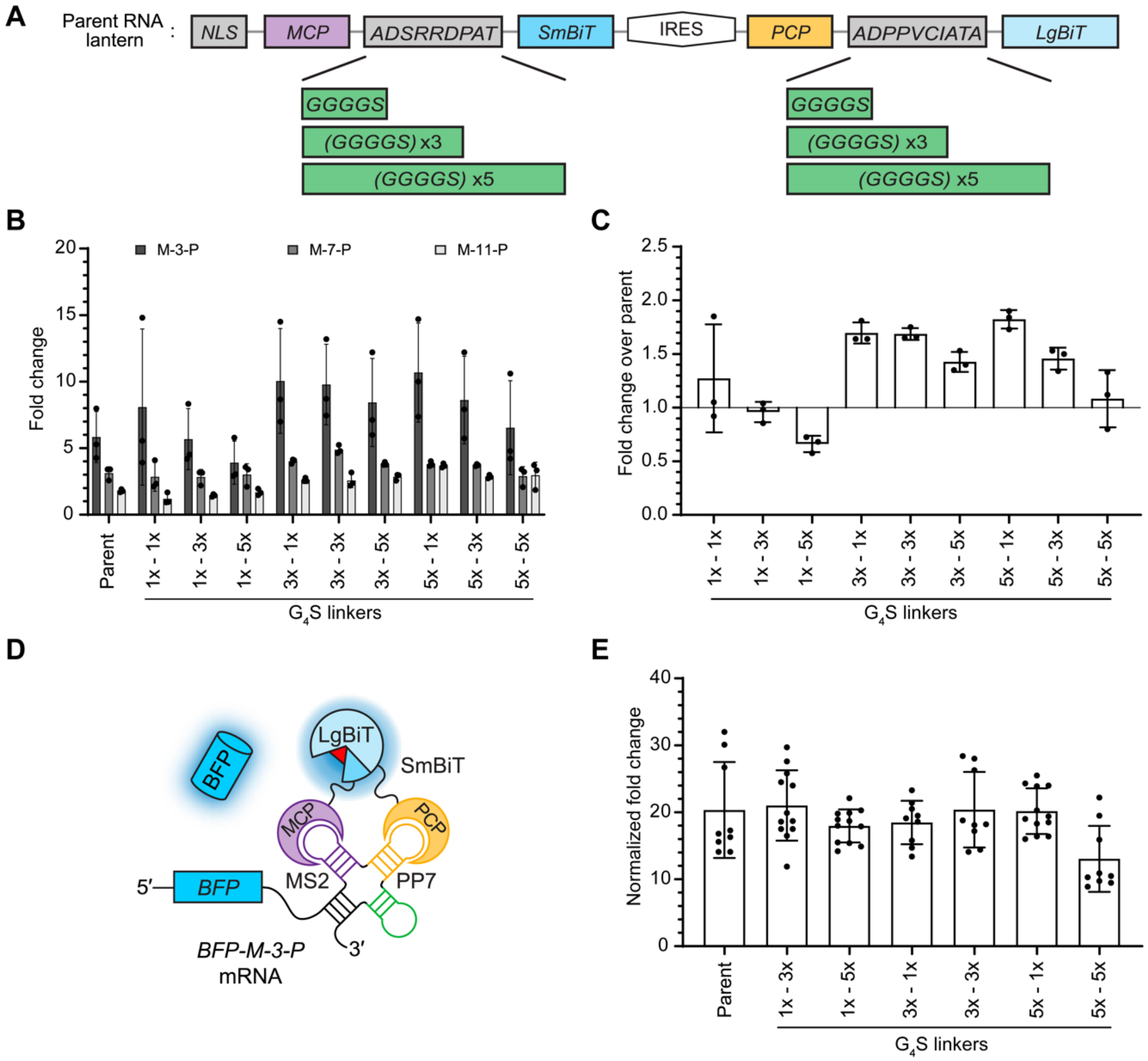

Supplementary Figure 6: Lantern linker lengths have a smaller effect on photon output than RNA bait structure. (**A**) Schematic of G4S linker changes in the RNA lantern. (**B**) Protein linkers comprising 1–5 copies of a glycine-serine repeating unit (G4S) were examined in IVTT. Luminescence outputs were recorded in the presence of various RNA baits. Data are plotted as the fold change in signal over no-RNA bait controls. (**C**) Fold change in luminescence observed with RNA lanterns comprising altered linkers and M-3-P versus the original lantern and M-3-P. No substantial increase in signal was observed with probes comprising G4S units. For (**B**)–(**C**), error bars represent the standard deviation (SD) for n = 3 replicates. (**D**) Schematic of an mRNA encoding blue fluorescent protein (BFP) and M-3-P in the 3′ UTR. Transcription of *BFP-M-3-P* mRNA recruits the RNA lantern, resulting in light production. BFP fluorescence enables confirmation of expression. (**E**) RNA lanterns comprising various linker lengths were examined. Cells stably expressing each lantern were transfected with the *BFP-M-3-P* construct. Data are plotted as the fold-change in signal over non-transfected cells. Luminescence measurements were normalized to BFP expression (assessed via flow cytometry). Nine replicates are shown, and the error bars represent the standard deviation (SD) for all replicates. Minimal improvement over the initial RNA lantern design was observed with varying glycine-serine linkers. The 5x-5x design (five G4S linkers in both protein fusions) exhibited reduced signal turn-on both *in vitro* and *in*

cellulo, suggesting disfavored complementation with increasing linker length. Source data for B, C, and E are provided as Source Data files.

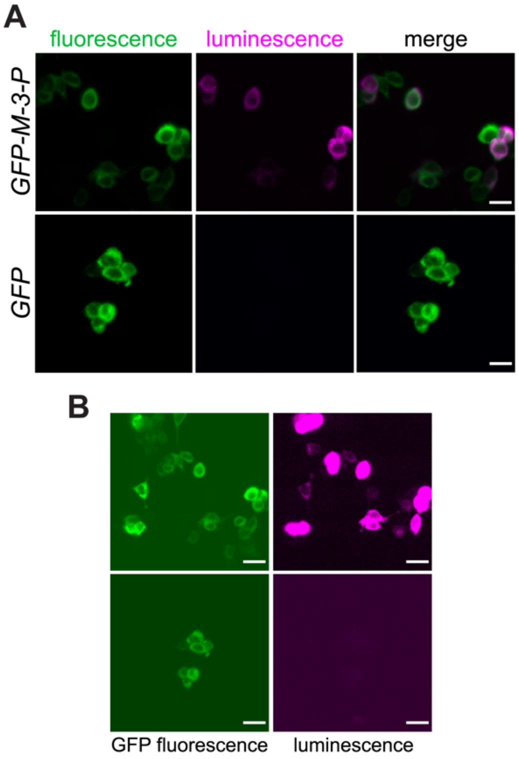

Supplementary Figure 7: RNA imaging *in cellulo* with bioluminescent lantern. (**A**) HEK293T cells expressing RNA lanterns were transfected with DNA encoding *GFP–M-3-P* or *GFP* only. The images shown derive from the experiment described in Fig. 5B. (**B**) Expanded fields of view and detection thresholds for cells transfected with DNA encoding *GFP–M-3-P* or *GFP* only from (**A**). Luminescence above background was observed in 62% (18/29) of GFP-expressing cells. Scale bars = 20 µm. Source data for A and B are provided as Source Data files.

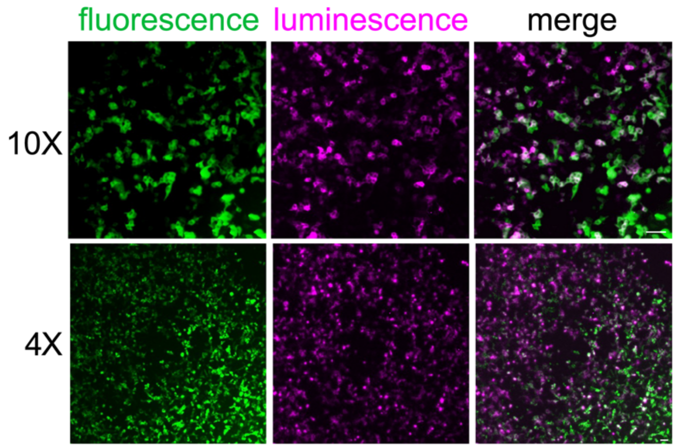

Supplementary Figure 8: Imaging of RNA lanterns in mammalian cells. HEK293T cells expressing RNA lanterns were transfected with DNA encoding *GFP–M-3-P*. Luminescence was observed in cells containing mRNAs with the M-3-P bait using both a 10X and 4X objective. Scale bars = 80 μm. Source data are provided as a Source Data file.

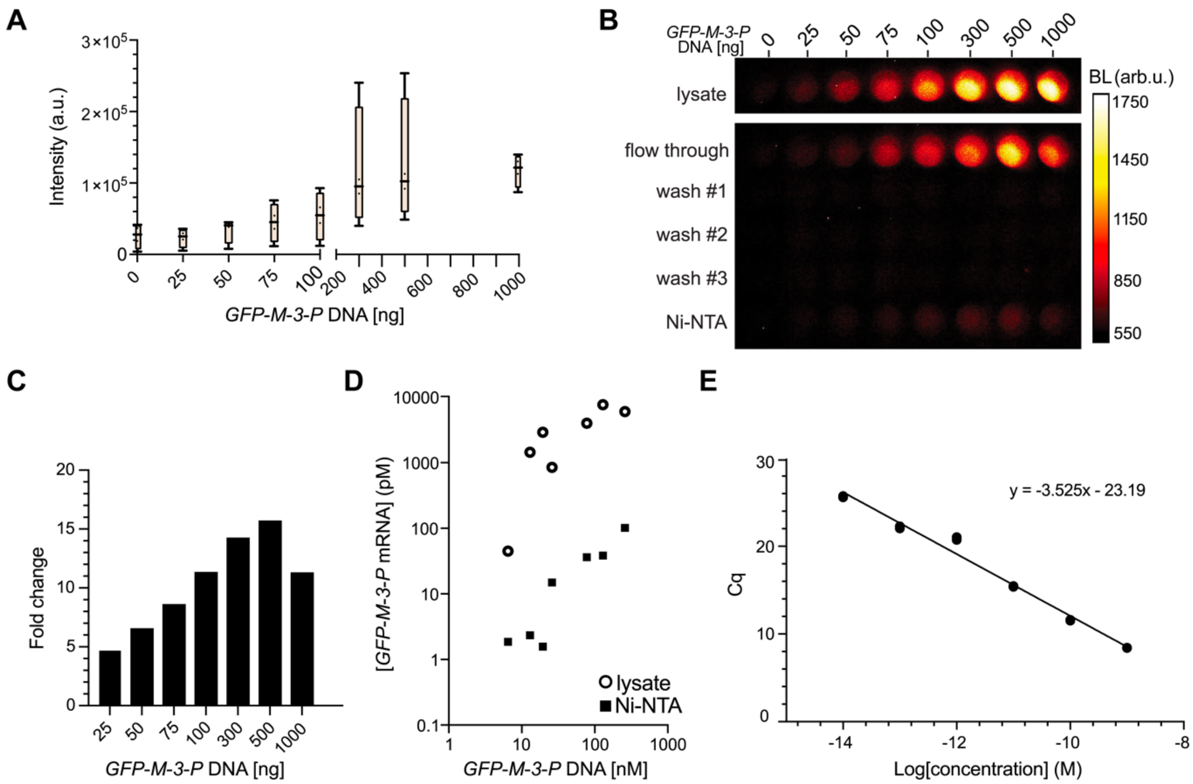

Supplementary Figure 9: Quantification of RNA lantern complexes in mammalian cells. (**A**) Bulk bioluminescent outputs from HEK293T cells expressing RNA lanterns. Cells were transfected with DNA encoding *GFP-M-3-P* (or no DNA) and analyzed. Error bars represent the standard deviation (SD) of the mean for $n = 4$ replicates. (**B**) Bioluminescence of RNA lantern complexes captured from homogenized cells from (A) using the Ni-NTA aptamer. (**C**) Fold change in bioluminescent signal from RNA lantern complexes retrieved in the presence of *GFP-M-3-P* versus no mRNA from (A-B). (**D**) Concentrations of *GFP-M-3-P* mRNA from cell lysate (open circles) and *GFP-M-3-P* mRNA (closed squares) captured on Ni-NTA resin from (B). Values were determined using real-time quantitative PCR (qPCR). (**E**) Standard curve from qPCR in (D), with ten-fold serial dilutions of *GFP-M-3-P* cDNA (10^{-14}–10^{-9} M). Data were used to establish the quantification cycle (Cq) relative to log[RNA]. Duplicate measurements were taken and are both displayed. BL, bioluminescence. arb.u., arbitrary units. Source data for A–E are provided as Source Data files.

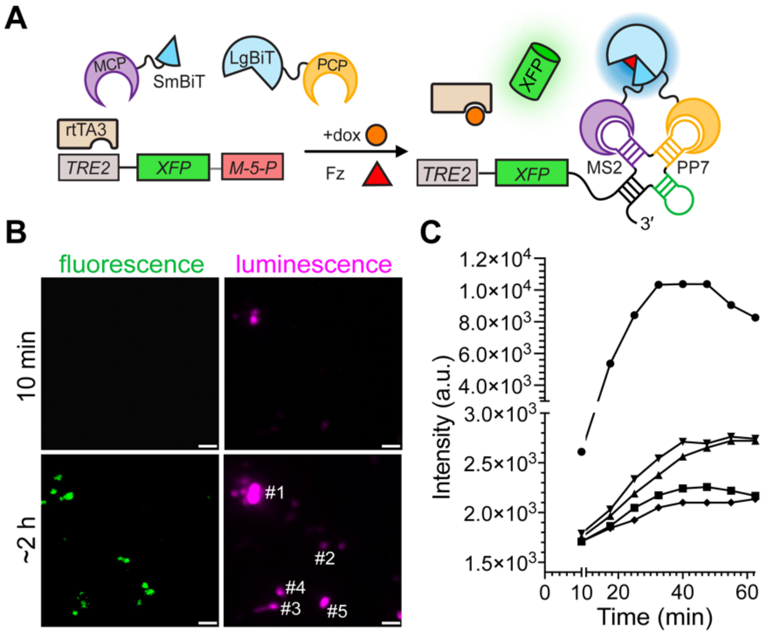

Supplementary Figure 10: Live-cell imaging of gene expression. (**A**) Schematic of doxycycline (dox)-inducible mRNA production. The reporter construct comprised a Tet-on repressor element (*TRE2*) upstream of a sequence encoding mRFP (XFP) with M-5-P in the 3′ UTR. Addition of dox promotes transcription. (**B**) Bioluminescent output from inducible model. HEK293T cells were transfected with plasmids encoding RNA lanterns and *TRE2-XFP-M-5-P*. Bioluminescence was measured for ~1 h (90 s acquisitions, 71 frames) post-dox addition. Fluorescence images were acquired at 10 min and ~2-h post-dox addition. Scale bars = 20 μm. (**C**) Luminescent outputs from individual cells (#1-5) analyzed over time. Source data for B and C are provided as Source Data files.

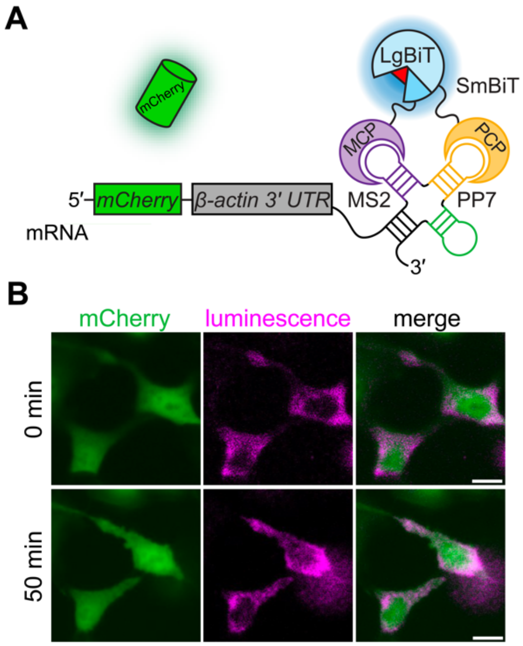

Supplementary Figure 11: Live-cell imaging of *mCherry-β-actin* during cellular stress. (A) Schematic for mRNA imaging during cellular stress. (**B**) Imaging cellular mRNA. HEK293T cells expressing RNA lanterns were transfected with DNA encoding *mCherry-β-actin* (with M-3-P located in the 3′ UTR). Cells were treated with sodium aresenite and imaged before and after treatment (0 and 50 min, respectively). Fluorescence readouts (mCherry) confirmed successful reporter transfection and expression. Scale bars = 20 µm. Source data for B are provided as a Source Data file.

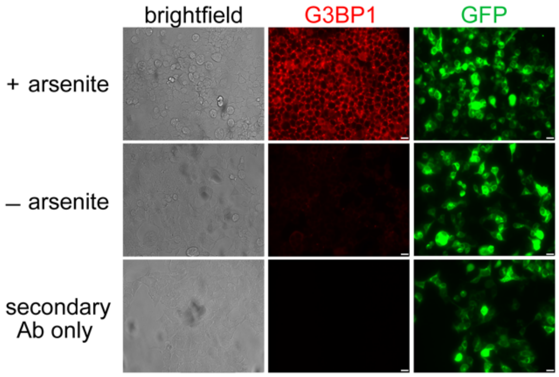

Supplementary Figure 12: G3BP1 expression in stressed cells. HEK293T cells stably expressing RNA lanterns were transfected with DNA encoding *CDK6–M-3-P–IRES-GFP*. Some cells were treated with arsenite (+) to induce cell stress. Cells were fixed and stained with anti-G3BP1 or no antibody (secondary only), followed by a fluorescent secondary antibody. Fluorescence images are shown. Scale bars = 20 µm. Source data are provided as a Source Data file.

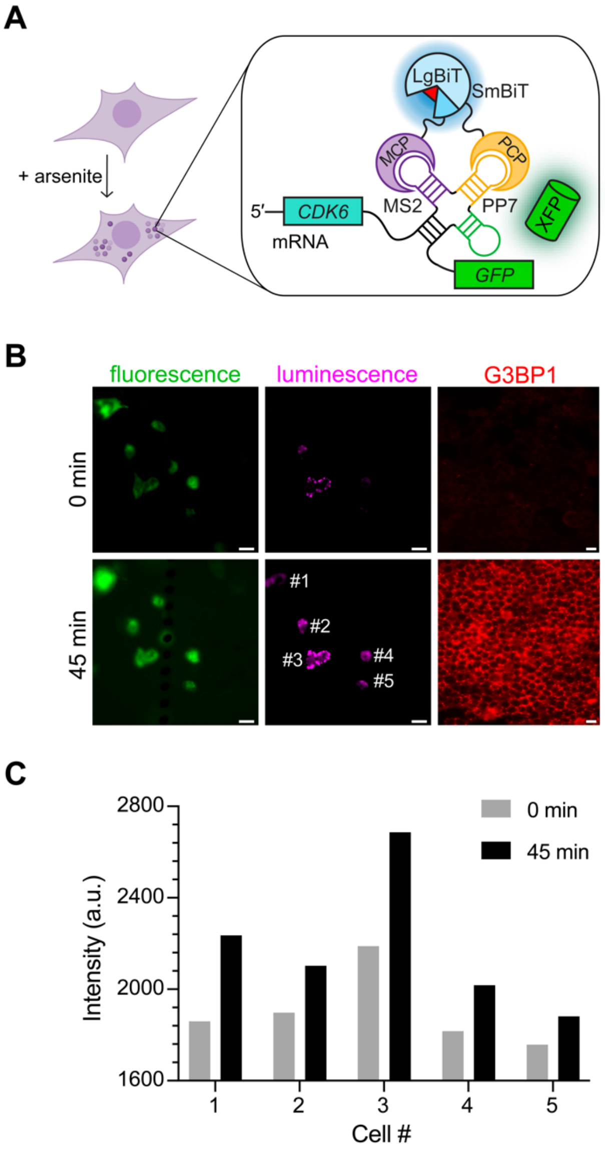

Supplementary Figure 13: Live-cell imaging of *CDK6* during cellular stress. (**A**) Schematic of mRNA trafficking upon arsenite treatment. The cell cartoon was created in BioRender (*Fuller, E. (2024) BioRender.com/p93f09.*) (**B**) Dynamic imaging of mRNAs. HEK293T cells expressing RNA lanterns were transfected with DNA encoding *CDK6-M-3-P-IRES-GFP*. Cells were treated with arsenite as in Fig. S11 and imaged over time. Fluorescence readouts confirmed successful reporter transfection and expression. Scale bars = 20 µm. Stress granule formation was confirmed by staining for G3BP1 expression. (**C**) Luminescent outputs from individual cells (#1-5) at 0 and 45 min. Source data for Supplementary Fig. 13(B) and (C) are provided as a Source Data file.

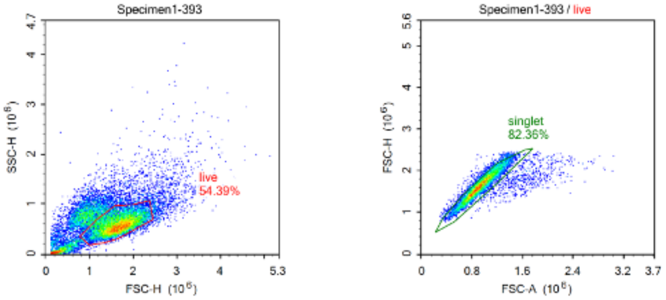

Supplementary Figure 14: Analysis of XFP expression with flow cytometry. Cells were analyzed for XFP expression on a Novocyte 3000 flow cytometer. Live cells were gated using FSC/SSC settings, and singlet cells were further gated. For each sample, 10,000 events were collected on the "singlet cell" gate.

Supplementary Table 1.

Features	Plasmid Vector	Promoter	Expression in	Used in
NLS - HA - MS2 - SmBiT114 - IRES - PP7 - LgBiT - FLAG	pCDNA 3.1/ pLenti 3rd generation	CMV	mammalian	Fig. 1D-F Fig. 2B-F Fig. 3A-B Fig. 4B,D,E Fig. 5B-C Fig. A-D S1 S2 S3 S4 S5 S6 S7 S8 S9 S10 S11 S12 S13 SM1-6
NLS - HA - MS2 - SmBiT99 - IRES - PP7 - LgBiT - FLAG	pCDNA 3.1	CMV	mammalian	S1
NLS - HA - MS2 - 3x SmBiT99 - IRES - PP7 - LgBiT - FLAG	pCDNA 3.1	CMV	mammalian	S1
SV40 NLS - HA - MS2 - 3x SmBiT114 - IRES - PP7 - LgBiT - FLAG	pCDNA 3.1	CMV	mammalian	S1
NLS - HA - MS2 - G4S - SmBiT114 - IRES - PP7 - G4S - LgBiT - FLAG	pCDNA 3.1	CMV	mammalian	S6
NLS - HA - MS2 - G4S - SmBiT114 - IRES - PP7 - G4Sx3 - LgBiT - FLAG	pCDNA 3.1	CMV	mammalian	S6
NLS - HA - MS2 - G4S - SmBiT114 - IRES - PP7 - G4Sx5 - LgBiT - FLAG	pCDNA 3.1	CMV	mammalian	S6
NLS - HA - MS2 - G4Sx3 - SmBiT114 - IRES - PP7 - G4S - LgBiT - FLAG	pCDNA 3.1	CMV	mammalian	S6
NLS - HA - MS2 - G4Sx3 - SmBiT114 - IRES - PP7 - G4Sx3 - LgBiT - FLAG	pCDNA 3.1	CMV	mammalian	S6
NLS - HA - MS2 - G4Sx3 - SmBiT114 - IRES - PP7 - G4Sx5 - LgBiT - FLAG	pCDNA 3.1	CMV	mammalian	S6
NLS - HA - MS2 - G4Sx5 - SmBiT114 - IRES - PP7 - G4S - LgBiT - FLAG	pCDNA 3.1	CMV	mammalian	S6
NLS - HA - MS2 - G4Sx5 - SmBiT114 - IRES - PP7 - G4Sx3 - LgBiT - FLAG	pCDNA 3.1	CMV	mammalian	S6
NLS - HA - MS2 - G4Sx5 - SmBiT114 - IRES - PP7 - G4Sx5 - LgBiT - FLAG	pCDNA 3.1	CMV	mammalian	S6
NLS - HA - MS2 - linker - SmBiT114 - IRES - PP7 - linker - YeLgBiT - FLAG	pCDNA 3.1	CMV	mammalian	Fig. 4G-H
NLS - HA - MS2 - linker - SmBiT114 - IRES - PP7 - linker - LumiLgBiT - FLAG	pCDNA 3.1	CMV	mammalian	Fig. 4G-H
NLS - HA - MS2 - FlucN - IRES - PP7 - FlucC - FLAG	pCDNA 3.1	CMV	mammalian	Fig. 4D-E
BFP - MS2-3-PP7	pCDNA 3.1	CMV	mammalian	Fig. 6C-D S6
BFP - MS2-3-PP7 (mut)	pCDNA 3.1	CMV	mammalian	Fig. 6C-D
Staygold - MS2-3-PP7	pCDNA 3.1	CMV	mammalian	Fig. 5B-C Fig 6A-B S7 S8
Staygold	pCDNA 3.1	CMV	mammalian	Fig. 5B-C Fig. 6A-B
CDK6-MS2-3-PP7-IRES-Staygold	pCDNA 3.1	CMV	mammalian	S12 S13
mCherry-β-actin 3' UTR-MS2-3-PP7	pCDNA 3.1	CMV	mammalian	Fig. 5D S11
TRE2- TagRFP657-MS2-5-PP7- 3' UTR-UBC- loxp- rtTA-Advanced-loxp-IRES	GIPZ	minimal CMV	mammalian	S10
Addgene #135997 pEGFP-C1-G3BP1-WT	pEGFP-C1	CMV	mammalian	Fig. 5D

Supplementary Note 1.
Plasmid, RNA sequences, and RT-qPCR primers:

Plasmid Sequences (CMV enhancer/ promoter)

NLS - HA - MCP - SmBiT114 - IRES - PCP - LgBiT - FLAG
cgttacataacttacggtaaatggcccgcctggctgaccgcccaacgacccccgcccattgacgtcaataatgacgtatgttccca
tagtaacgccaatagggactttccattgacgtcaatgggtggagtatttacggtaaactgcccacttggcagtacatcaagtgtatca
tatgccaagtacgccccctattgacgtcaatgacggtaaatggcccgcctggcattatgcccagtacatgaccttatgggactttcc
tacttggcagtacatctacgtattagtcatcgctattaccatggtgatgcggttttggcagtacatcaatgggcgtggatagcggtttg
actcacggggatttccaagtctccaccccattgacgtcaatgggagtttgttttggcaccaaaatcaacgggactttccaaaatgtc
gtaacaactccgccccattgacgcaaatgggcggtaggcgtgtacggtgggaggtctatataagcagagctctctggctaactag
agaacccactgcttactggcttatcgaaattaatacgactcactatagggagacccaagcttatgggcccaaaaaagaaaagaaa
agttggctacccctacgacgtgcccgactacgccatcgaaggccgccatatgctagccgttaaaatggcttctaactttactcagtt
cgttctcgtcgacaatggcggaactggcgacgtgactgtcgccccaagcaacttcgctaacgggatcgctgaatggatcagctct
aactcgcgttcacaggcttacaaagtaacctgtagcgttcgtcagagctctgcgcagaatcgcaaatacaccatcaaagtcgaggt
gcctaaaggcgcctggcgttcgtacttaaatatggaactaaccattccaattttcgccacgaattccgactgcgagcttattgttaag
gcaatgcaaggtctcctaaaagatggaaacccgattccctcagcaatcgcagcaaactccggcatctacgcggattctagacgg
gatcccgccaccgtgaccggctaccgcctgtttgaggagattctgtaacgtacgacgcgtggatccctccccccccctaacgtt
actggccgaagccgcttggaataaggccggtgtgcgtttgtctatatgttattttccaccatattgccgtcttttggcaatgtgagggc
ccggaaacctggccctgtcttcttgacgagcattcctaggggtctttcccctctcgccaaaggaatgcaaggtctgttgaatgtcgt
gaaggaagcagttcctctggaagcttcttgaagacaaacaacgtctgtagcgaccctttgcaggcagcggaaccccccacctgg
cgacaggtgcctctgcggccaaaagccacgtgtataagatacacctgcaaaggcggcacaaccccagtgccacgttgtgagttg
gatagttgtggaaagagtcaaatggctctcctcaagcgtattcaacaaggggctgaaggatgcccagaaggtaccccattgtatg
ggatctgatctggggcctcggtgcacatgctttacatgtgtttagtcgaggttaaaaaaacgtctaggccccccgaaccacgggga
cgtggttttcctttgaaaaacacgatgataatatggccacagcctgcaggatgggctccaaaaccatcgttctttcggtcggcgagg
ctactcgcactctgactgagatccagtccaccgcagaccgtcagatcttcgaagagaaggtcgggcctctggtgggtcggctgc
gcctcacggcttcgctccgtcaaaacggagccaagaccgcgtatcgcgtcaacctaaaactggatcaggcggacgtcgttgatt
ccggacttccgaaagtgcgctacactcaggtatggtcgcacgacgtgacaatcgttgcgaatagcaccgaggcctcgcgcaaat
cgttgtacgatttgaccaagtccctcgtcgcgacctcgcaggtcgaagatcttgtcgtcaaccttgtgccgctgggccgtgcggat
ccaccggtatgcatcgccaccgccttcacactcgaagatttcgttggggactgggaacagacagccgcctacaacctggaccaa
gtccttgaacagggaggtgtgtccagtttgctgcagaatctcgccgtgtccgtaactccgatccaaaggattgtccggagcggtga
aaatgccctgaagatcgacatccatgtcatcatcccgtatgaaggtctgagcgccgaccaaatggcccagatcgaagaggtgttt
aaggtggtgtaccctgtggatgatcatcactttaaggtgatcctgccctatggcacactggtaatcgacggggttacgccgaacat
gctgaactatttcggacggccgtatgaaggcatcgccgtgttcgacggcaaaaagatcactgtaacagggaccctgtggaacgg
caacaaaattatcgacgagcgcctgatcacccccgacggctccatgctgttccgagtaaccatcaacagcctcgaggactacaa
ggacgacgatgacaag

NLS - HA - MCP - SmBiT99 - IRES - PCP - LgBiT – FLAG
cgttacataacttacggtaaatggcccgcctggctgaccgcccaacgacccccgcccattgacgtcaataatgacgtatgttccca
tagtaacgccaatagggactttccattgacgtcaatgggtggagtatttacggtaaactgcccacttggcagtacatcaagtgtatca
tatgccaagtacgccccctattgacgtcaatgacggtaaatggcccgcctggcattatgcccagtacatgaccttatgggactttcc
tacttggcagtacatctacgtattagtcatcgctattaccatggtgatgcggttttggcagtacatcaatgggcgtggatagcggtttg
actcacggggatttccaagtctccaccccattgacgtcaatgggagtttgttttggcaccaaaatcaacgggactttccaaaatgtc
gtaacaactccgccccattgacgcaaatgggcggtaggcgtgtacggtgggaggtctatataagcagagctctctggctaactag
agaacccactgcttactggcttatcgaaattaatacgactcactatagggagacccaagcttatgggcccaaaaaagaaaagaaa

agttggctacccctacgacgtgcccgactacgccatcgaaggccgccatatgctagccgttaaaatggcttctaactttactcagtt
cgttctcgtcgacaatggcggaactggcgacgtgactgtcgccccaagcaacttcgctaacgggatcgctgaatggatcagctct
aactcgcgttcacaggcttacaaagtaacctgtagcgttcgtcagagctctgcgcagaatcgcaaatacaccatcaaagtcgaggt
gcctaaaggcgcctggcgttcgtacttaaatatggaactaaccattccaattttcgccacgaattccgactgcgagcttattgttaag
gcaatgcaaggtctcctaaaagatggaaacccgattccctcagcaatcgcagcaaactccggcatctacgcggattctagacgg
gatcccgccaccgtgaccggctaccgcctgtttgagaaaattagctaacgtacgacgcgtggatccctcccccccccctaacgtt
actggccgaagccgcttggaataaggccggtgtgcgtttgtctatatgttattttccaccatattgccgtcttttggcaatgtgagggc
ccggaaacctggccctgtcttcttgacgagcattcctaggggtctttcccctctcgccaaaggaatgcaaggtctgttgaatgtcgt
gaaggaagcagttcctctggaagcttcttgaagacaaacaacgtctgtagcgaccctttgcaggcagcggaaccccccacctgg
cgacaggtgcctctgcggccaaaagccacgtgtataagatacacctgcaaaggcggcacaaccccagtgccacgttgtgagttg
gatagttgtggaaagagtcaaatggctctcctcaagcgtattcaacaaggggctgaaggatgcccagaaggtaccccattgtatg
ggatctgatctggggcctcggtgcacatgctttacatgtgtttagtcgaggttaaaaaaacgtctaggccccccgaaccacgggga
cgtggttttcctttgaaaaacacgatgataatatggccacagcctgcaggatgggctccaaaaccatcgttctttcggtcggcgagg
ctactcgcactctgactgagatccagtccaccgcagaccgtcagatcttcgaagagaaggtcgggcctctggtgggtcggctgc
gcctcacggcttcgctccgtcaaaacggagccaagaccgcgtatcgcgtcaacctaaaactggatcaggcggacgtcgttgatt
ccggacttccgaaagtgcgctacactcaggtatggtcgcacgacgtgacaatcgttgcgaatagcaccgaggcctcgcgcaaat
cgttgtacgatttgaccaagtccctcgtcgcgacctcgcaggtcgaagatcttgtcgtcaaccttgtgccgctgggccgtgcggat
ccaccggtatgcatcgccaccgccttcacactcgaagatttcgttggggactgggaacagacagccgcctacaacctggaccaa
gtccttgaacagggaggtgtgtccagtttgctgcagaatctcgccgtgtccgtaactccgatccaaaggattgtccggagcggtga
aaatgccctgaagatcgacatccatgtcatcatcccgtatgaaggtctgagcgccgaccaaatggcccagatcgaagaggtgttt
aaggtggtgtaccctgtggatgatcatcactttaaggtgatcctgccctatggcacactggtaatcgacggggttacgccgaacat
gctgaactatttcggacggccgtatgaaggcatcgccgtgttcgacggcaaaaagatcactgtaacagggaccctgtggaacgg
caacaaaattatcgacgagcgcctgatcacccccgacggctccatgctgttccgagtaaccatcaacagcctcgaggactacaa
ggacgacgatgacaa

NLS - HA - MCP - 3X SmBiT99 - IRES - PCP - LgBiT – FLAG

cgttacataacttacggtaaatggcccgcctggctgaccgcccaacgacccccgcccattgacgtcaataatgacgtatgttccca
tagtaacgccaatagggactttccattgacgtcaatgggtggagtatttacggtaaactgcccacttggcagtacatcaagtgtatca
tatgccaagtacgccccctattgacgtcaatgacggtaaatggcccgcctggcattatgcccagtacatgaccttatgggactttcc
tacttggcagtacatctacgtattagtcatcgctattaccatggtgatgcggttttggcagtacatcaatgggcgtggatagcggtttg
actcacggggatttccaagtctccaccccattgacgtcaatgggagtttgttttggcaccaaaatcaacgggactttccaaaatgtc
gtaacaactccgccccattgacgcaaatgggcggtaggcgtgtacggtgggaggtctatataagcagagctctctggctaactag
agaacccactgcttactggcttatcgaaattaatacgactcactatagggagacccaagcttatgggcccaaaaaagaaaagaaa
agttggctacccctacgacgtgcccgactacgccatcgaaggccgccatatgctagccgttaaaatggcttctaactttactcagtt
cgttctcgtcgacaatggcggaactggcgacgtgactgtcgccccaagcaacttcgctaacgggatcgctgaatggatcagctct
aactcgcgttcacaggcttacaaagtaacctgtagcgttcgtcagagctctgcgcagaatcgcaaatacaccatcaaagtcgaggt
gcctaaaggcgcctggcgttcgtacttaaatatggaactaaccattccaattttcgccacgaattccgactgcgagcttattgttaag
gcaatgcaaggtctcctaaaagatggaaacccgattccctcagcaatcgcagcaaactccggcatctacgcggattctagacgg
gatcccgccaccgtgaccggctaccgcctgtttgagaaaattagcgggagcggaagtggggttaccggttatcgtctgttcgaaa
aaatcagcgggtccggatcgggtgtgacaggctatcgtctgtttgagaaaatttcctaacgtacgacgcgtggatccctccccccc
ccctaacgttactggccgaagccgcttggaataaggccggtgtgcgtttgtctatatgttattttccaccatattgccgtcttttggcaa
tgtgagggcccggaaacctggccctgtcttcttgacgagcattcctaggggtctttcccctctcgccaaaggaatgcaaggtctgtt
gaatgtcgtgaaggaagcagttcctctggaagcttcttgaagacaaacaacgtctgtagcgaccctttgcaggcagcggaacccc
ccacctggcgacaggtgcctctgcggccaaaagccacgtgtataagatacacctgcaaaggcggcacaaccccagtgccacgt
tgtgagttggatagttgtggaaagagtcaaatggctctcctcaagcgtattcaacaaggggctgaaggatgcccagaaggtaccc
cattgtatgggatctgatctggggcctcggtgcacatgctttacatgtgtttagtcgaggttaaaaaaacgtctaggccccccgaac
cacggggacgtggttttcctttgaaaaacacgatgataatatggccacagcctgcaggatgggctccaaaaccatcgttctttcggt
cggcgaggctactcgcactctgactgagatccagtccaccgcagaccgtcagatcttcgaagagaaggtcgggcctctggtgg

gtcggctgcgcctcacggcttcgctccgtcaaaacggagccaagaccgcgtatcgcgtcaacctaaaactggatcaggcggac
gtcgttgattccggacttccgaaagtgcgctacactcaggtatggtcgcacgacgtgacaatcgttgcgaatagcaccgaggcct
cgcgcaaatcgttgtacgatttgaccaagtccctcgtcgcgacctcgcaggtcgaagatcttgtcgtcaaccttgtgccgctgggc
cgtgcggatccaccggtatgcatcgccaccgccttcacactcgaagatttcgttggggactgggaacagacagccgcctacaac
ctggaccaagtccttgaacagggaggtgtgtccagtttgctgcagaatctcgccgtgtccgtaactccgatccaaaggattgtccg
gagcggtgaaaatgccctgaagatcgacatccatgtcatcatcccgtatgaaggtctgagcgccgaccaaatggcccagatcga
agaggtgtttaaggtggtgtaccctgtggatgatcatcactttaaggtgatcctgccctatggcacactggtaatcgacggggttac
gccgaacatgctgaactatttcggacggccgtatgaaggcatcgccgtgttcgacggcaaaaagatcactgtaacagggaccct
gtggaacggcaacaaaattatcgacgagcgcctgatcacccccgacggctccatgctgttccgagtaaccatcaacagcctcga
ggactacaaggacgacgatgacaag

SV40 NLS - HA - MCP - 3x SmBiT114 - IRES - PCP - LgBiT – FLAG

cgttacataacttacggtaaatggcccgcctggctgaccgcccaacgacccccgcccattgacgtcaataatgacgtatgttccca
tagtaacgccaatagggactttccattgacgtcaatgggtggagtatttacggtaaactgcccacttggcagtacatcaagtgtatca
tatgccaagtacgccccctattgacgtcaatgacggtaaatggcccgcctggcattatgcccagtacatgaccttatgggactttcc
tacttggcagtacatctacgtattagtcatcgctattaccatggtgatgcggttttggcagtacatcaatgggcgtggatagcggtttg
actcacggggatttccaagtctccaccccattgacgtcaatgggagtttgttttggcaccaaaatcaacgggactttccaaaatgtc
gtaacaactccgccccattgacgcaaatgggcggtaggcgtgtacggtgggaggtctatataagcagagctctctggctaactag
agaacccactgcttactggcttatcgaaattaatacgactcactatagggagacccaagcttatgggcccaaaaaagaaaagaaa
agttggctacccctacgacgtgcccgactacgccatcgaaggccgccatatgctagccgttaaaatggcttctaactttactcagtt
cgttctcgtcgacaatggcggaactggcgacgtgactgtcgccccaagcaacttcgctaacgggatcgctgaatggatcagctct
aactcgcgttcacaggcttacaaagtaacctgtagcgttcgtcagagctctgcgcagaatcgcaaatacaccatcaaagtcgaggt
gcctaaaggcgcctggcgttcgtacttaaatatggaactaaccattccaattttcgccacgaattccgactgcgagcttattgttaag
gcaatgcaaggtctcctaaaagatggaaacccgattccctcagcaatcgcagcaaactccggcatctacgcggattctagacgg
gatcccgccaccgtgaccggctaccgcctgtttgaggagattctggggagcggaagtggggtgaccggatatcggctgtttgag
gagattctggggtccggatcgggtgtgacagggtatcgcctgttcgaggaaatcctgtaacgtacgacgcgtggatccctcccc
cccctaacgttactggccgaagccgcttggaataaggccggtgtgcgtttgtctatatgttattttccaccatattgccgtcttttggc
aatgtgagggcccggaaacctggccctgtcttcttgacgagcattcctaggggtctttcccctctcgccaaaggaatgcaaggtct
gttgaatgtcgtgaaggaagcagttcctctggaagcttcttgaagacaaacaacgtctgtagcgaccctttgcaggcagcggaac
cccccacctggcgacaggtgcctctgcggccaaaagccacgtgtataagatacacctgcaaaggcggcacaaccccagtgcc
acgttgtgagttggatagttgtggaaagagtcaaatggctctcctcaagcgtattcaacaaggggctgaaggatgcccagaaggt
accccattgtatgggatctgatctggggcctcggtgcacatgctttacatgtgtttagtcgaggttaaaaaaacgtctaggccccc
gaaccacggggacgtggttttcctttgaaaaacacgatgataatatggccacagcctgcaggatgggctccaaaaccatcgttctt
tcggtcggcgaggctactcgcactctgactgagatccagtccaccgcagaccgtcagatcttcgaagagaaggtcgggcctctg
gtgggtcggctgcgcctcacggcttcgctccgtcaaaacggagccaagaccgcgtatcgcgtcaacctaaaactggatcaggc
ggacgtcgttgattccggacttccgaaagtgcgctacactcaggtatggtcgcacgacgtgacaatcgttgcgaatagcaccgag
gcctcgcgcaaatcgttgtacgatttgaccaagtccctcgtcgcgacctcgcaggtcgaagatcttgtcgtcaaccttgtgccgctg
ggccgtgcggatccaccggtatgcatcgccaccgccttcacactcgaagatttcgttggggactgggaacagacagccgcctac
aacctggaccaagtccttgaacagggaggtgtgtccagtttgctgcagaatctcgccgtgtccgtaactccgatccaaaggattgt
ccggagcggtgaaaatgccctgaagatcgacatccatgtcatcatcccgtatgaaggtctgagcgccgaccaaatggcccagat
cgaagaggtgtttaaggtggtgtaccctgtggatgatcatcactttaaggtgatcctgccctatggcacactggtaatcgacggggt
tacgccgaacatgctgaactatttcggacggccgtatgaaggcatcgccgtgttcgacggcaaaaagatcactgtaacagggac
cctgtggaacggcaacaaaattatcgacgagcgcctgatcacccccgacggctccatgctgttccgagtaaccatcaacagcctc
gaggactacaaggacgacgatgacaag

NLS - HA - MCP - G4S - SmBiT114 - IRES - PCP - G4S - LgBiT – FLAG

cgttacataacttacggtaaatggcccgcctggctgaccgcccaacgacccccgcccattgacgtcaataatgacgtatgttccca
tagtaacgccaatagggactttccattgacgtcaatgggtggagtatttacggtaaactgcccacttggcagtacatcaagtgtatca
tatgccaagtacgccccctattgacgtcaatgacggtaaatggcccgcctggcattatgcccagtacatgaccttatgggactttcc

tacttggcagtacatctacgtattagtcatcgctattaccatggtgatgcggttttggcagtacatcaatgggcgtggatagcggtttg
actcacggggatttccaagtctccaccccattgacgtcaatgggagtttgttttggcaccaaaatcaacgggactttccaaaatgtc
gtaacaactccgccccattgacgcaaatgggcggtaggcgtgtacggtgggaggtctatataagcagagctctctggctaactag
agaacccactgcttactggcttatcgaaattaatacgactcactatagggagacccaagcttatgggcccaaaaaagaaaagaaa
agttggctacccctacgacgtgcccgactacgccatcgaaggccgccatatgctagccgttaaaatggcttctaactttactcagtt
cgttctcgtcgacaatggcggaactggcgacgtgactgtcgccccaagcaacttcgctaacgggatcgctgaatggatcagctct
aactcgcgttcacaggcttacaaagtaacctgtagcgttcgtcagagctctgcgcagaatcgcaaatacaccatcaaagtcgaggt
gcctaaaggcgcctggcgttcgtacttaaatatggaactaaccattccaattttcgccacgaattccgactgcgagcttattgttaag
gcaatgcaaggtctcctaaaagatggaaacccgattccctcagcaatcgcagcaaactccggcatctacgatatcggcggcgga
ggctctgtgaccggctaccgcctgtttgaggagattctgtaacgtacgacgcgtggatccctcccccccccctaacgttactggcc
gaagccgcttggaataaggccggtgtgcgtttgtctatatgttattttccaccatattgccgtcttttggcaatgtgagggcccggaaa
cctggccctgtcttcttgacgagcattcctaggggtctttcccctctcgccaaaggaatgcaaggtctgttgaatgtcgtgaaggaa
gcagttcctctggaagcttcttgaagacaaacaacgtctgtagcgaccctttgcaggcagcggaaccccccacctggcgacagg
tgcctctgcggccaaaagccacgtgtataagatacacctgcaaaggcggcacaaccccagtgccacgttgtgagttggatagttg
tggaaagagtcaaatggctctcctcaagcgtattcaacaaggggctgaaggatgcccagaaggtaccccattgtatgggatctga
tctggggcctcggtgcacatgctttacatgtgtttagtcgaggttaaaaaaacgtctaggccccccgaaccacggggacgtggtttt
cctttgaaaaacacgatgataatatggccacagcctgcaggatgggctccaaaaccatcgttctttcggtcggcgaggctactcgc
actctgactgagatccagtccaccgcagaccgtcagatcttcgaagagaaggtcgggcctctggtgggtcggctgcgcctcacg
gcttcgctccgtcaaaacggagccaagaccgcgtatcgcgtcaacctaaaactggatcaggcggacgtcgttgattccggacttc
cgaaagtgcgctacactcaggtatggtcgcacgacgtgacaatcgttgcgaatagcaccgaggcctcgcgcaaatcgttgtacg
atttgaccaagtccctcgtcgcgacctcgcaggtcgaagatcttgtcgtcaaccttgtgccgctggccgtatcgatggcggagg
cgggtctttcacactcgaagatttcgttggggactgggaacagacagccgcctacaacctggaccaagtccttgaacagggagg
tgtgtccagtttgctgcagaatctcgccgtgtccgtaactccgatccaaaggattgtccggagcggtgaaaatgccctgaagatcg
acatccatgtcatcatcccgtatgaaggtctgagcgccgaccaaatggcccagatcgaagaggtgtttaaggtggtgtaccctgtg
gatgatcatcactttaaggtgatcctgccctatggcacactggtaatcgacggggttacgccgaacatgctgaactatttcggacgg
ccgtatgaaggcatcgccgtgttcgacggcaaaaagatcactgtaacagggaccctgtggaacggcaacaaaattatcgacgag
cgcctgatcacccccgacggctccatgctgttccgagtaaccatcaacagcctcgaggactacaaggacgacgatgacaag

NLS - HA - MCP - G4S - SmBiT114 - IRES - PCP - G4Sx3 - LgBiT – FLAG

cgttacataacttacggtaaatggcccgcctggctgaccgcccaacgacccccgcccattgacgtcaataatgacgtatgttccca
tagtaacgccaatagggactttccattgacgtcaatgggtggagtatttacggtaaactgcccacttggcagtacatcaagtgtatca
tatgccaagtacgccccctattgacgtcaatgacggtaaatggcccgcctggcattatgcccagtacatgaccttatgggactttcc
tacttggcagtacatctacgtattagtcatcgctattaccatggtgatgcggttttggcagtacatcaatgggcgtggatagcggtttg
actcacggggatttccaagtctccaccccattgacgtcaatgggagtttgttttggcaccaaaatcaacgggactttccaaaatgtc
gtaacaactccgccccattgacgcaaatgggcggtaggcgtgtacggtgggaggtctatataagcagagctctctggctaactag
agaacccactgcttactggcttatcgaaattaatacgactcactatagggagacccaagcttatgggcccaaaaaagaaaagaaa
agttggctacccctacgacgtgcccgactacgccatcgaaggccgccatatgctagccgttaaaatggcttctaactttactcagtt
cgttctcgtcgacaatggcggaactggcgacgtgactgtcgccccaagcaacttcgctaacgggatcgctgaatggatcagctct
aactcgcgttcacaggcttacaaagtaacctgtagcgttcgtcagagctctgcgcagaatcgcaaatacaccatcaaagtcgaggt
gcctaaaggcgcctggcgttcgtacttaaatatggaactaaccattccaattttcgccacgaattccgactgcgagcttattgttaag
gcaatgcaaggtctcctaaaagatggaaacccgattccctcagcaatcgcagcaaactccggcatctacgatatcggcggcgga
ggctctgtgaccggctaccgcctgtttgaggagattctgtaacgtacgacgcgtggatccctcccccccccctaacgttactggcc
gaagccgcttggaataaggccggtgtgcgtttgtctatatgttattttccaccatattgccgtcttttggcaatgtgagggcccggaaa
cctggccctgtcttcttgacgagcattcctaggggtctttcccctctcgccaaaggaatgcaaggtctgttgaatgtcgtgaaggaa
gcagttcctctggaagcttcttgaagacaaacaacgtctgtagcgaccctttgcaggcagcggaaccccccacctggcgacagg
tgcctctgcggccaaaagccacgtgtataagatacacctgcaaaggcggcacaaccccagtgccacgttgtgagttggatagttg
tggaaagagtcaaatggctctcctcaagcgtattcaacaaggggctgaaggatgcccagaaggtaccccattgtatgggatctga
tctggggcctcggtgcacatgctttacatgtgtttagtcgaggttaaaaaaacgtctaggccccccgaaccacggggacgtggtttt

cctttgaaaaacacgatgataatatggccacagcctgcaggatgggctccaaaaccatcgttctttcggtcggcgaggctactcgc
actctgactgagatccagtccaccgcagaccgtcagatcttcgaagagaaggtcgggcctctggtgggtcggctgcgcctcacg
gcttcgctccgtcaaaacggagccaagaccgcgtatcgcgtcaacctaaaactggatcaggcggacgtcgttgattccggacttc
cgaaagtgcgctacactcaggtatggtcgcacgacgtgacaatcgttgcgaatagcaccgaggcctcgcgcaaatcgttgtacg
atttgaccaagtccctcgtcgcgacctcgcaggtcgaagatcttgtcgtcaaccttgtgccgctgggccgtatcgatggcggagg
cgggtctggcggcggcggcagcggtggaggaggttctttcacactcgaagatttcgttggggactgggaacagacagccgcct
acaacctggaccaagtccttgaacagggaggtgtgtccagtttgctgcagaatctcgccgtgtccgtaactccgatccaaaggatt
gtccggagcggtgaaaatgccctgaagatcgacatccatgtcatcatcccgtatgaaggtctgagcgccgaccaaatggcccag
atcgaagaggtgtttaaggtggtgtaccctgtggatgatcatcactttaaggtgatcctgccctatggcacactggtaatcgacggg
gttacgccgaacatgctgaactatttcggacggccgtatgaaggcatcgccgtgttcgacggcaaaaagatcactgtaacaggga
ccctgtggaacggcaacaaaattatcgacgagcgcctgatcacccccgacggctccatgctgttccgagtaaccatcaacagcct
cgaggactacaaggacgacgatgacaag

NLS - HA - MCP - G4S - SmBiT114 - IRES - PCP - G4Sx5 - LgBiT – FLAG

cgttacataacttacggtaaatggcccgcctggctgaccgcccaacgacccccgcccattgacgtcaataatgacgtatgttccca
tagtaacgccaatagggactttccattgacgtcaatgggtggagtatttacggtaaactgcccacttggcagtacatcaagtgtatca
tatgccaagtacgccccctattgacgtcaatgacggtaaatggcccgcctggcattatgcccagtacatgaccttatgggactttcc
tacttggcagtacatctacgtattagtcatcgctattaccatggtgatgcggttttggcagtacatcaatgggcgtggatagcggtttg
actcacggggatttccaagtctccaccccattgacgtcaatgggagtttgttttggcaccaaaatcaacgggactttccaaaatgtc
gtaacaactccgccccattgacgcaaatgggcggtaggcgtgtacggtgggaggtctatataagcagagctctctggctaactag
agaacccactgcttactggcttatcgaaattaatacgactcactatagggagacccaagcttatgggcccaaaaaagaaaagaaa
agttggctacccctacgacgtgcccgactacgccatcgaaggccgccatatgctagccgttaaaatggcttctaactttactcagtt
cgttctcgtcgacaatggcggaactggcgacgtgactgtcgccccaagcaacttcgctaacgggatcgctgaatggatcagctct
aactcgcgttcacaggcttacaaagtaacctgtagcgttcgtcagagctctgcgcagaatcgcaaatacaccatcaaagtcgaggt
gcctaaaggcgcctggcgttcgtacttaaatatggaactaaccattccaattttcgccacgaattccgactgcgagcttattgttaag
gcaatgcaaggtctcctaaaagatggaaacccgattccctcagcaatcgcagcaaactccggcatctacgatatcggcggcgga
ggctctgtgaccggctaccgcctgtttgaggagattctgtaacgtacgacgcgtggatccctcccccccccctaacgttactggcc
gaagccgcttggaataaggccggtgtgcgtttgtctatatgttattttccaccatattgccgtcttttggcaatgtgagggcccggaaa
cctggccctgtcttcttgacgagcattcctaggggtctttcccctctcgccaaaggaatgcaaggtctgttgaatgtcgtgaaggaa
gcagttcctctggaagcttcttgaagacaaacaacgtctgtagcgaccctttgcaggcagcggaaccccccacctggcgacagg
tgcctctgcggccaaaagccacgtgtataagatacacctgcaaaggcggcacaaccccagtgccacgttgtgagttggatagttg
tggaaagagtcaaatggctctcctcaagcgtattcaacaaggggctgaaggatgcccagaaggtaccccattgtatgggatctga
tctggggcctcggtgcacatgctttacatgtgtttagtcgaggttaaaaaaacgtctaggccccccgaaccacggggacgtggtttt
cctttgaaaaacacgatgataatatggccacagcctgcaggatgggctccaaaaccatcgttctttcggtcggcgaggctactcgc
actctgactgagatccagtccaccgcagaccgtcagatcttcgaagagaaggtcgggcctctggtgggtcggctgcgcctcacg
gcttcgctccgtcaaaacggagccaagaccgcgtatcgcgtcaacctaaaactggatcaggcggacgtcgttgattccggacttc
cgaaagtgcgctacactcaggtatggtcgcacgacgtgacaatcgttgcgaatagcaccgaggcctcgcgcaaatcgttgtacg
atttgaccaagtccctcgtcgcgacctcgcaggtcgaagatcttgtcgtcaaccttgtgccgctgggccgtatcgatggcggagg
cgggtctggtggagggggatctggaggtggtgggtctggcggcggcggcagcggtggaggaggttctttcacactcgaagatt
tcgttggggactgggaacagacagccgcctacaacctggaccaagtccttgaacagggaggtgtgtccagtttgctgcagaatct
cgccgtgtccgtaactccgatccaaaggattgtccggagcggtgaaaatgccctgaagatcgacatccatgtcatcatcccgtatg
aaggtctgagcgccgaccaaatggcccagatcgaagaggtgtttaaggtggtgtaccctgtggatgatcatcactttaaggtgatc
ctgccctatggcacactggtaatcgacggggttacgccgaacatgctgaactatttcggacggccgtatgaaggcatcgccgtgtt
cgacggcaaaaagatcactgtaacagggaccctgtggaacggcaacaaaattatcgacgagcgcctgatcacccccgacggct
ccatgctgttccgagtaaccatcaacagcctcgaggactacaaggacgacgatgacaag

NLS - HA - MCP - G4SX3 - SmBiT114 - IRES – PCP - G4S - LgBiT – FLAG

cgttacataacttacggtaaatggcccgcctggctgaccgcccaacgacccccgcccattgacgtcaataatgacgtatgttccca
tagtaacgccaatagggactttccattgacgtcaatgggtggagtatttacggtaaactgcccacttggcagtacatcaagtgtatca

tatgccaagtacgccccctattgacgtcaatgacggtaaatggcccgcctggcattatgcccagtacatgaccttatgggactttcc
tacttggcagtacatctacgtattagtcatcgctattaccatggtgatgcggttttggcagtacatcaatgggcgtggatagcggtttg
actcacggggatttccaagtctccaccccattgacgtcaatgggagtttgttttggcaccaaaatcaacgggactttccaaaatgtc
gtaacaactccgccccattgacgcaaatgggcggtaggcgtgtacggtgggaggtctatataagcagagctctctggctaactag
agaacccactgcttactggcttatcgaaattaatacgactcactatagggagacccaagcttatgggcccaaaaaagaaaagaaa
agttggctacccctacgacgtgcccgactacgccatcgaaggccgccatatgctagccgttaaaatggcttctaactttactcagtt
cgttctcgtcgacaatggcggaactggcgacgtgactgtcgccccaagcaacttcgctaacgggatcgctgaatggatcagctct
aactcgcgttcacaggcttacaaagtaacctgtagcgttcgtcagagctctgcgcagaatcgcaaatacaccatcaaagtcgaggt
gcctaaaggcgcctggcgttcgtacttaaatatggaactaaccattccaattttcgccacgaattccgactgcgagcttattgttaag
gcaatgcaaggtctcctaaaagatggaaacccgattccctcagcaatcgcagcaaactccggcatctacgatatcggcggcgga
ggctctggaggcggcggaagcggtgggggtgggagcgtgaccggctaccgcctgtttgaggagattctgtaacgtacgacgc
gtggatccctcccccccccctaacgttactggccgaagccgcttggaataaggccggtgtgcgtttgtctatatgttattttccacca
tattgccgtcttttggcaatgtgagggcccggaaacctggccctgtcttcttgacgagcattcctaggggtctttcccctctcgccaa
aggaatgcaaggtctgttgaatgtcgtgaaggaagcagttcctctggaagcttcttgaagacaaacaacgtctgtagcgaccctttt
gcaggcagcggaacccccacctggcgacaggtgcctctgcggccaaaagccacgtgtataagatacacctgcaaaggcggc
acaaccccagtgccacgttgtgagttggatagttgtggaaagagtcaaatggctctcctcaagcgtattcaacaaggggctgaag
gatgcccagaaggtaccccattgtatgggatctgatctggggcctcggtgcacatgctttacatgtgtttagtcgaggttaaaaaaa
cgtctaggccccccgaaccacggggacgtggttttcctttgaaaaacacgatgataatatggccacagcctgcaggatgggctcc
aaaaccatcgttctttcggtcggcgaggctactcgcactctgactgagatccagtccaccgcagaccgtcagatcttcgaagaga
aggtcgggcctctggtgggtcggctgcgcctcacggcttcgctccgtcaaaacggagccaagaccgcgtatcgcgtcaacctaa
aactggatcaggcggacgtcgttgattccggacttccgaaagtgcgctacactcaggtatggtcgcacgacgtgacaatcgttgc
gaatagcaccgaggcctcgcgcaaatcgttgtacgatttgaccaagtccctcgtcgcgacctcgcaggtcgaagatcttgtcgtca
accttgtgccgctgggccgtatcgatggcggaggcgggtctttcacactcgaagatttcgttggggactgggaacagacagccg
cctacaacctggaccaagtccttgaacagggaggtgtgtccagtttgctgcagaatctcgccgtgtccgtaactccgatccaaagg
attgtccggagcggtgaaaatgccctgaagatcgacatccatgtcatcatcccgtatgaaggtctgagcgccgaccaaatggccc
agatcgaagaggtgtttaaggtggtgtaccctgtggatgatcatcactttaaggtgatcctgccctatggcacactggtaatcgacg
gggttacgccgaacatgctgaactatttcggacggccgtatgaaggcatcgccgtgttcgacggcaaaaagatcactgtaacag
ggaccctgtggaacggcaacaaaattatcgacgagcgcctgatcacccccgacggctccatgctgttccgagtaaccatcaaca
gcctcgaggactacaaggacgacgatgacaag

NLS - HA - MCP - G4Sx3 - SmBiT114 - IRES – PCP - G4Sx3 - LgBiT – FLAG

cgttacataacttacggtaaatggcccgcctggctgaccgcccaacgacccccgcccattgacgtcaataatgacgtatgttccca
tagtaacgccaatagggactttccattgacgtcaatgggtggagtatttacggtaaactgcccacttggcagtacatcaagtgtatca
tatgccaagtacgccccctattgacgtcaatgacggtaaatggcccgcctggcattatgcccagtacatgaccttatgggactttcc
tacttggcagtacatctacgtattagtcatcgctattaccatggtgatgcggttttggcagtacatcaatgggcgtggatagcggtttg
actcacggggatttccaagtctccaccccattgacgtcaatgggagtttgttttggcaccaaaatcaacgggactttccaaaatgtc
gtaacaactccgccccattgacgcaaatgggcggtaggcgtgtacggtgggaggtctatataagcagagctctctggctaactag
agaacccactgcttactggcttatcgaaattaatacgactcactatagggagacccaagcttatgggcccaaaaaagaaaagaaa
agttggctacccctacgacgtgcccgactacgccatcgaaggccgccatatgctagccgttaaaatggcttctaactttactcagtt
cgttctcgtcgacaatggcggaactggcgacgtgactgtcgccccaagcaacttcgctaacgggatcgctgaatggatcagctct
aactcgcgttcacaggcttacaaagtaacctgtagcgttcgtcagagctctgcgcagaatcgcaaatacaccatcaaagtcgaggt
gcctaaaggcgcctggcgttcgtacttaaatatggaactaaccattccaattttcgccacgaattccgactgcgagcttattgttaag
gcaatgcaaggtctcctaaaagatggaaacccgattccctcagcaatcgcagcaaactccggcatctacgatatcggcggcgga
ggctctggaggcggcggaagcggtgggggtgggagcgtgaccggctaccgcctgtttgaggagattctgtaacgtacgacgc
gtggatccctcccccccccctaacgttactggccgaagccgcttggaataaggccggtgtgcgtttgtctatatgttattttccacca
tattgccgtcttttggcaatgtgagggcccggaaacctggccctgtcttcttgacgagcattcctaggggtctttcccctctcgccaa
aggaatgcaaggtctgttgaatgtcgtgaaggaagcagttcctctggaagcttcttgaagacaaacaacgtctgtagcgaccctttt
gcaggcagcggaacccccacctggcgacaggtgcctctgcggccaaaagccacgtgtataagatacacctgcaaaggcggc

acaaccccagtgccacgttgtgagttggatagttgtggaaagagtcaaatggctctcctcaagcgtattcaacaaggggctgaag
gatgcccagaaggtaccccattgtatgggatctgatctggggcctcggtgcacatgctttacatgtgtttagtcgaggttaaaaaa
cgtctaggccccccgaaccacggggacgtggttttcctttgaaaaacacgatgataatatggccacagcctgcaggatgggctcc
aaaaccatcgttctttcggtcggcgaggctactcgcactctgactgagatccagtccaccgcagaccgtcagatcttcgaagaga
aggtcgggcctctggtgggtcggctgcgcctcacggcttcgctccgtcaaaacggagccaagaccgcgtatcgcgtcaacctaa
aactggatcaggcggacgtcgttgattccggacttccgaaagtgcgctacactcaggtatggtcgcacgacgtgacaatcgttgc
gaatagcaccgaggcctcgcgcaaatcgttgtacgatttgaccaagtccctcgtcgcgacctcgcaggtcgaagatcttgtcgtca
accttgtgccgctgggccgtatcgatggcggaggcgggtctggcggcggcggcagcggtggaggaggttctttcacactcgaa
gatttcgttggggactgggaacagacagccgcctacaacctggaccaagtccttgaacagggaggtgtgtccagtttgctgcaga
atctcgccgtgtccgtaactccgatccaaaggattgtccggagcggtgaaaatgccctgaagatcgacatccatgtcatcatcccg
tatgaaggtctgagcgccgaccaaatggcccagatcgaagaggtgtttaaggtggtgtaccctgtggatgatcatcactttaaggt
gatcctgccctatggcacactggtaatcgacggggttacgccgaacatgctgaactatttcggacggccgtatgaaggcatcgcc
gtgttcgacggcaaaaagatcactgtaacagggaccctgtggaacggcaacaaaattatcgacgagcgcctgatcacccccga
cggctccatgctgttccgagtaaccatcaacagcctcgaggactacaaggacgacgatgacaag

NLS - HA - MCP - G4SX3 - SmBiT114 - IRES - PCP - G4SX5 - LgBiT – FLAG

cgttacataacttacggtaaatggcccgcctggctgaccgcccaacgacccccgcccattgacgtcaataatgacgtatgttccca
tagtaacgccaatagggactttccattgacgtcaatgggtggagtatttacggtaaactgcccacttggcagtacatcaagtgtatca
tatgccaagtacgccccctattgacgtcaatgacggtaaatggcccgcctggcattatgcccagtacatgaccttatgggactttcc
tacttggcagtacatctacgtattagtcatcgctattaccatggtgatgcggttttggcagtacatcaatgggcgtggatagcggtttg
actcacggggatttccaagtctccaccccattgacgtcaatgggagtttgttttggcaccaaaatcaacgggactttccaaaatgtc
gtaacaactccgccccattgacgcaaatgggcggtaggcgtgtacggtgggaggtctatataagcagagctctctggctaactag
agaacccactgcttactggcttatcgaaattaatacgactcactatagggagacccaagcttatgggcccaaaaaagaaaagaaa
agttggctacccctacgacgtgcccgactacgccatcgaaggccgccatatgctagccgttaaaatggcttctaactttactcagtt
cgttctcgtcgacaatggcggaactggcgacgtgactgtcgccccaagcaacttcgctaacgggatcgctgaatggatcagctct
aactcgcgttcacaggcttacaaagtaacctgtagcgttcgtcagagctctgcgcagaatcgcaaatacaccatcaaagtcgaggt
gcctaaaggcgcctggcgttcgtacttaaatatggaactaaccattccaattttcgccacgaattccgactgcgagcttattgttaag
gcaatgcaaggtctcctaaaagatggaaacccgattccctcagcaatcgcagcaaactccggcatctacgatatcggcggcgga
ggctctggaggcggcggaagcggtgggggtgggagcgtgaccggctaccgcctgtttgaggagattctgtaacgtacgacgc
gtggatccctcccccccccctaacgttactggccgaagccgcttggaataaggccggtgtgcgtttgtctatatgttattttccacca
tattgccgtcttttggcaatgtgagggcccggaaacctggccctgtcttcttgacgagcattcctaggggtctttcccctctcgccaa
aggaatgcaaggtctgttgaatgtcgtgaaggaagcagttcctctggaagcttcttgaagacaaacaacgtctgtagcgaccctttg
caggcagcggaaccccccacctggcgacaggtgcctctgcggccaaaagccacgtgtataagatacacctgcaaaggcggc
acaaccccagtgccacgttgtgagttggatagttgtggaaagagtcaaatggctctcctcaagcgtattcaacaaggggctgaag
gatgcccagaaggtaccccattgtatgggatctgatctggggcctcggtgcacatgctttacatgtgtttagtcgaggttaaaaaa
cgtctaggccccccgaaccacggggacgtggttttcctttgaaaaacacgatgataatatggccacagcctgcaggatgggctcc
aaaaccatcgttctttcggtcggcgaggctactcgcactctgactgagatccagtccaccgcagaccgtcagatcttcgaagaga
aggtcgggcctctggtgggtcggctgcgcctcacggcttcgctccgtcaaaacggagccaagaccgcgtatcgcgtcaacctaa
aactggatcaggcggacgtcgttgattccggacttccgaaagtgcgctacactcaggtatggtcgcacgacgtgacaatcgttgc
gaatagcaccgaggcctcgcgcaaatcgttgtacgatttgaccaagtccctcgtcgcgacctcgcaggtcgaagatcttgtcgtca
accttgtgccgctgggccgtatcgatggcggaggcgggtctggtggagggggatctggaggtggtgggtctggcggcggcgg
cagcggtggaggaggttctttcacactcgaagatttcgttggggactgggaacagacagccgcctacaacctggaccaagtcctt
gaacagggaggtgtgtccagtttgctgcagaatctcgccgtgtccgtaactccgatccaaaggattgtccggagcggtgaaaatg
ccctgaagatcgacatccatgtcatcatcccgtatgaaggtctgagcgccgaccaaatggcccagatcgaagaggtgtttaaggt
ggtgtaccctgtggatgatcatcactttaaggtgatcctgccctatggcacactggtaatcgacggggttacgccgaacatgctga
actatttcggacggccgtatgaaggcatcgccgtgttcgacggcaaaaagatcactgtaacagggaccctgtggaacggcaaca
aaattatcgacgagcgcctgatcacccccgacggctccatgctgttccgagtaaccatcaacagcctcgaggactacaaggacg
acgatgacaag

NLS - HA - MCP - G4SX5 - SmBiT114 - IRES - PCP - G4S – LgBiT – FLAG

cgttacataacttacggtaaatggcccgcctggctgaccgcccaacgacccccgcccattgacgtcaataatgacgtatgttccca
tagtaacgccaatagggactttccattgacgtcaatgggtggagtatttacggtaaactgcccacttggcagtacatcaagtgtatca
tatgccaagtacgccccctattgacgtcaatgacggtaaatggcccgcctggcattatgcccagtacatgaccttatgggactttcc
tacttggcagtacatctacgtattagtcatcgctattaccatggtgatgcggttttggcagtacatcaatgggcgtggatagcggtttg
actcacggggatttccaagtctccaccccattgacgtcaatgggagtttgttttggcaccaaaatcaacgggactttccaaaatgtc
gtaacaactccgccccattgacgcaaatgggcggtaggcgtgtacggtgggaggtctatataagcagagctctctggctaactag
agaacccactgcttactggcttatcgaaattaatacgactcactatagggagacccaagcttatgggcccaaaaaagaaaagaaa
agttggctacccctacgacgtgcccgactacgccatcgaaggccgccatatgctagccgttaaaatggcttctaactttactcagtt
cgttctcgtcgacaatggcggaactggcgacgtgactgtcgccccaagcaacttcgctaacgggatcgctgaatggatcagctct
aactcgcgttcacaggcttacaaagtaacctgtagcgttcgtcagagctctgcgcagaatcgcaaatacaccatcaaagtcgaggt
gcctaaaggcgcctggcgttcgtacttaaatatggaactaaccattccaattttcgccacgaattccgactgcgagcttattgttaag
gcaatgcaaggtctcctaaaagatggaaacccgattccctcagcaatcgcagcaaactccggcatctacgatatcggcggcgga
ggctctggtggaggaggttctggcggcggtggttccggaggcggcggaagcggtgggggtgggagcgtgaccggctaccgc
ctgtttgaggagattctgtaacgtacgacgcgtggatccctcccccccccctaacgttactggccgaagccgcttggaataaggcc
ggtgtgcgtttgtctatatgttattttccaccatattgccgtcttttggcaatgtgagggcccggaaacctggccctgtcttcttgacga
gcattcctaggggtctttcccctctcgccaaaggaatgcaaggtctgttgaatgtcgtgaaggaagcagttcctctggaagcttcttg
aagacaaacaacgtctgtagcgaccctttgcaggcagcggaaccccccacctggcgacaggtgcctctgcggccaaaagcca
cgtgtataagatacacctgcaaaggcggcacaaccccagtgccacgttgtgagttggatagttgtggaaagagtcaaatggctct
cctcaagcgtattcaacaaggggctgaaggatgcccagaaggtaccccattgtatgggatctgatctggggcctcggtgcacatg
ctttacatgtgtttagtcgaggttaaaaaaacgtctaggccccccgaaccacggggacgtggttttcctttgaaaaacacgatgata
atatggccacagcctgcaggatgggctccaaaaccatcgttctttcggtcggcgaggctactcgcactctgactgagatccagtcc
accgcagaccgtcagatcttcgaagagaaggtcgggcctctggtgggtcggctgcgcctcacggcttcgctccgtcaaaacgg
agccaagaccgcgtatcgcgtcaacctaaaactggatcaggcggacgtcgttgattccggacttccgaaagtgcgctacactca
ggtatggtcgcacgacgtgacaatcgttgcgaatagcaccgaggcctcgcgcaaatcgttgtacgatttgaccaagtccctcgtc
gcgacctcgcaggtcgaagatcttgtcgtcaaccttgtgccgctgggccgtatcgatggcggaggcgggtctttcacactcgaag
atttcgttggggactgggaacagacagccgcctacaacctggaccaagtccttgaacagggaggtgtgtccagtttgctgcagaa
tctcgccgtgtccgtaactccgatccaaaggattgtccggagcggtgaaaatgccctgaagatcgacatccatgtcatcatcccgt
atgaaggtctgagcgccgaccaaatggcccagatcgaagaggtgtttaaggtggtgtaccctgtggatgatcatcactttaaggtg
atcctgccctatggcacactggtaatcgacggggttacgccgaacatgctgaactatttcggacggccgtatgaaggcatcgccg
tgttcgacggcaaaaagatcactgtaacagggaccctgtggaacggcaacaaaattatcgacgagcgcctgatcacccccgac
ggctccatgctgttccgagtaaccatcaacagcctcgaggactacaaggacgacgatgacaag

NLS - HA – MCP - G4Sx5 - SmBiT114 - IRES - PCP - G4Sx3 - LgBiT – FLAG

cgttacataacttacggtaaatggcccgcctggctgaccgcccaacgacccccgcccattgacgtcaataatgacgtatgttccca
tagtaacgccaatagggactttccattgacgtcaatgggtggagtatttacggtaaactgcccacttggcagtacatcaagtgtatca
tatgccaagtacgccccctattgacgtcaatgacggtaaatggcccgcctggcattatgcccagtacatgaccttatgggactttcc
tacttggcagtacatctacgtattagtcatcgctattaccatggtgatgcggttttggcagtacatcaatgggcgtggatagcggtttg
actcacggggatttccaagtctccaccccattgacgtcaatgggagtttgttttggcaccaaaatcaacgggactttccaaaatgtc
gtaacaactccgccccattgacgcaaatgggcggtaggcgtgtacggtgggaggtctatataagcagagctctctggctaactag
agaacccactgcttactggcttatcgaaattaatacgactcactatagggagacccaagcttatgggcccaaaaaagaaaagaaa
agttggctacccctacgacgtgcccgactacgccatcgaaggccgccatatgctagccgttaaaatggcttctaactttactcagtt
cgttctcgtcgacaatggcggaactggcgacgtgactgtcgccccaagcaacttcgctaacgggatcgctgaatggatcagctct
aactcgcgttcacaggcttacaaagtaacctgtagcgttcgtcagagctctgcgcagaatcgcaaatacaccatcaaagtcgaggt
gcctaaaggcgcctggcgttcgtacttaaatatggaactaaccattccaattttcgccacgaattccgactgcgagcttattgttaag
gcaatgcaaggtctcctaaaagatggaaacccgattccctcagcaatcgcagcaaactccggcatctacgatatcggcggcgga
ggctctggtggaggaggttctggcggcggtggttccggaggcggcggaagcggtgggggtgggagcgtgaccggctaccgc
ctgtttgaggagattctgtaacgtacgacgcgtggatccctcccccccccctaacgttactggccgaagccgcttggaataaggcc

ggtgtgcgtttgtctatatgttattttccaccatattgccgtcttttggcaatgtgagggcccggaaacctggccctgtcttcttgacga
gcattcctaggggtctttcccctctcgccaaaggaatgcaaggtctgttgaatgtcgtgaaggaagcagttcctctggaagcttcttg
aagacaaacaacgtctgtagcgaccctttgcaggcagcggaaccccccacctggcgacaggtgcctctgcggccaaaagcca
cgtgtataagatacacctgcaaaggcggcacaaccccagtgccacgttgtgagttggatagttgtggaaagagtcaaatggctct
cctcaagcgtattcaacaaggggctgaaggatgcccagaaggtaccccattgtatgggatctgatctggggcctcggtgcacatg
ctttacatgtgtttagtcgaggttaaaaaaacgtctaggccccccgaaccacggggacgtggttttcctttgaaaaacacgatgata
atatggccacagcctgcaggatgggctccaaaaccatcgttctttcggtcggcgaggctactcgcactctgactgagatccagtcc
accgcagaccgtcagatcttcgaagagaaggtcgggcctctggtgggtcggctgcgcctcacggcttcgctccgtcaaaacgg
agccaagaccgcgtatcgcgtcaacctaaaactggatcaggcggacgtcgttgattccggacttccgaaagtgcgctacactca
ggtatggtcgcacgacgtgacaatcgttgcgaatagcaccgaggcctcgcgcaaatcgttgtacgatttgaccaagtccctcgtc
gcgacctcgcaggtcgaagatcttgtcgtcaaccttgtgccgctgggccgtatcgatggcggaggcgggtctggcggcggcgg
cagcggtggaggaggttctttcacactcgaagatttcgttggggactgggaacagacagccgcctacaacctggaccaagtcctt
gaacagggaggtgtgtccagtttgctgcagaatctcgccgtgtccgtaactccgatccaaaggattgtccggagcggtgaaaatg
ccctgaagatcgacatccatgtcatcatcccgtatgaaggtctgagcgccgaccaaatggcccagatcgaagaggtgtttaaggt
ggtgtaccctgtggatgatcatcactttaaggtgatcctgccctatggcacactggtaatcgacggggttacgccgaacatgctga
actatttcggacggccgtatgaaggcatcgccgtgttcgacggcaaaaagatcactgtaacagggaccctgtggaacggcaaca
aaattatcgacgagcgcctgatcacccccgacggctccatgctgttccgagtaaccatcaacagcctcgaggactacaaggacg
acgatgacaag

NLS - HA – MCP - G4Sx5 - SmBiT114 - IRES - PCP - G4Sx5 - LgBiT – FLAG

cgttacataacttacggtaaatggcccgcctggctgaccgcccaacgacccccgcccattgacgtcaataatgacgtatgttccca
tagtaacgccaatagggactttccattgacgtcaatgggtggagtatttacggtaaactgcccacttggcagtacatcaagtgtatca
tatgccaagtacgccccctattgacgtcaatgacggtaaatggcccgcctggcattatgcccagtacatgaccttatgggactttcc
tacttggcagtacatctacgtattagtcatcgctattaccatggtgatgcggttttggcagtacatcaatgggcgtggatagcggtttg
actcacggggatttccaagtctccaccccattgacgtcaatgggagtttgttttggcaccaaaatcaacgggactttccaaaatgtc
gtaacaactccgccccattgacgcaaatgggcggtaggcgtgtacggtgggaggtctatataagcagagctctctggctaactag
agaacccactgcttactggcttatcgaaattaatacgactcactatagggagacccaagcttatgggcccaaaaaagaaaagaaa
agttggctacccctacgacgtgcccgactacgccatcgaaggccgccatatgctagccgttaaaatggcttctaactttactcagtt
cgttctcgtcgacaatggcggaactggcgacgtgactgtcgccccaagcaacttcgctaacgggatcgctgaatggatcagctct
aactcgcgttcacaggcttacaaagtaacctgtagcgttcgtcagagctctgcgcagaatcgcaaatacaccatcaaagtcgaggt
gcctaaaggcgcctggcgttcgtacttaaatatggaactaaccattccaattttcgccacgaattccgactgcgagcttattgttaag
gcaatgcaaggtctcctaaaagatggaaacccgattccctcagcaatcgcagcaaactccggcatctacgatatcggcggcgga
ggctctggtggaggaggttctggcggcggtggttccggaggcggcggaagcggtgggggtgggagcgtgaccggctaccgc
ctgtttgaggagattctgtaacgtacgacgcgtggatccctcccccccccctaacgttactggccgaagccgcttggaataaggcc
ggtgtgcgtttgtctatatgttattttccaccatattgccgtcttttggcaatgtgagggcccggaaacctggccctgtcttcttgacga
gcattcctaggggtctttcccctctcgccaaaggaatgcaaggtctgttgaatgtcgtgaaggaagcagttcctctggaagcttcttg
aagacaaacaacgtctgtagcgaccctttgcaggcagcggaaccccccacctggcgacaggtgcctctgcggccaaaagcca
cgtgtataagatacacctgcaaaggcggcacaaccccagtgccacgttgtgagttggatagttgtggaaagagtcaaatggctct
cctcaagcgtattcaacaaggggctgaaggatgcccagaaggtaccccattgtatgggatctgatctggggcctcggtgcacatg
ctttacatgtgtttagtcgaggttaaaaaaacgtctaggccccccgaaccacggggacgtggttttcctttgaaaaacacgatgata
atatggccacagcctgcaggatgggctccaaaaccatcgttctttcggtcggcgaggctactcgcactctgactgagatccagtcc
accgcagaccgtcagatcttcgaagagaaggtcgggcctctggtgggtcggctgcgcctcacggcttcgctccgtcaaaacgg
agccaagaccgcgtatcgcgtcaacctaaaactggatcaggcggacgtcgttgattccggacttccgaaagtgcgctacactca
ggtatggtcgcacgacgtgacaatcgttgcgaatagcaccgaggcctcgcgcaaatcgttgtacgatttgaccaagtccctcgtc
gcgacctcgcaggtcgaagatcttgtcgtcaaccttgtgccgctgggccgtatcgatggcggaggcgggtctggtggaggggg
atctggaggtggtgggtctggcggcggcggcagcggtggaggaggttctttcacactcgaagatttcgttggggactgggaaca
gacagccgcctacaacctggaccaagtccttgaacagggaggtgtgtccagtttgctgcagaatctcgccgtgtccgtaactccg
atccaaaggattgtccggagcggtgaaaatgccctgaagatcgacatccatgtcatcatcccgtatgaaggtctgagcgccgacc

aaatggcccagatcgaagaggtgtttaaggtggtgtaccctgtggatgatcatcactttaaggtgatcctgccctatggcacactgg
taatcgacggggttacgccgaacatgctgaactatttcggacggccgtatgaaggcatcgccgtgttcgacggcaaaaagatcac
tgtaacagggaccctgtggaacggcaacaaaattatcgacgagcgcctgatcacccccgacggctccatgctgttccgagtaac
catcaacagcctcgaggactacaaggacgacgatgacaag

NLS - HA – MCP - linker - SmBi114 - IRES - PCP -linker - YeLgBiT – FLAG

cgttacataacttacggtaaatggcccgcctggctgaccgcccaacgacccccgcccattgacgtcaataatgacgtatgttccca
tagtaacgccaatagggactttccattgacgtcaatgggtggagtatttacggtaaactgcccacttggcagtacatcaagtgtatca
tatgccaagtacgccccctattgacgtcaatgacggtaaatggcccgcctggcattatgcccagtacatgaccttatgggactttcc
tacttggcagtacatctacgtattagtcatcgctattaccatggtgatgcggttttggcagtacatcaatgggcgtggatagcggtttg
actcacggggatttccaagtctccaccccattgacgtcaatgggagtttgttttggcaccaaaatcaacgggactttccaaaatgtc
gtaacaactccgccccattgacgcaaatgggcggtaggcgtgtacggtgggaggtctatataagcagagctctctggctaactag
agaacccactgcttactggcttatcgaaattaatacgactcactatagggagacccaagcttatgggcccaaaaagaaaagaaa
agttggctacccctacgacgtgcccgactacgccatcgaaggccgccatatgctagccgttaaaatggcttctaactttactcagtt
cgttctcgtcgacaatggcggaactggcgacgtgactgtcgccccaagcaacttcgctaacgggatcgctgaatggatcagctct
aactcgcgttcacaggcttacaaagtaacctgtagcgttcgtcagagctctgcgcagaatcgcaaatacaccatcaaagtcgaggt
gcctaaaggcgcctggcgttcgtacttaaatatggaactaaccattccaattttcgccacgaattccgactgcgagcttattgttaag
gcaatgcaaggtctcctaaaagatggaaacccgattccctcagcaatcgcagcaaactccggcatctacgcggattctagacgg
gatcccgccaccgtgaccggctaccgcctgtttgaggagattctgtaacgtacgacgcgtggatccctcccccccccctaacgtt
actggccgaagccgcttggaataaggccggtgtgcgtttgtctatatgttattttccaccatattgccgtcttttggcaatgtgagggc
ccggaaacctggccctgtcttcttgacgagcattcctaggggtctttcccctctcgccaaaggaatgcaaggtctgttgaatgtcgt
gaaggaagcagttcctctggaagcttcttgaagacaaacaacgtctgtagcgaccctttgcaggcagcggaaccccccacctgg
cgacaggtgcctctgcggccaaaagccacgtgtataagatacacctgcaaaggcggcacaaccccagtgccacgttgtgagttg
gatagttgtggaaagagtcaaatggctctcctcaagcgtattcaacaaggggctgaaggatgcccagaaggtaccccattgtatg
ggatctgatctggggcctcggtgcacatgctttacatgtgtttagtcgaggttaaaaaaacgtctaggccccccgaaccacgggga
cgtggttttcctttgaaaaacacgatgataatatggccacagcctgcaggatgggctccaaaaccatcgttctttcggtcggcgagg
ctactcgcactctgactgagatccagtccaccgcagaccgtcagatcttcgaagagaaggtcgggcctctggtgggtcggctgc
gcctcacggcttcgctccgtcaaaacggagccaagaccgcgtatcgcgtcaacctaaaactggatcaggcggacgtcgttgatt
ccggacttccgaaagtgcgctacactcaggtatggtcgcacgacgtgacaatcgttgcgaatagcaccgaggcctcgcgcaaat
cgttgtacgatttgaccaagtccctcgtcgcgacctcgcaggtcgaagatcttgtcgtcaaccttgtgccgctgggccgtgcggat
ccaccggtatgcatcgccaccgccgtgagcaagggcgaggagctgttcaccggggtggtgcccatcctggtcgagctggacg
gcgacgtaaacggccacaagttcagcgtgtccggcgagggcgagggcgatgccacctacggcaagctgaccctgaagctgat
ctgcaccaccggcaagctgcccgtgccctggcccaccctcgtgaccaccctgggctacggcctgcagtgcttcgcccgctacc
ccgaccacatgaagcagcacgacttcttcaagtccgccatgcccgaaggctacgtccaggagcgcaccatcttcttcaaggacg
acggcaactacaagacccgcgccgaggtgaagttcgagggcgacaccctggtgaaccgcatcgagctgaagggcatcgactt
caaggaggacggcaacatcctggggcacaagctggagtacaactacaacagccacaacgtctatatcaccgccgacaagcag
aagaacggcatcaaggccaacttcaagatccgccacaacatcgaggacggcggcgtgcagctcgccgaccactaccagcaga
acacccccatcggcgacggccccgtgctgctgcccgacaaccactacctgagctaccagtccgccctgagcaaagaccccaac
gagaagcgcgatcacatggtcctgctggagttcgtgaccgccgcgatgttcacactcgaagatttcgttggggactgggaacag
acagccgcctacaacctggaccaagtccttgaacagggaggtgtgtccagtttgctgcagaatctcgccgtgtccgtaactccgat
ccaaaggattgtccggagcggtgaaaatgccctgaagatcgacatccatgtcatcatcccgtatgaaggtctgagcgccgacca
aatggcccagatcgaagaggtgtttaaggtggtgtaccctgtggatgatcatcactttaaggtgatcctgccctatggcacactggt
aatcgacggggttacgccgaacatgctgaactatttcggacggccgtatgaaggcatcgccgtgttcgacggcaaaaagatcact
gtaacagggaccctgtggaacggcaacaaaattatcgacgagcgcctgatcacccccgacggctccatgctgttccgagtaacc
atcaacagcctcgaggactacaaggacgacgatgacaag

NLS - HA – MCP - linker - SmBiT114 - IRES - PCP - linker - LumiLgBiT – FLAG

cgttacataacttacggtaaatggcccgcctggctgaccgcccaacgacccccgcccattgacgtcaataatgacgtatgttccca
tagtaacgccaatagggactttccattgacgtcaatgggtggagtatttacggtaaactgcccacttggcagtacatcaagtgtatca

tatgccaagtacgccccctattgacgtcaatgacggtaaatggcccgcctggcattatgcccagtacatgaccttatgggactttcc
tacttggcagtacatctacgtattagtcatcgctattaccatggtgatgcggttttggcagtacatcaatgggcgtggatagcggtttg
actcacggggatttccaagtctccaccccattgacgtcaatgggagtttgttttggcaccaaaatcaacgggactttccaaaatgtc
gtaacaactccgccccattgacgcaaatgggcggtaggcgtgtacggtgggaggtctatataagcagagctctctggctaactag
agaacccactgcttactggcttatcgaaattaatacgactcactatagggagacccaagcttatgggcccaaaaaagaaaagaaa
agttggctacccctacgacgtgcccgactacgccatcgaaggccgccatatgctagccgttaaaatggcttctaactttactcagtt
cgttctcgtcgacaatggcggaactggcgacgtgactgtcgccccaagcaacttcgctaacgggatcgctgaatggatcagctct
aactcgcgttcacaggcttacaaagtaacctgtagcgttcgtcagagctctgcgcagaatcgcaaatacaccatcaaagtcgaggt
gcctaaaggcgcctggcgttcgtacttaaatatggaactaaccattccaattttcgccacgaattccgactgcgagcttattgttaag
gcaatgcaaggtctcctaaaagatggaaacccgattccctcagcaatcgcagcaaactccggcatctacgcggattctagacgg
gatcccgccaccgtgaccggctaccgcctgtttgaggagattctgtaacgtacgacgcgtggatccctccccccccctaacgtt
actggccgaagccgcttggaataaggccggtgtgcgtttgtctatatgttattttccaccatattgccgtcttttggcaatgtgagggc
ccggaaacctggccctgtcttcttgacgagcattcctaggggtctttcccctctcgccaaaggaatgcaaggtctgttgaatgtcgt
gaaggaagcagttcctctggaagcttcttgaagacaaacaacgtctgtagcgaccctttgcaggcagcggaaccccccacctgg
cgacaggtgcctctgcggccaaaagccacgtgtataagatacacctgcaaaggcggcacaaccccagtgccacgttgtgagttg
gatagttgtggaaagagtcaaatggctctcctcaagcgtattcaacaaggggctgaaggatgcccagaaggtaccccattgtatg
ggatctgatctggggcctcggtgcacatgctttacatgtgtttagtcgaggttaaaaaaacgtctaggccccccgaaccacgggga
cgtggttttcctttgaaaaacacgatgataatatggccacagcctgcaggatgggctccaaaaccatcgttctttcggtcggcgagg
ctactcgcactctgactgagatccagtccaccgcagaccgtcagatcttcgaagagaaggtcgggcctctggtgggtcggctgc
gcctcacggcttcgctccgtcaaaacggagccaagaccgcgtatcgcgtcaacctaaaactggatcaggcggacgtcgttgatt
ccggacttccgaaagtgcgctacactcaggtatggtcgcacgacgtgacaatcgttgcgaatagcaccgaggcctcgcgcaaat
cgttgtacgatttgaccaagtccctcgtcgcgacctcgcaggtcgaagatcttgtcgtcaaccttgtgccgctgggccgtgcggat
ccaccggtatgcatcgccaccgccgtgagcaagggcgaggcagtgatcaaggagttcatgcggttcaaggtgcacatggagg
gctccatgaacggccacgagttcgagatcgagggcgagggcgagggccgcccctacgagggcacccagaccgccaagctg
aaggtgaccaagggtggccccctgcccttctcctgggacatcctgtcccctcagttcatgtacggctccagggccttcatcaagca
ccccgccgacatccccgactactataagcagtccttccccgagggcttcaagtgggagcgcgtgatgaacttcgaggacggcg
gcgccgtgaccgtgacccaggacacctccctggaggacggcaccctgatctacaaggtgaagctccgcggcaccaacttccct
cctgacggccccgtaatgcagaagaagacaatgggctgggaagcgtccaccgagcggttgtaccccgaggacggcgtgctga
agggcgacattaagatggccctgcgcctgaaggacggcggccgctacctggcggacttcaagaccacctacaaggccaagaa
gcccgtgcagatgcccggcgcctacaacgtcgaccgcaagttggacatcacctcccacaacgaggactacaccgtggtggaac
agtacgaacgctccgagggccgccactccaccggaaagttcacactcgaagatttcgttggggactgggaacagacagccgcc
tacaacctggaccaagtccttgaacagggaggtgtgtccagtttgctgcagaatctcgccgtgtccgtaactccgatccaaaggat
tgtccggagcggtgaaaatgccctgaagatcgacatccatgtcatcatcccgtatgaaggtctgagcgccgaccaaatggccca
gatcgaagaggtgtttaaggtggtgtaccctgtggatgatcatcactttaaggtgatcctgccctatggcacactggtaatcgacgg
ggttacgccgaacatgctgaactatttcggacggccgtatgaaggcatcgccgtgttcgacggcaaaaagatcactgtaacagg
gaccctgtggaacggcaacaaaattatcgacgagcgcctgatcacccccgacggctccatgctgttccgagtaaccatcaacag
cctcgaggactacaaggacgacgatgacaag

NLS - HA - MCP - FlucN - IRES - PCP - FlucC – FLAG

cgttacataacttacggtaaatggcccgcctggctgaccgcccaacgacccccgcccattgacgtcaataatgacgtatgttccca
tagtaacgccaatagggactttccattgacgtcaatgggtggagtatttacggtaaactgcccacttggcagtacatcaagtgtatca
tatgccaagtacgccccctattgacgtcaatgacggtaaatggcccgcctggcattatgcccagtacatgaccttatgggactttcc
tacttggcagtacatctacgtattagtcatcgctattaccatggtgatgcggttttggcagtacatcaatgggcgtggatagcggtttg
actcacggggatttccaagtctccaccccattgacgtcaatgggagtttgttttggcaccaaaatcaacgggactttccaaaatgtc
gtaacaactccgccccattgacgcaaatgggcggtaggcgtgtacggtgggaggtctatataagcagagctcgtttagtgaaccg
tcagatcgcctggagacgccatccacgctgttttgacctccatagaagacaccgactctagagccaccatgggcccaaaaaaga
aaagaaaagttggctacccctacgacgtgcccgactacgccatcgaaggccgccatatgctagccgttaaaatggcttctaacttt
actcagttcgttctcgtcgacaatggcggaactggcgacgtgactgtcgccccaagcaacttcgctaacgggatcgctgaatgga

tcagctctaactcgcgttcacaggcttacaaagtaacctgtagcgttcgtcagagctctgcgcagaatcgcaaatacaccatcaaa
gtcgaggtgcctaaaggcgcctggcgttcgtacttaaatatggaactaaccattccaattttcgccacgaattccgactgcgagctt
attgttaaggcaatgcaaggtctcctaaaagatggaaacccgattccctcagcaatcgcagcaaactccggcatctacgcggattc
tagacgggatcccgccaccgaagacgccaaaaacataaagaaaggcccggcgccattctatccgctggaagatggaaccgct
ggagagcaactgcataaggctatgaagagatacgccctggttcctggaacaattgcttttacagatgcacatatcgaggtggacat
cacttacgctgagtacttcgaaatgtccgttcggttggcagaagctatgaaacgatatgggctgaatacaaatcacagaatcgtcgt
atgcagtgaaaactctcttcaattctttatgccggtgttgggcgcgttatttatcggagttgcagttgcgcccgcgaacgacatttata
atgaacgtgaattgctcaacagtatgggcatttcgcagcctaccgtggtgttcgtttccaaaaaggggttgcaaaaaattttgaacgt
gcaaaaaaagctcccaatcatccaaaaaattattatcatggattctaaaacggattaccagggatttcagtcgatgtacacgttcgtc
acatctcatctacctcccggttttaatgaatacgattttgtgccagagtccttcgatagggacaagacaattgcactgatcatgaactc
ctctggatctactggtctgcctaaaggtgtcgctctgcctcatagaactgcctgcgtgagattctcgcatgccagagatcctatttttg
gcaatcaaatcattccggatactgcgattttaagtgttgttccattccatcacggttttggaatgtttactacactcggatatttgatatgt
ggatttcgagtcgtcttaatgtatagatttgaagaagagctgtttctgaggagccttcaggattacaagattcaaagtgcgctgctggt
gccaaccctattctccttcttcgccaaaagcactctgattgacaaatacgatttatctaatttacacgaaattgcttctggtggcgctcc
cctctctaaggaagtcggggaagcggttgccaagaggttccatctgccaggtatcaggcaaggatatgggctcactgagactac
atcagctattctgattacacccgagggggatgataaaccgggcgcggtcggtaaagttgttccattttttgaagcgaaggttgtgga
tctggataccgggaaaacgctgggcgttaatcaaagaggcgaactgtgtgtgagaggtcctatgattatgtcctaacgtacgacg
cgtggatccctcccccccccctaacgttactggccgaagccgcttggaataaggccggtgtgcgtttgtctatatgttattttccacc
atattgccgtcttttggcaatgtgagggcccggaaacctggccctgtcttcttgacgagcattcctaggggtctttcccctctcgcca
aaggaatgcaaggtctgttgaatgtcgtgaaggaagcagttcctctggaagcttcttgaagacaaacaacgtctgtagcgaccctttt
gcaggcagcggaaccccccacctggcgacaggtgcctctgcggccaaaagccacgtgtataagatacacctgcaaaggcggc
acaaccccagtgccacgttgtgagttggatagttgtggaaagagtcaaatggctctcctcaagcgtattcaacaaggggctgaag
gatgcccagaaggtaccccattgtatgggatctgatctggggcctcggtgcacatgctttacatgtgtttagtcgaggttaaaaaa
cgtctaggccccccgaaccacggggacgtggttttcctttgaaaaacacgatgataatatggccacagcctgcaggatgggctcc
aaaaccatcgttctttcggtcggcgaggctactcgcactctgactgagatccagtccaccgcagaccgtcagatcttcgaagaga
aggtcgggcctctggtgggtcggctgcgcctcacggcttcgctccgtcaaaacggagccaagaccgcgtatcgcgtcaacctaa
aactggatcaggcggacgtcgttgattccggacttccgaaagtgcgctacactcaggtatggtcgcacgacgtgacaatcgttgc
gaatagcaccgaggcctcgcgcaaatcgttgtacgatttgaccaagtccctcgtcgcgacctcgcaggtcgaagatcttgtcgtca
accttgtgccgctgggccgtgcggatccaccggtatgcatcgccaccgccctatgattatgtccggttatgtaaacaatccggaa
gcgaccaacgccttgattgacaaggatggatggctacattctggagacatagcttactgggacgaagacgaacacttcttcatcgt
tgaccgcctgaagtctctgattaagtacaaaggctatcaggtggctcccgctgaattggaatccatcttgctccaacaccccaacat
cttcgacgcaggtgtcgcaggtcttcccgacgatgacgccggtgaacttcccgccgccgttgttgttttggagcacggaaagacg
atgacggaaaaagagatcgtggattacgtcgccagtcaagtaacaaccgcgaaaaagttgcgcggaggagttgtgtttgtggac
gaagtaccgaaaggtcttaccggaaaactcgacgcaagaaaaatcagagagatcctcataaaggccaagaagggcggaaaga
tcgccgtgctcgaggactacaaggacgacgatgacaag

BFP - MS2-3-PP7

cgttacataacttacggtaaatggcccgcctggctgaccgcccaacgacccccgcccattgacgtcaataatgacgtatgttccca
tagtaacgccaatagggactttccattgacgtcaatgggtggagtatttacggtaaactgcccacttggcagtacatcaagtgtatca
tatgccaagtacgccccctattgacgtcaatgacggtaaatggcccgcctggcattatgcccagtacatgaccttatgggactttcc
tacttggcagtacatctacgtattagtcatcgctattaccatggtgatgcggttttggcagtacatcaatgggcgtggatagcggtttg
actcacggggatttccaagtctccaccccattgacgtcaatgggagtttgttttggcaccaaaatcaacgggactttccaaaatgtc
gtaacaactccgccccattgacgcaaatgggcggtaggcgtgtacggtgggaggtctatataagcagagctctctggctaactag
agaacccactgcttactggcttatcgaaattaatacgactcactatagggagacccaagcttcgccaccatgagcgagctgattaa
ggagaacatgcacatgaagctgtacatggagggcaccgtggacaaccatcacttcaagtgcacatccgagggcgaaggcaag
ccctacgagggcacccagaccatgagaatcaaggtggtcgagggcggccctctccccttcgccttcgacatcctggctactagc
ttcctctacggcagcaagaccttcatcaaccacacccagggcatccccgacttcttcaagcagtccttccctgagggcttcacatg
ggagagagtcaccacatacgaagacgggggcgtgctgaccgctacccaggacaccagcctccaggacggctgcctcatctac

aacgtcaagatcagaggggtgaacttcacatccaacggccctgtgatgcagaagaaaacactcggctgggaggccttcaccga
gacgctgtaccccgctgacggcggcctggaaggcagaaacgacatggccctgaagctcgtgggcgggagccatctgatcgca
aacatcaagaccacatatagatccaagaaacccgctaagaacctcaagatgcctggcgtctactatgtggactacagactggaaa
gaatcaaggaggccaacaacgagacctacgtcgagcagcacgaggtggcagtggccagatactgcgacctccctagcaaact
ggggcacaagcttaattaagaattctgcagatatccatcacactggcggccgcgggagacgagcatcagccgtcgagagcaga
cgatatggcgtcgctcggcaattgaaaaaacgccctccc

BFP - MS2-3-PP7 (mut)

cgttacataacttacggtaaatggcccgcctggctgaccgcccaacgacccccgcccattgacgtcaataatgacgtatgttccca
tagtaacgccaatagggactttccattgacgtcaatgggtggagtatttacggtaaactgcccacttggcagtacatcaagtgtatca
tatgccaagtacgccccctattgacgtcaatgacggtaaatggcccgcctggcattatgcccagtacatgaccttatgggactttcc
tacttggcagtacatctacgtattagtcatcgctattaccatggtgatgcggttttggcagtacatcaatgggcgtggatagcggtttg
actcacggggatttccaagtctccaccccattgacgtcaatgggagtttgttttggcaccaaaatcaacgggactttccaaaatgtc
gtaacaactccgccccattgacgcaaatgggcggtaggcgtgtacggtgggaggtctatataagcagagctctctggctaactag
agaacccactgcttactggcttatcgaaattaatacgactcactatagggagacccaagcttcgccaccatgagcgagctgattaa
ggagaacatgcacatgaagctgtacatggagggcaccgtggacaaccatcacttcaagtgcacatccgagggcgaaggcaag
ccctacgagggcacccagaccatgagaatcaaggtggtcgagggcggccctctcccctttcgccttcgacatcctggctactagc
ttcctctacggcagcaagaccttcatcaaccacacccagggcatccccgacttcttcaagcagtccttccctgagggcttcacatg
ggagagagtcaccacatacgaagacgggggcgtgctgaccgctacccaggacaccagcctccaggacggctgcctcatctac
aacgtcaagatcagaggggtgaacttcacatccaacggccctgtgatgcagaagaaaacactcggctgggaggccttcaccga
gacgctgtaccccgctgacggcggcctggaaggcagaaacgacatggccctgaagctcgtgggcgggagccatctgatcgca
aacatcaagaccacatatagatccaagaaacccgctaagaacctcaagatgcctggcgtctactatgtggactacagactggaaa
gaatcaaggaggccaacaacgagacctacgtcgagcagcacgaggtggcagtggccagatactgcgacctccctagcaaact
ggggcacaagcttaattaagaattctgcagatatccatcacactggcggccgcgggagacgagcatcagccgtcgagagcgac
gttatggcgtcgctcggcaattgaaaaaacgccctccc

Staygold – M-3-P

cgttacataacttacggtaaatggcccgcctggctgaccgcccaacgacccccgcccattgacgtcaataatgacgtatgttccca
tagtaacgccaatagggactttccattgacgtcaatgggtggagtatttacggtaaactgcccacttggcagtacatcaagtgtatca
tatgccaagtacgccccctattgacgtcaatgacggtaaatggcccgcctggcattatgcccagtacatgaccttatgggactttcc
tacttggcagtacatctacgtattagtcatcgctattaccatggtgatgcggttttggcagtacatcaatgggcgtggatagcggtttg
actcacggggatttccaagtctccaccccattgacgtcaatgggagtttgttttggcaccaaaatcaacgggactttccaaaatgtc
gtaacaactccgccccattgacgcaaatgggcggtaggcgtgtacggtgggaggtctatataagcagagctctctggctaactag
agaacccactgcttactggcttatcgaaattaatacgactcactatagggagacccaagcttgcggccgccaccatgctgctgccc
gtcccccctgctgctgggcctgctgggcgccgccgcggatccgatggtgagcaagggcgaggagctgtttacaggcgtgaccc
ccttcaagttccagctgaagggcaccatcaacggcaagagcttcaccgtggaaggcgagggcgagggcaatagccacgagg
gcagccacaaaggcaagtacgtgtgcaccagcggcaaactgccaatgtcttgggccgccctgggaactagcttcggctatggc
atgaagtactacaccaagtaccccagcggcctgaagaactggttccacgaggtgatgcccgagggcttcacctacgacagaca
catccagtacaagggcgacggcagcatccacgccaagcaccagcacttcatgaagaacggcacctaccacaacatcgtggagt
tcaccggccaggacttcaaggagaacagccccgtgctgaccggcgacatgaacgtgagcctgcccaacgaggtgcagcacat
ccccagagatgacggcgtggagtgcccagtgaccctgctgtaccctctgctgagcgacaagagcaagtgcgtggaggcctacc
agaacaccatcatcaagcccctgcacaatcagccagcccccgatgtgccataccactggatcagaaagcagtacacccagagc
aaggacgacaccgaggagagagaccacatcatccagagcgagaccctggaggcccacctgccatggcacgagccttctgctt
ctgccgtgaaggatgaactgtaagaattctgcagatatccatcacactggcggccgcgggagacgagcatcagccgtcgagag
cagacgatatggcgtcgctcggcaattgaaaaaacgccctccc

Staygold

Cgttacataacttacggtaaatggcccgcctggctgaccgcccaacgacccccgcccattgacgtcaataatgacgtatgttccc
atagtaacgccaatagggactttccattgacgtcaatgggtggagtatttacggtaaactgcccacttggcagtacatcaagtgtatc
atatgccaagtacgccccctattgacgtcaatgacggtaaatggcccgcctggcattatgcccagtacatgaccttatgggactttc

ctacttggcagtacatctacgtattagtcatcgctattaccatggtgatgcggttttggcagtacatcaatgggcgtggatagcggttt
gactcacggggatttccaagtctccaccccattgacgtcaatgggagtttgttttggcaccaaaatcaacgggactttccaaaatgt
cgtaacaactccgccccattgacgcaaatgggcggtaggcgtgtacggtgggaggtctatataagcagagctctctggctaacta
gagaacccactgcttactggcttatcgaaattaatacgactcactatagggagacccaagcttatggcgtctactcccttcaagttcc
agctgaagggcaccatcaacggcaagagcttcaccgtggaaggcgagggcgagggcaatagccacgagggcagccacaaa
ggcaagtacgtgtgcaccagcggcaaactgccaatgtcttgggccgccctgggaactagcttcggctatggcatgaagtactac
accaagtaccccagcggcctgaagaactggttccacgaggtgatgcccgagggcttcacctacgacagacacatccagtacaa
gggcgacggcagcatccacgccaagcaccagcacttcatgaagaacggcacctaccacaacatcgtggagttcaccggccag
gacttcaaggagaacagccccgtgctgaccggcgacatgaacgtgagcctgcccaacgaggtgcagcacatccccagagatg
acggcgtggagtgcccagtgaccctgctgtaccctctgctgagcgacaagagcaagtgcgtggaggcctaccagaacaccatc
atcaagcccctgcacaatcagccagcccccgatgtgccataccactggatcagaaagcagtacacccagagcaaggacgacac
cgaggagagagaccacatcatccagagcgagaccctggaggcccacctg

CDK6-*MS2*-*3*-*PP7*-IRES-Staygold

cgttacataacttacggtaaatggcccgcctggctgaccgcccaacgacccccgcccattgacgtcaataatgacgtatgttccca
tagtaacgccaatagggactttccattgacgtcaatgggtggagtatttacggtaaactgcccacttggcagtacatcaagtgtatca
tatgccaagtacgccccctattgacgtcaatgacggtaaatggcccgcctggcattatgcccagtacatgaccttatgggactttcc
tacttggcagtacatctacgtattagtcatcgctattaccatggtgatgcggttttggcagtacatcaatgggcgtggatagcggtttg
actcacggggatttccaagtctccaccccattgacgtcaatgggagtttgttttggcaccaaaatcaacgggactttccaaaatgtc
gtaacaactccgccccattgacgcaaatgggcggtaggcgtgtacggtgggaggtctatataagcagagctctctggctaactag
agaacccactgcttactggcttatcgaaattaatacgactcactatagggagacccaagcttgctaccggtcgccaccatgggcat
ggagaaggacagcctgagtcgcgccgatcagcagtatgagtgcgtggcggagatcggcgaaggcgcctatgggaaggtgttc
aaggcccgcgacctgaagaacggcggccgcttcgtggctctgaagcgcgtgcgagtgcagaccagtgaggagggcatgccg
ctctccaccatccgcgaggtggcggtgctgaggcacctggagaccttcgagcaccccaacgtggtcaggttgtttgatgtgtgca
cagtgtcacggacggacagagaaaccaagcttacactagtgtttgagcatgttgatcaagacttgaccacttacttggataaagttc
cagagcccggcgtacccacagaaaccataaaggatatgatgtttcagcttctccgaggtctggactttcttcattctcacagagtag
tgcatcgtgatctgaaaccgcagaacattctggtgaccagcagtggacagataaagctggctgactttggccttgcccgcatctat
agttttcagatggcccttacctcggtggtcgtcacgctgtggtaccgagccccagaagtcctgctccagtccagctatgccacccc
tgtggacctctggagtgtcggttgcatctttgcagaaatgtttcgcagaaagcctctttttcgtggaagttcagacgtggatcaactag
gaaaaatcttggacatcattggactcccaggagaggaagactggcctagggacgtggcccttccccggcaggcttttcattccaa
atctgctcaacccatcgagaagtttgtgacagatattgacgaactaggcaaagacctacttctgaaatgcctgacgtttaatccagct
aaaaggatatccgcctacggcgccctgaatcacccgtacttccaagatctggagagatacaaggacaacctgaactctcacctgc
catccaaccagagcacctcggagctgaacacagcctgagaattctgcagatatccatcacactggcggccgcgggagacgagc
atcagccgtcggagagcagacgatatggcgtcgctcggcaattgaaaaaacgcccctccctcgagcatgcagctagcgggccc
tattctatagtgtcacctaaatgctagagctcgctgatcagcctcgatccctcccccccccctaacgttactggccgaagccgcttg
gaataaggccggtgtgcgtttgtctatatgttattttccaccatattgccgtcttttggcaatgtgagggcccggaaacctggccctgt
cttcttgacgagcattcctaggggtctttcccctctcgccaaaggaatgcaaggtctgttgaatgtcgtgaaggaagcagttcctctg
gaagcttcttgaagacaaacaacgtctgtagcgaccctttgcaggcagcggaaccccccacctggcgacaggtgcctctgcggc
caaaagccacgtgtataagatacacctgcaaaggcggcacaaccccagtgccacgttgtgagttggatagttgtggaaagagtc
aaatggctctcctcaagcgtattcaacaaggggctgaaggatgcccagaaggtaccccattgtatgggatctgatctggggcctc
ggtgcacatgctttacatgtgtttagtcgaggttaaaaaaacgtctaggccccccgaaccacggggacgtggttttcctttgaaaaa
cacgatgataatatggccacagcctgcaggatgggcatgctgctgcccgtcccccctgctgctgggcctgctgggcgccgccgc
ggatccgatggtgagcaagggcgaggagctgtttacaggcgtgaccccccttcaagttccagctgaagggcaccatcaacggca
agagcttcaccgtggaaggcgagggcgagggcaatagccacgagggcagccacaaaggcaagtacgtgtgcaccagcggc
aaactgccaatgtcttgggccgccctgggaactagcttcggctatggcatgaagtactacaccaagtaccccagcggcctgaag
aactggttccacgaggtgatgcccgagggcttcacctacgacagacacatccagtacaagggcgacggcagcatccacgccaa
gcaccagcacttcatgaagaacggcacctaccacaacatcgtggagttcaccggccaggacttcaaggagaacagccccgtgc
tgaccggcgacatgaacgtgagcctgcccaacgaggtgcagcacatccccagagatgacggcgtggagtgcccagtgaccct

gctgtaccctctgctgagcgacaagagcaagtgcgtggaggcctaccagaacaccatcatcaagcccctgcacaatcagccag
cccccgatgtgccataccactggatcagaaagcagtacacccagagcaaggacgacaccgaggagagagaccacatcatcca
gagcgagaccctggaggcccacctgccatggcacgagccttctgcttctgccgtgaaggatgaactgtaa

mCherry-β-actin 3’ UTR-*MS2*-*3*-PP7

cgttacataacttacggtaaatggcccgcctggctgaccgcccaacgacccccgcccattgacgtcaataatgacgtatgttccca
tagtaacgccaatagggactttccattgacgtcaatgggtggagtatttacggtaaactgcccacttggcagtacatcaagtgtatca
tatgccaagtacgccccctattgacgtcaatgacggtaaatggcccgcctggcattatgcccagtacatgaccttatgggactttcc
tacttggcagtacatctacgtattagtcatcgctattaccatggtgatgcggttttggcagtacatcaatgggcgtggatagcggtttg
actcacggggatttccaagtctccaccccattgacgtcaatgggagtttgttttggcaccaaaatcaacgggactttccaaaatgtc
gtaacaactccgccccattgacgcaaatgggcggtaggcgtgtacggtgggaggtctatataagcagagctctctggctaactag
agaacccactgcttactggcttatcgaaattaatacgactcactatagggagacccaagcttcgccaccatgatggtgagcaagg
gcgaggaggataacatggccatcatcaaggagttcatgcgcttcaaggtgcacatggagggctccgtgaacggccacgagttc
gagatcgagggcgagggcgagggccgcccctacgagggcacccagaccgccaagctgaaggtgaccaagggtggcccccct
gcccttcgcctgggacatcctgtcccctcagttcatgtacggctccaaggcctacgtgaagcaccccgccgacatccccgactac
ttgaagctgtccttccccgagggcttcaagtgggagcgcgtgatgaacttcgaggacggcggcgtggtgaccgtgacccagga
ctcctccctgcaggacggcgagttcatctacaaggtgaagctgcgcggcaccaacttcccctccgacggccccgtaatgcagaa
gaagaccatgggctgggaggcctcctccgagcggatgtaccccgaggacggcgccctgaagggcgagatcaagcagaggct
gaagctgaaggacggcggccactacgacgctgaggtcaagaccacctacagggccaagaagcccgtgcagctgcccggcg
cctacaacgtcaacatcaagttggacatcacctcccacaacgaggactacaccatcgtggaacagtacgaacgcgccgagggc
cgccactccaccggcggcatggacgagctgtacaagtaaggatcttagtcattccaaatatgagatgcgttgttacaggaagtccc
ttgccatcctaaaagccaccccacttctctctaaggagaatggcccagtcctctcccaagtccacacaggggaggtgatagcattg
ctttcgtgtaaattatgtaatgcaaaatttttttaatcttcgccttaatactttttttattttgttttattttgaatgatgagccttcgtgccccccc
ttcccccttttttgtcccccaacttgagatgtatgaaggcttttggtctccctgggagtgggtggaggcagccagggcttacctgtac
actgacttgagaccagttgattacgaattcggatccgggagacgagcatcagccgtcgagagcagacgatatggcgtcgctcgg
caattgaaaaaacgccctccc

TRE2-TagRFP657-MS2-5-PP7-3' UTR-UBC- loxp- rtTA-Advanced-loxp-IRES

tccctatcagtgatagagaaaagtgaaagtcgagtttaccactccctatcagtgatagagaaaagtgaaagtcgagtttaccactcc
ctatcagtgatagagaaaagtgaaagtcgagtttaccactccctatcagtgatagagaaaagtgaaagtcgagtttaccactcccta
tcagtgatagagaaaagtgaaagtcgagtttaccactccctatcagtgatagagaaaagtgaaagtcgagtttaccactccctatca
gtgatagagaaaagtgaaagtcgagctcggtacccgggtcgaggtaggcgtgtacggtgggaggcctatataagcagagctcg
tttagtgaaccgtcagatcgcctggagacgccatccacgctgttttgacctccatagaagacaccgggaccgatccagcctaccg
gtcgccaccatgagcgagctgatcaccgagaacatgcacatgaagctgtacatggagggcaccgtgaacaaccaccacttcaa
gtgcacatccgagggcgaaggcaagccctacgagggcacccagacccagagaatcaaggtggtcgagggcggccctctccc
cttcgccttcgacatcctggctaccagcttcatgtacggcagtcacaccttcatcaaccacacccagggcatccccgacttctgga
agcagtccttccctgagggcttcacatgggagagagtcaccacatacgaagacgggggcgtgctgaccgctacccaggacacc
agcctccaggacggctgcctcatctacaacgtcaagatcagaggggtgaacttcccatccaacggccctgtgatgcagaagaaa
acactcggctgggaggcccacaccgagatgctgtaccccgctgacggcggcctggaaggcagaaccgcgctggccctgaag
ctcgtgggcgggggccacctgatctgcaacttcaagaccacatacagatccaagaaacccgctaagaacctcaagatgcccgg
cgtctactatgtggactacagactggaaagaatcaaggaggccgacaaagagacctacgtcgagcagcacgaggtggctgtgg
ccagatactgcgacctccctagcaaactggggcacaagcttaattaataagaattctgcagatatccatcacactggcggccgcg
ggagacgagcatcagccgtcggagagcagacgatatggcgtcgctcggcaattgaaaaaacgcccctccctcgagcatgcatc
tagagggccctattctatagtgtcacctaaatgctagagctcgctgatcagcctcgatttcaattggaagacgcctgtcaaggacac
cgacgtggagtcgccccgcgggtatcagggccttttgacaggccaatcctatagtgagtcgtattaaattacgcgtaagatctggc
ctccgcgccgggttttggcgcctcccgcgggcgcccccctcctcacggcgagcgctgccacgtcagacgaagggcgcagcg
agcgtcctgatccttccgcccggacgctcaggacagcggcccgctgctcataagactcggccttagaaccccagtatcagcaga
aggacattttaggacgggacttgggtgactctagggcactggttttctttccagagagcggaacaggcgaggaaaagtagtccctt
ctcggcgattctgcggagggatctccgtggggcggtgaacgccgatgattatataaggacgcgccgggtgtggcacagctagtt

ccgtcgcagccgggatttgggtcgcggttcttgtttgtggatcgctgtgatcgtcacttggtgagtagcgggctgctgggctggcc
ggggctttcgtggccgccgggccgctcggtgggacggaagcgtgtggagagaccgccaagggctgtagtctgggtccgcga
gcaaggttgccctgaactgggggttgggggggagcgcagcaaaatggcggctgttcccgagtcttgaatggaagacgcttgtga
ggcgggctgtgaggtcgttgaaacaaggtgggggcatggtgggcggcaagaacccaaggtcttgaggccttcgctaatgcg
ggaaagctcttattcgggtgagatgggctggggcaccatctggggaccctgacgtgaagtttgtcactgactggagaactcggttt
gtcgtctgttgcgggggcggcagttatggcggtgccgttgggcagtgcacccgtacctttgggagcgcgcgccctcgtcgtgtc
gtgacgtcacccgttctgttggcttataatgcagggtggggccacctgccggtaggtgtgcggtaggcttttctccgtcgcaggac
gcagggttcgggcctagggtaggctctcctgaatcgacaggcgccggacctctggtgaggggagggataagtgaggcgtcag
tttctttggtcggttttatgtacctatcttcttaagtagctgaagctccggttttgaactatgcgctcggggttggcgagtgtgttttgtga
agttttttaggcaccttttgaaatgtaatcatttgggtcaatatgtaattttcagtgttagactagtaaattgtccgctaaattctggccgttt
ttggctttttgttagacggatccataacttcgtataatgtatgctatacgaagttatgccaccatgtctagactggacaagagcaaagt
cataaacggagctctggaattactcaatggtgtcggtatcgaaggcctgacgacaaggaaactcgctcaaaagctgggagttga
gcagcctaccctgtactggcacgtgaagaacaagcgggccctgctcgatgccctgccaatcgagatgctggacaggcatcatac
ccacttctgccccctggaaggcgagtcatggcaagactttctgcggaacaacgccaagtcataccgctgtgctctcctctcacatc
gcgacggggctaaagtgcatctcggcacccgcccaacagagaaacagtacgaaaccctggaaaatcagctcgcgttcctgtgt
cagcaaggcttctccctggagaacgcactgtacgctctgtccgccgtgggccactttacactgggctgcgtattggaggaacagg
agcatcaagtagcaaaagaggaaagagagacacctaccaccgattctatgcccccacttctgagacaagcaattgagctgttcga
ccggcagggagccgaacctgccttccttttcggcctggaactaatcatatgtggcctggagaaacagctaaagtgcgaaagcgg
cgggccgaccgacgcccttgacgattttgacttagacatgctcccagccgatgcccttgacgactttgaccttgatatgctgcctgc
tgacgctcttgacgattttgaccttgacatgctccccgggtaaataacttcgtatagcatacattatacgaagttatggcgcgccaatt
ccgcccctctccctcccccccccctaacgttactggccgaagccgcttggaataaggccggtgtgcgtttgtctatatgttattttcc
accatattgccgtcttttggcaatgtgagggcccggaaacctggccctgtcttcttgacgagcattcctaggggtctttcccctctcg
ccaaaggaatgcaaggtctgttgaatgtcgtgaaggaagcagttcctctggaagcttcttgaagacaaacaacgtctgtagcgac
cctttgcaggcagcggaaccccccacctggcgacaggtgcctctgcggccaaaagccacgtgtataagatacacctgcaaagg
cggcacaaccccagtgccacgttgtgagttggatagttgtggaaagagtcaaatggctctcctcaagcgtattcaacaaggggct
gaaggatgcccagaaggtaccccattgtatgggatctgatctggggcctcggtgcacatgctttacatgtgtttagtcgaggttaaa
aaaacgtctaggccccccgaaccacggggacgtggttttcctttgaaaaacacgataata

RNA bait DNA sequences (T7 promoter)

Flexible
gtaatacgactcactataggcacgagcatcagccgtgcctccaggtcgaatcttcaaacgagcagacgatatggcgtcgctcg
MS2
gtaatacgactcactataggcacgagcatcagccgtgcc
PP7
gtaatacgactcactataggagcagacgatatggcgtcgctcg
MS2-3-PP7^{Mut}-Ni
gtaatacgactcactatagggagacgagcatcagccgtcgagagcgacgttatggcgtcgctcggcaattgaaaaaacgccctc
cc
MS2-0-PP7-Ni
gtaatacgactcactatagggacacgagcatcagccgtggagcagacgatatggcgtcgctcgcgaattgaaaaaaccgctccc
MS2-1-PP7-Ni
gtaatacgactcactatagggacgcgagcatcagccgcgagagcagacgatatggcgtcgctcgcgaattgaaaaaaccgctc
cc

MS2-3-PP7-Ni
gtaatacgactcactatagggagacgagcatcagccgtcgagagcagacgatatggcgtcgctcggcaattgaaaaaacgccct ccc
MS2-5-PP7-Ni
gtaatacgactcactatagggagacgagcatcagccgtcggagagcagacgatatggcgtcgctcggcaattgaaaaaacgcc cctccc
MS2-7-PP7-Ni
gtaatacgactcactatagggagacgagcatcagccgtccggagagcagacgatatggcgtcgctcggcaattgaaaaaacgc cccgtccc
MS2-9-PP7-Ni
gtaatacgactcactatagggagacgagcatcagccgtccgagagagcagacgatatggcgtcgctcggcaattgaaaaaacg ccctcgtccc
MS2-11-PP7-Ni
gtaatacgactcactatagggagacgagcatcagccgtccgacgagagcagacgatatggcgtcgctcggcaattgaaaaaac gcccgtcgtccc
MS2-13-Ni-PP7
gtaatacgactcactatagggagacgagcatcagccgtcctgagcggcaattgaaaaaacgccgagcagacgatatggcgtcg ctcgctcagtccc
MS2-21-PP7-Ni
gtaatacgactcactatagggagacgagcatcagccgtctcgacgagcaagagcagacgatatggcgtcgctcggcaattgaa aaaacgcctgctcgtcgatccc
7-MS2-7-PP7-Ni
gtaatacgactcactatagggacgacgagcatcagccgtcgcggagagcagacgatatggcgtcgctcggcaattgaaaaaac gccccgtccc
8-MS2-7-PP7-Ni
gtaatacgactcactatagggacgactgagcatcagccagtcgcggagagcagacgatatggcgtcgctcggcaattgaaaaa acgccccgtccc
7-MS2-3-PP7-Ni
gtaatacgactcactatagggacgacgagcatcagccgtcggagagcagacgatatggcgtcgctcggcaattgaaaaaacgc cctccc
8-MS2-3-PP7-Ni
gtaatacgactcactatagggacgactgagcatcagccagtcggagagcagacgatatggcgtcgctcggcaattgaaaaaac gccctccc
6-MS2-3-6-PP7-Ni
gtaatacgactcactatagggagacgagcatcagccgtcgagcagacgatatggcgtcgcggcaattgaaaaaacgccctccc
6-MS2-3-9-PP7-Ni
gtaatacgactcactatagggagacgagcatcagccgtcgagagcgagacgatatggcgtccgctcggcaattgaaaaaacgc cctccc
6-MS2-3-10-PP7-Ni
gtaatacgactcactatagggagacgagcatcagccgtcgagagcgcagacgatatggcgtcgcgctcggcaattgaaaaaac gccctccc
PP7-9-MS2-Ni
gtaatacgactcactatagggagagcagacgatatggcgtcgctcccagacacgagcatcagccgtgggcaattgaaaaaacg ccctggtccc

RT-qPCR Primer sequences

M-3-P
Forward ATGCTCGAGGGAGGGCGTTTTTC

StayGold
Forward GAGGAGAGAGACCACATCATCCAGAG
Reverse TTACAGTTCATCCTTCACGGCAGAAGC
