# Supplementary material for: A modular platform for bioluminescent RNA tracking
Source: Nat Commun. 2024 Nov 18;15:9992. doi: 10.1038/s41467-024-54263-5 (PMC11574019; doi:10.1038/s41467-024-54263-5)
Supplement: Supplementary file 2 — Description of Additional Supplementary Files [file 41467_2024_54263_MOESM2_ESM.pdf]

DESCRIPTION OF ADDITIONAL SUPPLEMENTARY FILES

Supplementary Data 1. Sequences of oligonucleotides used for synthesizing M-X-P and plasmid constructs. All constructs were confirmed by gel or sequencing.

Supplementary Movies 1-2. Representative dynamic imaging of β-actin under cellular stress. HEK293T cells expressing RNA lanterns were transfected with mCherry-β-actin–M-3-P (with M-3-P in the 3' UTR). Cells were treated with sodium arsenite and imaged as in Fig. S11. Luminescence images were acquired continuously for 40-60 min, using 90 s acquisitions. Samples movies are shown. Scale bars = 20 µm.

Supplementary Movies 3-5. Representative dynamic imaging of CDK6 under cellular stress. HEK293T cells expressing RNA lanterns were transfected with CDK6–M-3-P–IRES-GFP. Cells were treated with sodium arsenite and imaged as in Fig. S13. Luminescence images were acquired continuously for 60 min, using 90 s acquisitions. Samples movies are shown. Scale bars = 20 µm.
