# Supplementary material for: A modular platform for bioluminescent RNA tracking
Source: Nat Commun. 2024 Nov 18;15:9992. doi: 10.1038/s41467-024-54263-5 (PMC11574019; doi:10.1038/s41467-024-54263-5)
Supplement: Supplementary file 9 — Reporting Summary [file 41467_2024_54263_MOESM9_ESM.pdf]

nature portfolio

Corresponding author(s): Steward, Luptak, Prescher

Last updated by author(s): Sep 26, 2024

Reporting Summary

Nature Portfolio wishes to improve the reproducibility of the work that we publish. This form provides structure for consistency and transparency in reporting. For further information on Nature Portfolio policies, see our Editorial Policies and the Editorial Policy Checklist.

Statistics

For all statistical analyses, confirm that the following items are present in the figure legend, table legend, main text, or Methods section.

n/a	Confirmed	
☐	☒	The exact sample size (*n*) for each experimental group/condition, given as a discrete number and unit of measurement
☐	☒	A statement on whether measurements were taken from distinct samples or whether the same sample was measured repeatedly
☐	☒	The statistical test(s) used AND whether they are one- or two-sided<br>*Only common tests should be described solely by name; describe more complex techniques in the Methods section.*
☒	☐	A description of all covariates tested
☐	☒	A description of any assumptions or corrections, such as tests of normality and adjustment for multiple comparisons
☐	☒	A full description of the statistical parameters including central tendency (e.g. means) or other basic estimates (e.g. regression coefficient) AND variation (e.g. standard deviation) or associated estimates of uncertainty (e.g. confidence intervals)
☐	☒	For null hypothesis testing, the test statistic (e.g. *F*, *t*, *r*) with confidence intervals, effect sizes, degrees of freedom and *P* value noted<br>*Give P values as exact values whenever suitable.*
☒	☐	For Bayesian analysis, information on the choice of priors and Markov chain Monte Carlo settings
☒	☐	For hierarchical and complex designs, identification of the appropriate level for tests and full reporting of outcomes
☐	☒	Estimates of effect sizes (e.g. Cohen's *d*, Pearson's *r*), indicating how they were calculated

Our web collection on statistics for biologists contains articles on many of the points above.

Software and code

Policy information about availability of computer code

Data collection

In vitro and biochemical studies were collected with an Andor iXon Ultra 888 EMCCD camera (Oxford Instruments) equipped with an F 0.95 lens (Schneider) with an EM gain of 100 or 500 or with an IVIS Lumina II (Xenogen) CCD camera chilled to –90 °C. The stage was kept at 37 °C during the imaging session, and the camera was controlled using Living Image software.

For bioluminescence microscopy live cell images were captured on an Olympus IX71 microscope equipped with an Andor iXon Ultra 888 EMCCD camera and a 40X oil objective (Olympus UPlanApo 40X/1.00 oil iris). Images were captured with an EM gain of 1000, 10 MHz horizontal read-out rate, 4.33 µs vertical clock speed, and an acquisition time of 180 s. The microscope stage was kept warm with a heating pad to maintain cell viability and to encourage enzyme turnover. Fluorescence images were captured immediately following luminescence imaging, using a blue LED light source (ThorLabs, Solis-470C). Fluorescent images were captured using the EMCCD's conventional mode with an acquisition time of 0. 5s

In addition, for bioluminescence microscopy live cells images were captured on an Olympus IX71 microscope equipped with an HNu 512 EMCCD camera and a 40X objective (Olympus UPlanApo 40X/1.00 oil iris), 20X objective (Olympus LUCPlanFL 20X Ph1), 10X objective (Olympus UPlanFL N 10x/0.30 Ph1) and 4X objective (Olympus PlanC N 4x/0.10na). Images were captured with an EM gain of 1000, 10 MHz horizontal read-out rate, 7.55 fps, and an acquisition time of 90 s. Fluorescence images were captured either immediately before or following luminescence imaging, using a blue or green light source (Lycco Flashlight). Fluorescence emission was filtered using GFP (Olympus UM52), cy3 (Chroma UN41001 FITC CIN43662) and Texas Red (Olympus UM52) filters. Fluorescent images were captured using the EMCCD's conventional mode with an acquisition time of 1,2 and 4 seconds respectively.

	For imaging of in vivo cell transplants, images were captured on an Andor iXon Ultra 888 EMCCD camera equipped with an F 0.95 lens. Images were acquired with an EM gain of 1000, an acquisition time of 300 s, and signal was captured as photons.
Data analysis	Data from in vitro and biochemical studies from the Andor were analyzed with ImageJ (Fiji3) where images were Z-stacked in and densitometry was used to measure peak intensity values or were used to determine fold change over background and no RNA controls. Data collected from the IVIS were analyzed by selecting regions of interests for quantification and total flux values were analyzed using Living Image software. All data were exported to Microsoft Excel or Prism 10 (GraphPad) for further analyses.<br><br>Data from bioluminescence microscopy was processed with ImageJ (Fiji3) and colocalization was determined with the JaCoP plug-in 35.<br><br>Data from in vivo cell transplants were processed using ImageJ (Fiji3) . Total flux (p/s) was determined based on a given region of interest (ROI) for each implantation. Background correction was accomplished by averaging ROIs containing no luminescence. Prism10 (GraphPad) was used to determine significant differences (unpaired, two-tailed t-test) between groups.

For manuscripts utilizing custom algorithms or software that are central to the research but not yet described in published literature, software must be made available to editors and reviewers. We strongly encourage code deposition in a community repository (e.g. GitHub). See the Nature Portfolio guidelines for submitting code & software for further information.

Data

Policy information about availability of data

All manuscripts must include a data availability statement. This statement should provide the following information, where applicable:
- Accession codes, unique identifiers, or web links for publicly available datasets
- A description of any restrictions on data availability
- For clinical datasets or third party data, please ensure that the statement adheres to our policy

The data that support the findings of this study are available from the corresponding authors upon reasonable request.

Research involving human participants, their data, or biological material

Policy information about studies with human participants or human data. See also policy information about sex, gender (identity/presentation), and sexual orientation and race, ethnicity and racism.

Reporting on sex and gender	*Use the terms sex (biological attribute) and gender (shaped by social and cultural circumstances) carefully in order to avoid confusing both terms. Indicate if findings apply to only one sex or gender; describe whether sex and gender were considered in study design; whether sex and/or gender was determined based on self-reporting or assigned and methods used.*<br>*Provide in the source data disaggregated sex and gender data, where this information has been collected, and if consent has been obtained for sharing of individual-level data; provide overall numbers in this Reporting Summary. Please state if this information has not been collected.*<br>*Report sex- and gender-based analyses where performed, justify reasons for lack of sex- and gender-based analysis.*
Reporting on race, ethnicity, or other socially relevant groupings	*Please specify the socially constructed or socially relevant categorization variable(s) used in your manuscript and explain why they were used. Please note that such variables should not be used as proxies for other socially constructed/relevant variables (for example, race or ethnicity should not be used as a proxy for socioeconomic status).*<br>*Provide clear definitions of the relevant terms used, how they were provided (by the participants/respondents, the researchers, or third parties), and the method(s) used to classify people into the different categories (e.g. self-report, census or administrative data, social media data, etc.)*<br>*Please provide details about how you controlled for confounding variables in your analyses.*
Population characteristics	*Describe the covariate-relevant population characteristics of the human research participants (e.g. age, genotypic information, past and current diagnosis and treatment categories). If you filled out the behavioural & social sciences study design questions and have nothing to add here, write "See above."*
Recruitment	*Describe how participants were recruited. Outline any potential self-selection bias or other biases that may be present and how these are likely to impact results.*
Ethics oversight	*Identify the organization(s) that approved the study protocol.*

Note that full information on the approval of the study protocol must also be provided in the manuscript.

Field-specific reporting

Please select the one below that is the best fit for your research. If you are not sure, read the appropriate sections before making your selection.

[x] Life sciences [ ] Behavioural & social sciences [ ] Ecological, evolutionary & environmental sciences

For a reference copy of the document with all sections, see nature.com/documents/nr-reporting-summary-flat.pdf

Life sciences study design

All studies must disclose on these points even when the disclosure is negative.

Sample size	No statistical method was used to predetermine sample size. Sample size was chosen to allow for reliable statistical analysis. For in vitro studies sample size was determined by having at least three biological replicates performed by each first author, resulting in n=9 or more replicates. For static in cellulo studies replicates were determined by imaging the bioluminescence platform at least in triplicate and for dynamic in cellulo studies replicates were determined by imaged the RNA tag overtime at least in duplicate. In vivo studies replicates were determined by having at least an n=2 mice.
Data exclusions	No data were excluded from the analysis.
Replication	In vitro determination of biochemical validation of RNA mediated complementation of the RNA lanterns were determined in minimum duplicates, and in different experiments. In cellulo and in vivo experiments were imaged to validate RNA reconstitution of the RNA lanterns at least in minimum duplicates. All replicates were successful.
Randomization	No randomization was applied, due to the nature of the experiments.
Blinding	No blinding of the experimental set ups or analysis were applied, due to the nature of the experiments.

Reporting for specific materials, systems and methods

We require information from authors about some types of materials, experimental systems and methods used in many studies. Here, indicate whether each material, system or method listed is relevant to your study. If you are not sure if a list item applies to your research, read the appropriate section before selecting a response.

Materials & experimental systems

n/a	Involved in the study	
☐	☒	Antibodies
☐	☒	Eukaryotic cell lines
☒	☐	Palaeontology and archaeology
☐	☒	Animals and other organisms
☒	☐	Clinical data
☒	☐	Dual use research of concern
☒	☐	Plants

Methods

n/a	Involved in the study	
☒	☐	ChIP-seq
☐	☒	Flow cytometry
☒	☐	MRI-based neuroimaging

Antibodies

Antibodies used

Part of commercially available beads used for magnetic pull downs:
Pierce™ Anti-HA Magnetic Beads
Vendor: ThermoFisher
Catalog #: 88836
Lot #: YA353979
Binding Capacity : 0.1 ug/mL

Pierce™ Anti-DYKDDDDK Magnetic Agarose
Vendor: ThermoFisher
Catalog #: A36797
Lot #: YC367978
Binding Capacity : 3.2 ug/uL

Stress granule formation validation:
G3BP1 (E9G1M) XP® Rabbit mAb
Vendor: Cell Signaling
Catalog #: 61559T
Lot #: 3
Dilution: 1:100

Goat anti-Rabbit IgG (H+L) Cross-Adsorbed Secondary Antibody, Alexa Fluor™ 594
Vendor: Invitrogen
Catalog#: A11012
RRID: AB_2534079
Lot#: 2812001
Dilution: 2 µg/mL

Validation	Certificate of analysis provided by vendor/ validated in Supplementary Figure 12

Eukaryotic cell lines

Policy information about cell lines and Sex and Gender in Research

Cell line source(s)	HEK-293T provided by ATCC.
Authentication	Cell lines were not further authenticated.
Mycoplasma contamination	Cell lines have been tested and are negative.
Commonly misidentified lines (See ICLAC register)	No commonly misidentified cell lines were used.

Animals and other research organisms

Policy information about studies involving animals; ARRIVE guidelines recommended for reporting animal research, and Sex and Gender in Research

Laboratory animals	Rosa26tdTomato male mice obtained from the Jackson Laboratory (JAX: 007905).
Wild animals	The study did not involve wild animals.
Reporting on sex	Findings apply to only male mice. Sex was not considered in the experimental design.
Field-collected samples	This study did not involve samples collected from the field.
Ethics oversight	Mouse experiments were approved by the UC Irvine Animal Care and Use Committee (IACUC) in compliance with the National Institute of Health guidelines.

Note that full information on the approval of the study protocol must also be provided in the manuscript.

Plants

Seed stocks	*Report on the source of all seed stocks or other plant material used. If applicable, state the seed stock centre and catalogue number. If plant specimens were collected from the field, describe the collection location, date and sampling procedures.*
Novel plant genotypes	*Describe the methods by which all novel plant genotypes were produced. This includes those generated by transgenic approaches, gene editing, chemical/radiation-based mutagenesis and hybridization. For transgenic lines, describe the transformation method, the number of independent lines analyzed and the generation upon which experiments were performed. For gene-edited lines, describe the editor used, the endogenous sequence targeted for editing, the targeting guide RNA sequence (if applicable) and how the editor was applied.*
Authentication	*Describe any authentication procedures for each seed stock used or novel genotype generated. Describe any experiments used to assess the effect of a mutation and, where applicable, how potential secondary effects (e.g. second site T-DNA insertions, mosiacism, off-target gene editing) were examined.*

Flow Cytometry

Plots

Confirm that:

- [x] The axis labels state the marker and fluorochrome used (e.g. CD4-FITC).
- [x] The axis scales are clearly visible. Include numbers along axes only for bottom left plot of group (a 'group' is an analysis of identical markers).
- [x] All plots are contour plots with outliers or pseudocolor plots.
- [x] A numerical value for number of cells or percentage (with statistics) is provided.

Methodology

Sample preparation	Cells were incubated at 37 °C in a 5% CO2 humidified chamber. Cells were serially passaged using trypsin (0.25% in HBSS, Gibco). Cells were centrifuged for 500 x g for 5 min and washed 2x with PBS and then resuspended in 200 µL FACS buffer before transfer to a 96-well plate for flow cytometry analysis.
Instrument	Expression of all transient cell lines were checked via flow cytometry using an ACEA NovoCyte flow cytometer and the appropriate filter settings.

Software	Fluorescence was analyzed and quantified using the NovoExpress software (ACEA).
Cell population abundance	*Describe the abundance of the relevant cell populations within post-sort fractions, providing details on the purity of the samples and how it was determined.*
Gating strategy	Live cells and single cell populations were gated based on HEK control cells using FSC/SSC settings, and singlet cells were further gated. For each sample, 10,000 events were collected on the "singlet cell" gate.

☒ Tick this box to confirm that a figure exemplifying the gating strategy is provided in the Supplementary Information.
