# Supplementary figures and images for: A modular platform for bioluminescent RNA tracking (part 2 of 2)
Source: Nat Commun. 2024 Nov 18;15:9992. doi: 10.1038/s41467-024-54263-5 (PMC11574019; doi:10.1038/s41467-024-54263-5)

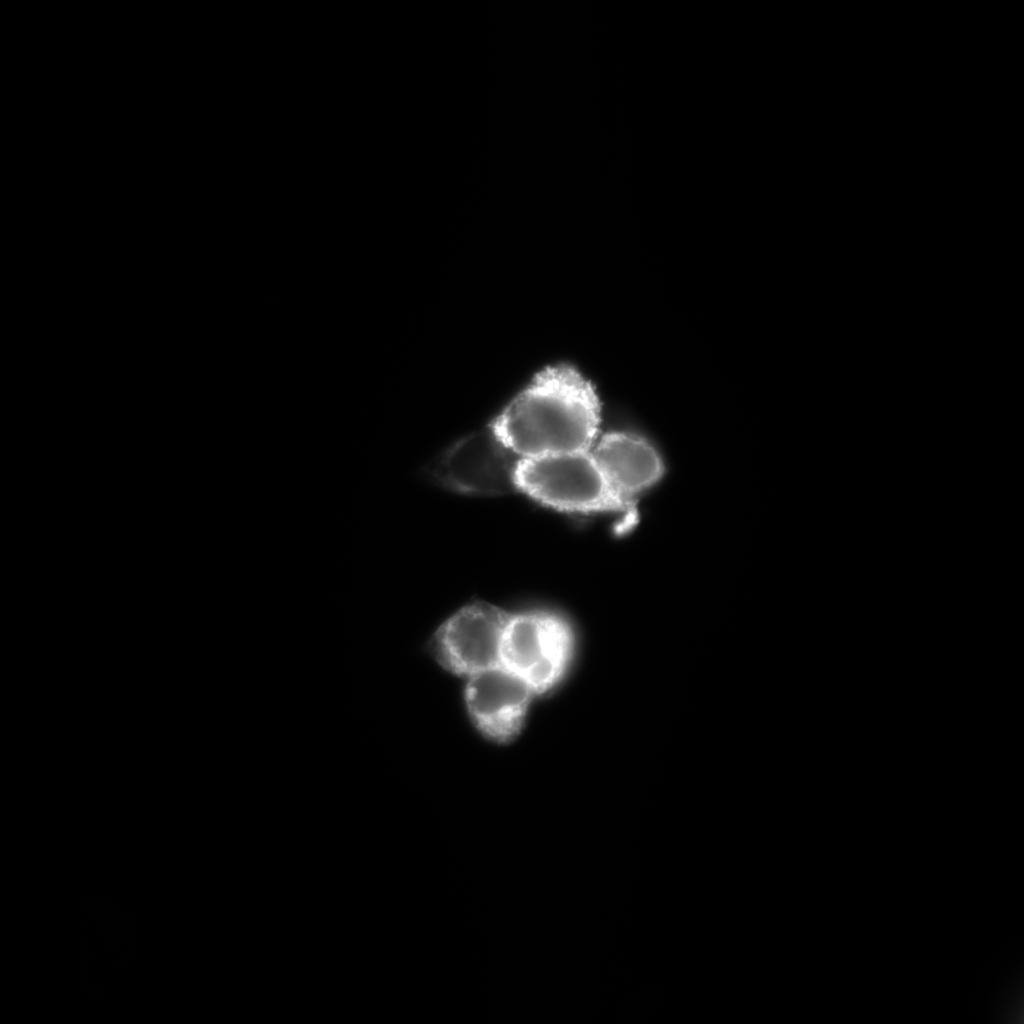

Supplement: Supplementary file 11 — Source Data [file 41467_2024_54263_MOESM11_ESM.zip › Source Data/Fig. 5/Fig. 5 B/Stable probe HEK + 300 probe + 100staygoldcontrol/fluorescence/StayGoldcontrol100ng_17_X1.tif]

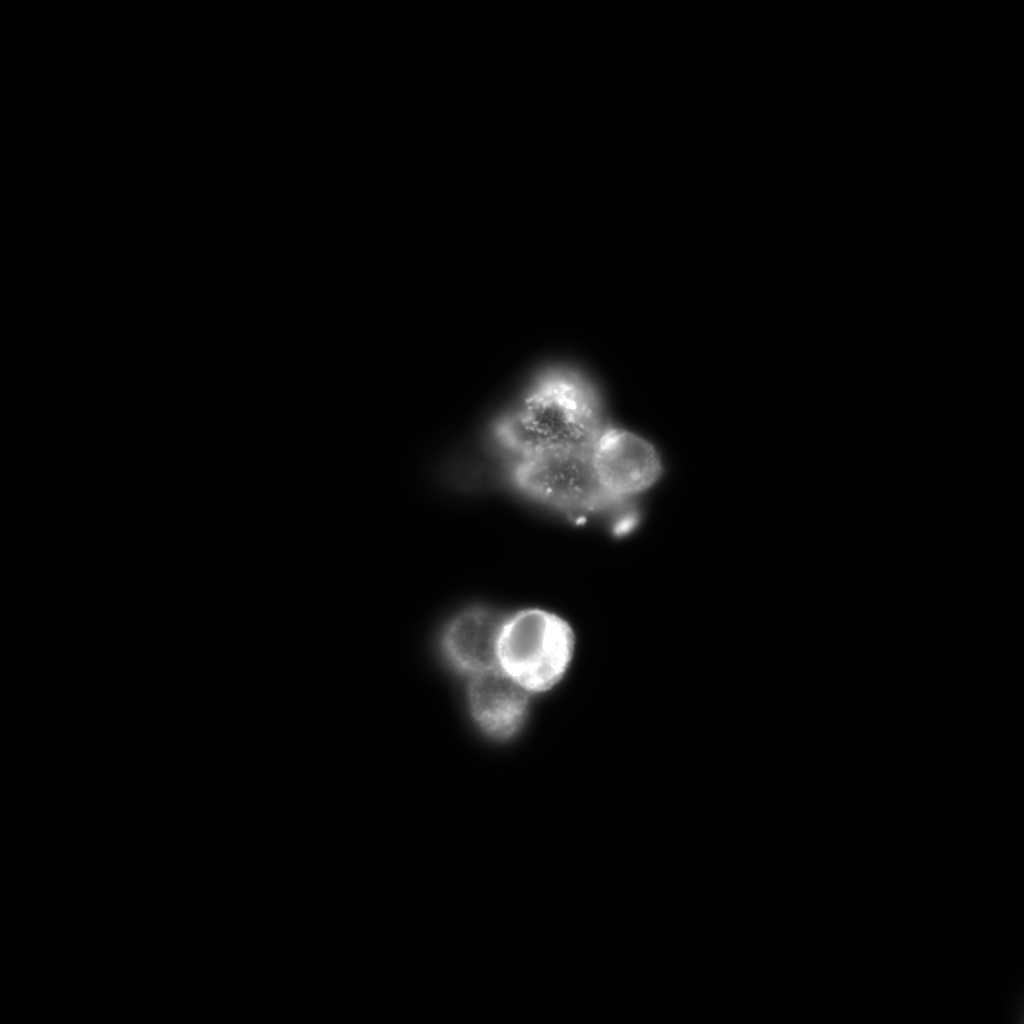

Supplement: Supplementary file 11 — Source Data [file 41467_2024_54263_MOESM11_ESM.zip › Source Data/Fig. 5/Fig. 5 B/Stable probe HEK + 300 probe + 100staygoldcontrol/fluorescence/StayGoldcontrol100ng_21_X1.tif]

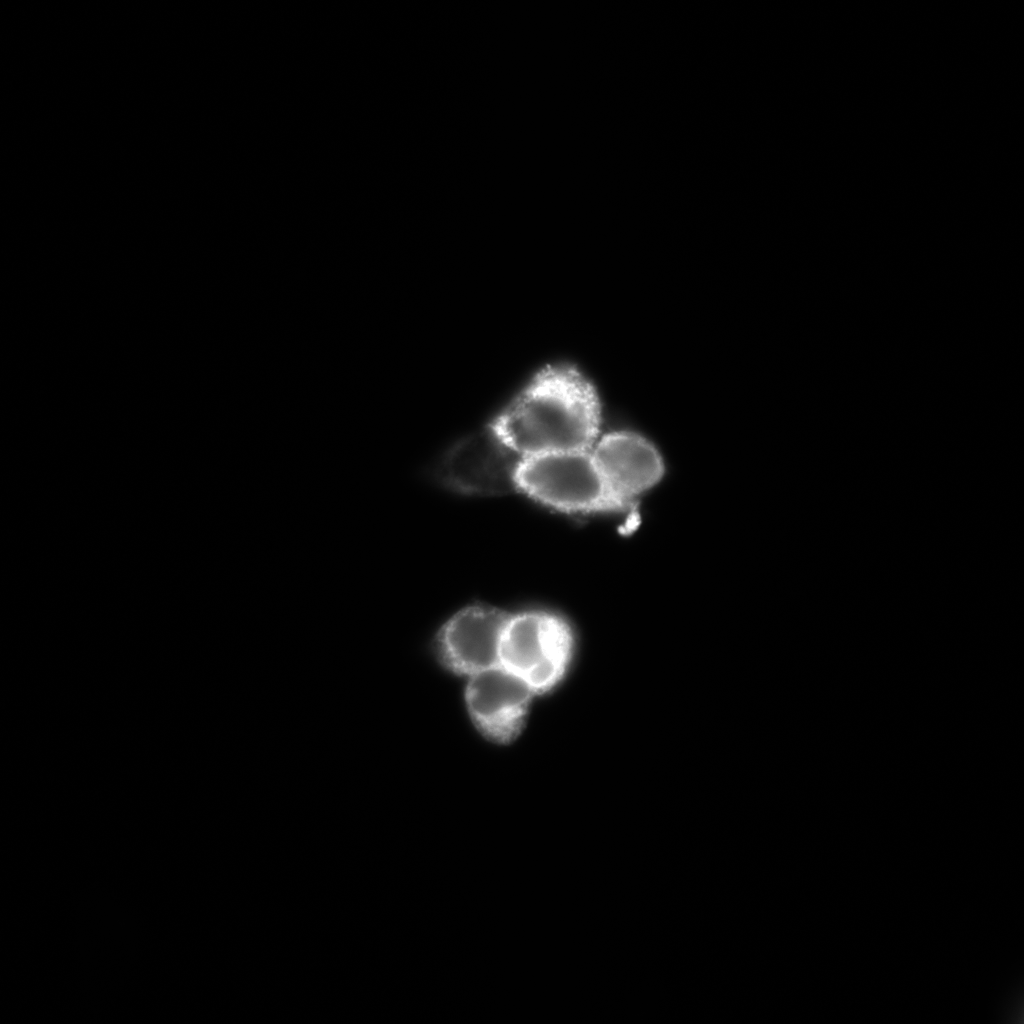

Supplement: Supplementary file 11 — Source Data [file 41467_2024_54263_MOESM11_ESM.zip › Source Data/Fig. 5/Fig. 5 B/Stable probe HEK + 300 probe + 100staygoldcontrol/fluorescence/StayGoldcontrol100ng_1_X1.tif]

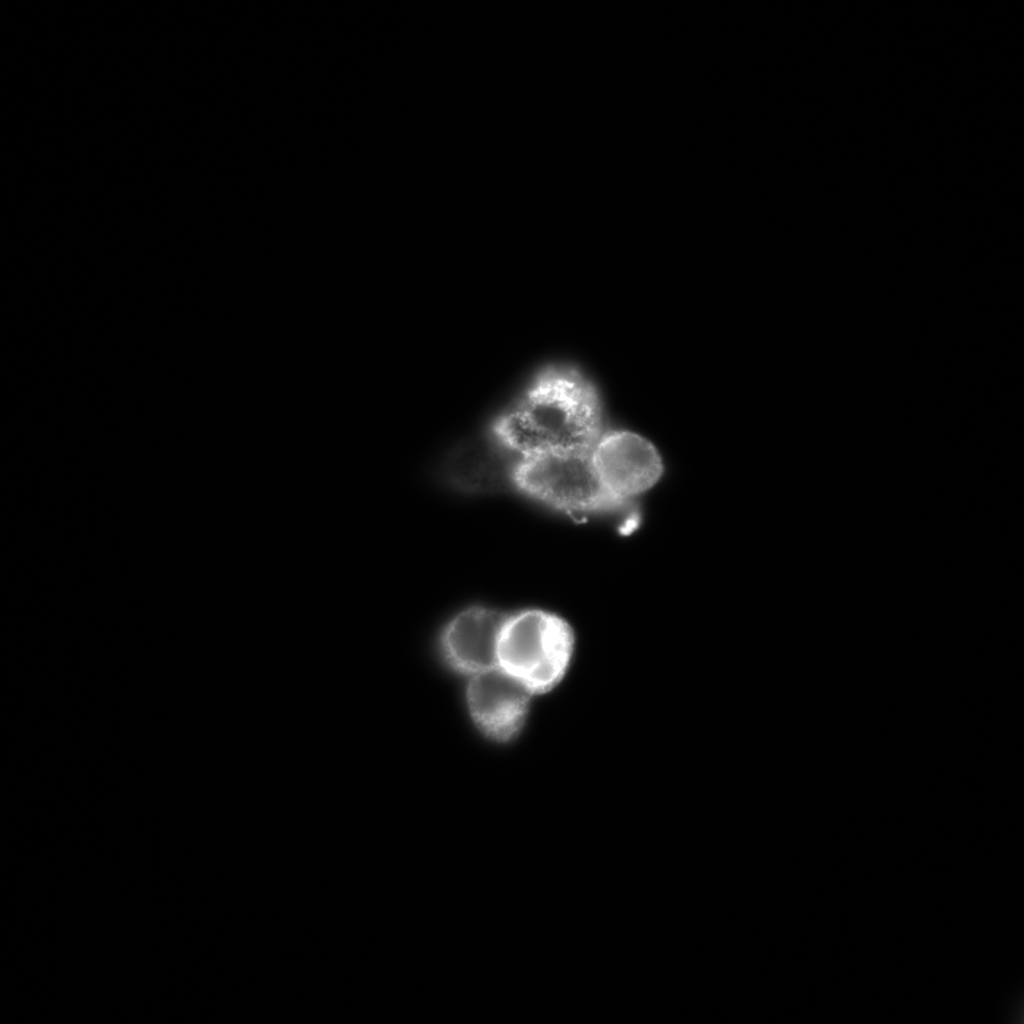

Supplement: Supplementary file 11 — Source Data [file 41467_2024_54263_MOESM11_ESM.zip › Source Data/Fig. 5/Fig. 5 B/Stable probe HEK + 300 probe + 100staygoldcontrol/fluorescence/StayGoldcontrol100ng_3_X1.tif]

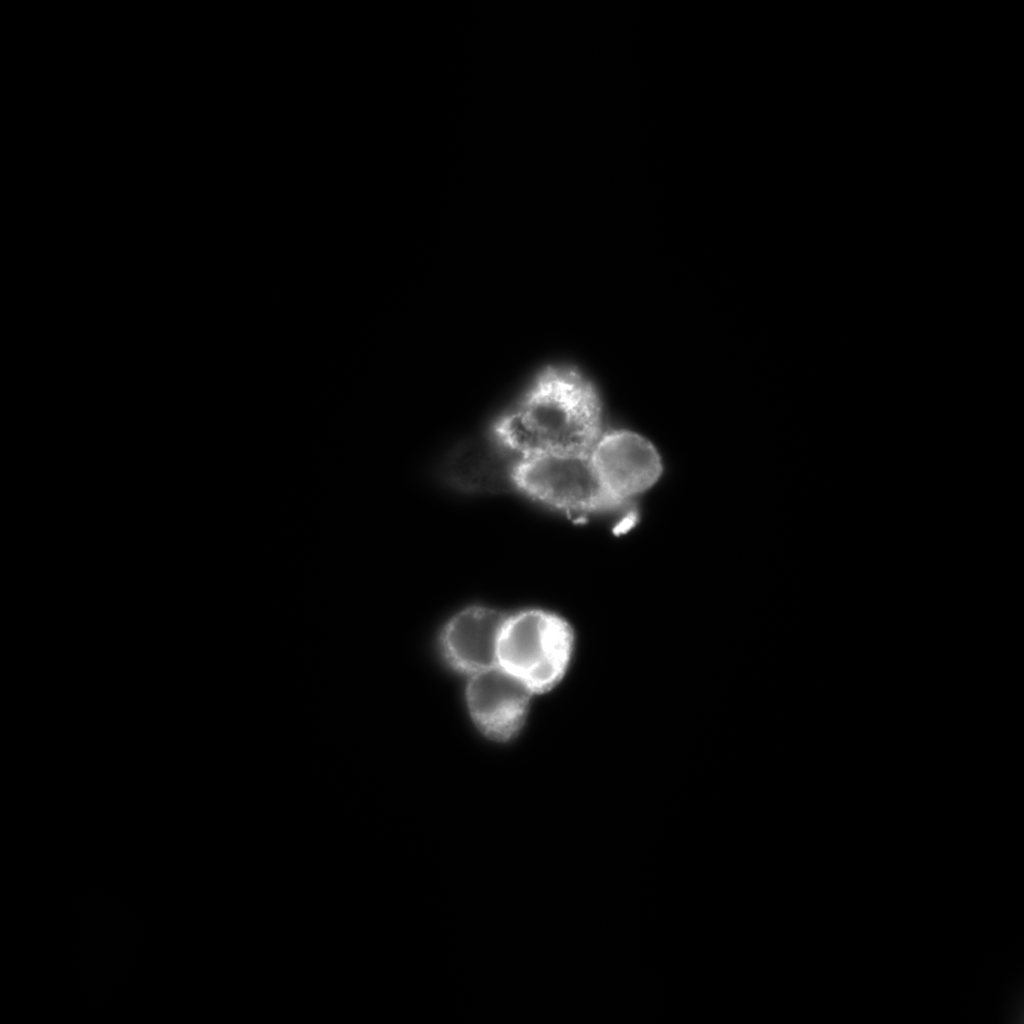

Supplement: Supplementary file 11 — Source Data [file 41467_2024_54263_MOESM11_ESM.zip › Source Data/Fig. 5/Fig. 5 B/Stable probe HEK + 300 probe + 100staygoldcontrol/fluorescence/StayGoldcontrol100ng_19_X1.tif]

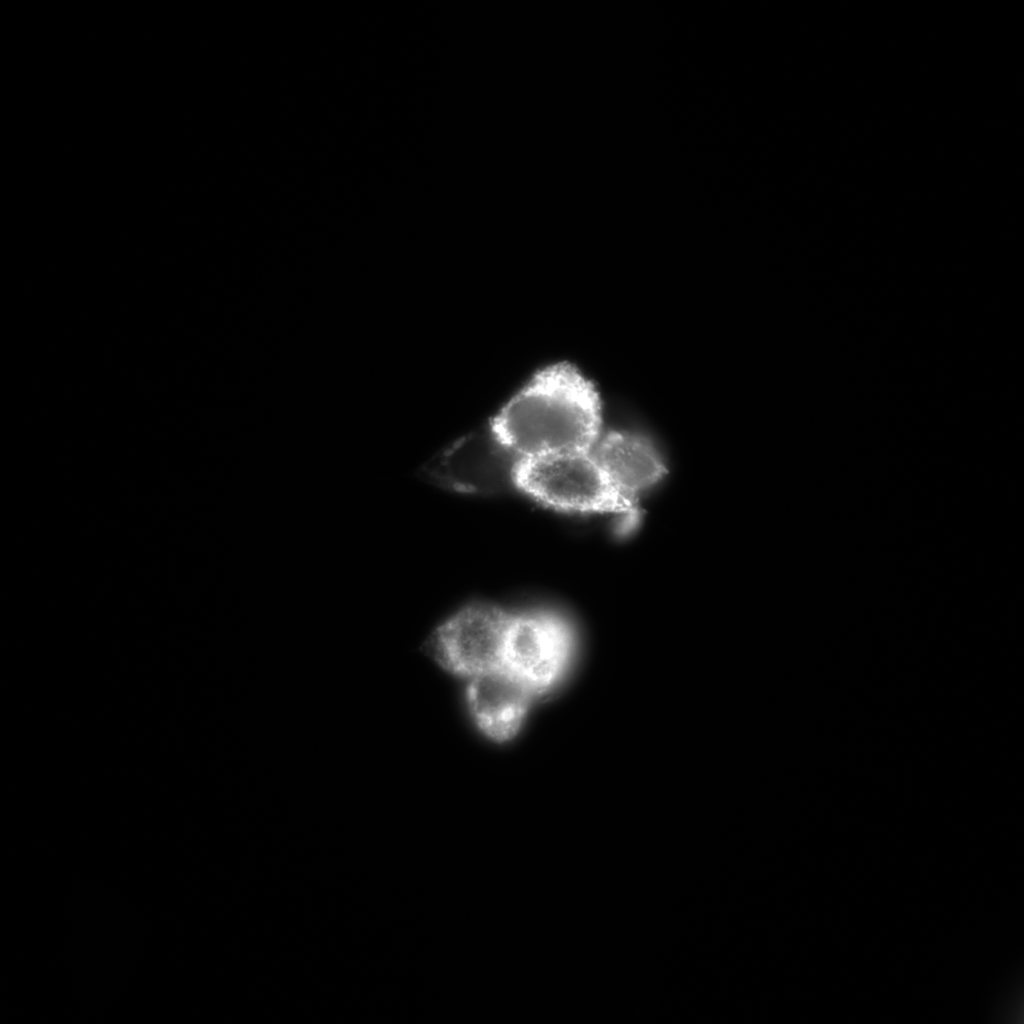

Supplement: Supplementary file 11 — Source Data [file 41467_2024_54263_MOESM11_ESM.zip › Source Data/Fig. 5/Fig. 5 B/Stable probe HEK + 300 probe + 100staygoldcontrol/fluorescence/StayGoldcontrol100ng_15_X1.tif]

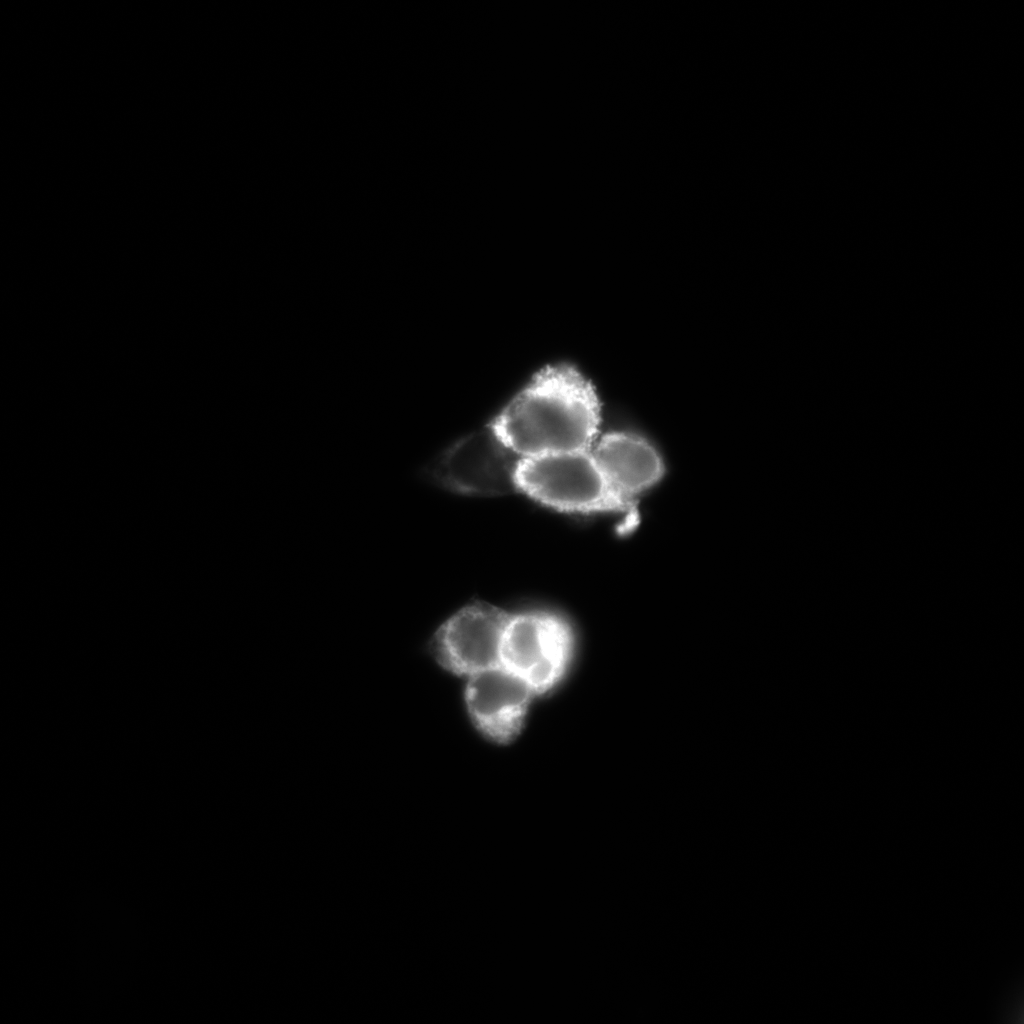

Supplement: Supplementary file 11 — Source Data [file 41467_2024_54263_MOESM11_ESM.zip › Source Data/Fig. 5/Fig. 5 B/Stable probe HEK + 300 probe + 100staygoldcontrol/fluorescence/StayGoldcontrol100ng_7_X1.tif]

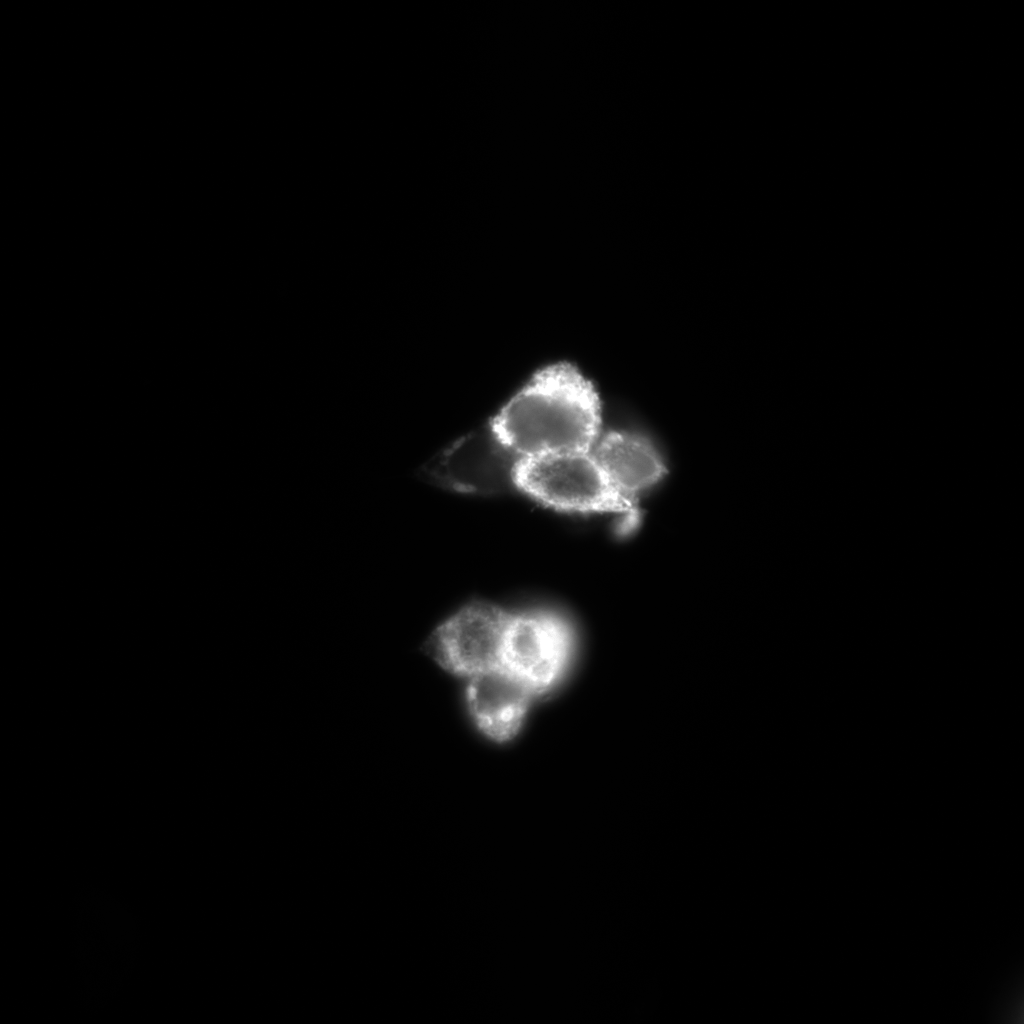

Supplement: Supplementary file 11 — Source Data [file 41467_2024_54263_MOESM11_ESM.zip › Source Data/Fig. 5/Fig. 5 B/Stable probe HEK + 300 probe + 100staygoldcontrol/fluorescence/StayGoldcontrol100ng_11_X1.tif]

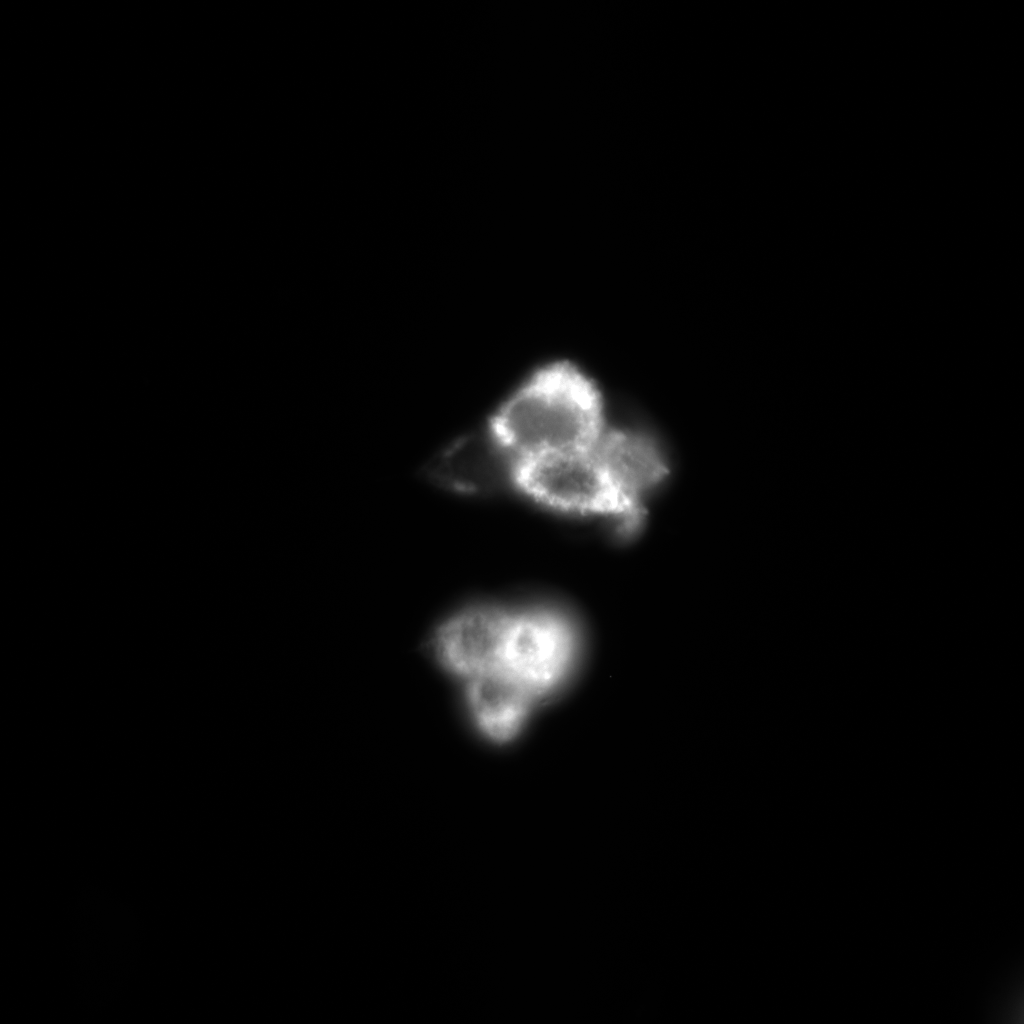

Supplement: Supplementary file 11 — Source Data [file 41467_2024_54263_MOESM11_ESM.zip › Source Data/Fig. 5/Fig. 5 B/Stable probe HEK + 300 probe + 100staygoldcontrol/fluorescence/StayGoldcontrol100ng_13_X1.tif]

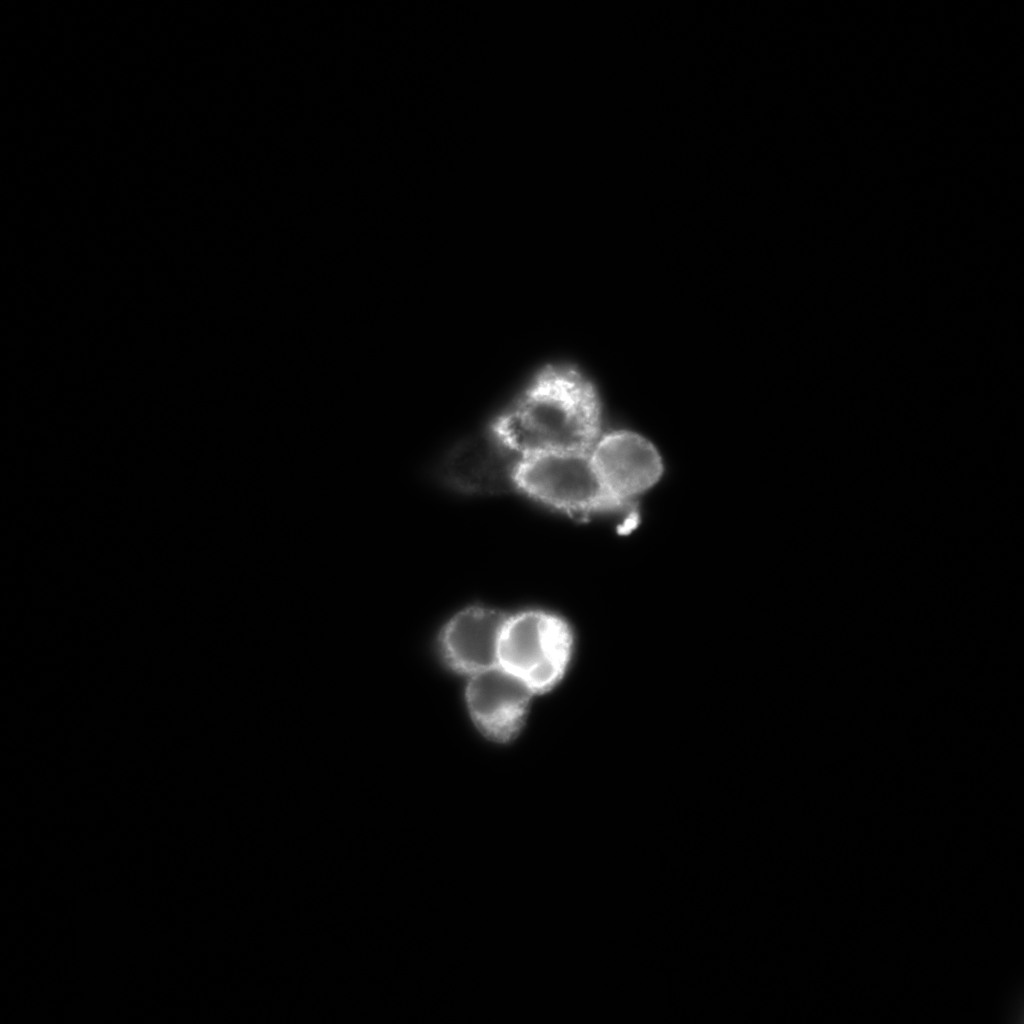

Supplement: Supplementary file 11 — Source Data [file 41467_2024_54263_MOESM11_ESM.zip › Source Data/Fig. 5/Fig. 5 B/Stable probe HEK + 300 probe + 100staygoldcontrol/fluorescence/StayGoldcontrol100ng_5_X1.tif]

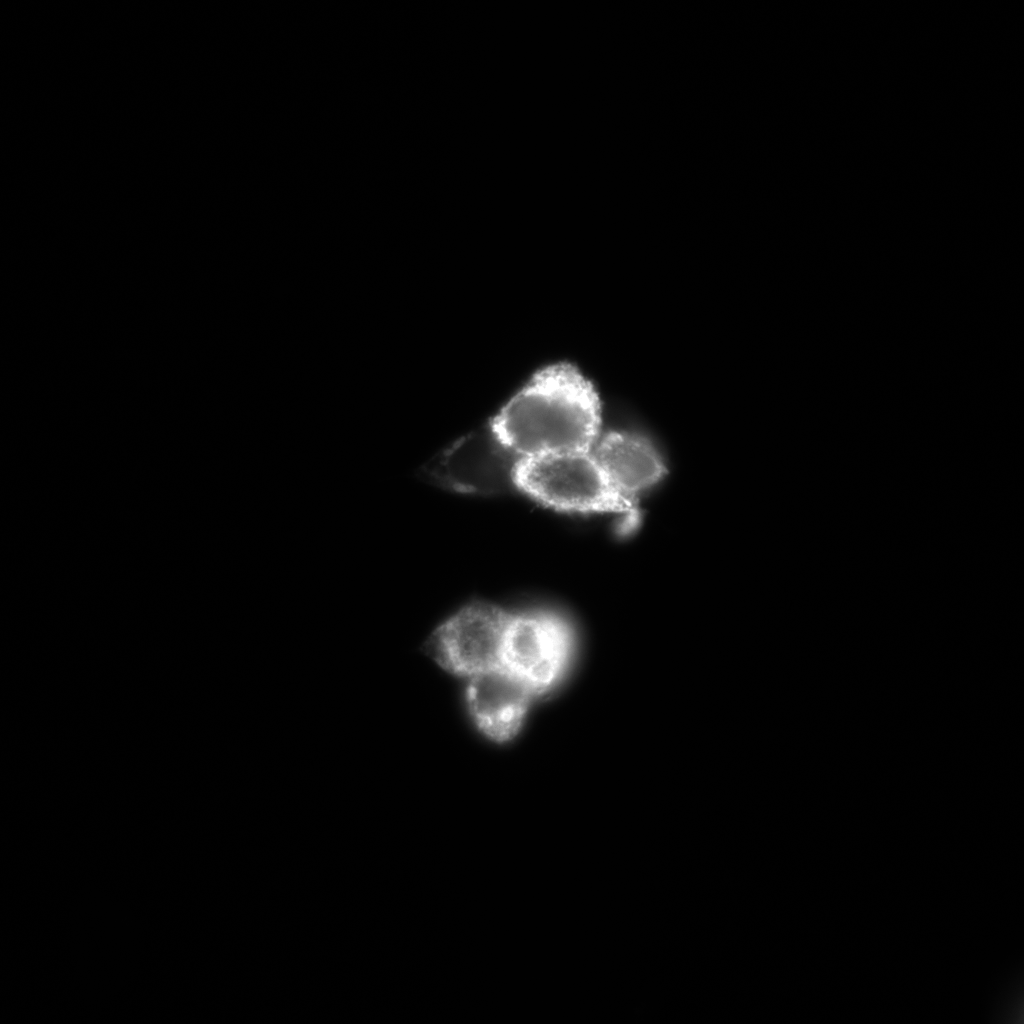

Supplement: Supplementary file 11 — Source Data [file 41467_2024_54263_MOESM11_ESM.zip › Source Data/Fig. 5/Fig. 5 B/Stable probe HEK + 300 probe + 100staygoldcontrol/fluorescence/StayGoldcontrol100ng_9_X1.tif]

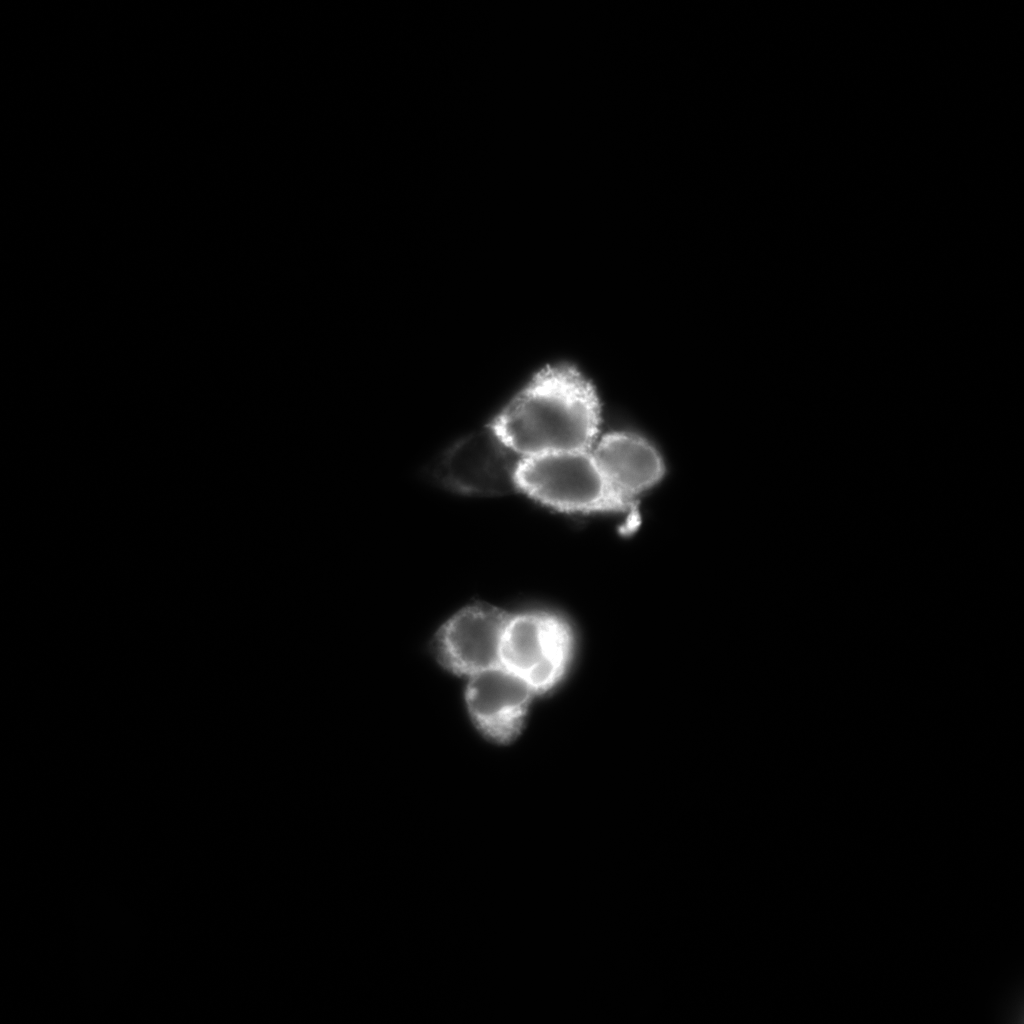

Supplement: Supplementary file 11 — Source Data [file 41467_2024_54263_MOESM11_ESM.zip › Source Data/Fig. 5/Fig. 5 B/Stable probe HEK + 300 probe + 100staygoldcontrol/fluorescence/StayGoldcontrol100ng_0_X1.tif]

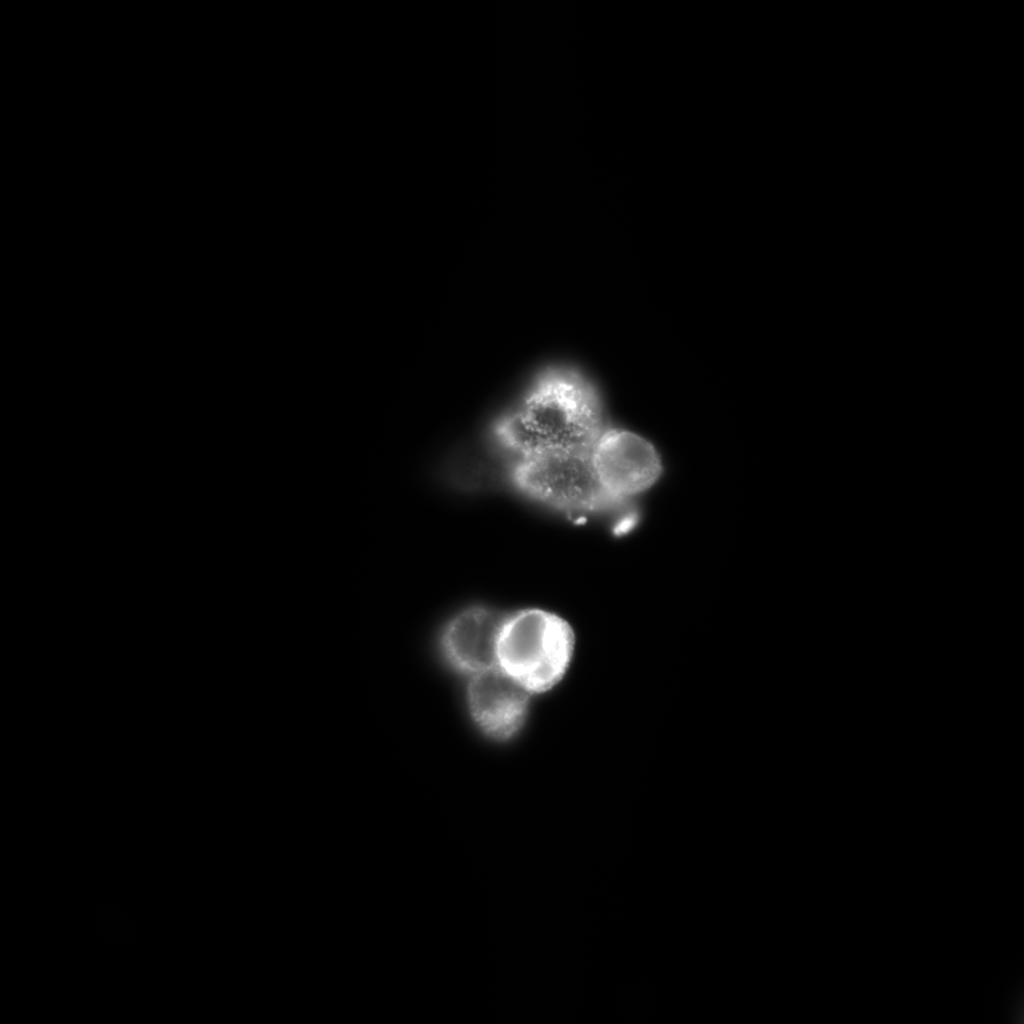

Supplement: Supplementary file 11 — Source Data [file 41467_2024_54263_MOESM11_ESM.zip › Source Data/Fig. 5/Fig. 5 B/Stable probe HEK + 300 probe + 100staygoldcontrol/fluorescence/StayGoldcontrol100ng_20_X1.tif]

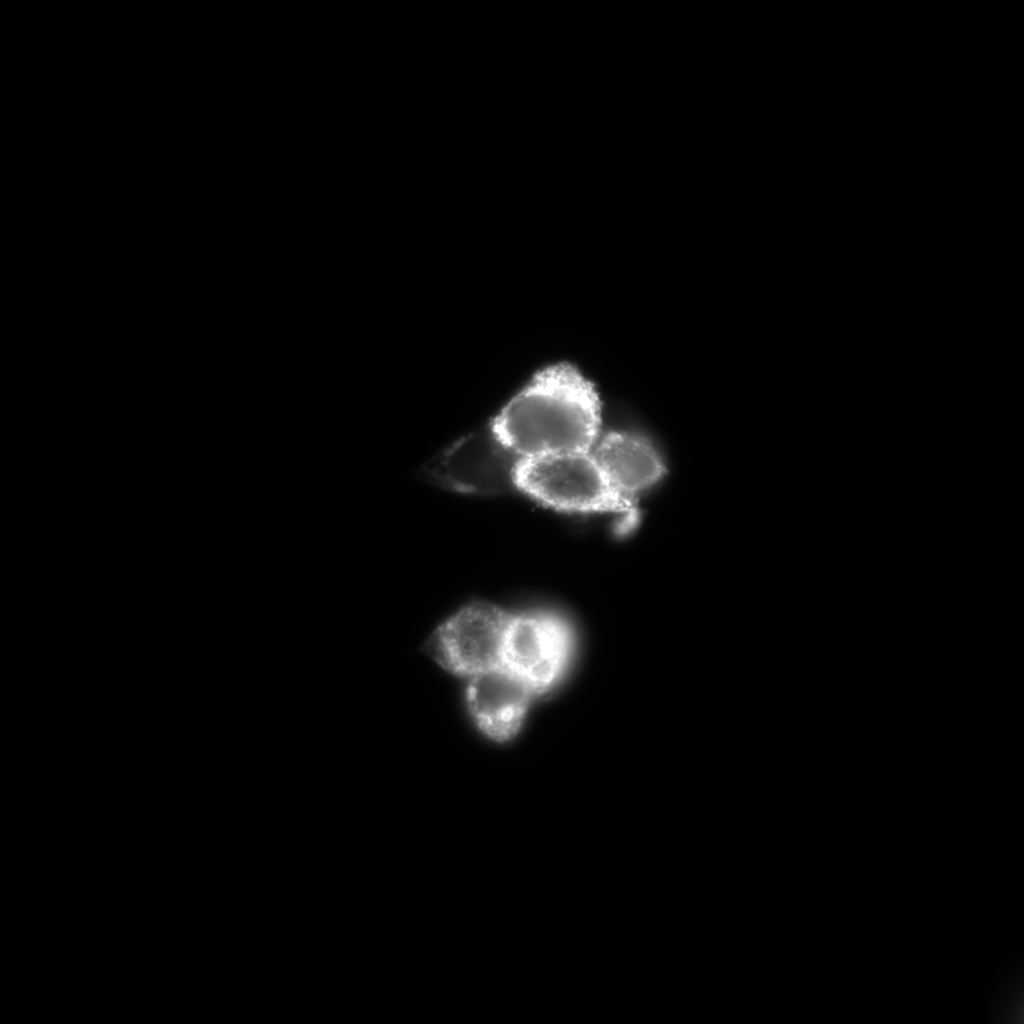

Supplement: Supplementary file 11 — Source Data [file 41467_2024_54263_MOESM11_ESM.zip › Source Data/Fig. 5/Fig. 5 B/Stable probe HEK + 300 probe + 100staygoldcontrol/fluorescence/StayGoldcontrol100ng_16_X1.tif]

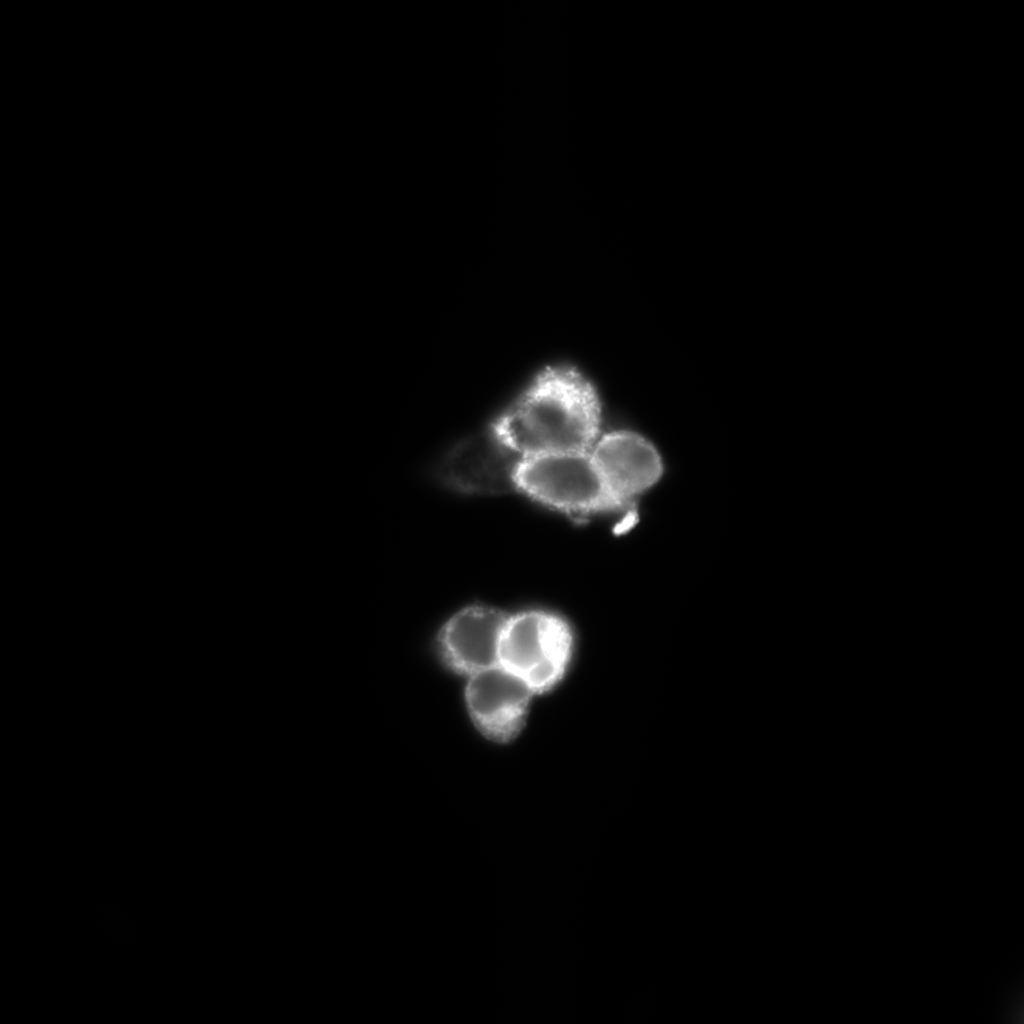

Supplement: Supplementary file 11 — Source Data [file 41467_2024_54263_MOESM11_ESM.zip › Source Data/Fig. 5/Fig. 5 B/Stable probe HEK + 300 probe + 100staygoldcontrol/fluorescence/StayGoldcontrol100ng_18_X1.tif]

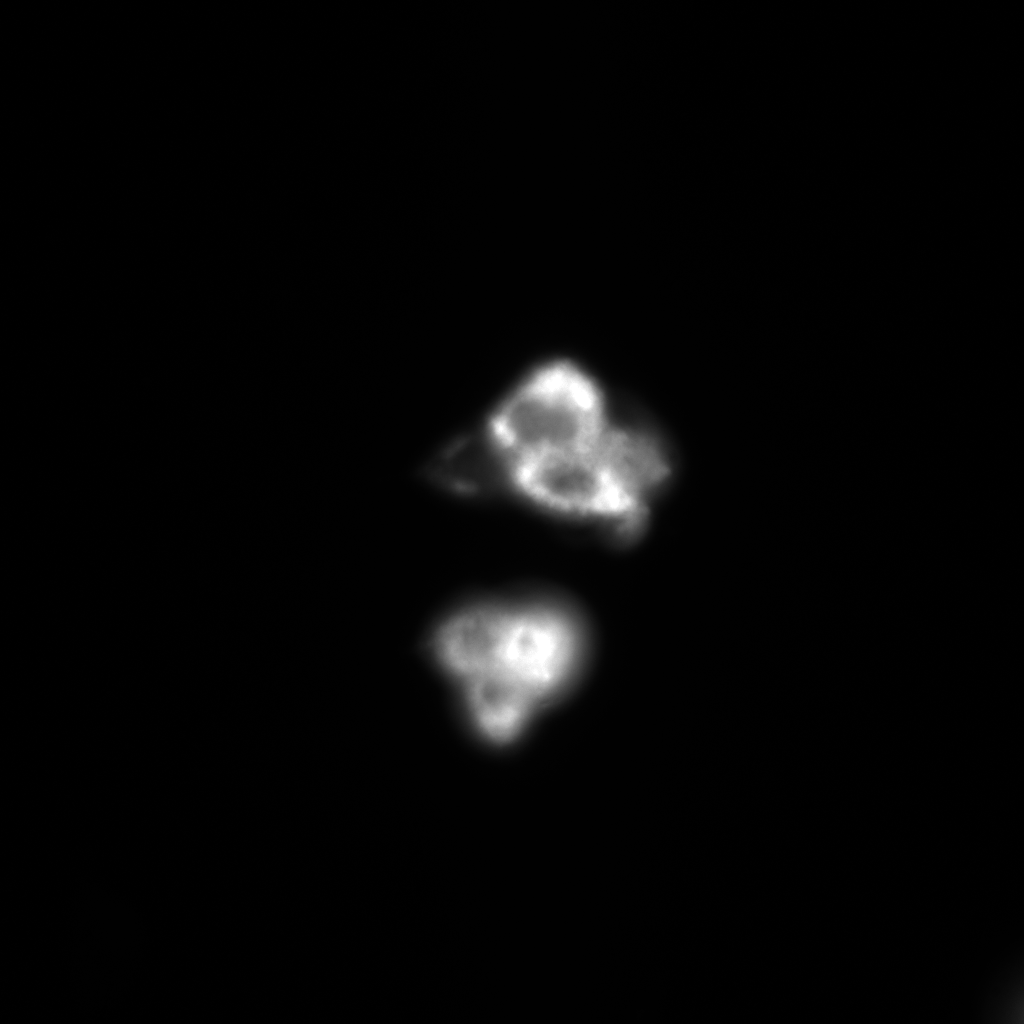

Supplement: Supplementary file 11 — Source Data [file 41467_2024_54263_MOESM11_ESM.zip › Source Data/Fig. 5/Fig. 5 B/Stable probe HEK + 300 probe + 100staygoldcontrol/fluorescence/StayGoldcontrol100ng_14_X1.tif]

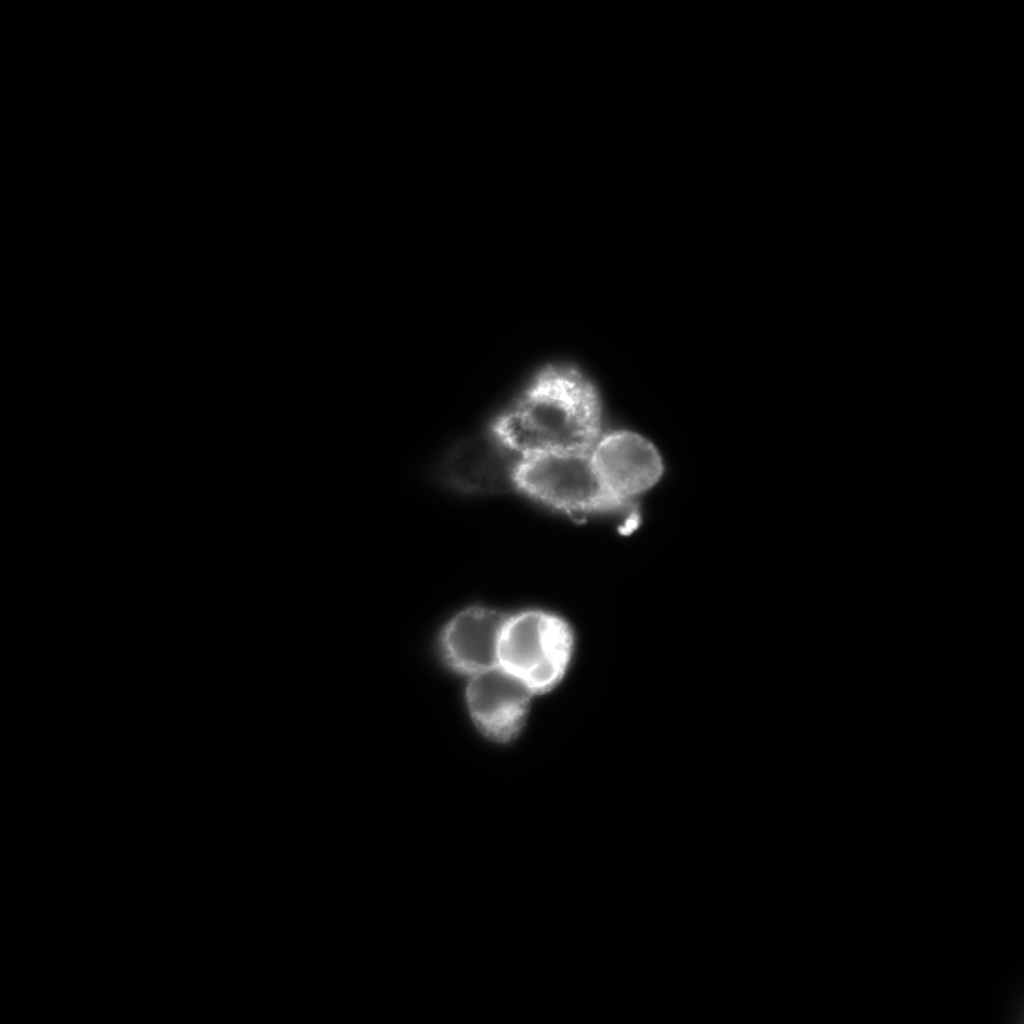

Supplement: Supplementary file 11 — Source Data [file 41467_2024_54263_MOESM11_ESM.zip › Source Data/Fig. 5/Fig. 5 B/Stable probe HEK + 300 probe + 100staygoldcontrol/fluorescence/StayGoldcontrol100ng_2_X1.tif]

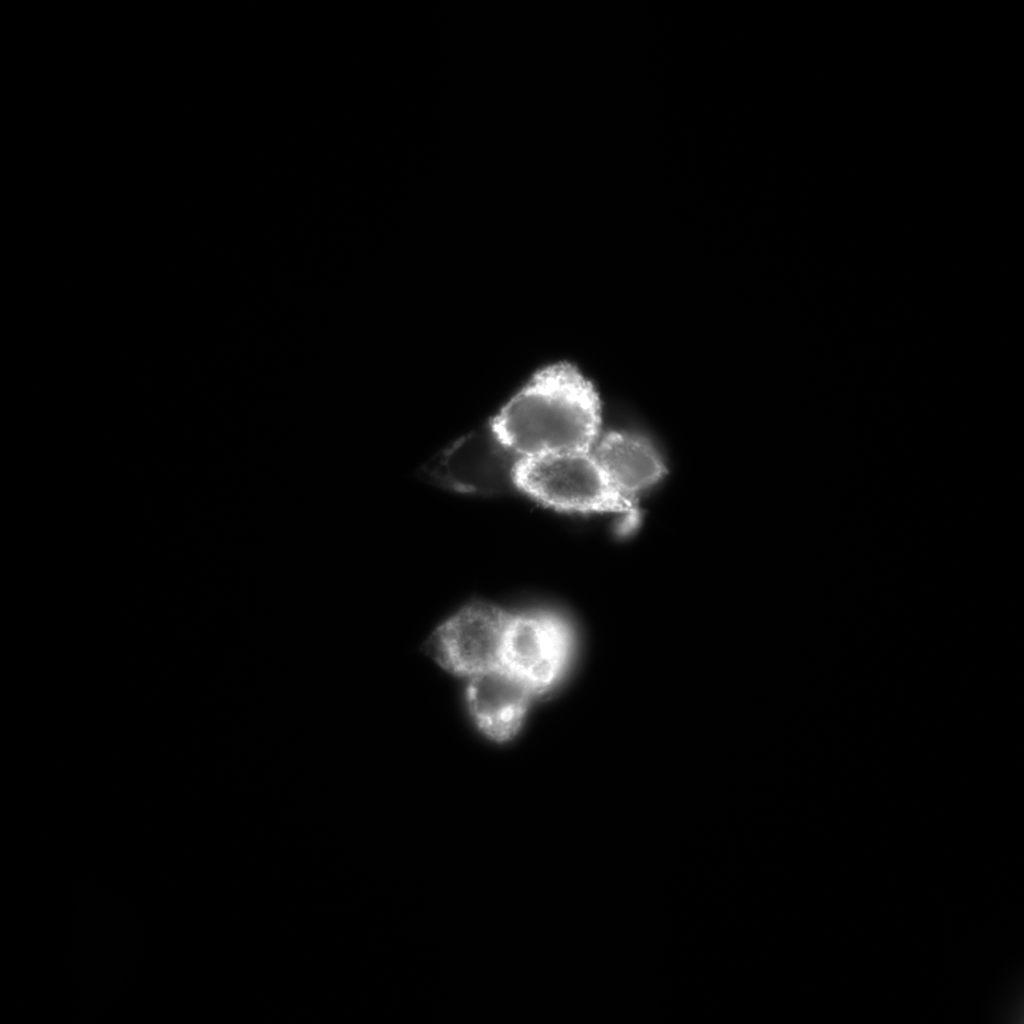

Supplement: Supplementary file 11 — Source Data [file 41467_2024_54263_MOESM11_ESM.zip › Source Data/Fig. 5/Fig. 5 B/Stable probe HEK + 300 probe + 100staygoldcontrol/fluorescence/StayGoldcontrol100ng_10_X1.tif]

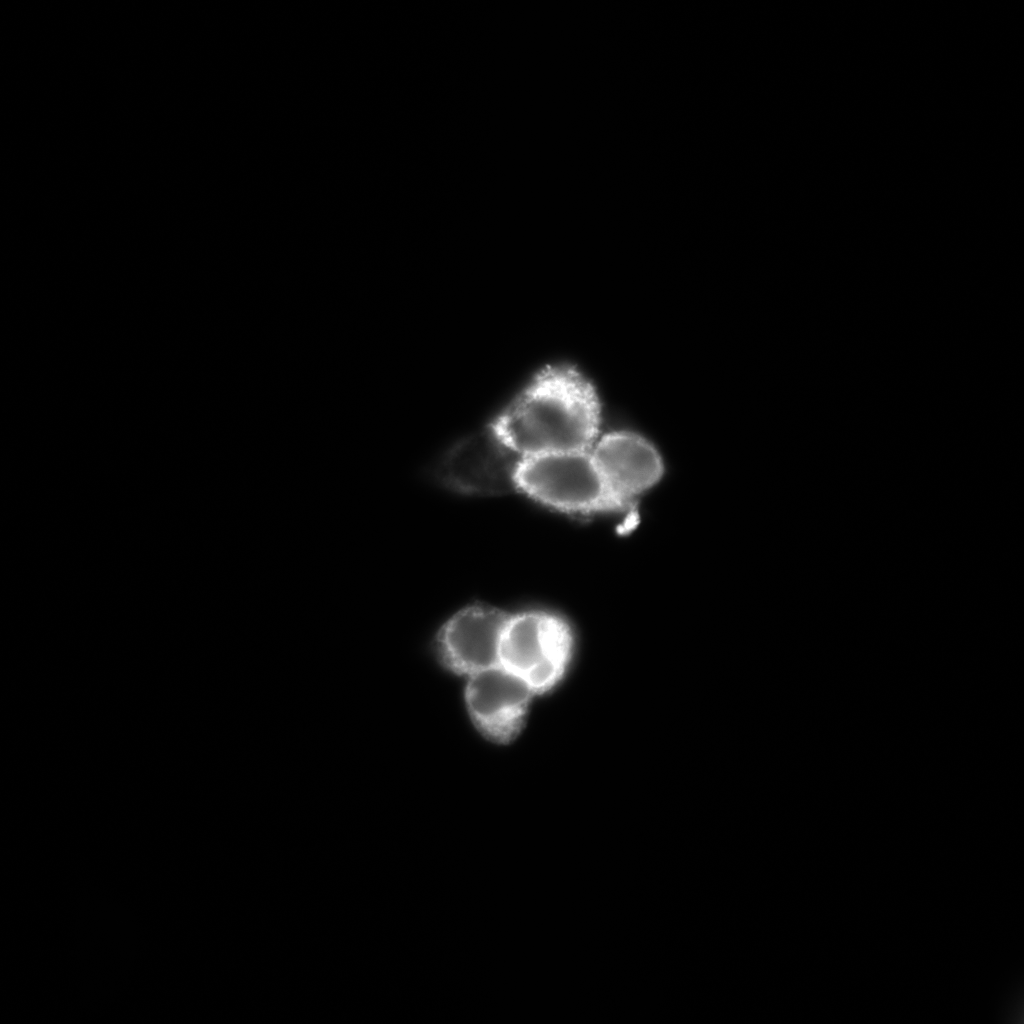

Supplement: Supplementary file 11 — Source Data [file 41467_2024_54263_MOESM11_ESM.zip › Source Data/Fig. 5/Fig. 5 B/Stable probe HEK + 300 probe + 100staygoldcontrol/fluorescence/StayGoldcontrol100ng_6_X1.tif]

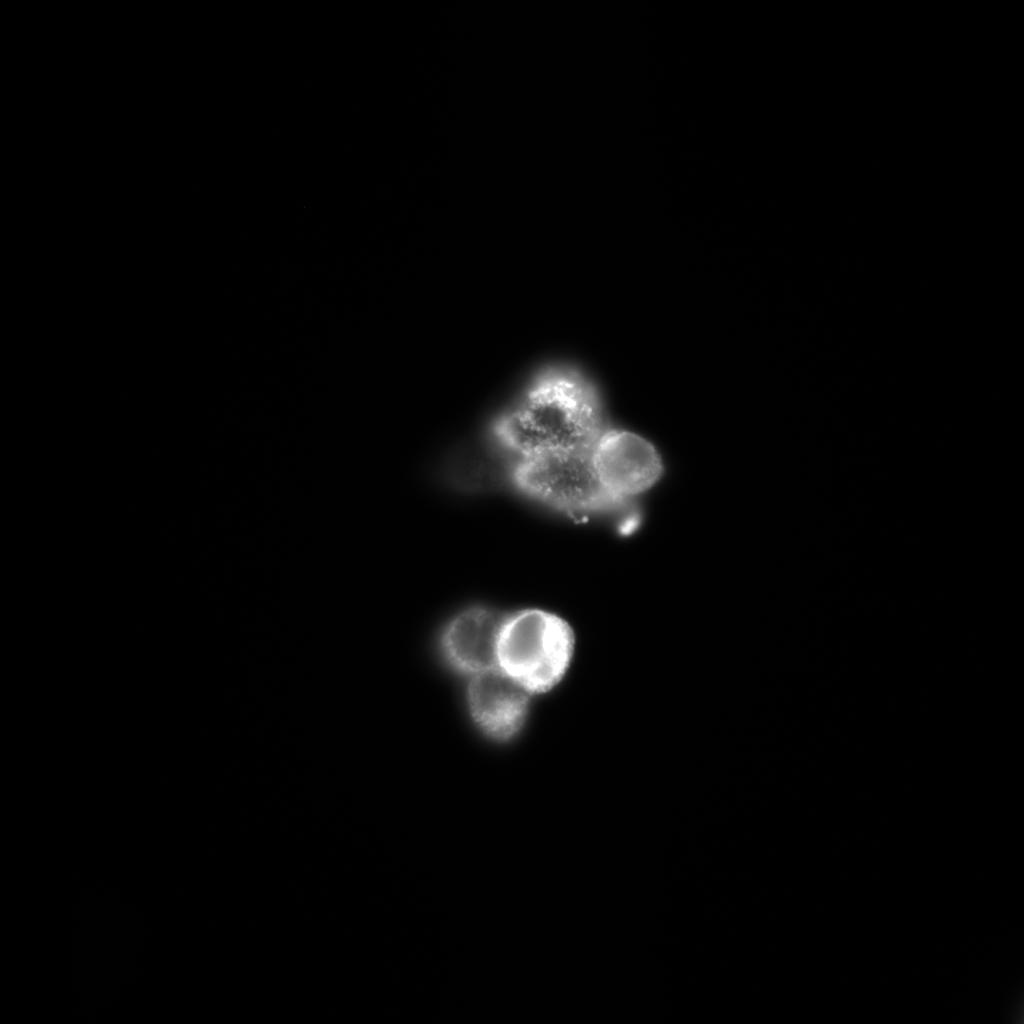

Supplement: Supplementary file 11 — Source Data [file 41467_2024_54263_MOESM11_ESM.zip › Source Data/Fig. 5/Fig. 5 B/Stable probe HEK + 300 probe + 100staygoldcontrol/fluorescence/StayGoldcontrol100ng_4_X1.tif]

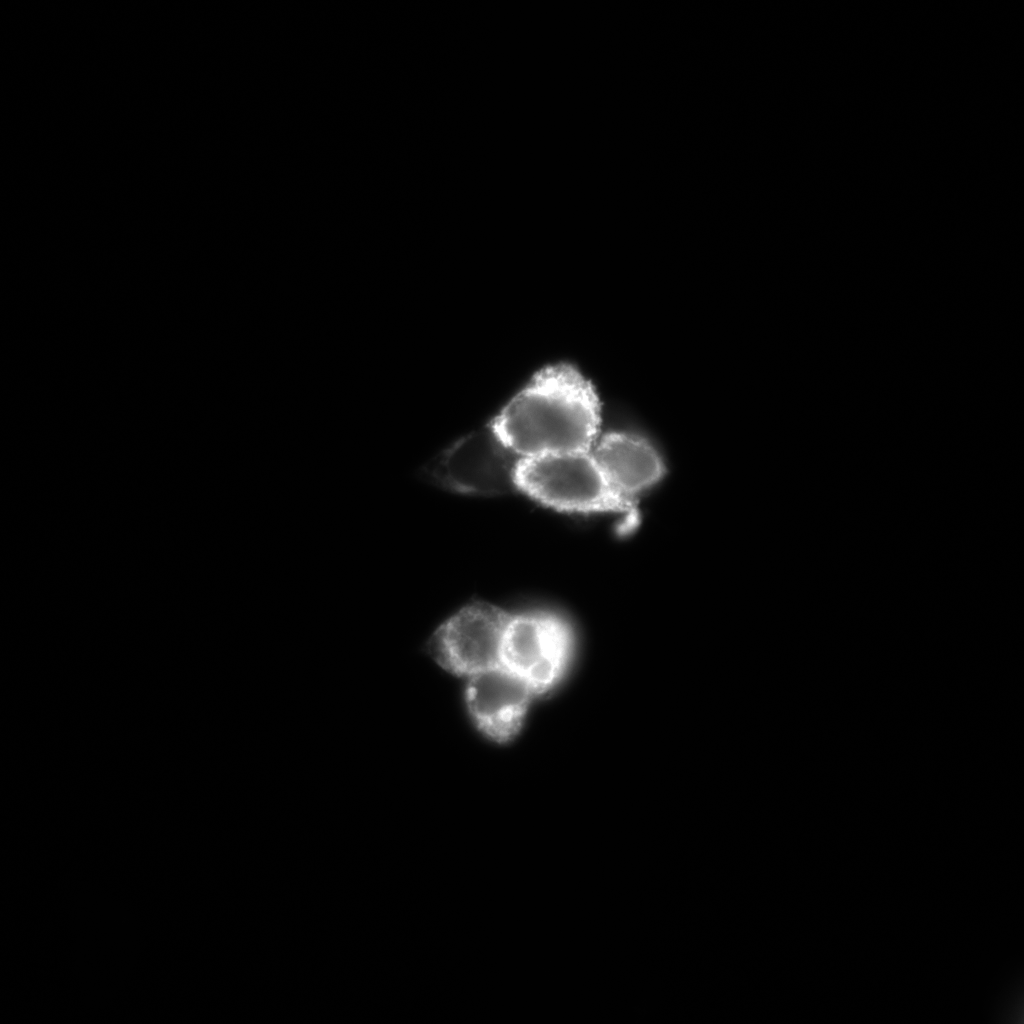

Supplement: Supplementary file 11 — Source Data [file 41467_2024_54263_MOESM11_ESM.zip › Source Data/Fig. 5/Fig. 5 B/Stable probe HEK + 300 probe + 100staygoldcontrol/fluorescence/StayGoldcontrol100ng_8_X1.tif]

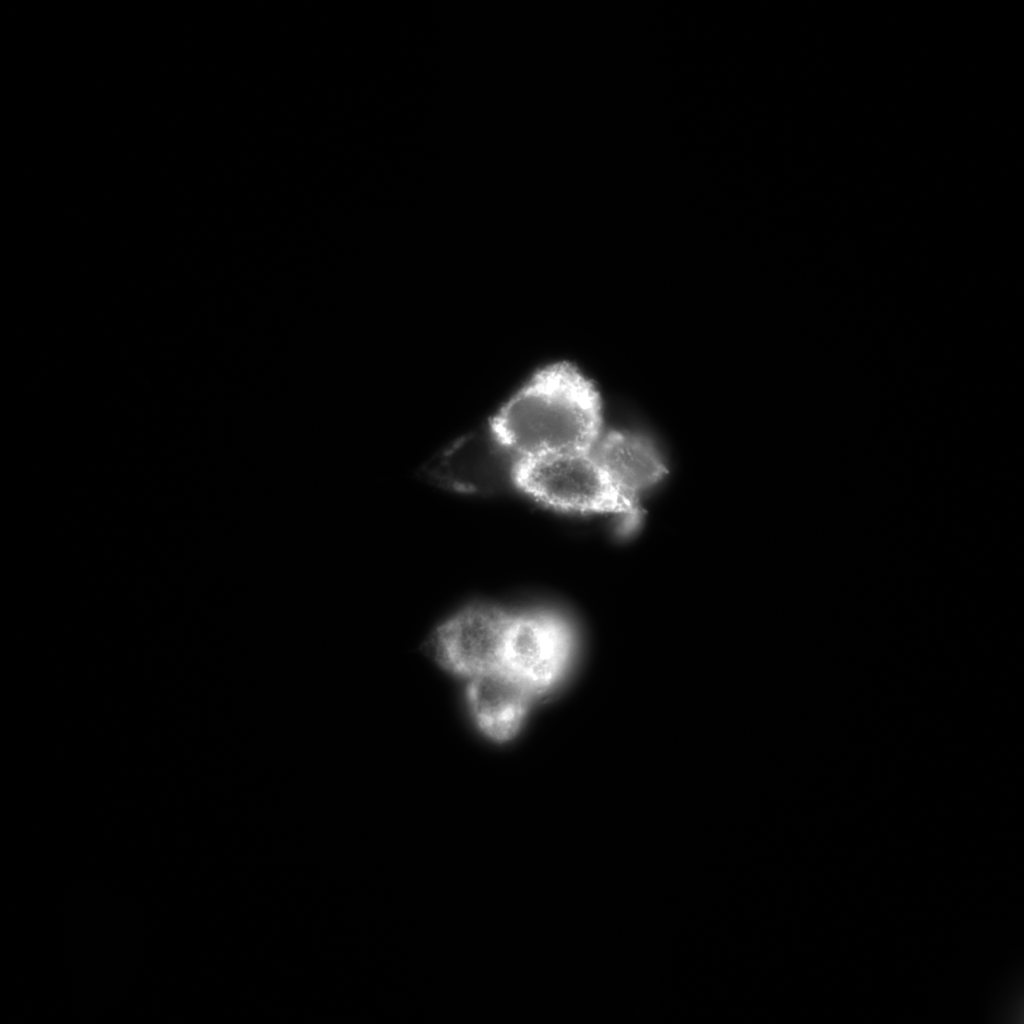

Supplement: Supplementary file 11 — Source Data [file 41467_2024_54263_MOESM11_ESM.zip › Source Data/Fig. 5/Fig. 5 B/Stable probe HEK + 300 probe + 100staygoldcontrol/fluorescence/StayGoldcontrol100ng_12_X1.tif]

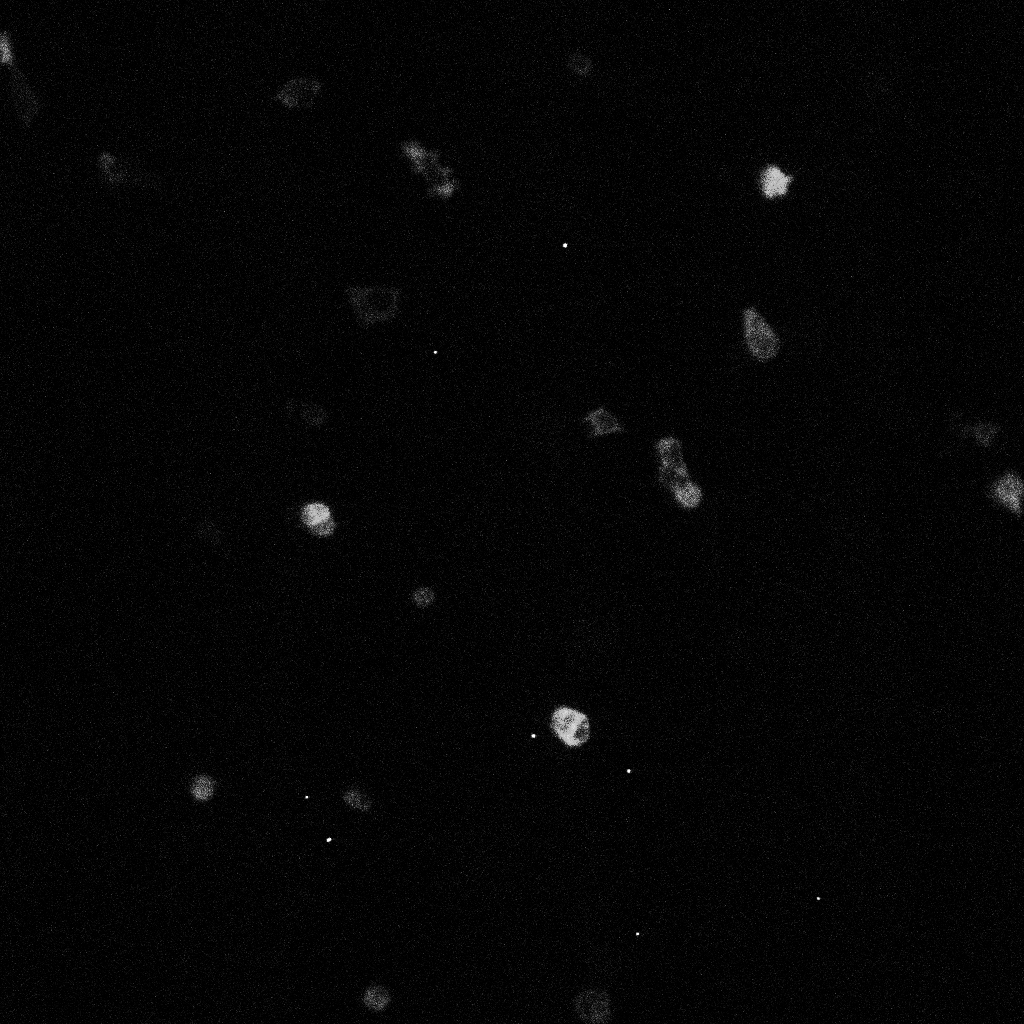

Supplement: Supplementary file 11 — Source Data [file 41467_2024_54263_MOESM11_ESM.zip › Source Data/Fig. 5/Fig. 5 B/Stable probe HEK + 300 probe + 100 staygoldm3p/lumi/20x/Lumi20x180s_1_X1.tif]

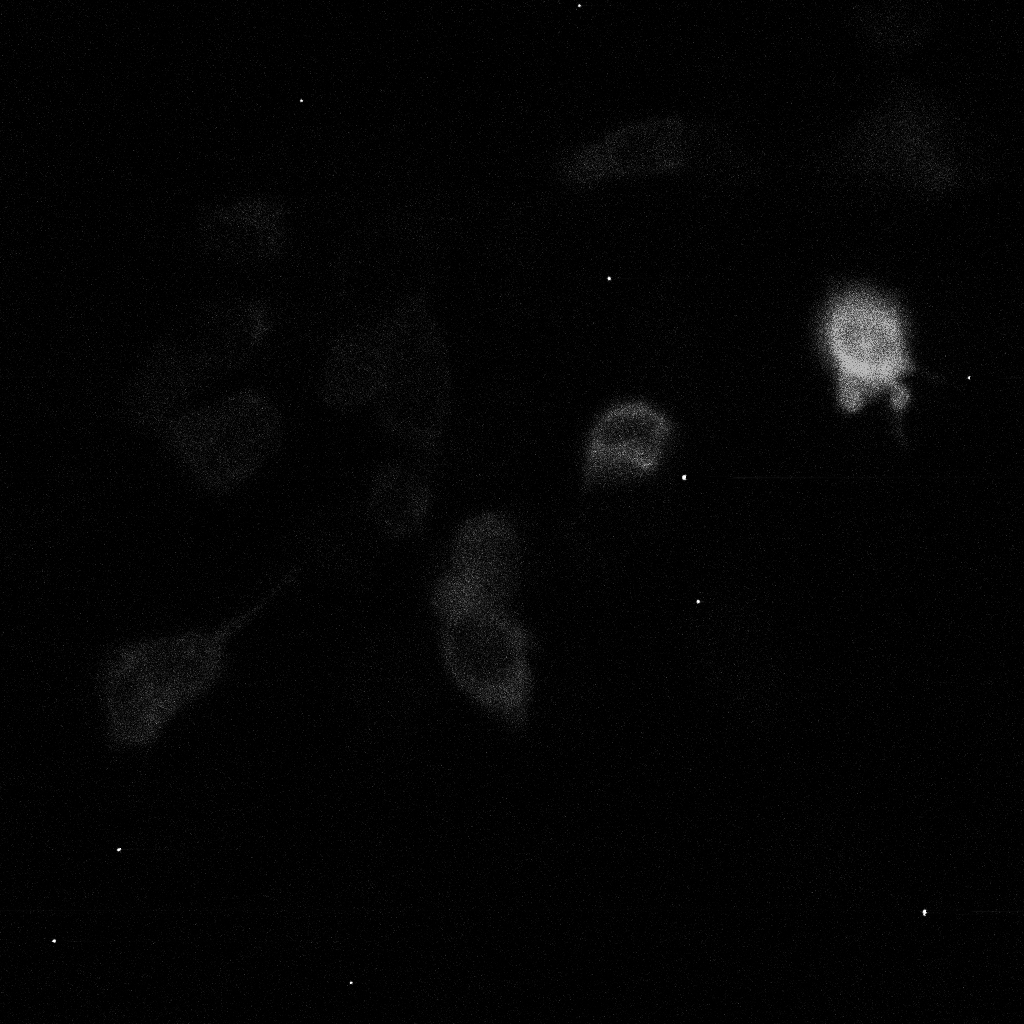

Supplement: Supplementary file 11 — Source Data [file 41467_2024_54263_MOESM11_ESM.zip › Source Data/Fig. 5/Fig. 5 B/Stable probe HEK + 300 probe + 100 staygoldm3p/lumi/3rd/Lumi180s_1_X1.tif]

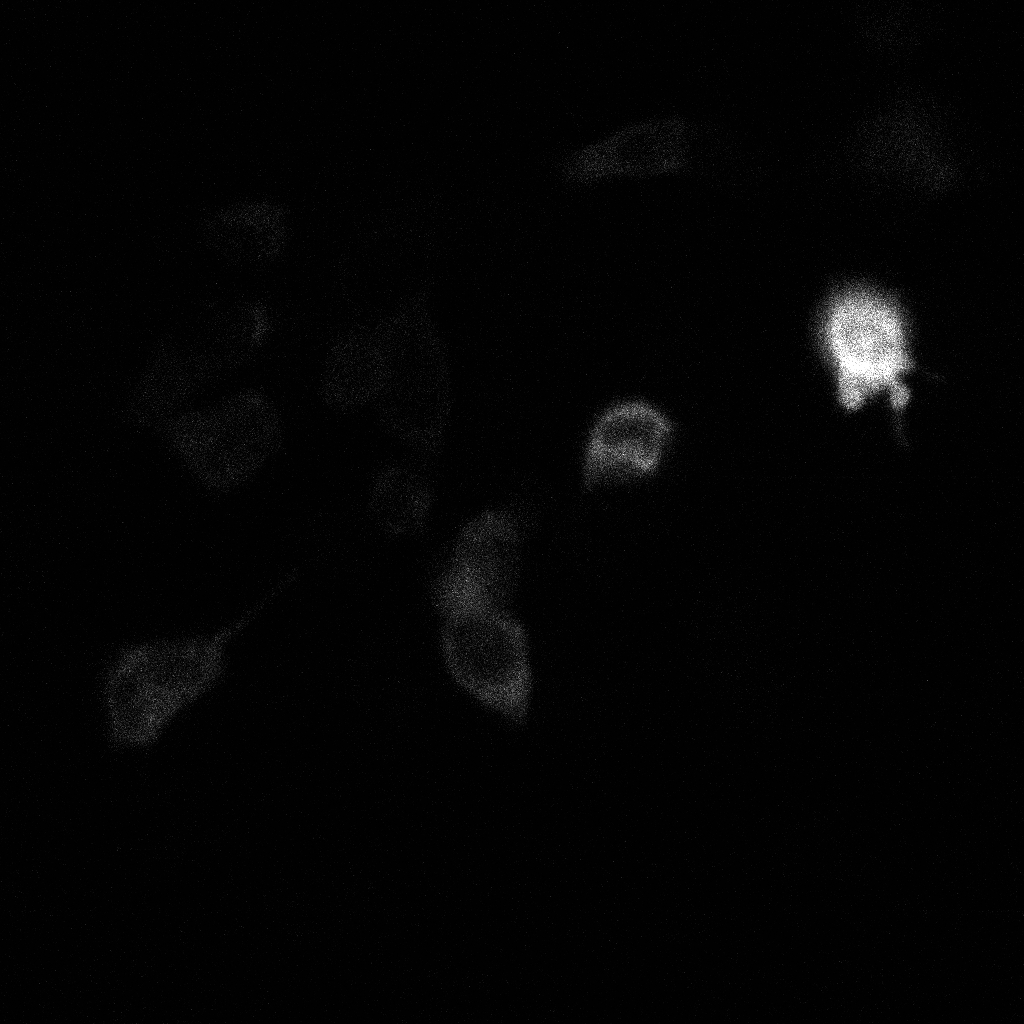

Supplement: Supplementary file 11 — Source Data [file 41467_2024_54263_MOESM11_ESM.zip › Source Data/Fig. 5/Fig. 5 B/Stable probe HEK + 300 probe + 100 staygoldm3p/lumi/3rd/Lumi180s_1_X2.tif]

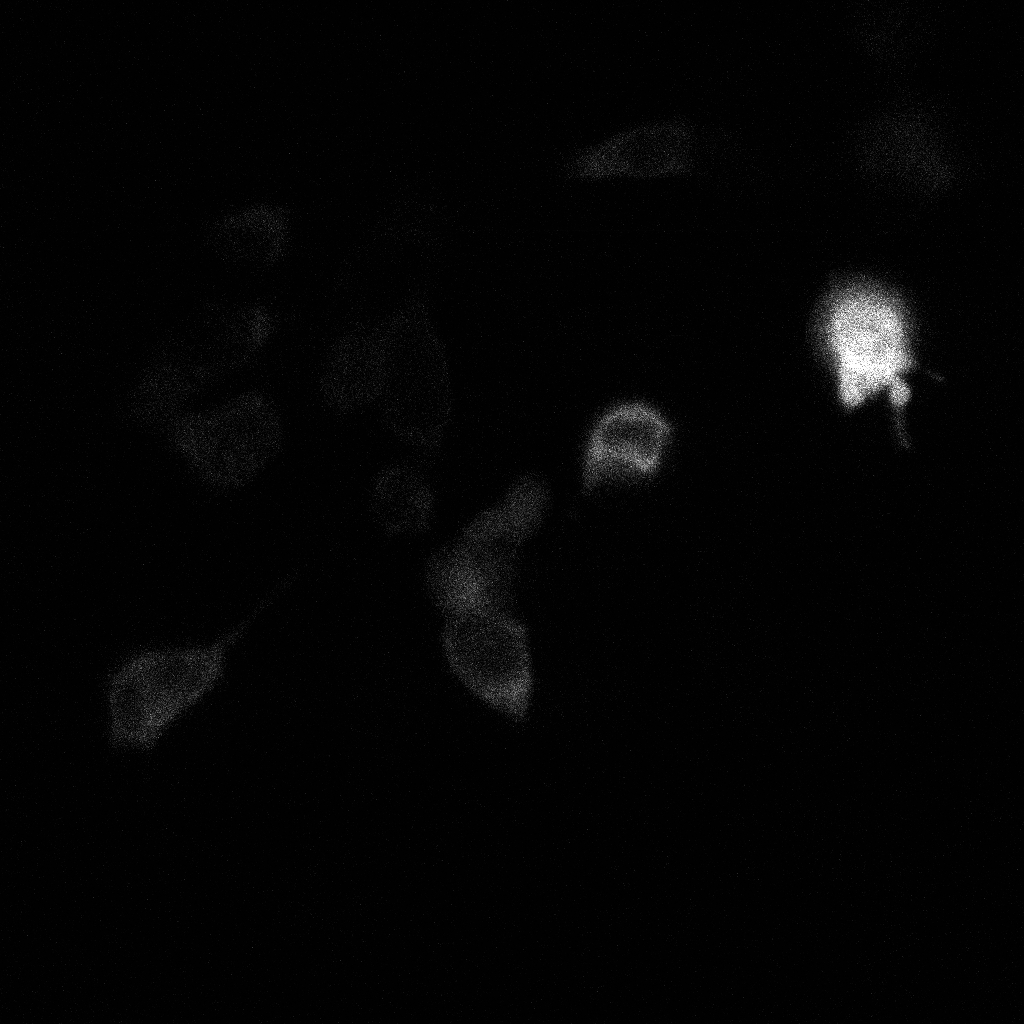

Supplement: Supplementary file 11 — Source Data [file 41467_2024_54263_MOESM11_ESM.zip › Source Data/Fig. 5/Fig. 5 B/Stable probe HEK + 300 probe + 100 staygoldm3p/lumi/3rd/Lumi180s_1_X3.tif]

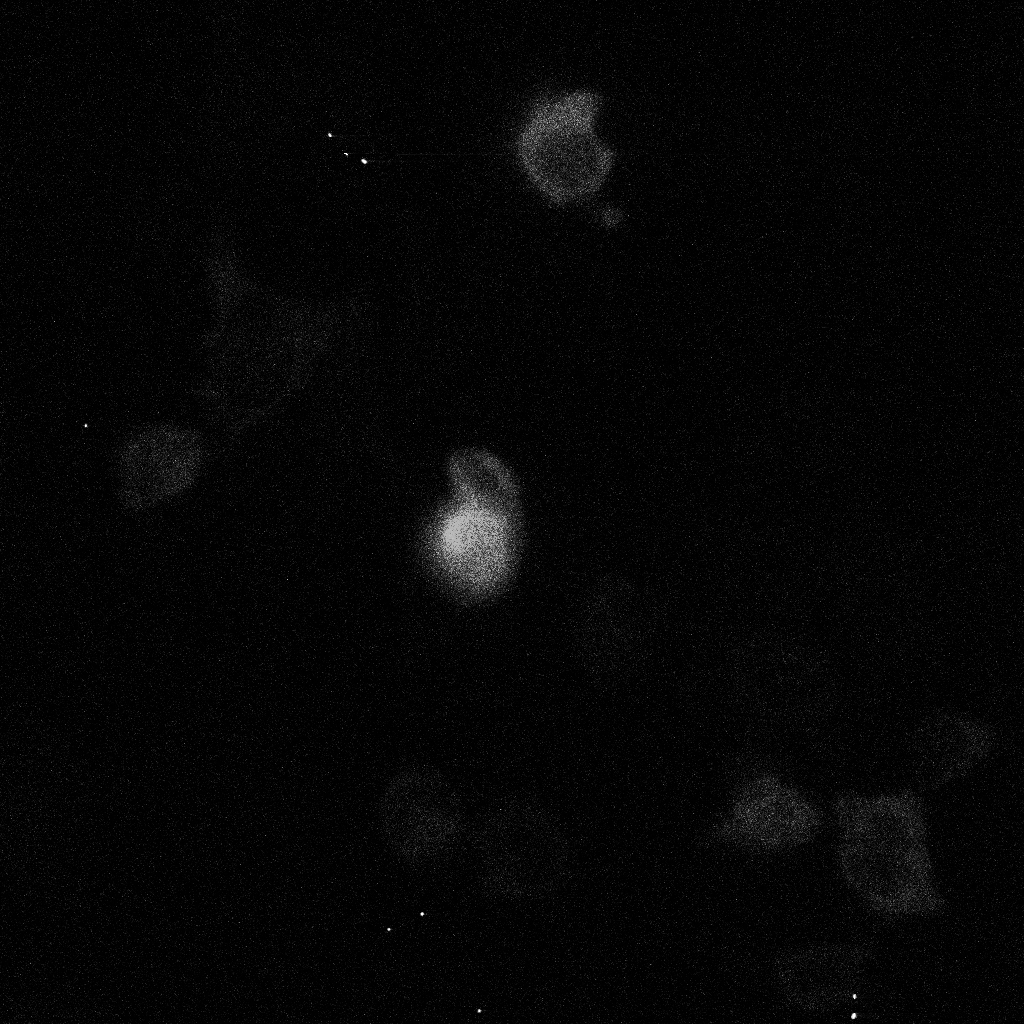

Supplement: Supplementary file 11 — Source Data [file 41467_2024_54263_MOESM11_ESM.zip › Source Data/Fig. 5/Fig. 5 B/Stable probe HEK + 300 probe + 100 staygoldm3p/lumi/2nd/Lumi180s_1_X1.tif]

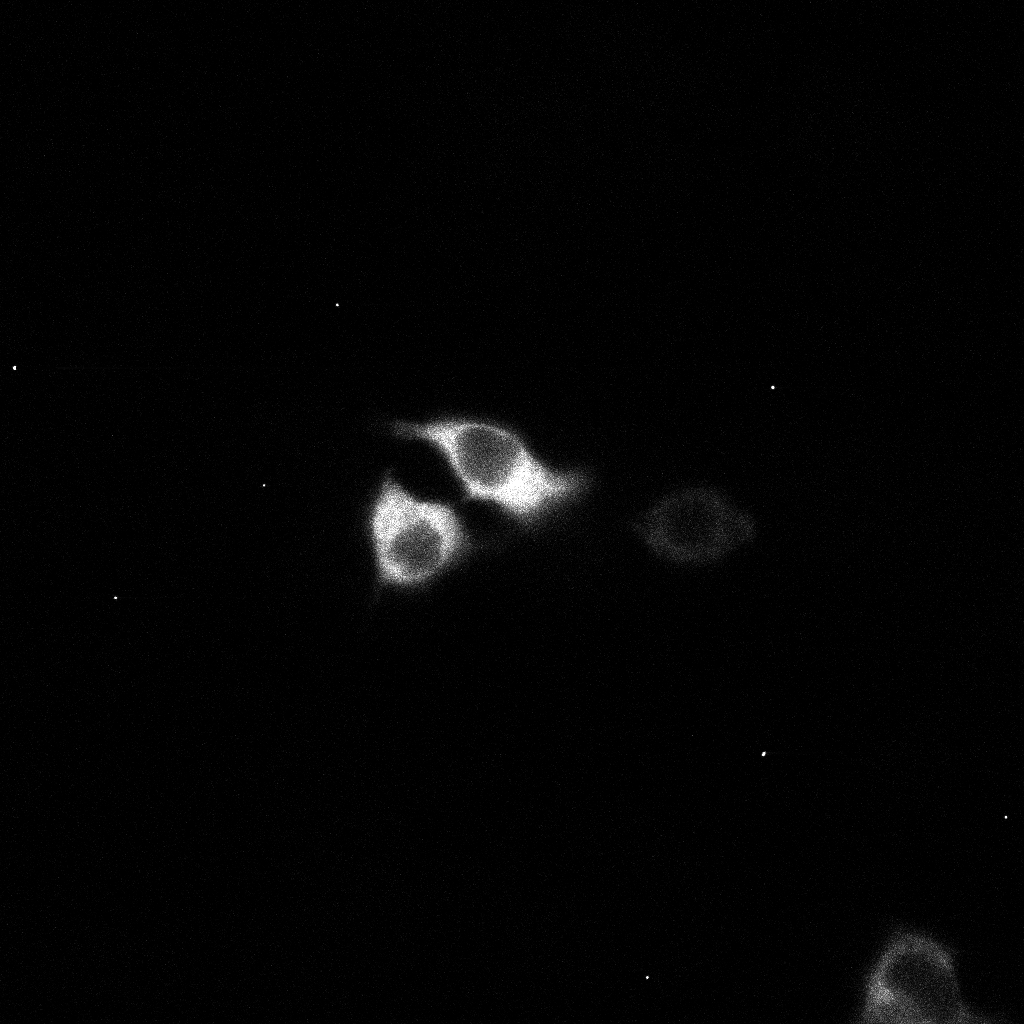

Supplement: Supplementary file 11 — Source Data [file 41467_2024_54263_MOESM11_ESM.zip › Source Data/Fig. 5/Fig. 5 B/Stable probe HEK + 300 probe + 100 staygoldm3p/edited/green_blue/Stack-lumi.tif]

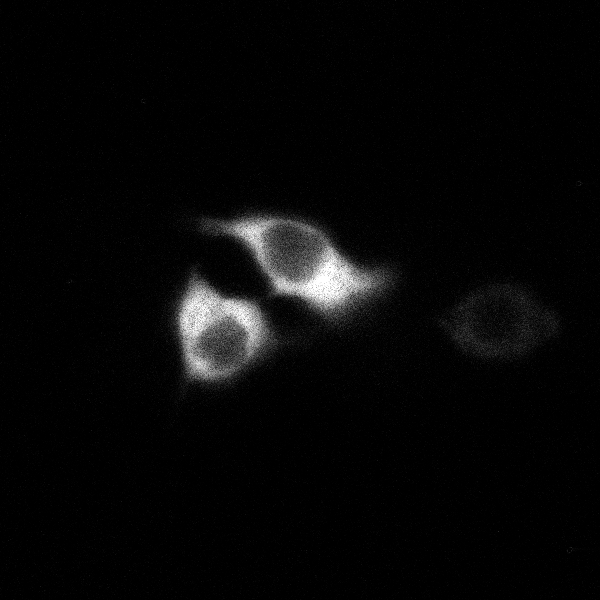

Supplement: Supplementary file 11 — Source Data [file 41467_2024_54263_MOESM11_ESM.zip › Source Data/Fig. 5/Fig. 5 B/Stable probe HEK + 300 probe + 100 staygoldm3p/edited/green_blue/Composite-600-ROI.tif]

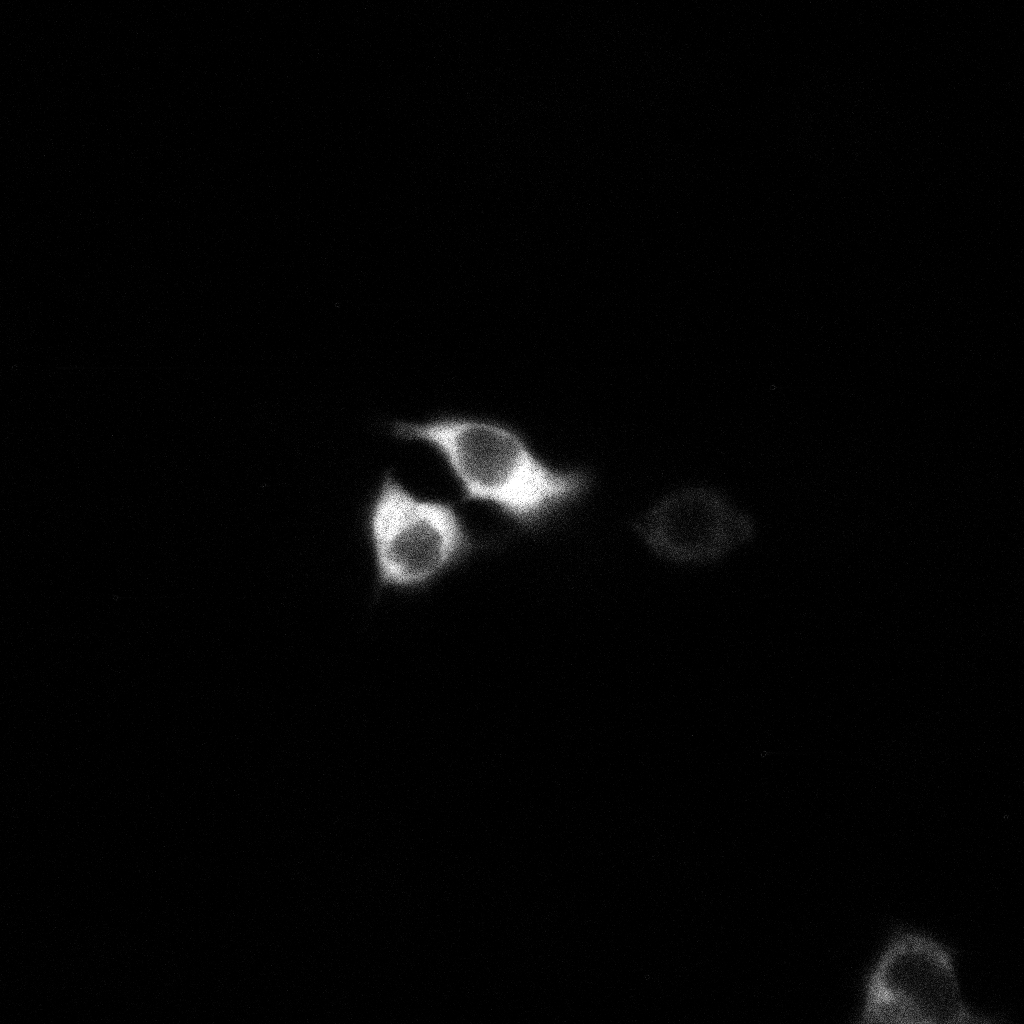

Supplement: Supplementary file 11 — Source Data [file 41467_2024_54263_MOESM11_ESM.zip › Source Data/Fig. 5/Fig. 5 B/Stable probe HEK + 300 probe + 100 staygoldm3p/edited/green_blue/Composite.tif]

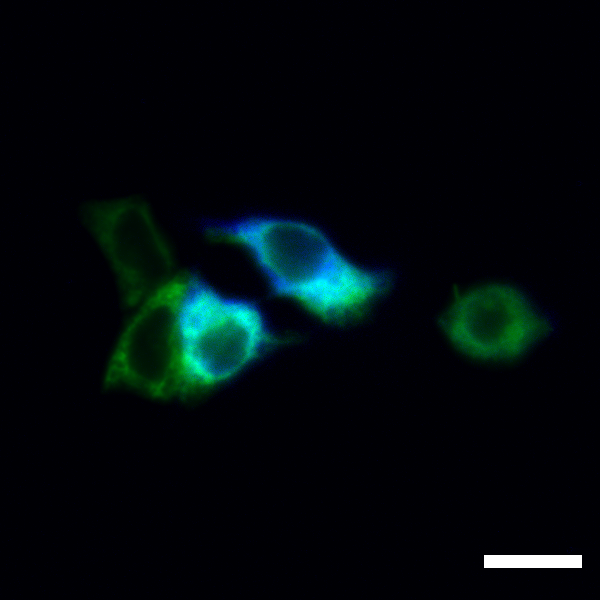

Supplement: Supplementary file 11 — Source Data [file 41467_2024_54263_MOESM11_ESM.zip › Source Data/Fig. 5/Fig. 5 B/Stable probe HEK + 300 probe + 100 staygoldm3p/edited/green_blue/Composite-600-ROIw20um scale bar.tif (RGB).tif]

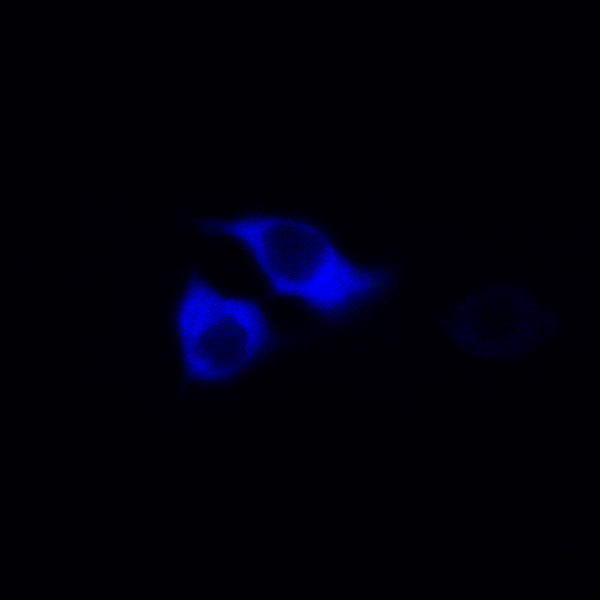

Supplement: Supplementary file 11 — Source Data [file 41467_2024_54263_MOESM11_ESM.zip › Source Data/Fig. 5/Fig. 5 B/Stable probe HEK + 300 probe + 100 staygoldm3p/edited/green_blue/Lumi-RGB.tif (RGB).tif]

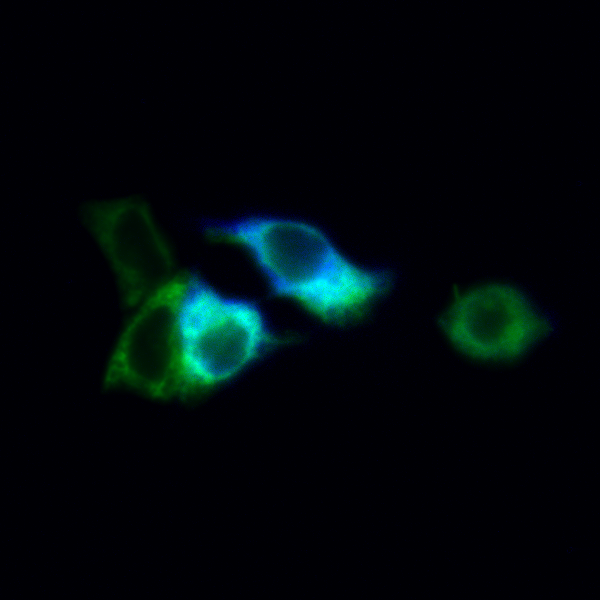

Supplement: Supplementary file 11 — Source Data [file 41467_2024_54263_MOESM11_ESM.zip › Source Data/Fig. 5/Fig. 5 B/Stable probe HEK + 300 probe + 100 staygoldm3p/edited/green_blue/Composite-600-ROI-RGB.tif (RGB).tif]

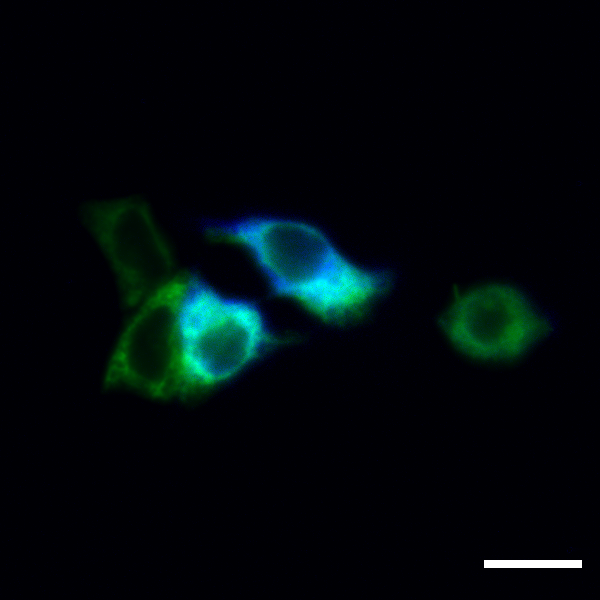

Supplement: Supplementary file 11 — Source Data [file 41467_2024_54263_MOESM11_ESM.zip › Source Data/Fig. 5/Fig. 5 B/Stable probe HEK + 300 probe + 100 staygoldm3p/edited/green_blue/Composite-600-ROIw20um scale bar(RGB).tif]

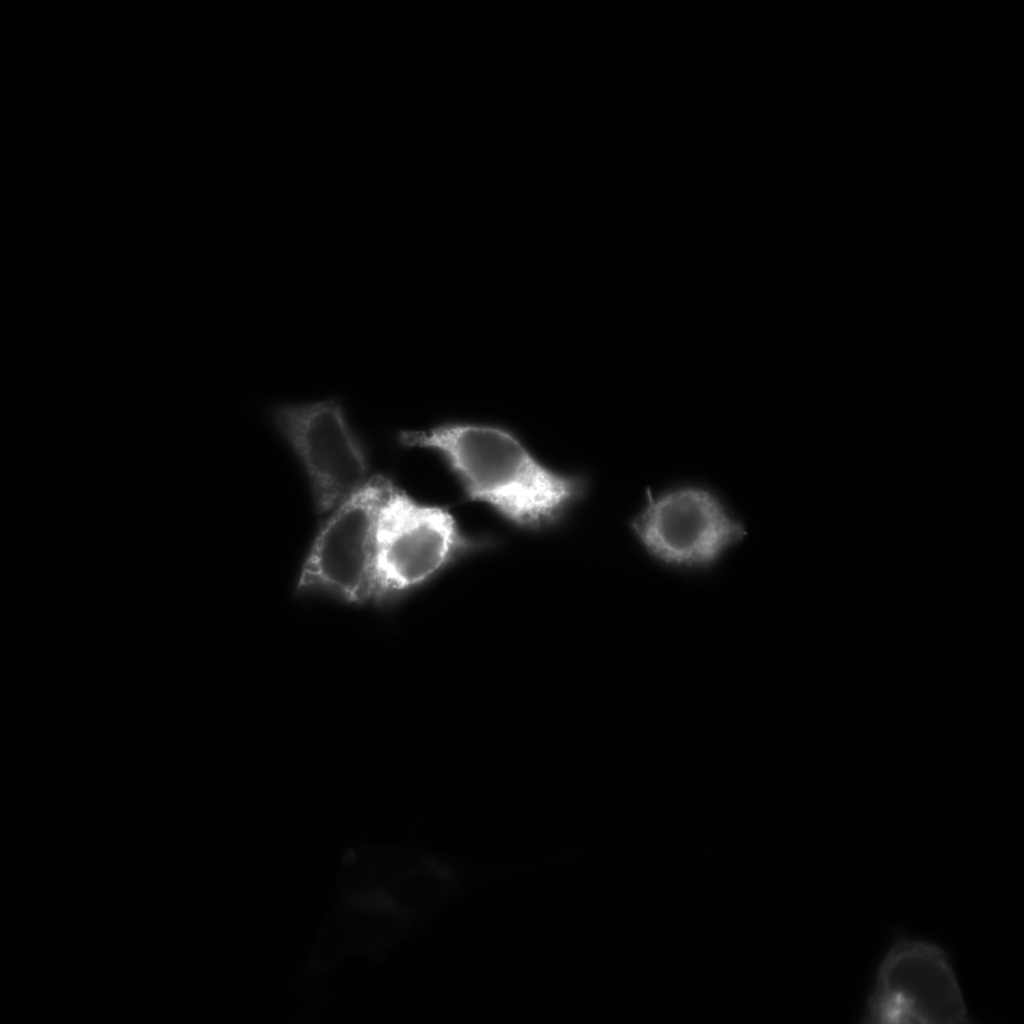

Supplement: Supplementary file 11 — Source Data [file 41467_2024_54263_MOESM11_ESM.zip › Source Data/Fig. 5/Fig. 5 B/Stable probe HEK + 300 probe + 100 staygoldm3p/edited/green_blue/Stack-StayGold.tif]

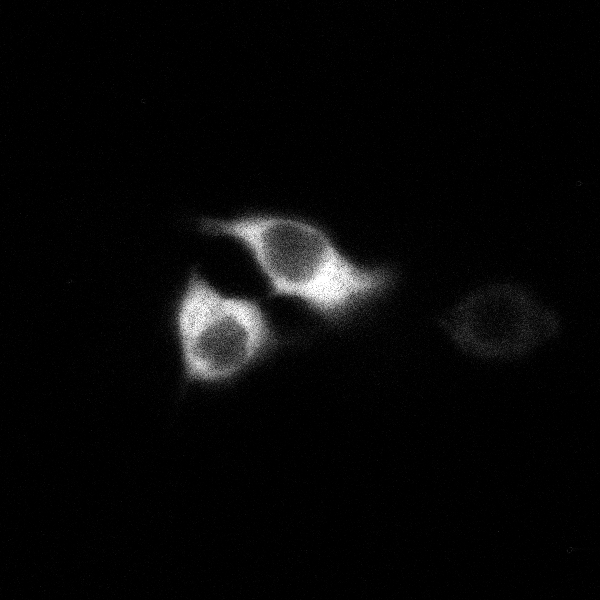

Supplement: Supplementary file 11 — Source Data [file 41467_2024_54263_MOESM11_ESM.zip › Source Data/Fig. 5/Fig. 5 B/Stable probe HEK + 300 probe + 100 staygoldm3p/edited/green_blue/Composite-600-ROIw20um scale bar.tif]

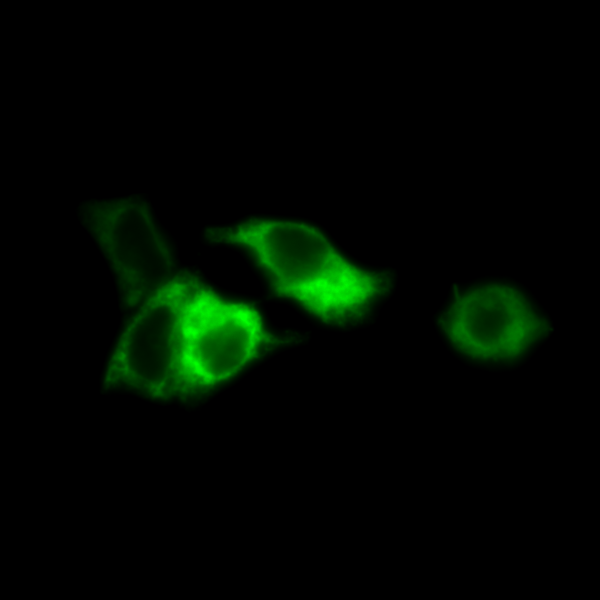

Supplement: Supplementary file 11 — Source Data [file 41467_2024_54263_MOESM11_ESM.zip › Source Data/Fig. 5/Fig. 5 B/Stable probe HEK + 300 probe + 100 staygoldm3p/edited/green_blue/StayGold-RGB.tif (RGB).tif]

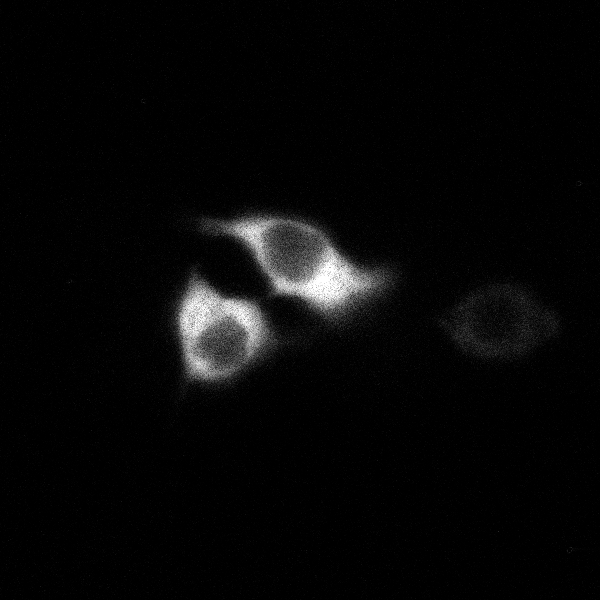

Supplement: Supplementary file 11 — Source Data [file 41467_2024_54263_MOESM11_ESM.zip › Source Data/Fig. 5/Fig. 5 B/Stable probe HEK + 300 probe + 100 staygoldm3p/edited/green_magenta/Composite-600-ROI-mag.tif]

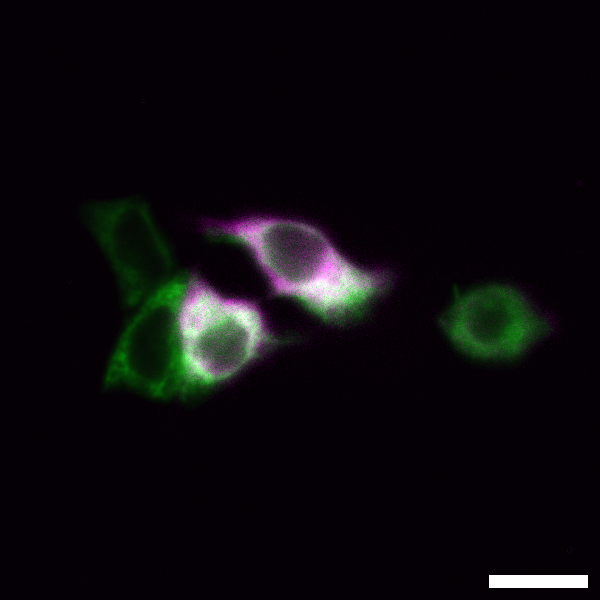

Supplement: Supplementary file 11 — Source Data [file 41467_2024_54263_MOESM11_ESM.zip › Source Data/Fig. 5/Fig. 5 B/Stable probe HEK + 300 probe + 100 staygoldm3p/edited/green_magenta/Composite-600-ROI-mag-20umscalebar(RGB).tif]

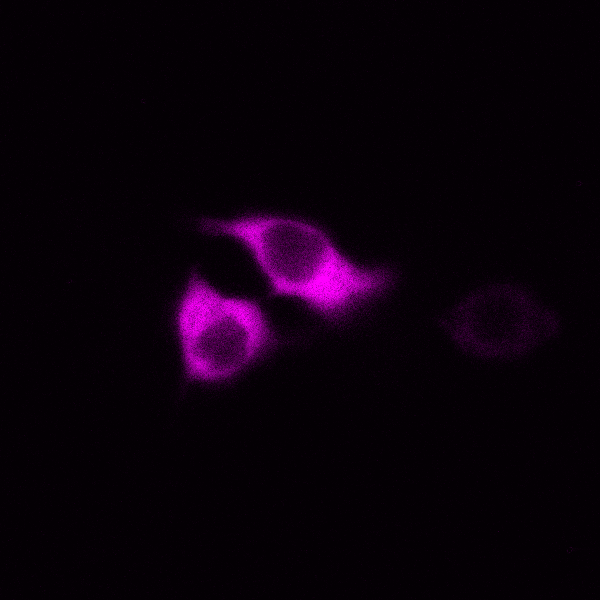

Supplement: Supplementary file 11 — Source Data [file 41467_2024_54263_MOESM11_ESM.zip › Source Data/Fig. 5/Fig. 5 B/Stable probe HEK + 300 probe + 100 staygoldm3p/edited/green_magenta/Composite-600-ROI-mag-lumi(RGB).tif]

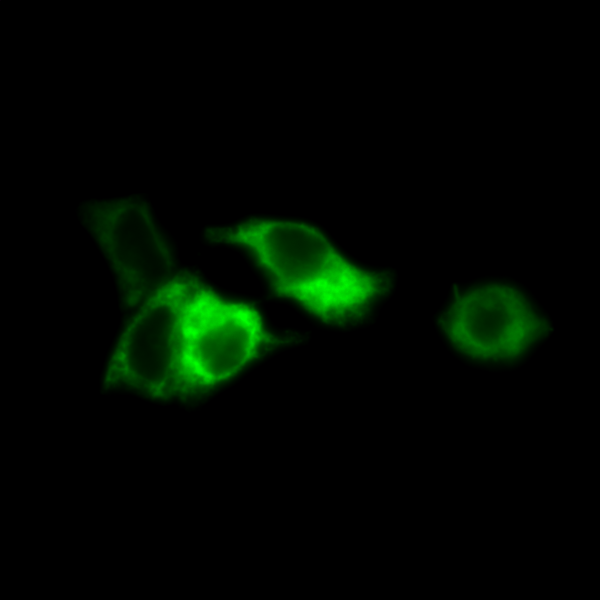

Supplement: Supplementary file 11 — Source Data [file 41467_2024_54263_MOESM11_ESM.zip › Source Data/Fig. 5/Fig. 5 B/Stable probe HEK + 300 probe + 100 staygoldm3p/edited/green_magenta/Composite-600-ROI-StayGold (RGB).tif]

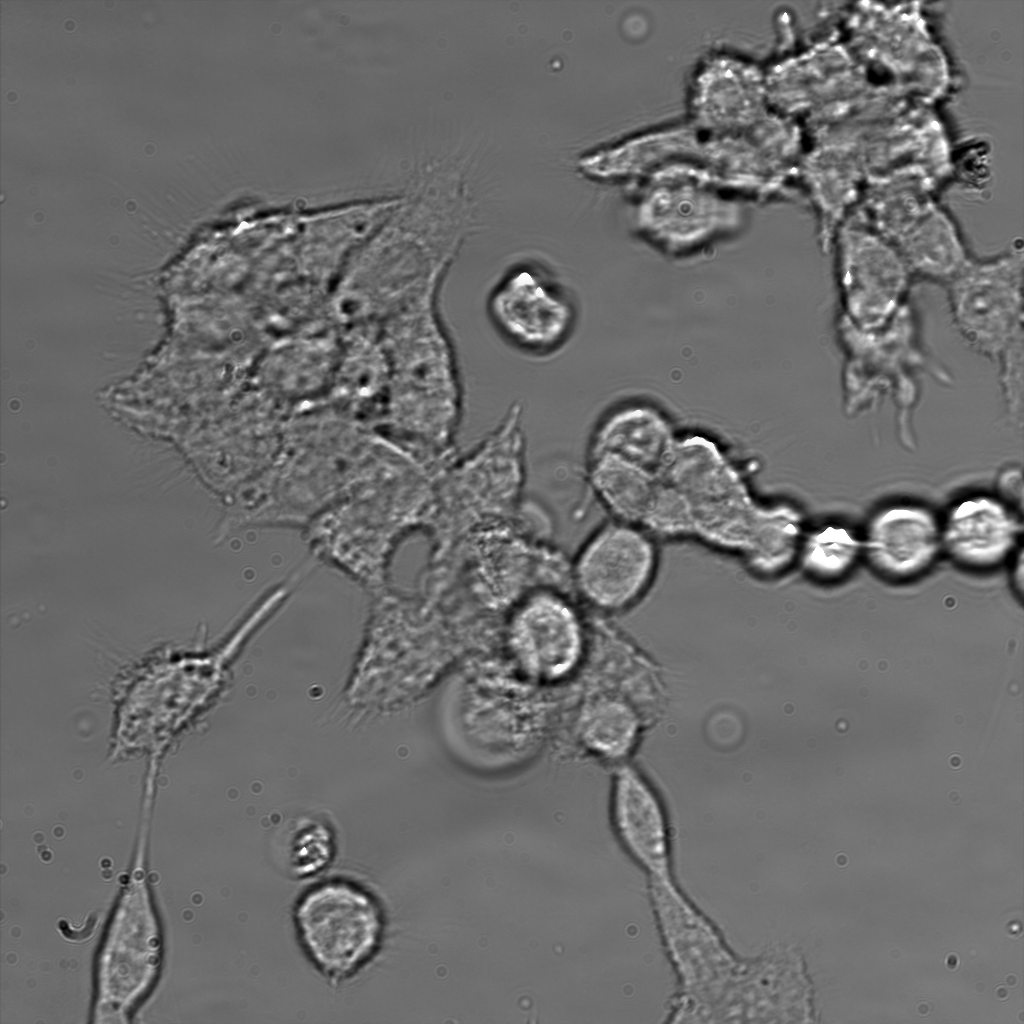

Supplement: Supplementary file 11 — Source Data [file 41467_2024_54263_MOESM11_ESM.zip › Source Data/Fig. 5/Fig. 5 B/Stable probe HEK + 300 probe + 100 staygoldm3p/BF/3rd/40X-brightfield_1_X1.tif]

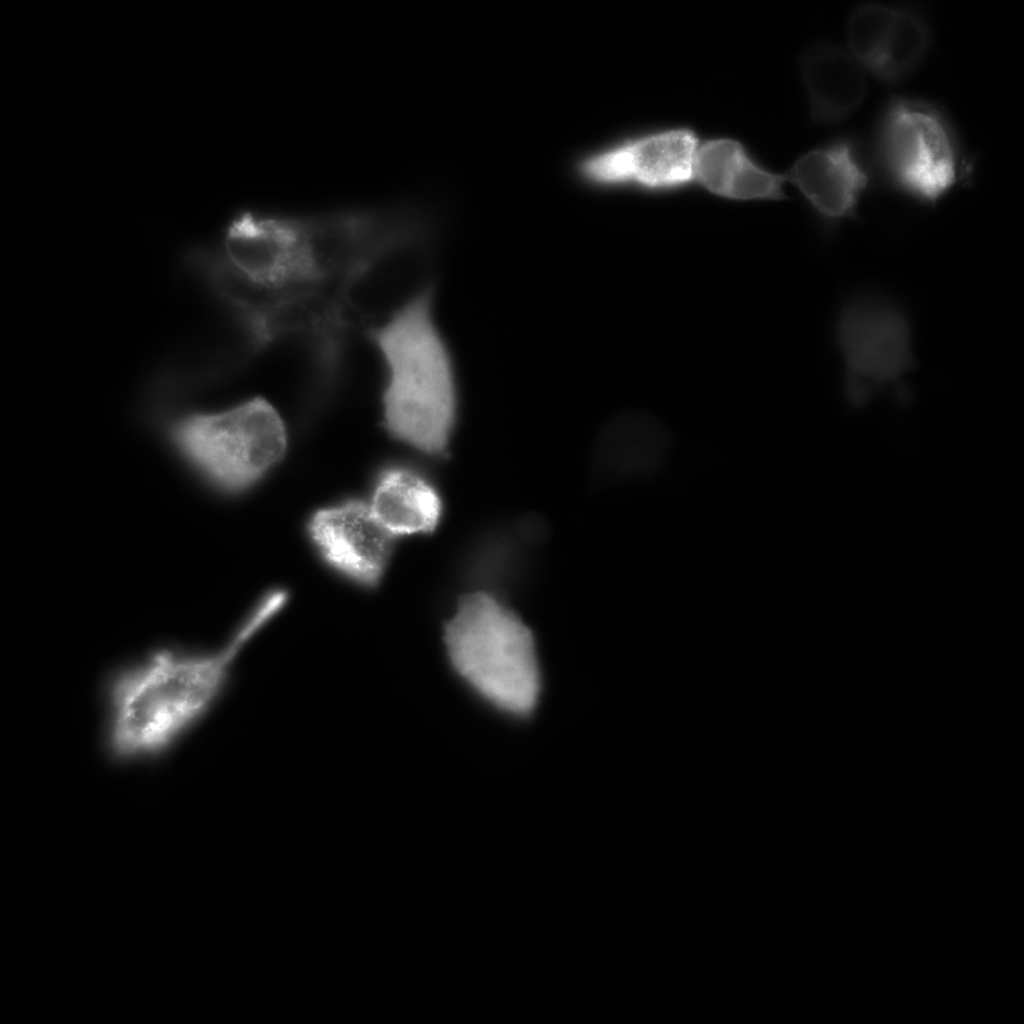

Supplement: Supplementary file 11 — Source Data [file 41467_2024_54263_MOESM11_ESM.zip › Source Data/Fig. 5/Fig. 5 B/Stable probe HEK + 300 probe + 100 staygoldm3p/fluorescence/3rd/StayGoldm3p100ng_7_X1.tif]

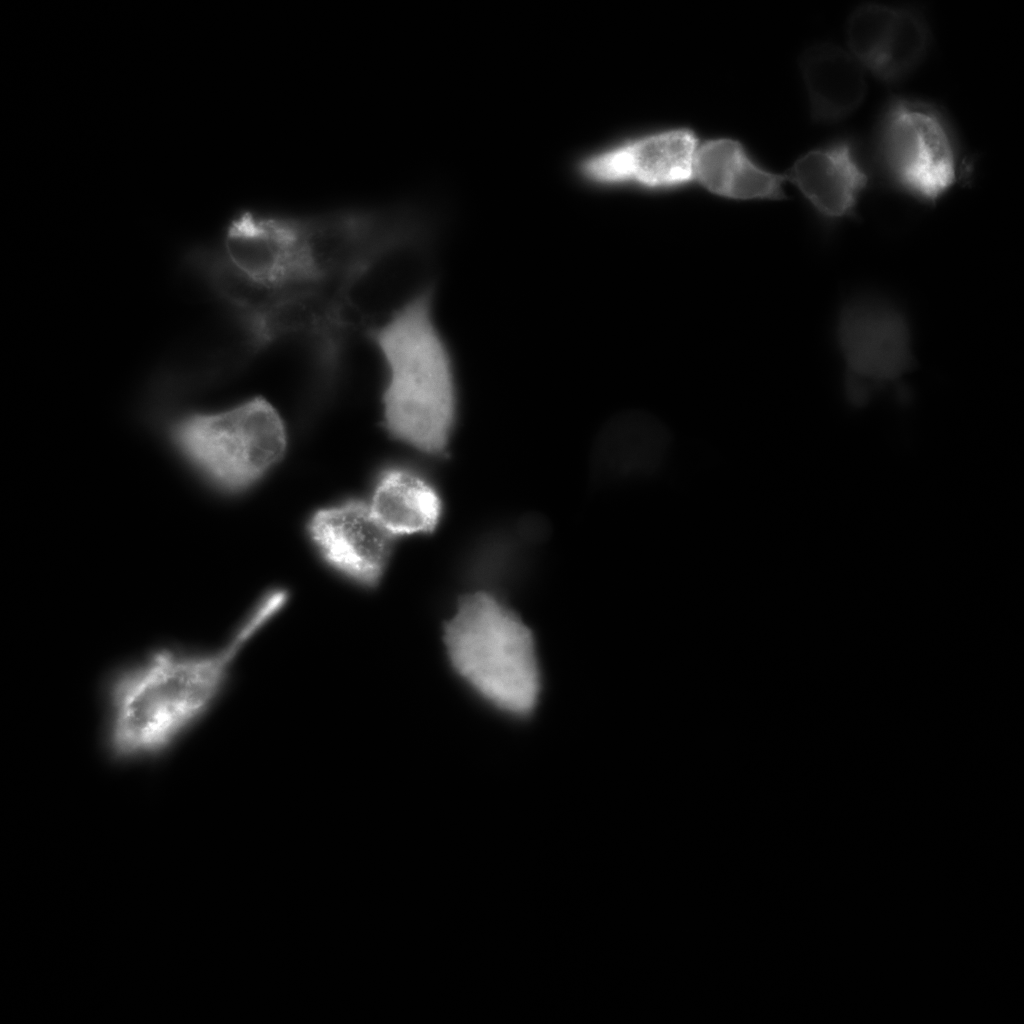

Supplement: Supplementary file 11 — Source Data [file 41467_2024_54263_MOESM11_ESM.zip › Source Data/Fig. 5/Fig. 5 B/Stable probe HEK + 300 probe + 100 staygoldm3p/fluorescence/3rd/StayGoldm3p100ng_5_X1.tif]

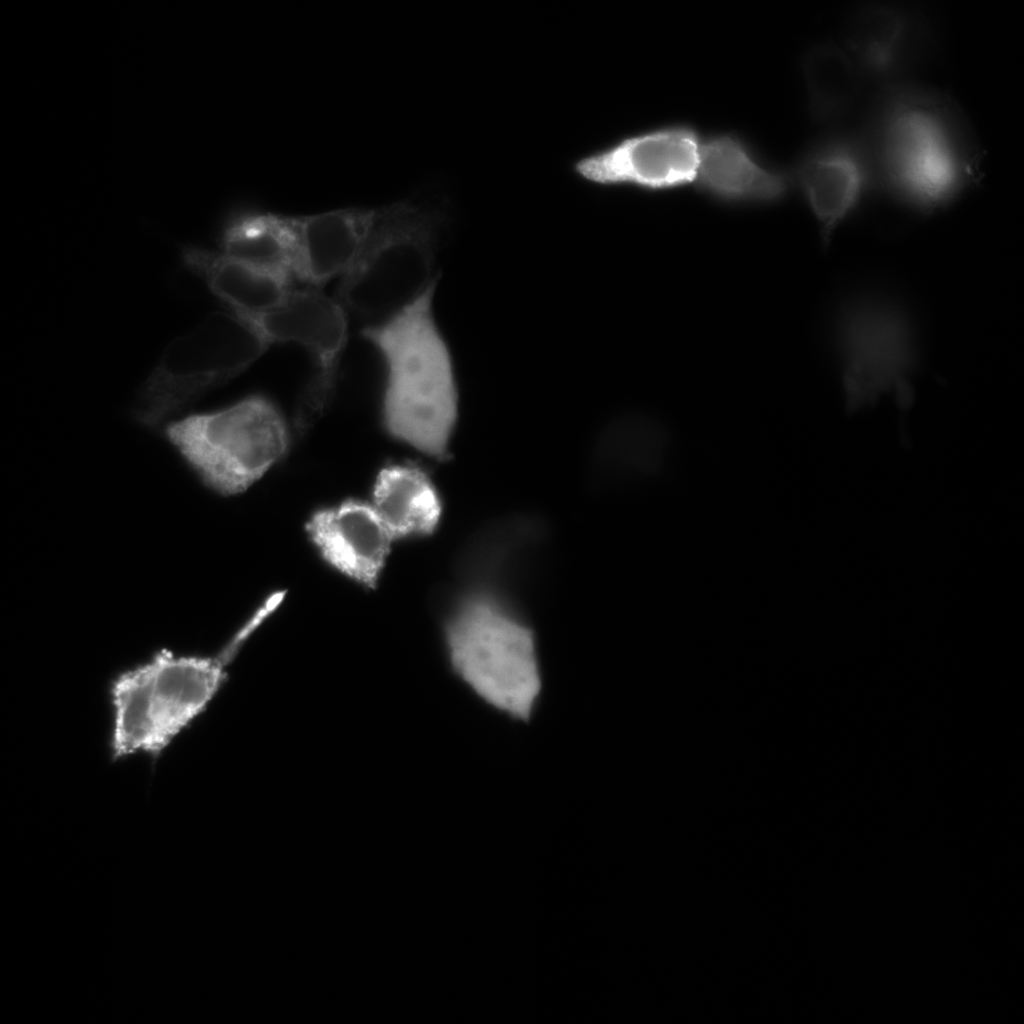

Supplement: Supplementary file 11 — Source Data [file 41467_2024_54263_MOESM11_ESM.zip › Source Data/Fig. 5/Fig. 5 B/Stable probe HEK + 300 probe + 100 staygoldm3p/fluorescence/3rd/StayGoldm3p100ng_9_X1.tif]

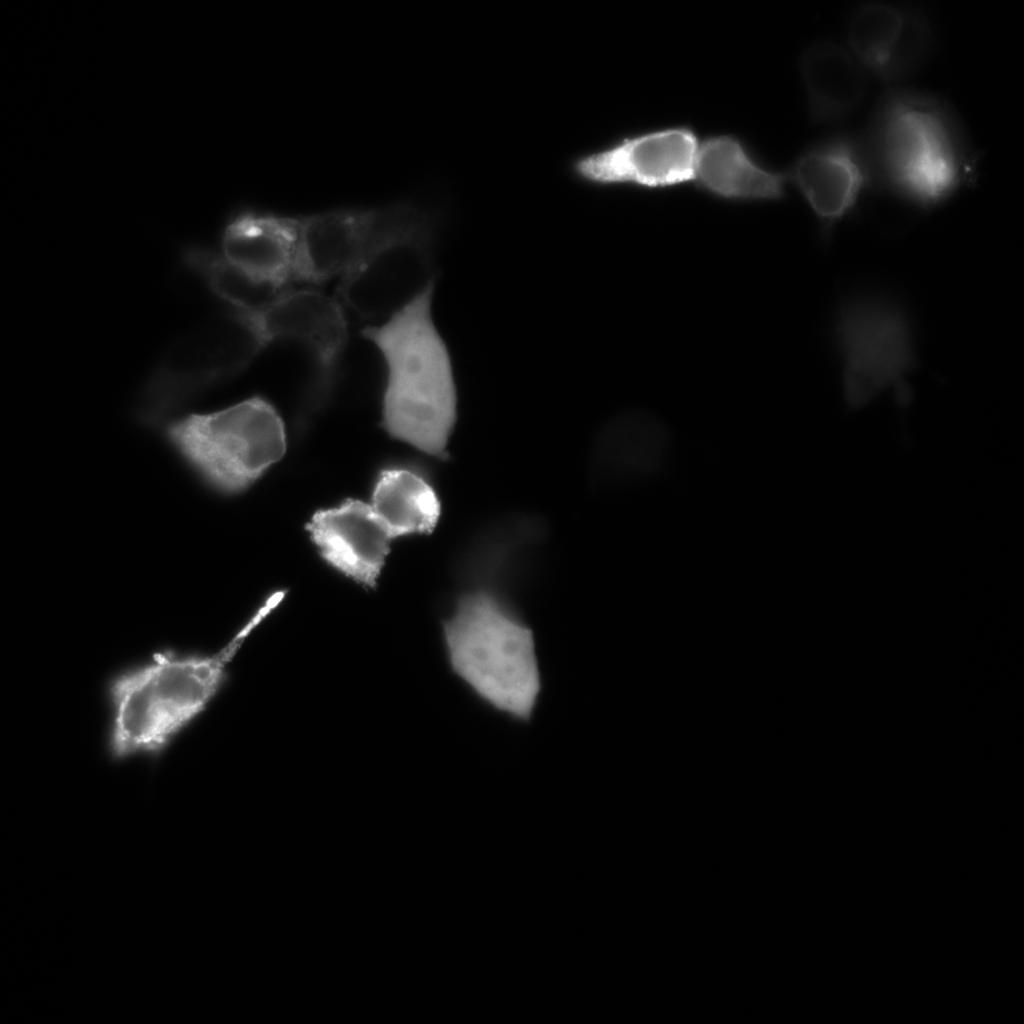

Supplement: Supplementary file 11 — Source Data [file 41467_2024_54263_MOESM11_ESM.zip › Source Data/Fig. 5/Fig. 5 B/Stable probe HEK + 300 probe + 100 staygoldm3p/fluorescence/3rd/StayGoldm3p100ng_1_X1.tif]

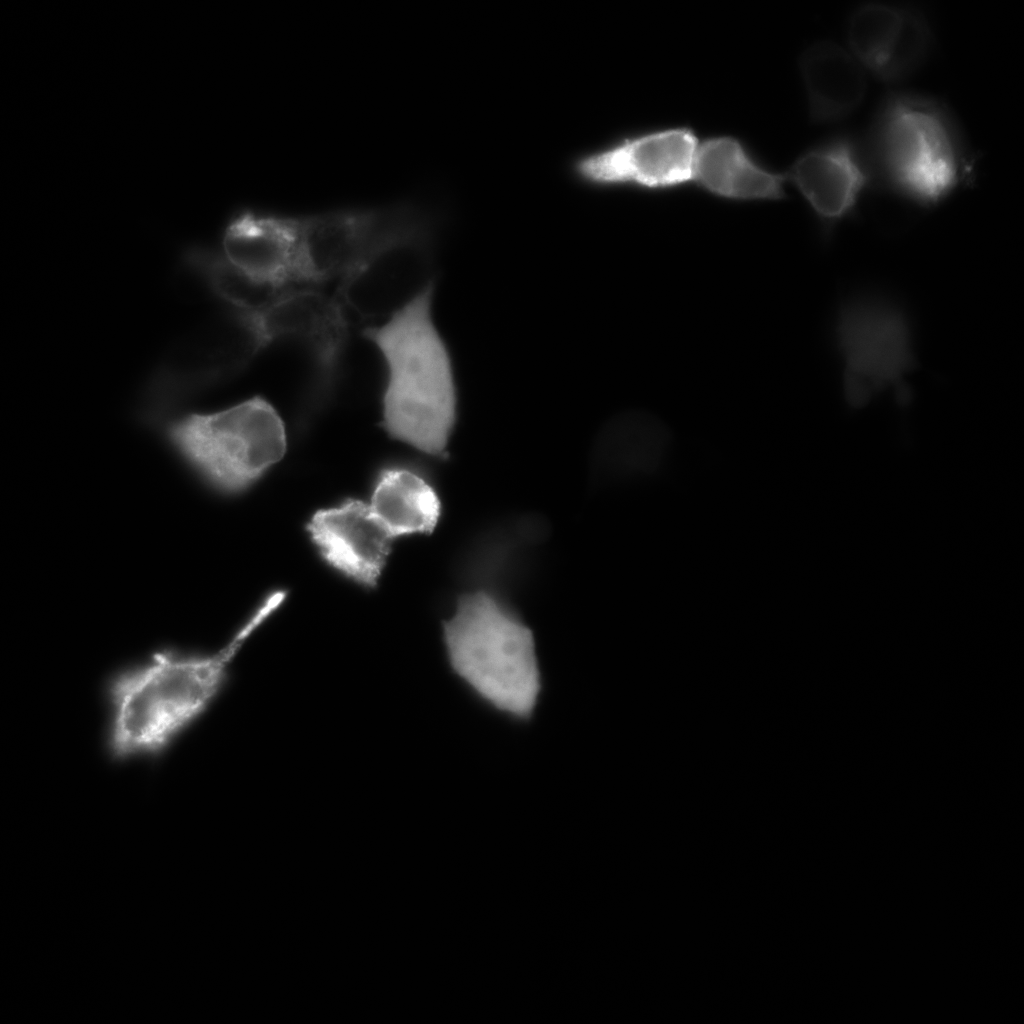

Supplement: Supplementary file 11 — Source Data [file 41467_2024_54263_MOESM11_ESM.zip › Source Data/Fig. 5/Fig. 5 B/Stable probe HEK + 300 probe + 100 staygoldm3p/fluorescence/3rd/StayGoldm3p100ng_3_X1.tif]

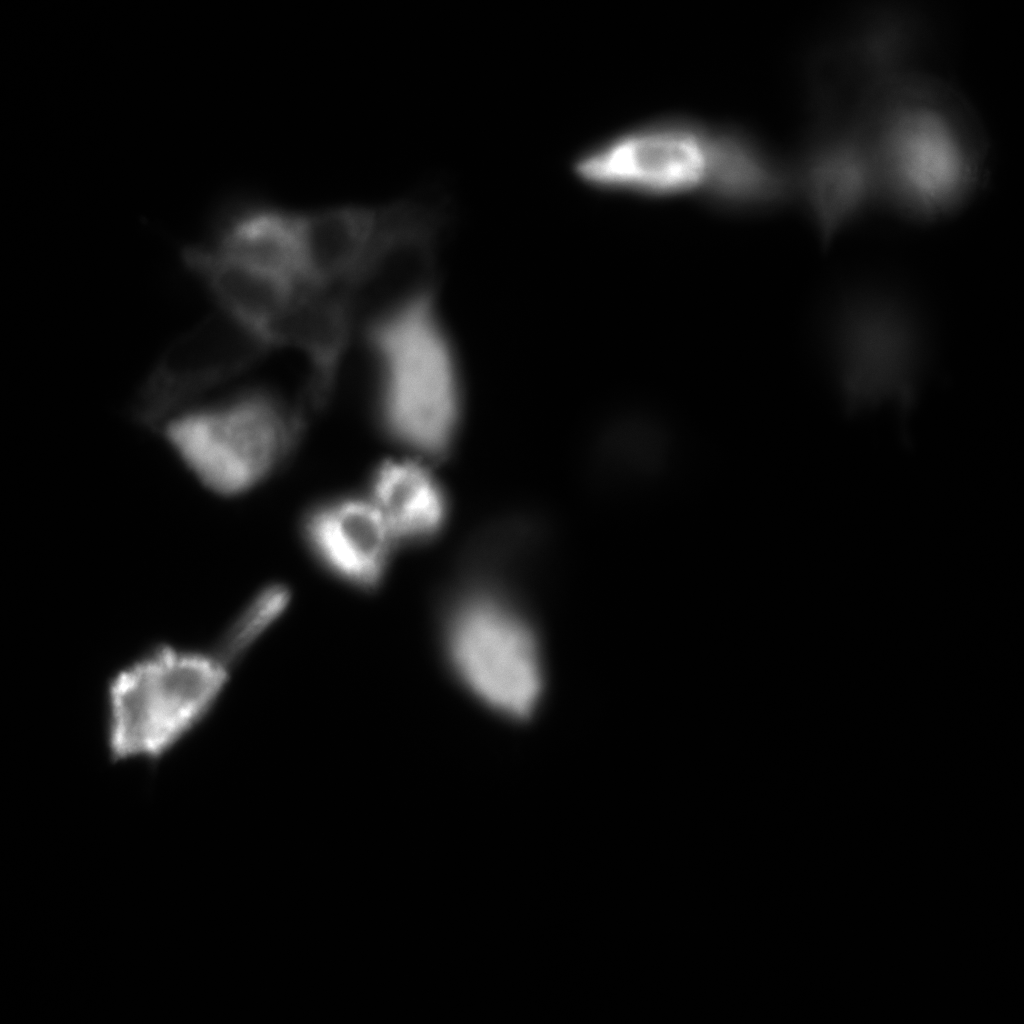

Supplement: Supplementary file 11 — Source Data [file 41467_2024_54263_MOESM11_ESM.zip › Source Data/Fig. 5/Fig. 5 B/Stable probe HEK + 300 probe + 100 staygoldm3p/fluorescence/3rd/StayGoldm3p100ng_11_X1.tif]

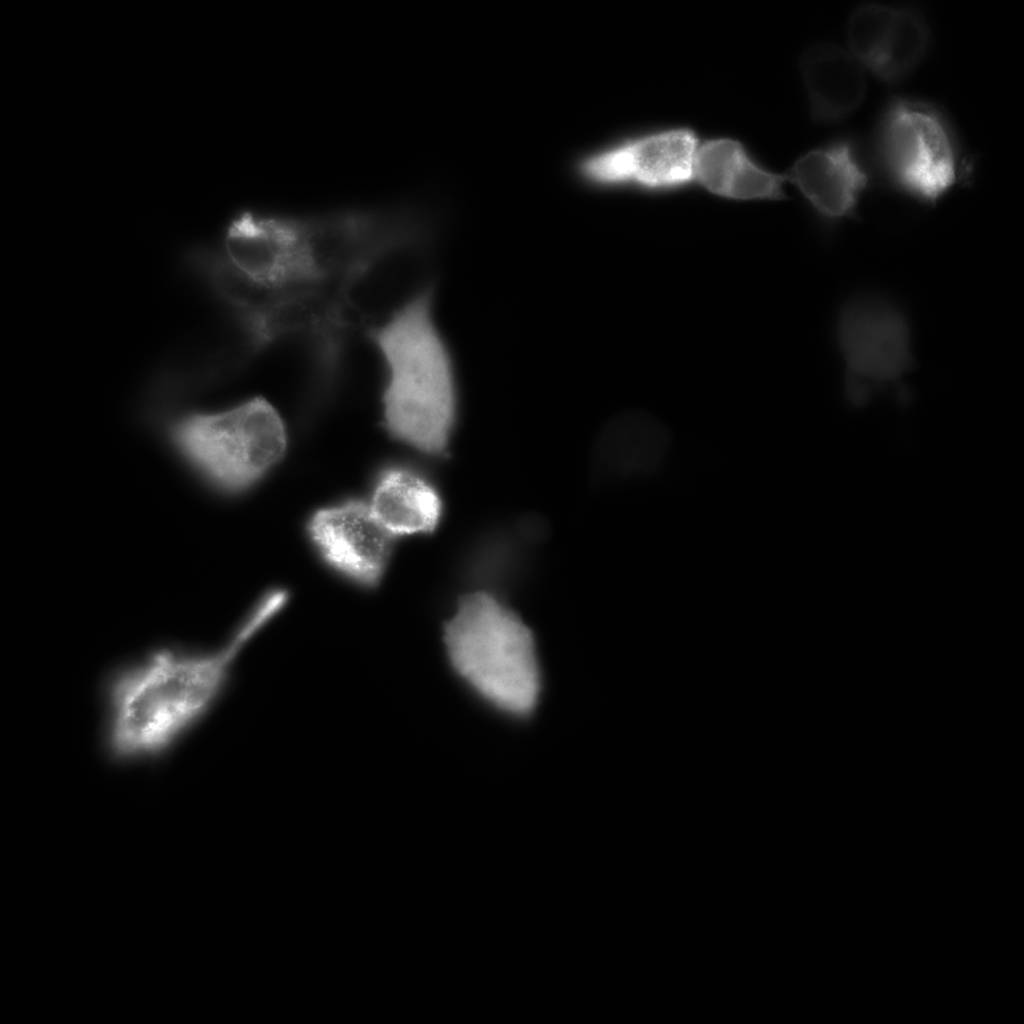

Supplement: Supplementary file 11 — Source Data [file 41467_2024_54263_MOESM11_ESM.zip › Source Data/Fig. 5/Fig. 5 B/Stable probe HEK + 300 probe + 100 staygoldm3p/fluorescence/3rd/StayGoldm3p100ng_6_X1.tif]

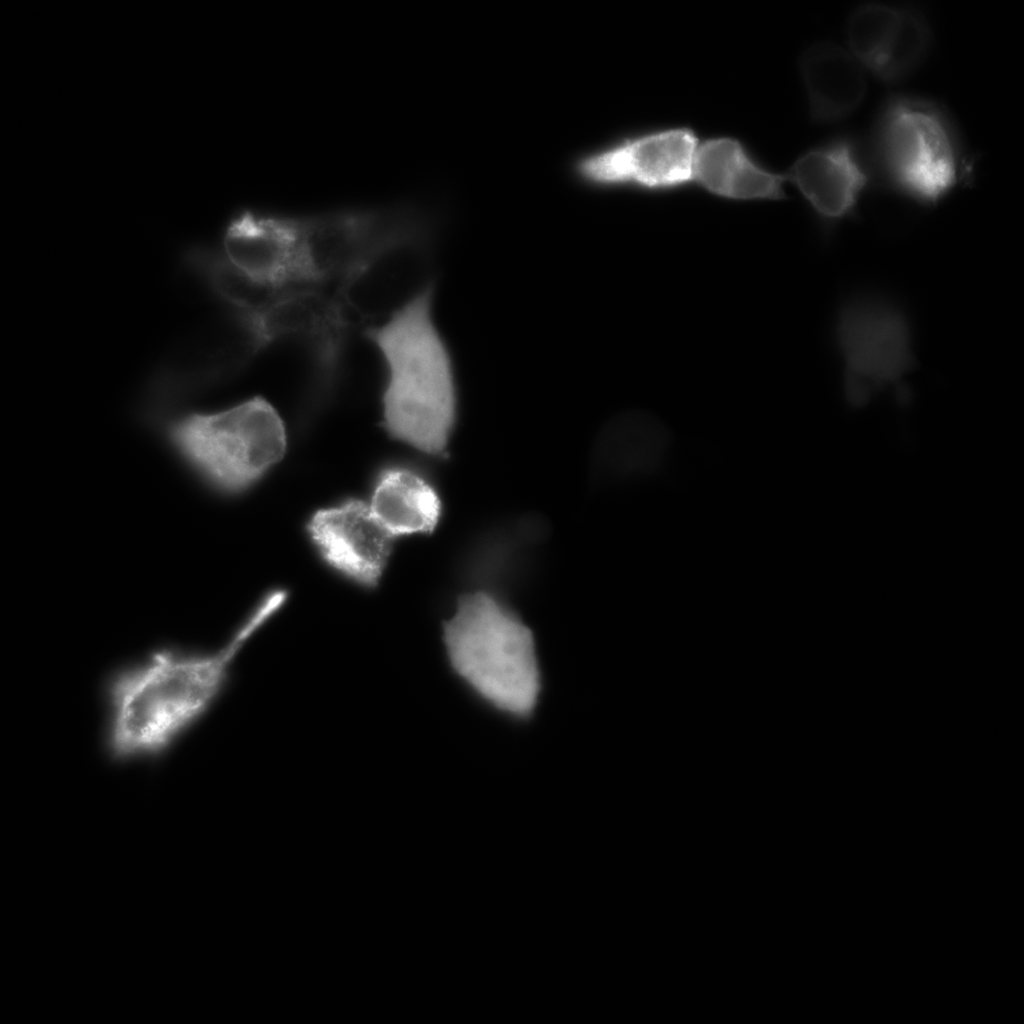

Supplement: Supplementary file 11 — Source Data [file 41467_2024_54263_MOESM11_ESM.zip › Source Data/Fig. 5/Fig. 5 B/Stable probe HEK + 300 probe + 100 staygoldm3p/fluorescence/3rd/StayGoldm3p100ng_4_X1.tif]

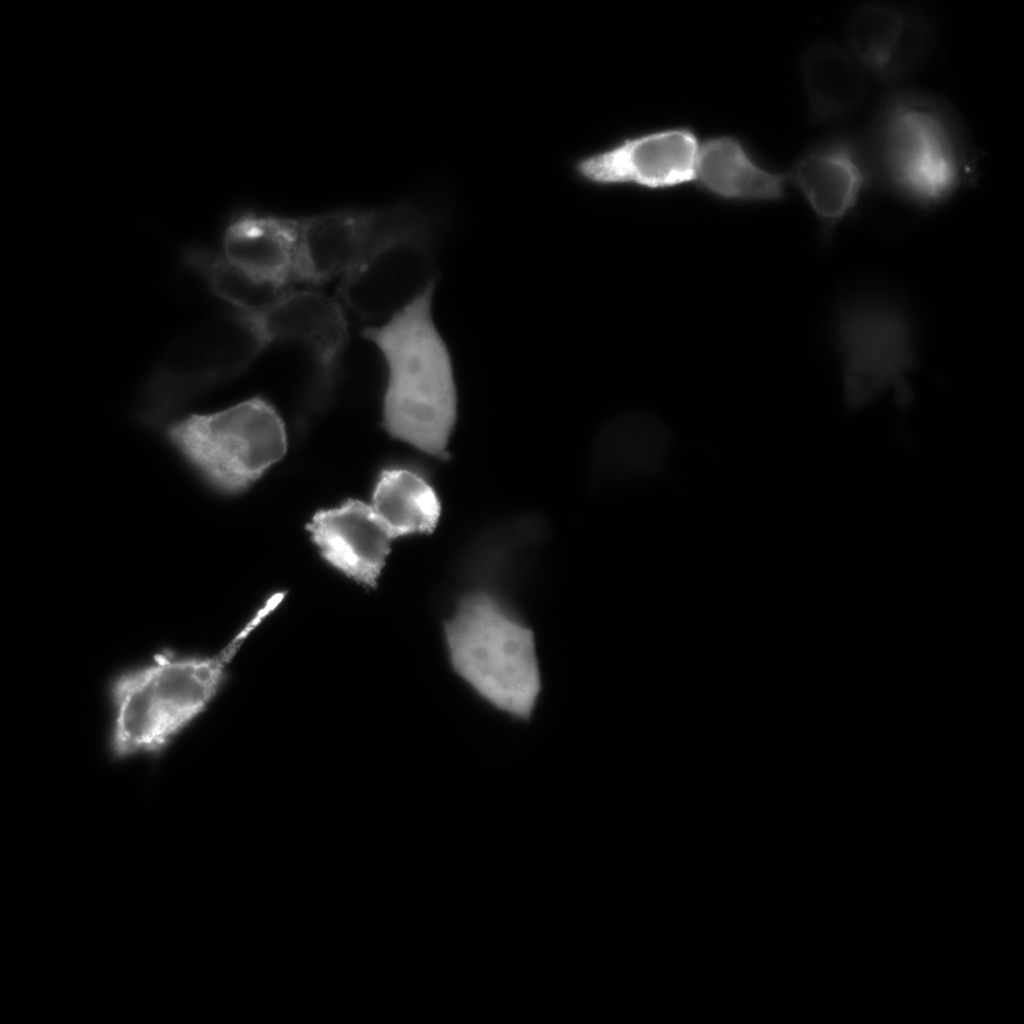

Supplement: Supplementary file 11 — Source Data [file 41467_2024_54263_MOESM11_ESM.zip › Source Data/Fig. 5/Fig. 5 B/Stable probe HEK + 300 probe + 100 staygoldm3p/fluorescence/3rd/StayGoldm3p100ng_8_X1.tif]

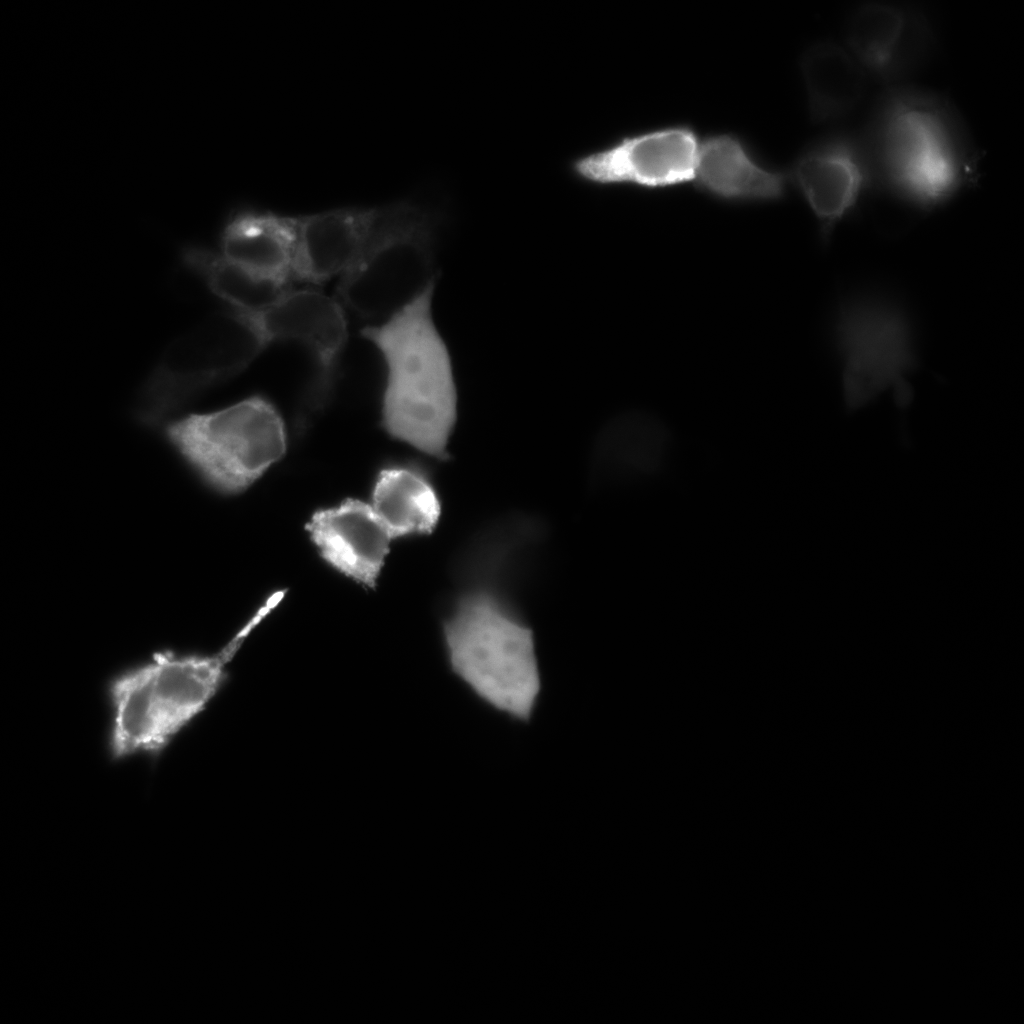

Supplement: Supplementary file 11 — Source Data [file 41467_2024_54263_MOESM11_ESM.zip › Source Data/Fig. 5/Fig. 5 B/Stable probe HEK + 300 probe + 100 staygoldm3p/fluorescence/3rd/StayGoldm3p100ng_0_X1.tif]

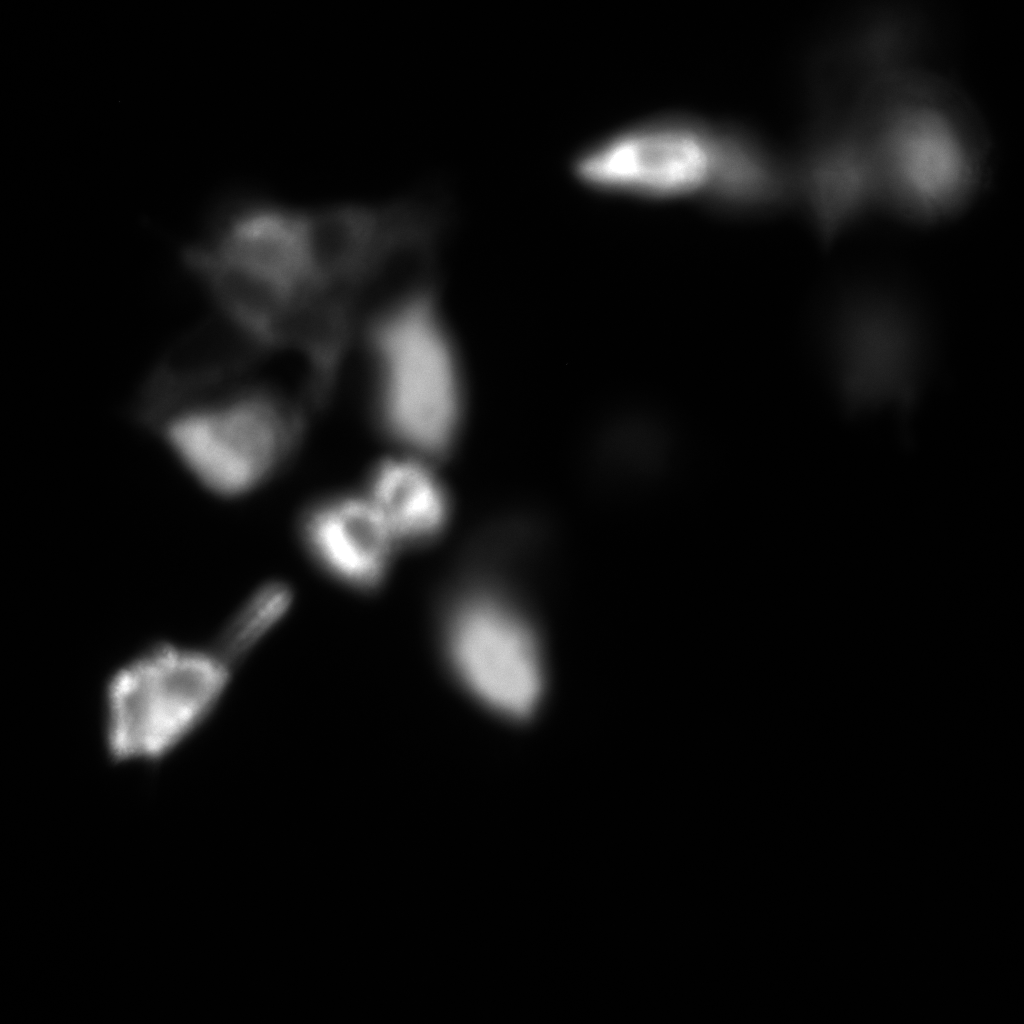

Supplement: Supplementary file 11 — Source Data [file 41467_2024_54263_MOESM11_ESM.zip › Source Data/Fig. 5/Fig. 5 B/Stable probe HEK + 300 probe + 100 staygoldm3p/fluorescence/3rd/StayGoldm3p100ng_12_X1.tif]

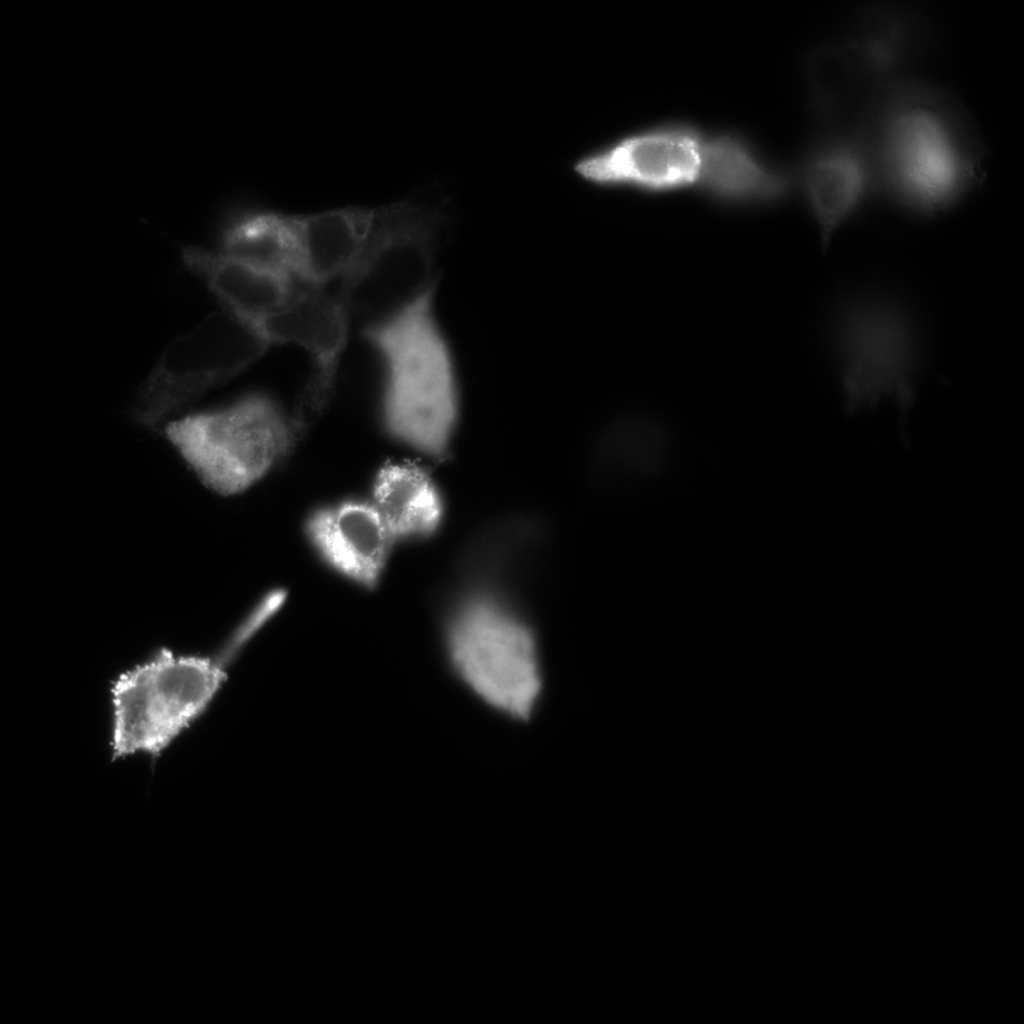

Supplement: Supplementary file 11 — Source Data [file 41467_2024_54263_MOESM11_ESM.zip › Source Data/Fig. 5/Fig. 5 B/Stable probe HEK + 300 probe + 100 staygoldm3p/fluorescence/3rd/StayGoldm3p100ng_10_X1.tif]

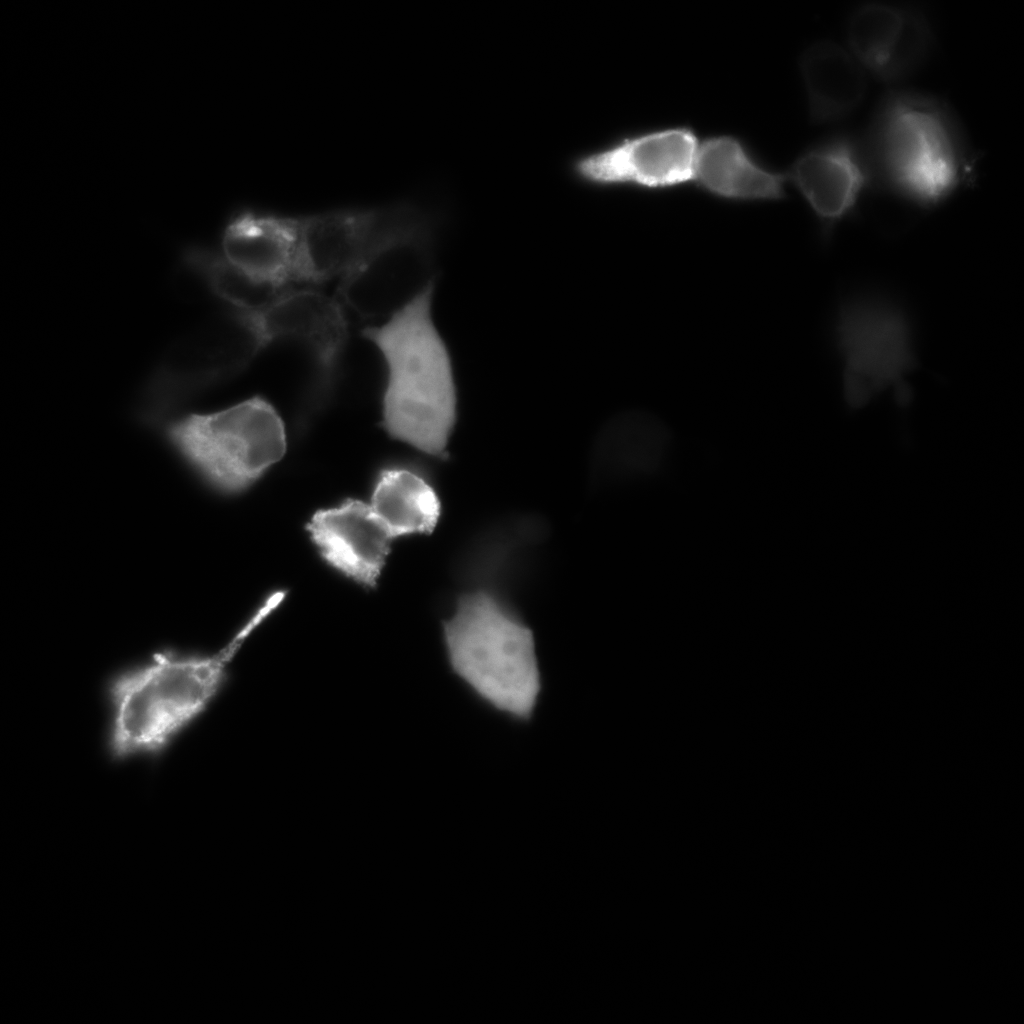

Supplement: Supplementary file 11 — Source Data [file 41467_2024_54263_MOESM11_ESM.zip › Source Data/Fig. 5/Fig. 5 B/Stable probe HEK + 300 probe + 100 staygoldm3p/fluorescence/3rd/StayGoldm3p100ng_2_X1.tif]

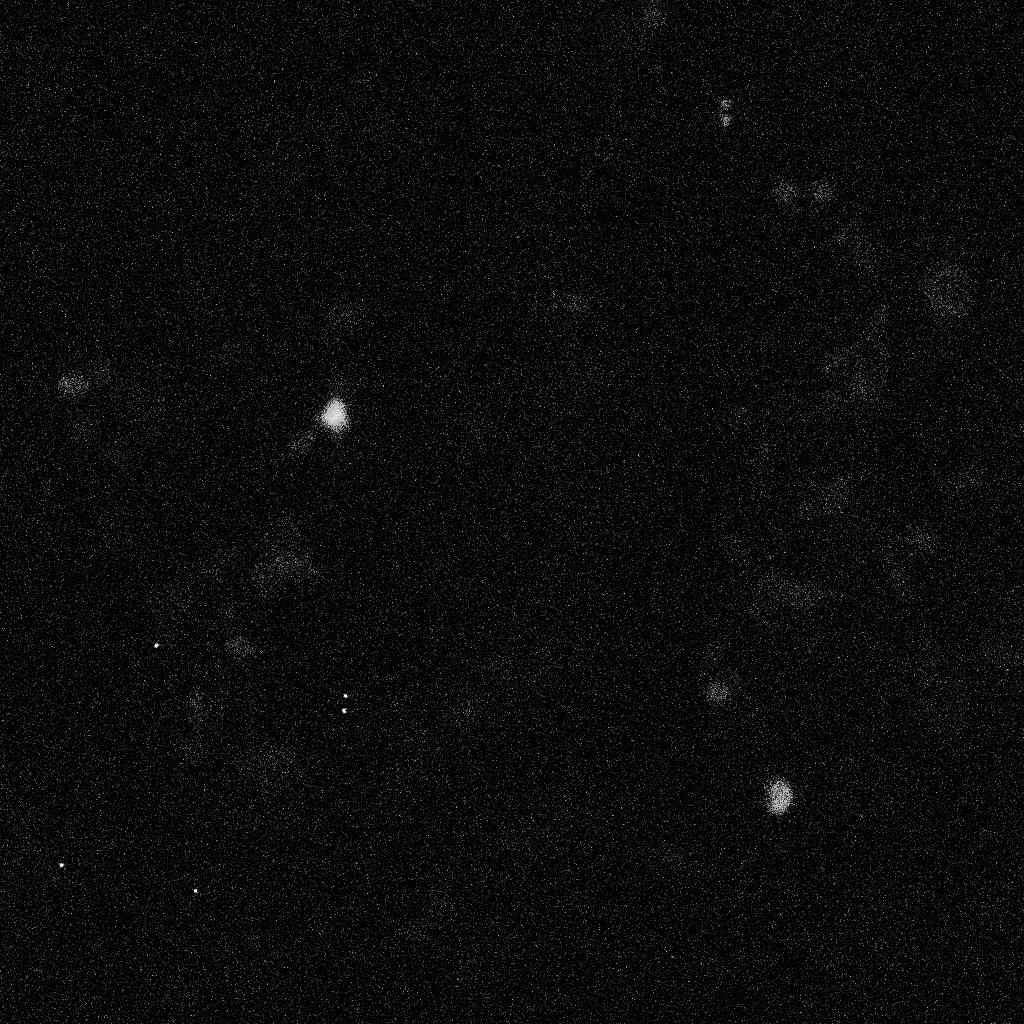

Supplement: Supplementary file 11 — Source Data [file 41467_2024_54263_MOESM11_ESM.zip › Source Data/Fig. 5/Fig. 5 B/Stable probe HEK + 300 probe + 100staygoldcontrol/lumi/20x/Lumi20x180s_1_X1.tif]

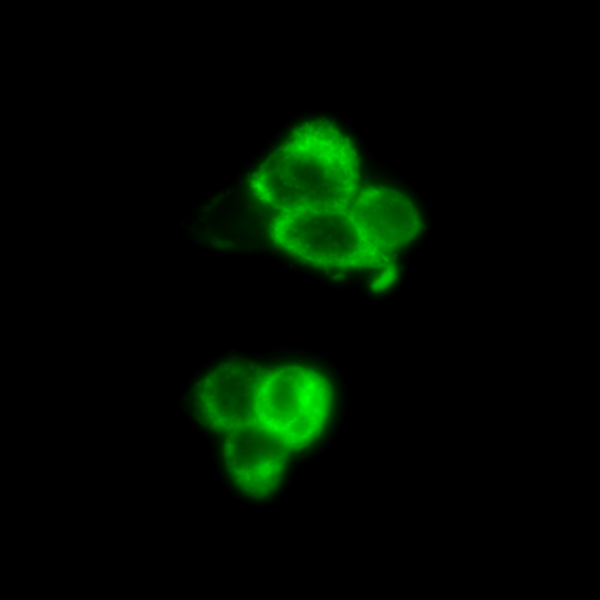

Supplement: Supplementary file 11 — Source Data [file 41467_2024_54263_MOESM11_ESM.zip › Source Data/Fig. 5/Fig. 5 B/Stable probe HEK + 300 probe + 100staygoldcontrol/edited/ROI 600/Composite-ROI-600-StayGold.tif (RGB).tif]

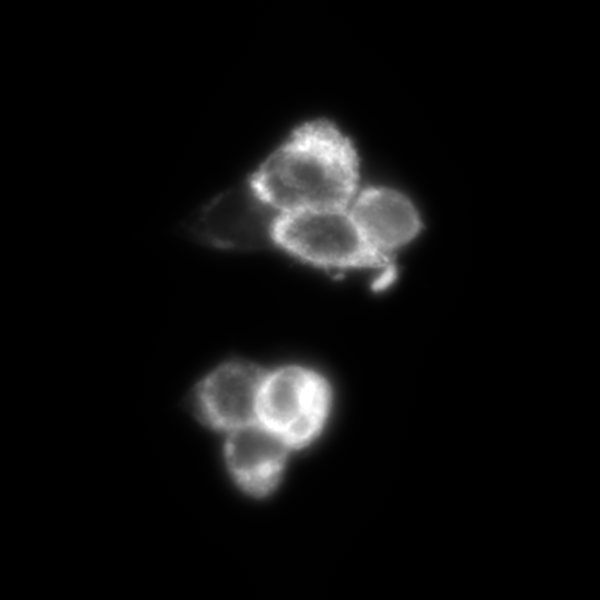

Supplement: Supplementary file 11 — Source Data [file 41467_2024_54263_MOESM11_ESM.zip › Source Data/Fig. 5/Fig. 5 B/Stable probe HEK + 300 probe + 100staygoldcontrol/edited/ROI 600/Composite-ROI-600.tif]

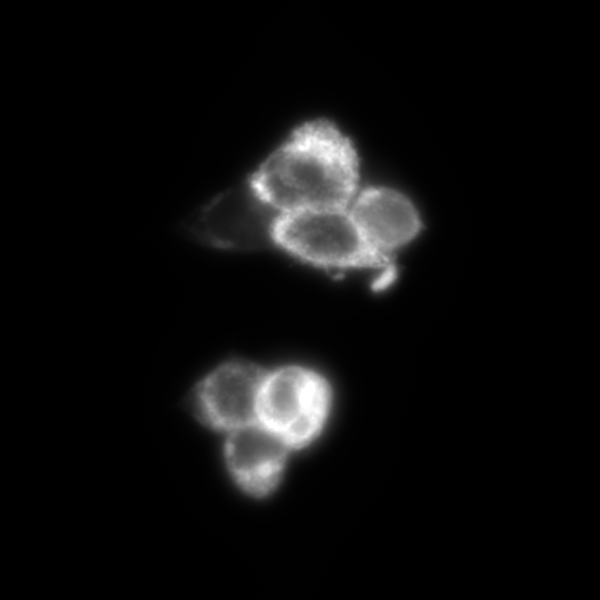

Supplement: Supplementary file 11 — Source Data [file 41467_2024_54263_MOESM11_ESM.zip › Source Data/Fig. 5/Fig. 5 B/Stable probe HEK + 300 probe + 100staygoldcontrol/edited/ROI 600/Composite-ROI-600-20umscalebar.tif]

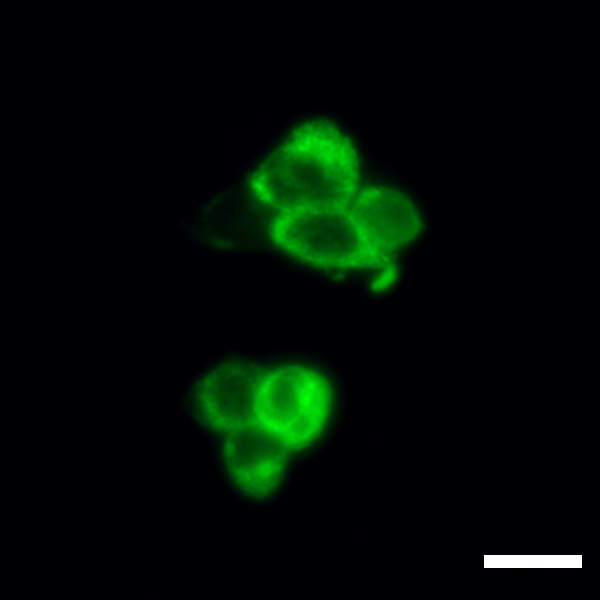

Supplement: Supplementary file 11 — Source Data [file 41467_2024_54263_MOESM11_ESM.zip › Source Data/Fig. 5/Fig. 5 B/Stable probe HEK + 300 probe + 100staygoldcontrol/edited/ROI 600/Composite-ROI-600-20umscalebar.tif (RGB).tif]

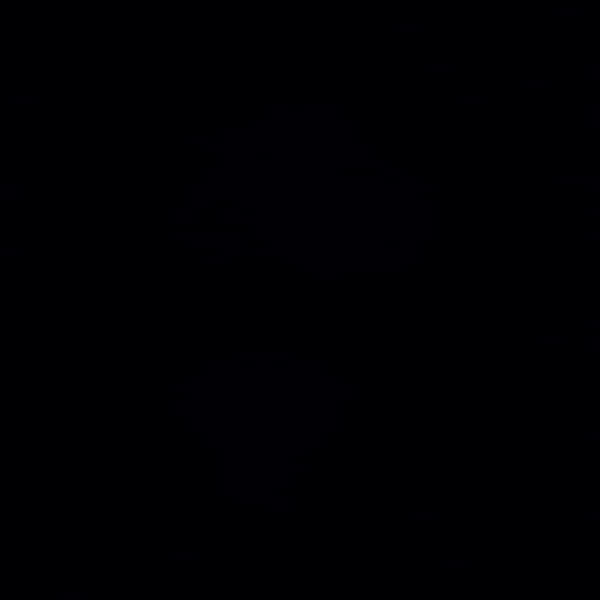

Supplement: Supplementary file 11 — Source Data [file 41467_2024_54263_MOESM11_ESM.zip › Source Data/Fig. 5/Fig. 5 B/Stable probe HEK + 300 probe + 100staygoldcontrol/edited/ROI 600/Composite-ROI-600-Lumi.tif (RGB).tif]

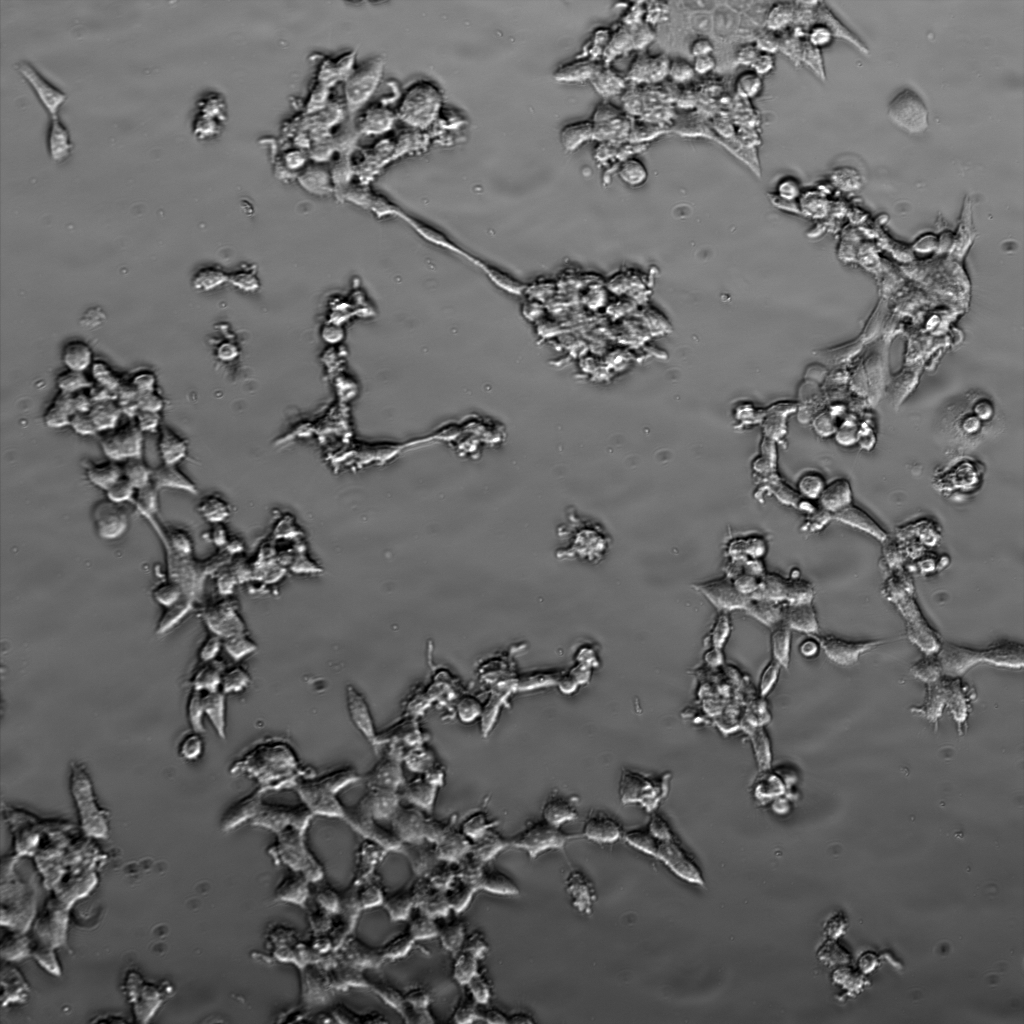

Supplement: Supplementary file 11 — Source Data [file 41467_2024_54263_MOESM11_ESM.zip › Source Data/Fig. 5/Fig. 5 B/Stable probe HEK + 300 probe + 100staygoldcontrol/BF/20x/20X-brightfield_2_X1.tif]

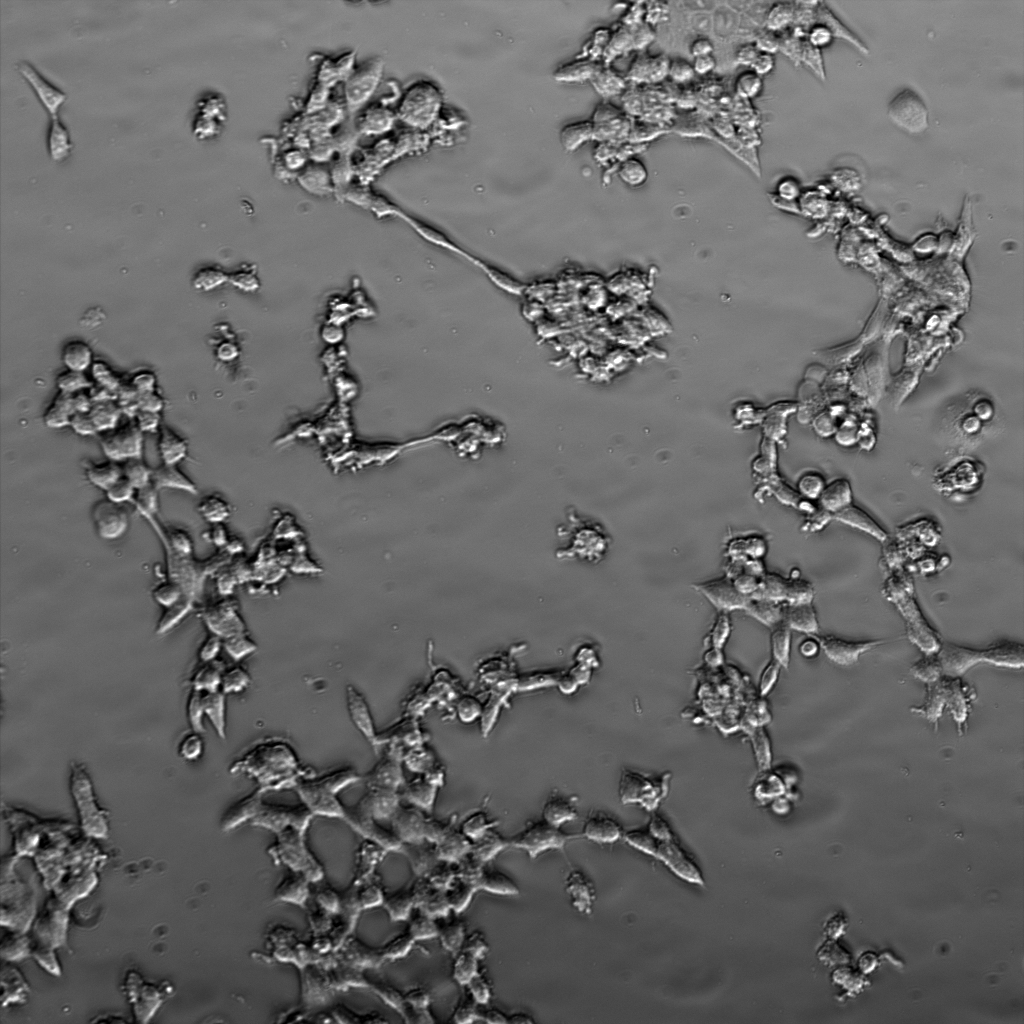

Supplement: Supplementary file 11 — Source Data [file 41467_2024_54263_MOESM11_ESM.zip › Source Data/Fig. 5/Fig. 5 B/Stable probe HEK + 300 probe + 100staygoldcontrol/BF/20x/20X-brightfield_1_X1.tif]

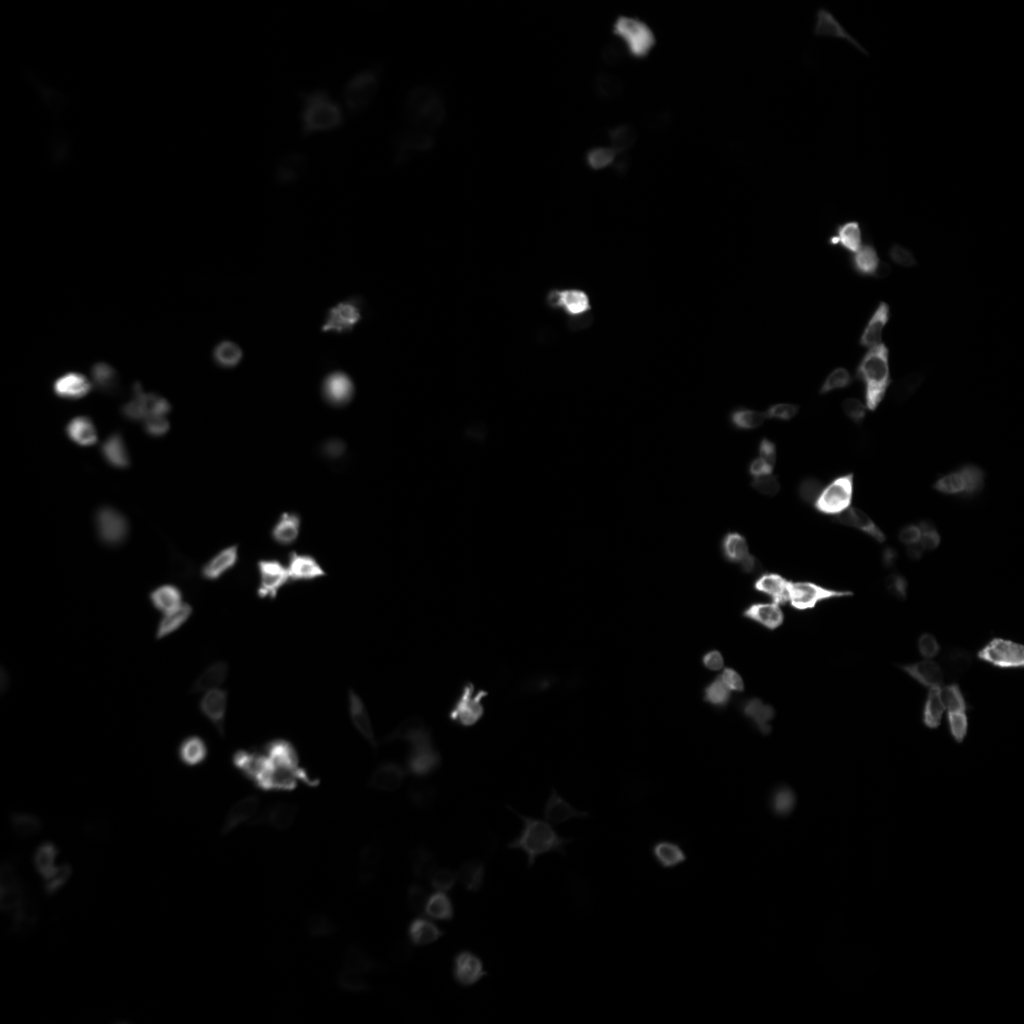

Supplement: Supplementary file 11 — Source Data [file 41467_2024_54263_MOESM11_ESM.zip › Source Data/Fig. 5/Fig. 5 B/Stable probe HEK + 300 probe + 100staygoldcontrol/fluorescence/20x/StayGold_1_X1.tif]

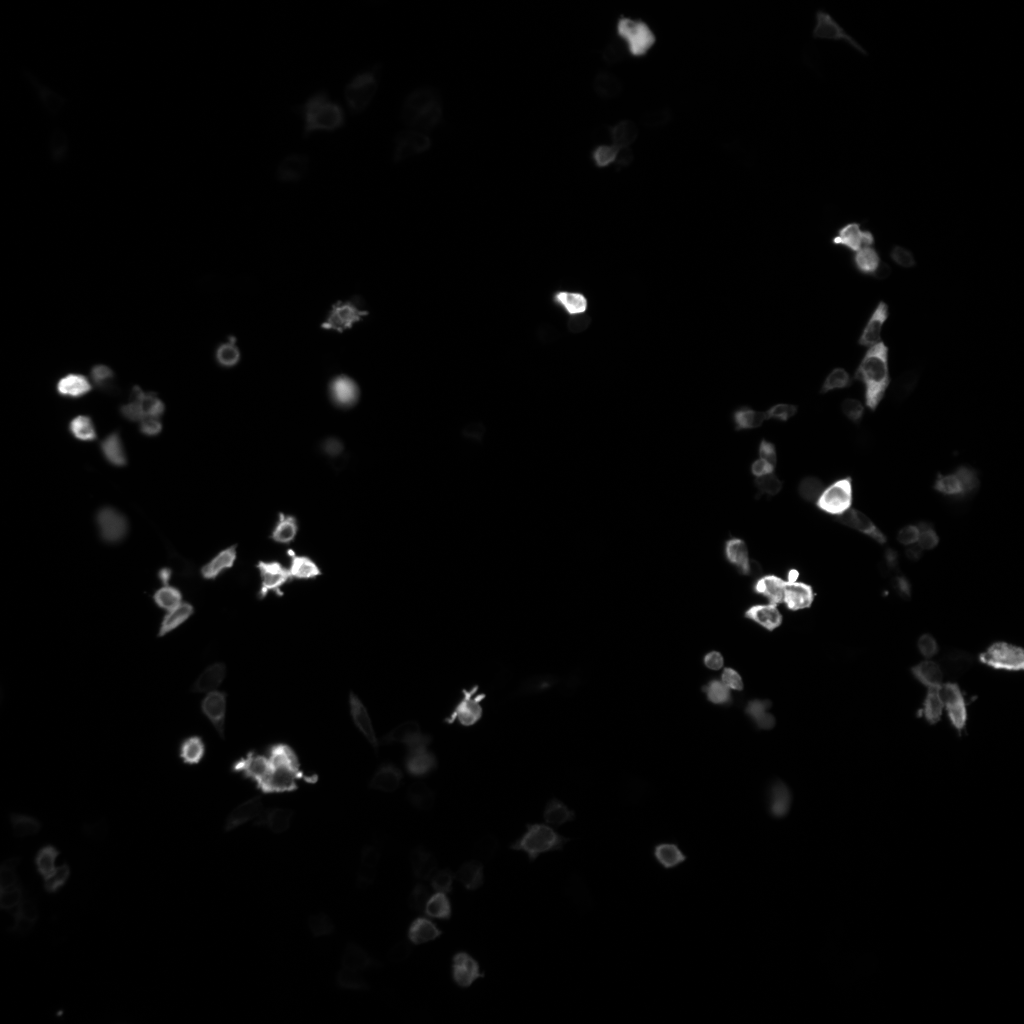

Supplement: Supplementary file 11 — Source Data [file 41467_2024_54263_MOESM11_ESM.zip › Source Data/Fig. 5/Fig. 5 B/Stable probe HEK + 300 probe + 100staygoldcontrol/fluorescence/20x/StayGoldcontrol100ng_1_X1.tif]

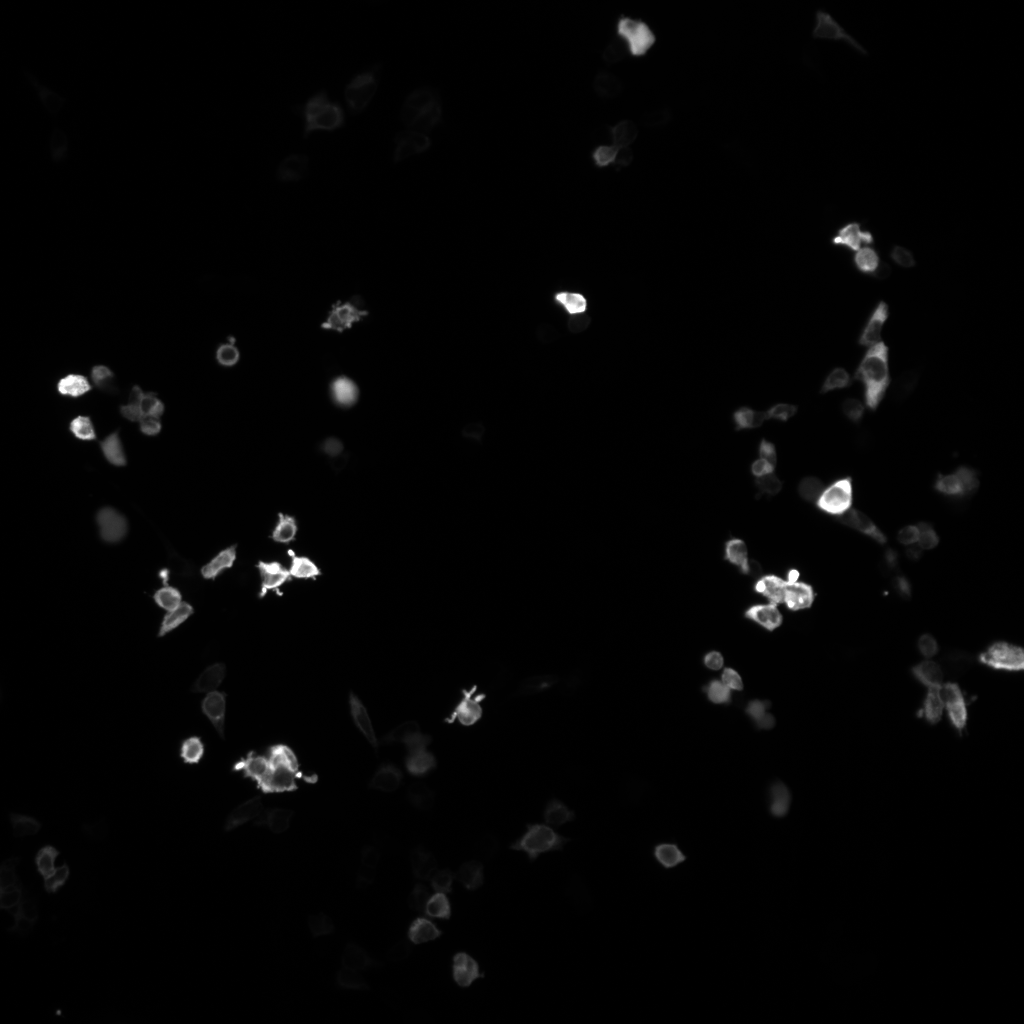

Supplement: Supplementary file 11 — Source Data [file 41467_2024_54263_MOESM11_ESM.zip › Source Data/Fig. 5/Fig. 5 B/Stable probe HEK + 300 probe + 100staygoldcontrol/fluorescence/20x/StayGoldcontrol100ng_3_X1.tif]

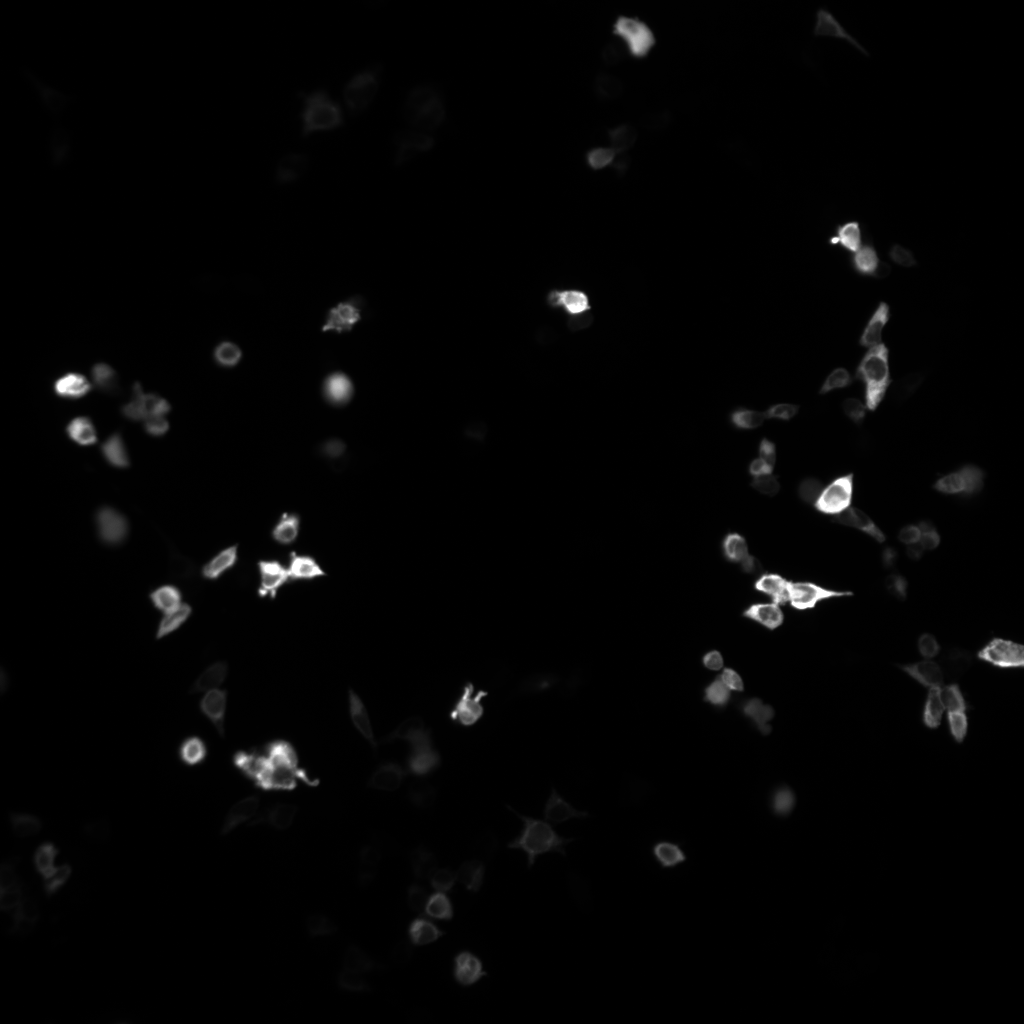

Supplement: Supplementary file 11 — Source Data [file 41467_2024_54263_MOESM11_ESM.zip › Source Data/Fig. 5/Fig. 5 B/Stable probe HEK + 300 probe + 100staygoldcontrol/fluorescence/20x/StayGold_3_X1.tif]

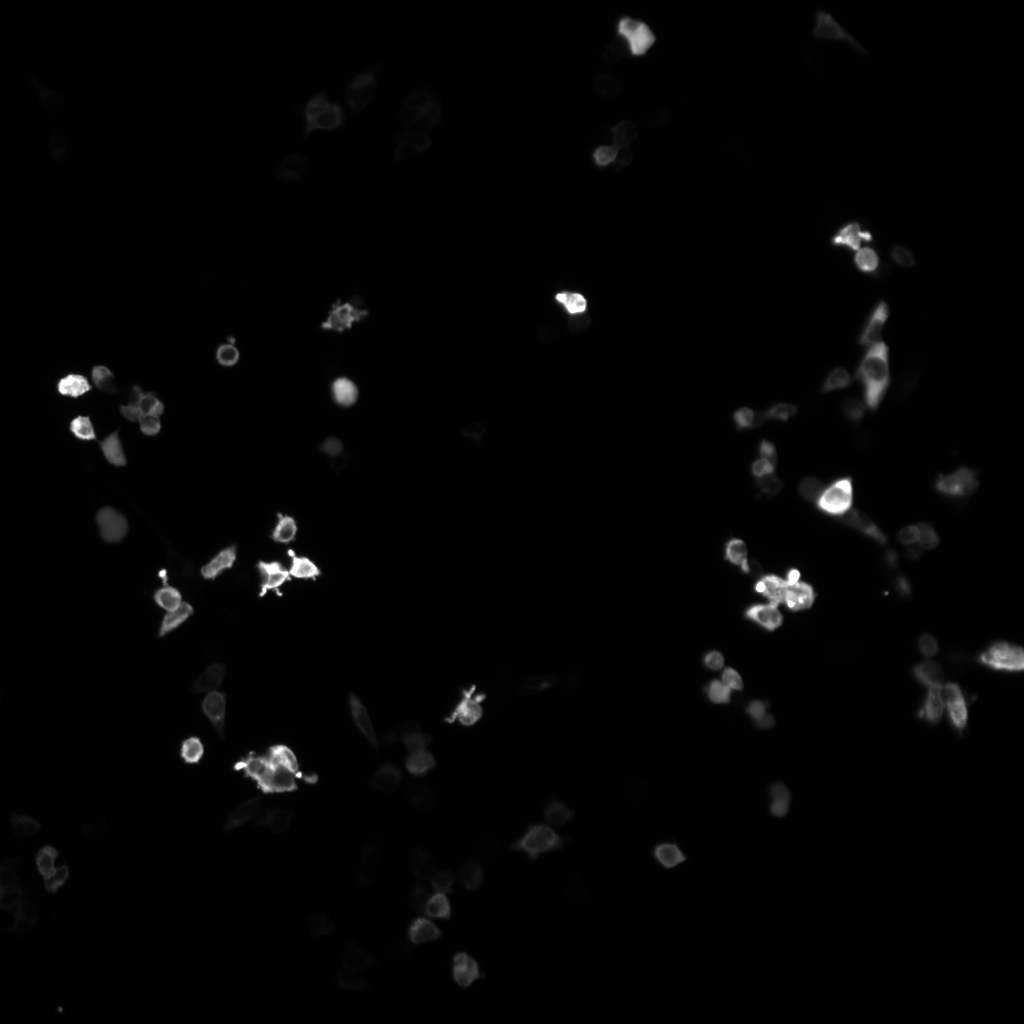

Supplement: Supplementary file 11 — Source Data [file 41467_2024_54263_MOESM11_ESM.zip › Source Data/Fig. 5/Fig. 5 B/Stable probe HEK + 300 probe + 100staygoldcontrol/fluorescence/20x/StayGoldcontrol100ng_7_X1.tif]

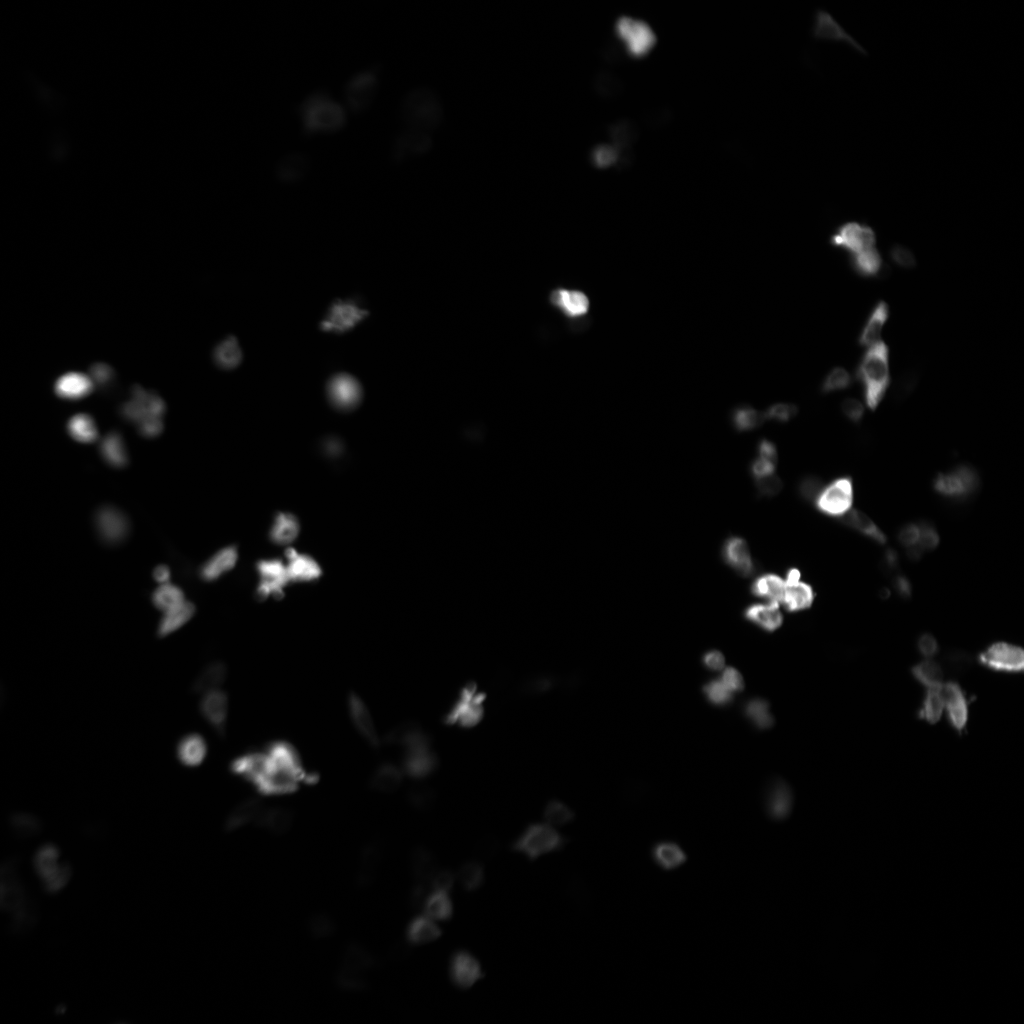

Supplement: Supplementary file 11 — Source Data [file 41467_2024_54263_MOESM11_ESM.zip › Source Data/Fig. 5/Fig. 5 B/Stable probe HEK + 300 probe + 100staygoldcontrol/fluorescence/20x/StayGoldcontrol100ng_11_X1.tif]

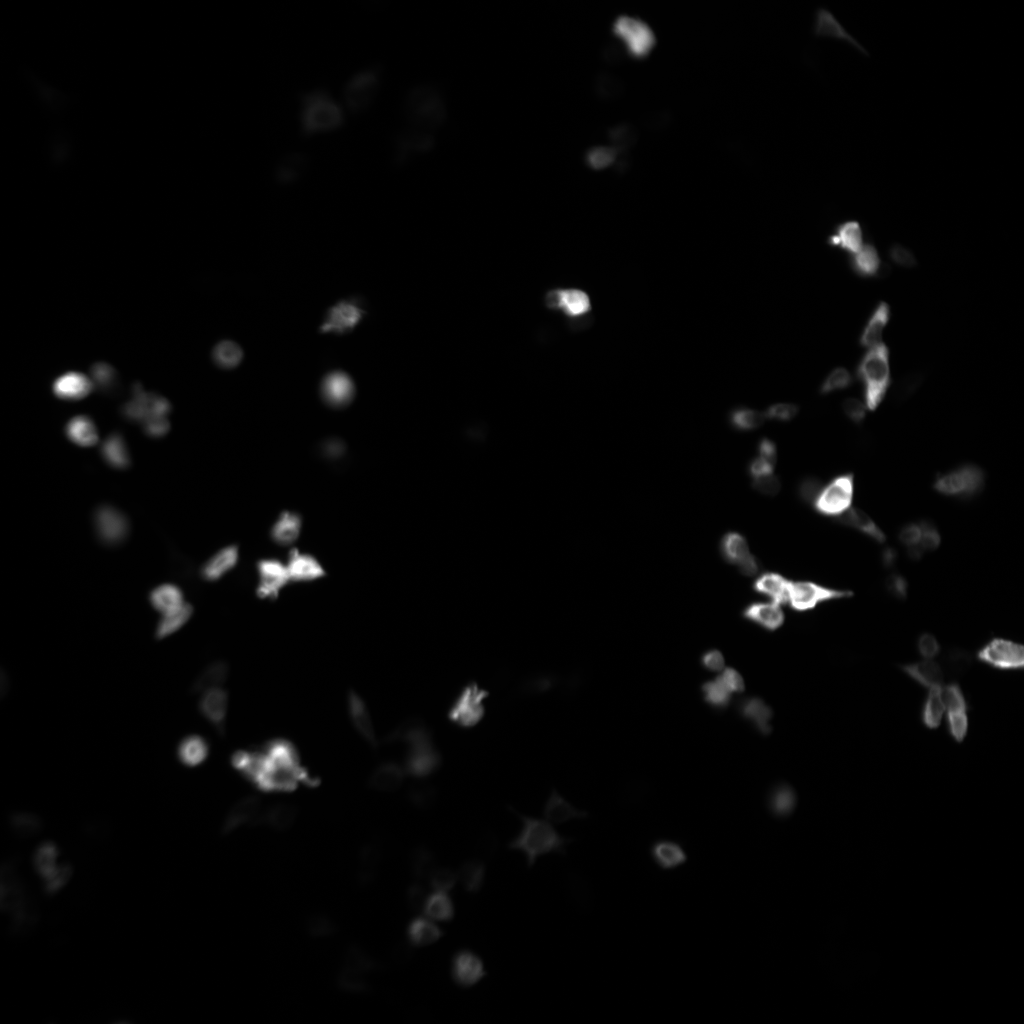

Supplement: Supplementary file 11 — Source Data [file 41467_2024_54263_MOESM11_ESM.zip › Source Data/Fig. 5/Fig. 5 B/Stable probe HEK + 300 probe + 100staygoldcontrol/fluorescence/20x/StayGold_5_X1.tif]

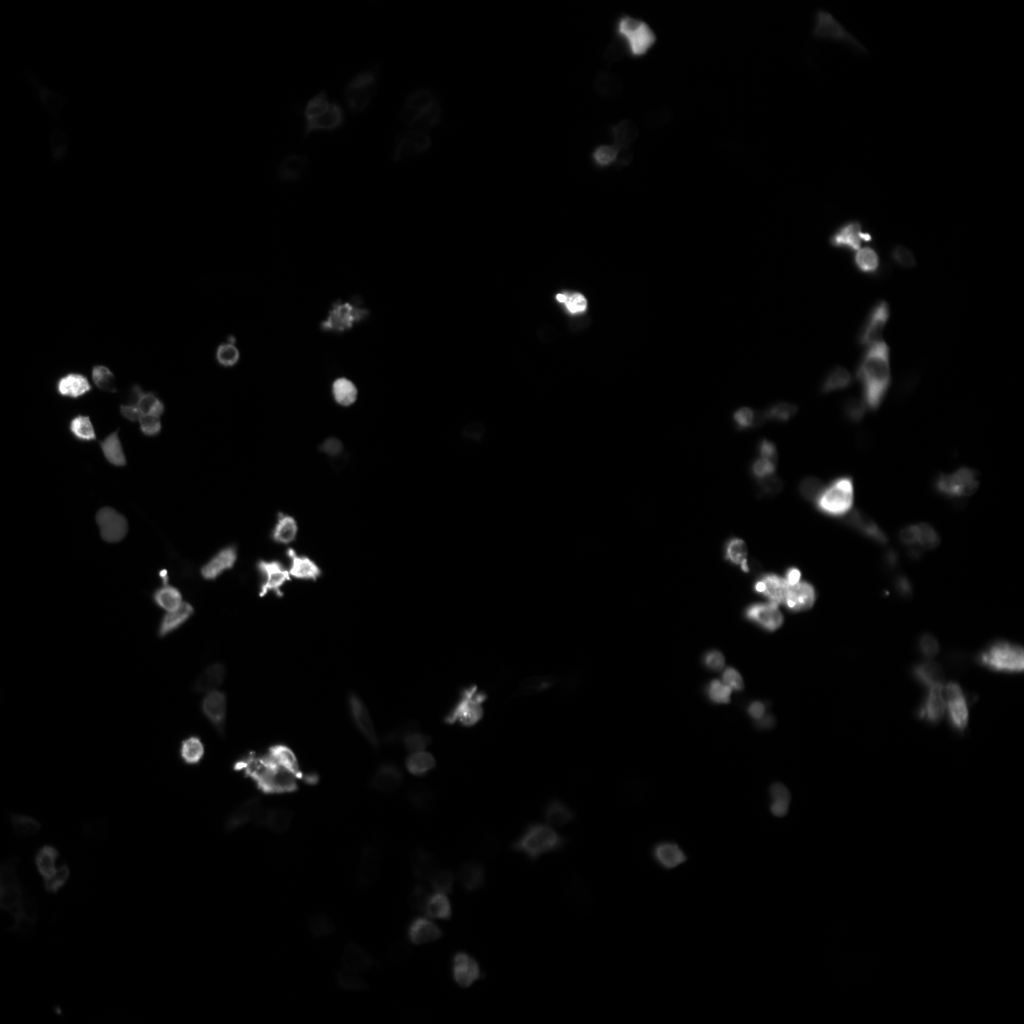

Supplement: Supplementary file 11 — Source Data [file 41467_2024_54263_MOESM11_ESM.zip › Source Data/Fig. 5/Fig. 5 B/Stable probe HEK + 300 probe + 100staygoldcontrol/fluorescence/20x/StayGoldcontrol100ng_5_X1.tif]

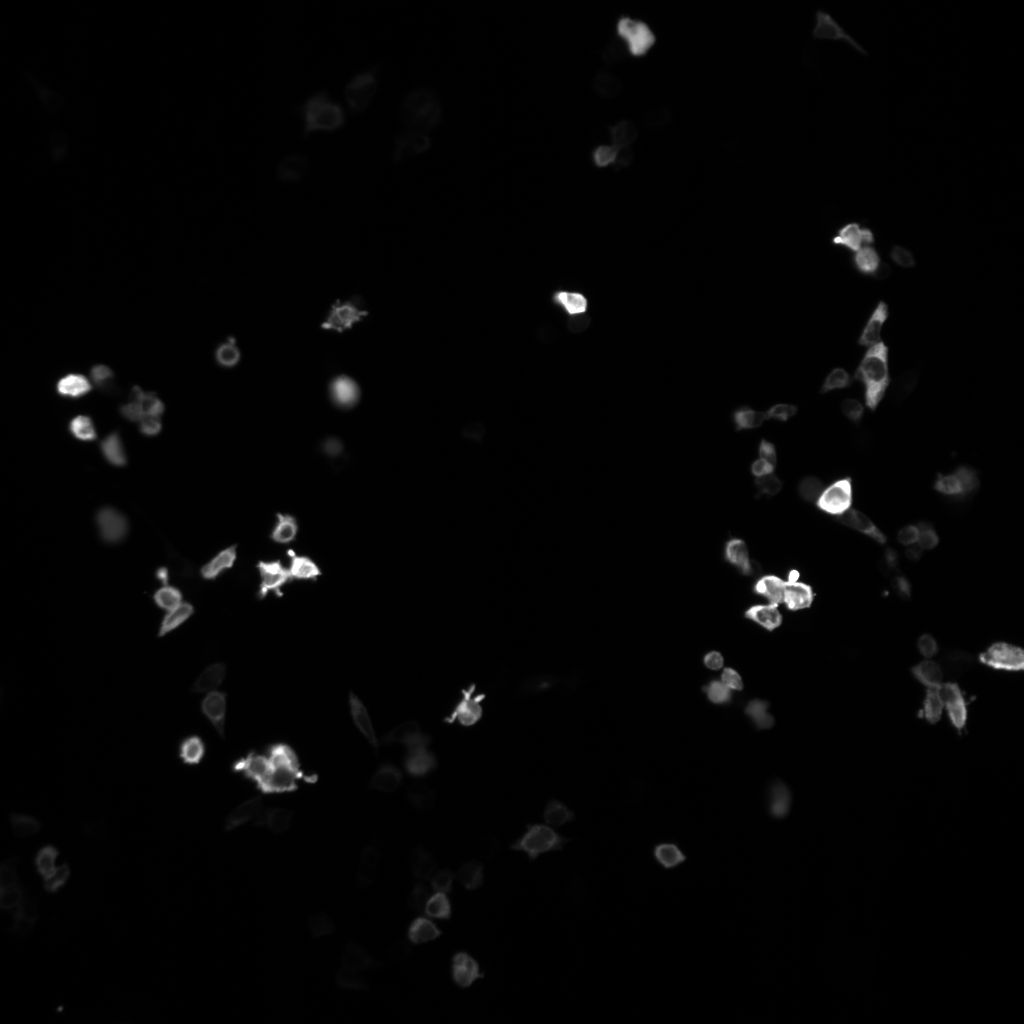

Supplement: Supplementary file 11 — Source Data [file 41467_2024_54263_MOESM11_ESM.zip › Source Data/Fig. 5/Fig. 5 B/Stable probe HEK + 300 probe + 100staygoldcontrol/fluorescence/20x/StayGoldcontrol100ng_9_X1.tif]

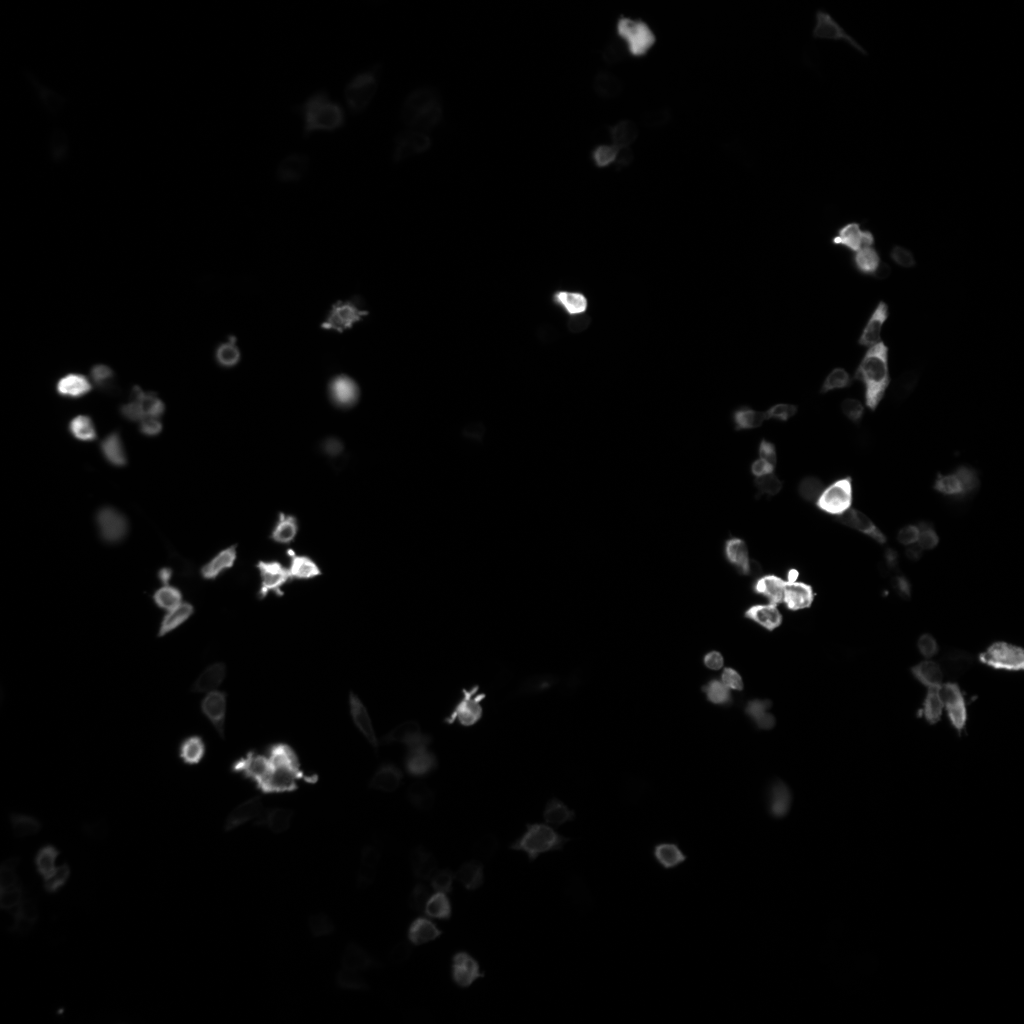

Supplement: Supplementary file 11 — Source Data [file 41467_2024_54263_MOESM11_ESM.zip › Source Data/Fig. 5/Fig. 5 B/Stable probe HEK + 300 probe + 100staygoldcontrol/fluorescence/20x/StayGoldcontrol100ng_0_X1.tif]

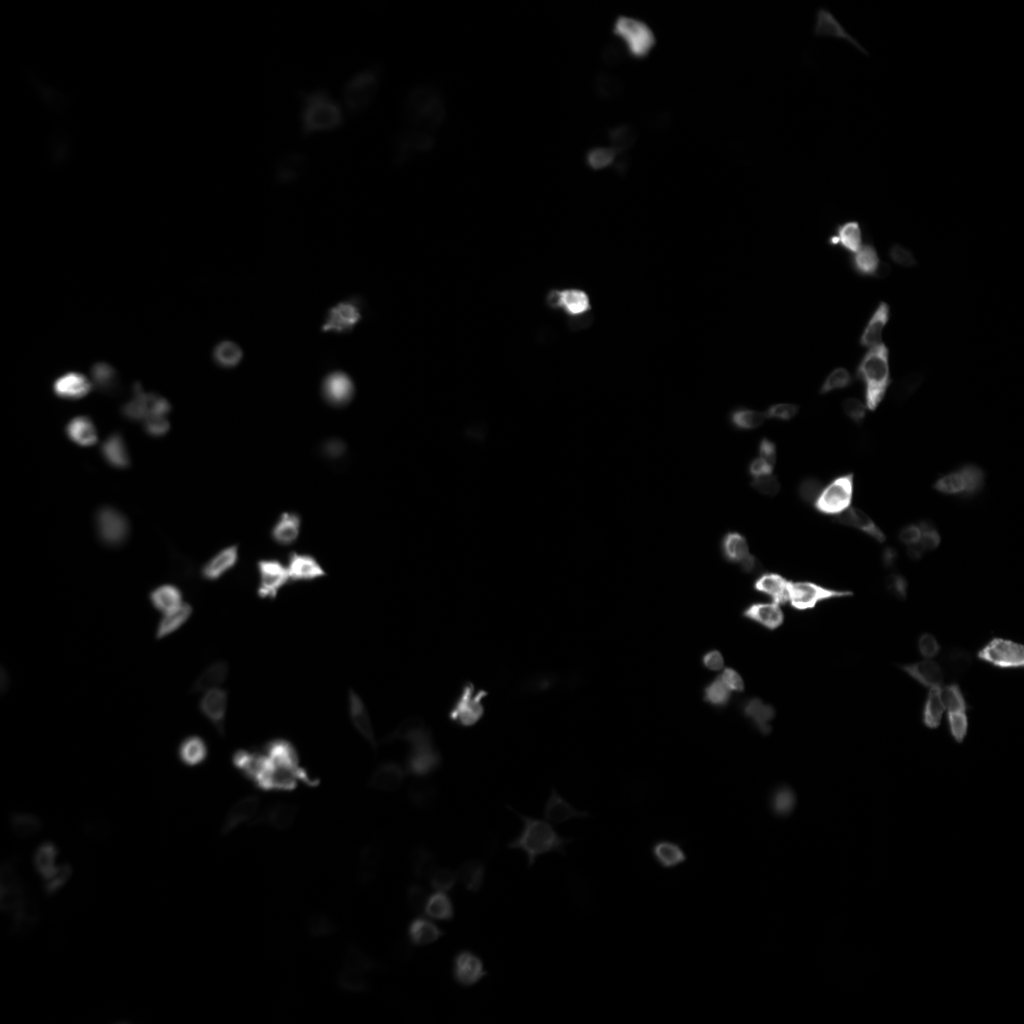

Supplement: Supplementary file 11 — Source Data [file 41467_2024_54263_MOESM11_ESM.zip › Source Data/Fig. 5/Fig. 5 B/Stable probe HEK + 300 probe + 100staygoldcontrol/fluorescence/20x/StayGold_0_X1.tif]

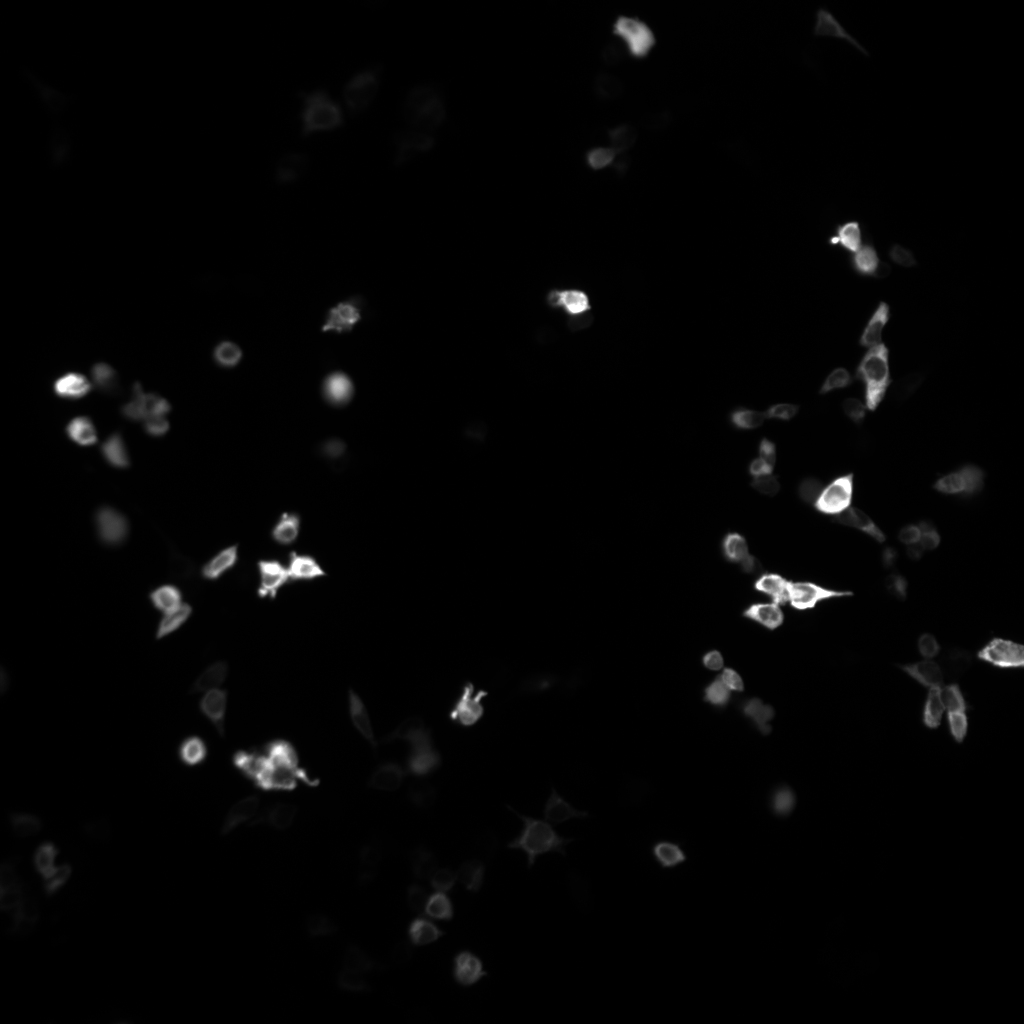

Supplement: Supplementary file 11 — Source Data [file 41467_2024_54263_MOESM11_ESM.zip › Source Data/Fig. 5/Fig. 5 B/Stable probe HEK + 300 probe + 100staygoldcontrol/fluorescence/20x/StayGold_2_X1.tif]

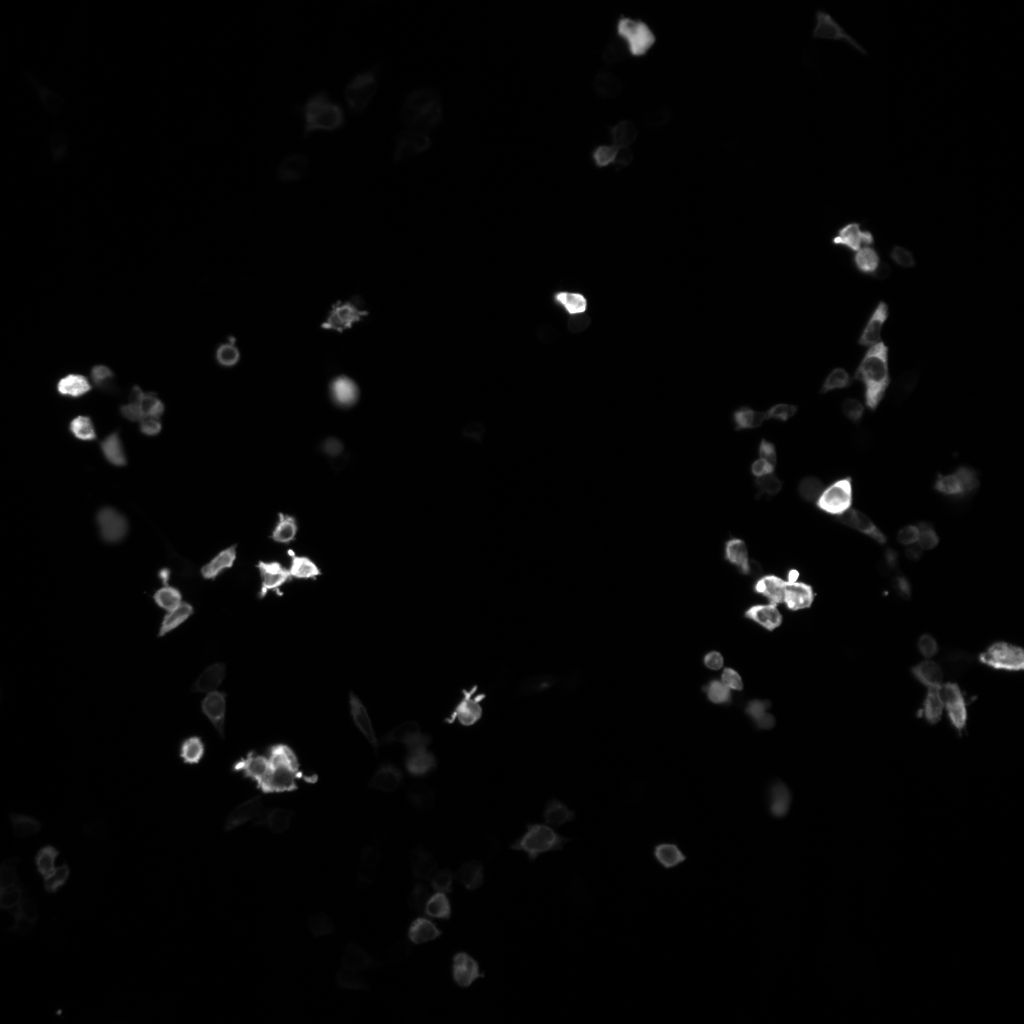

Supplement: Supplementary file 11 — Source Data [file 41467_2024_54263_MOESM11_ESM.zip › Source Data/Fig. 5/Fig. 5 B/Stable probe HEK + 300 probe + 100staygoldcontrol/fluorescence/20x/StayGoldcontrol100ng_2_X1.tif]

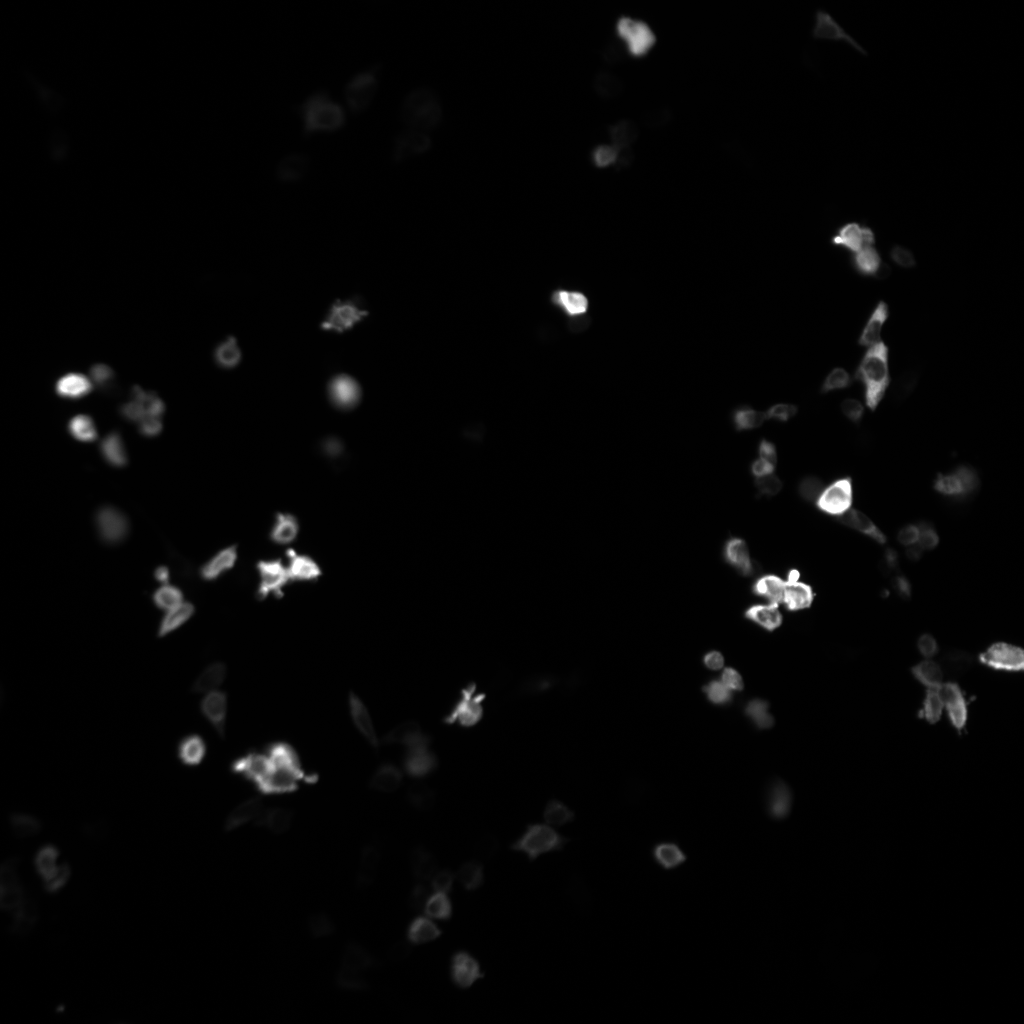

Supplement: Supplementary file 11 — Source Data [file 41467_2024_54263_MOESM11_ESM.zip › Source Data/Fig. 5/Fig. 5 B/Stable probe HEK + 300 probe + 100staygoldcontrol/fluorescence/20x/StayGoldcontrol100ng_10_X1.tif]

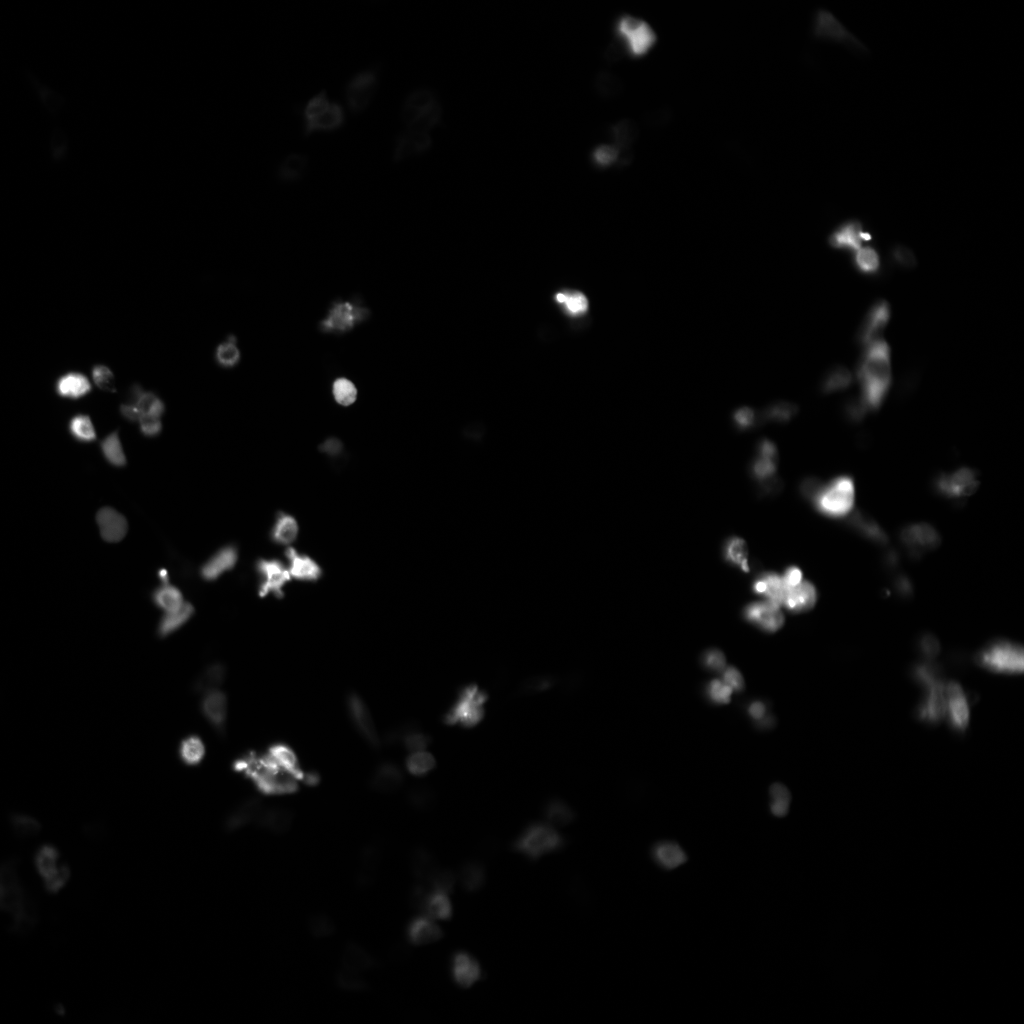

Supplement: Supplementary file 11 — Source Data [file 41467_2024_54263_MOESM11_ESM.zip › Source Data/Fig. 5/Fig. 5 B/Stable probe HEK + 300 probe + 100staygoldcontrol/fluorescence/20x/StayGoldcontrol100ng_6_X1.tif]

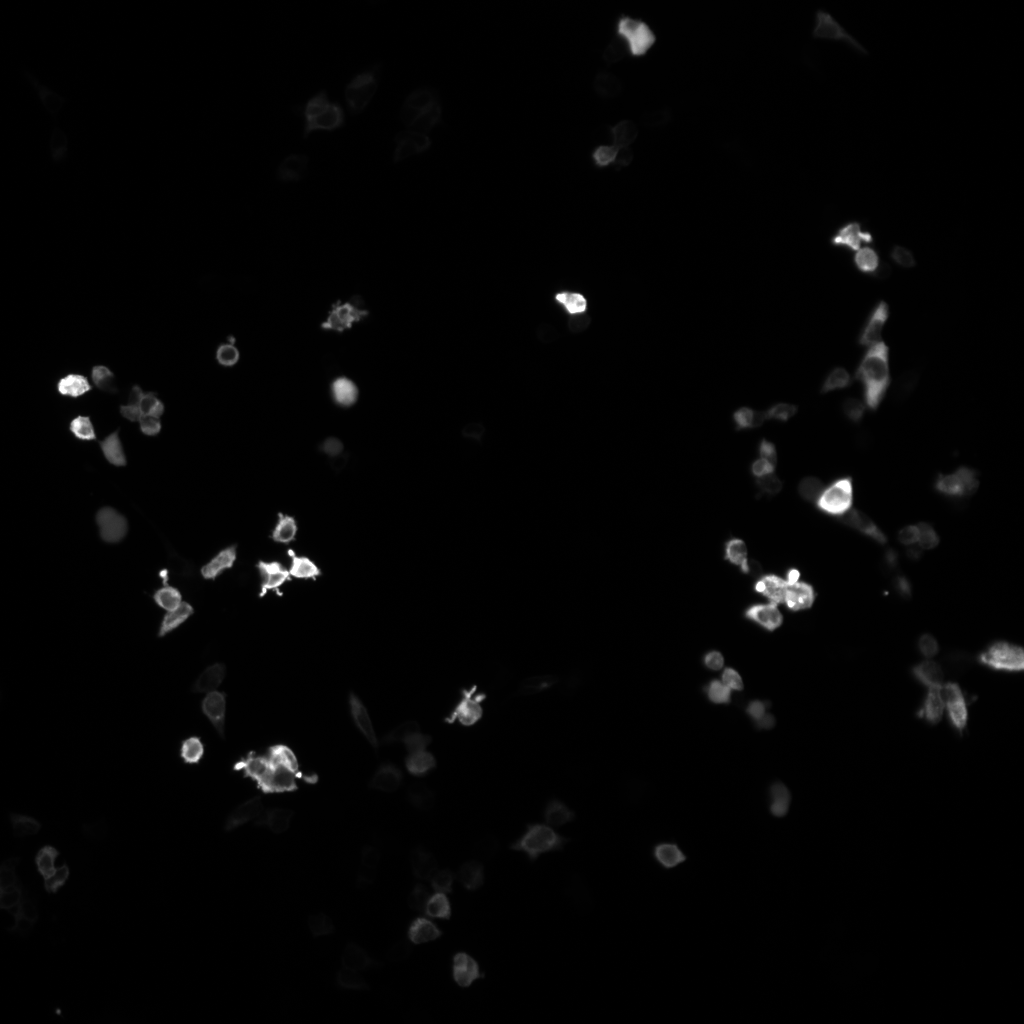

Supplement: Supplementary file 11 — Source Data [file 41467_2024_54263_MOESM11_ESM.zip › Source Data/Fig. 5/Fig. 5 B/Stable probe HEK + 300 probe + 100staygoldcontrol/fluorescence/20x/StayGoldcontrol100ng_4_X1.tif]

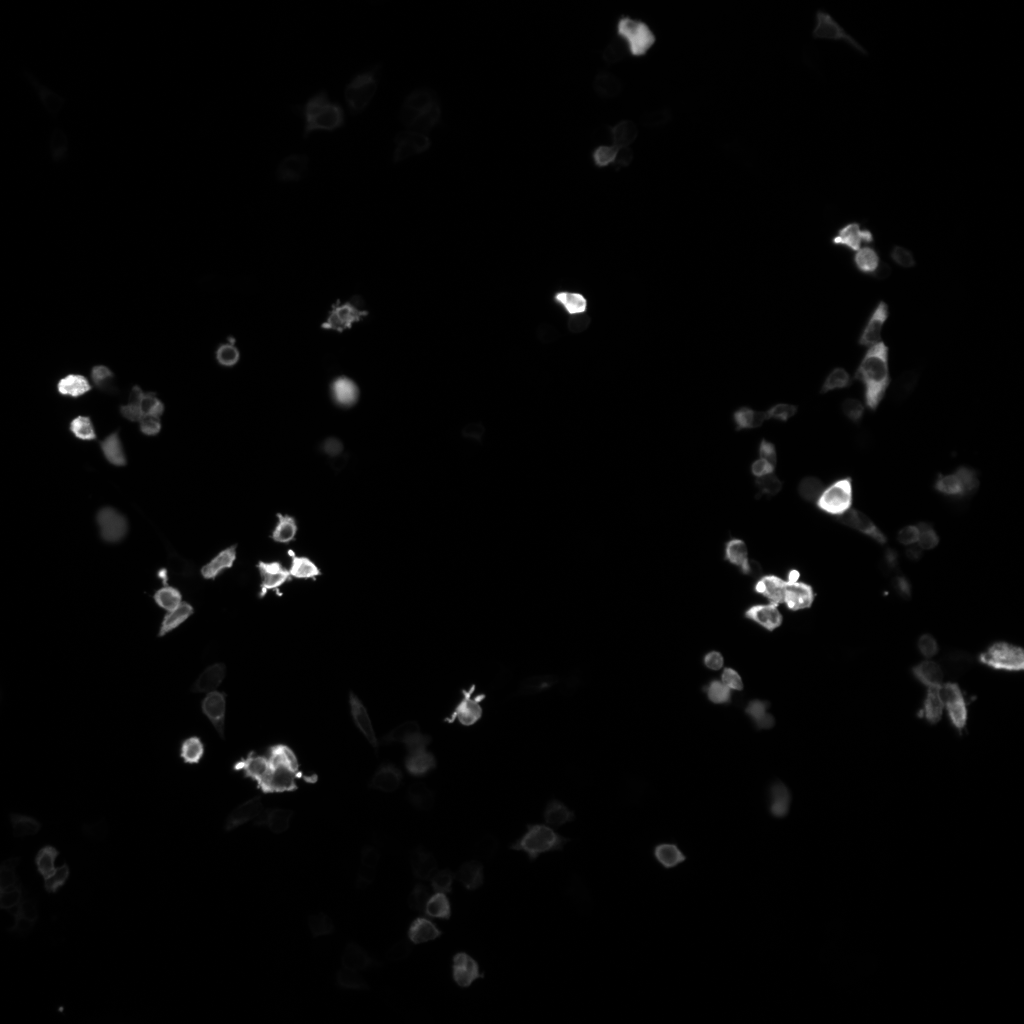

Supplement: Supplementary file 11 — Source Data [file 41467_2024_54263_MOESM11_ESM.zip › Source Data/Fig. 5/Fig. 5 B/Stable probe HEK + 300 probe + 100staygoldcontrol/fluorescence/20x/StayGoldcontrol100ng_8_X1.tif]

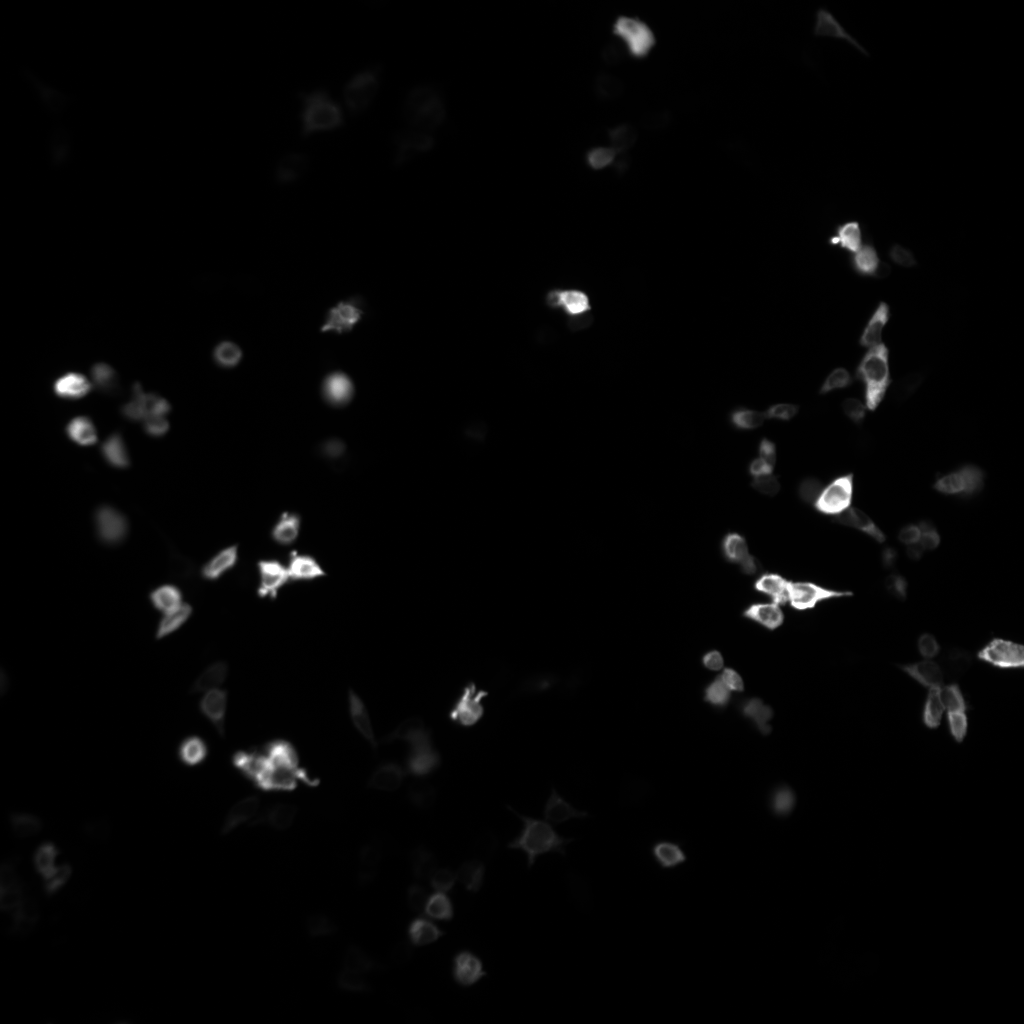

Supplement: Supplementary file 11 — Source Data [file 41467_2024_54263_MOESM11_ESM.zip › Source Data/Fig. 5/Fig. 5 B/Stable probe HEK + 300 probe + 100staygoldcontrol/fluorescence/20x/StayGold_4_X1.tif]

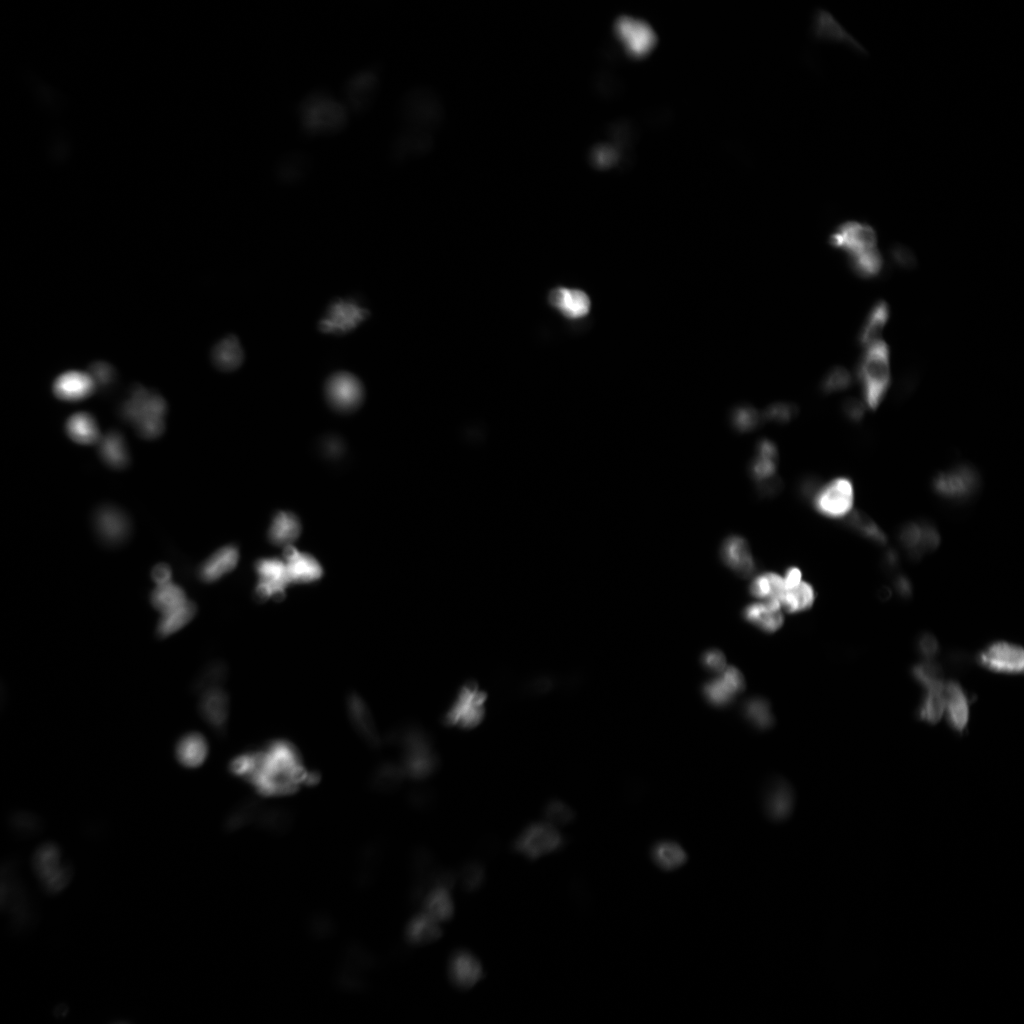

Supplement: Supplementary file 11 — Source Data [file 41467_2024_54263_MOESM11_ESM.zip › Source Data/Fig. 5/Fig. 5 B/Stable probe HEK + 300 probe + 100staygoldcontrol/fluorescence/20x/StayGoldcontrol100ng_12_X1.tif]
