# Supplementary figures and images for: A modular platform for bioluminescent RNA tracking (part 1 of 2)
Source: Nat Commun. 2024 Nov 18;15:9992. doi: 10.1038/s41467-024-54263-5 (PMC11574019; doi:10.1038/s41467-024-54263-5)

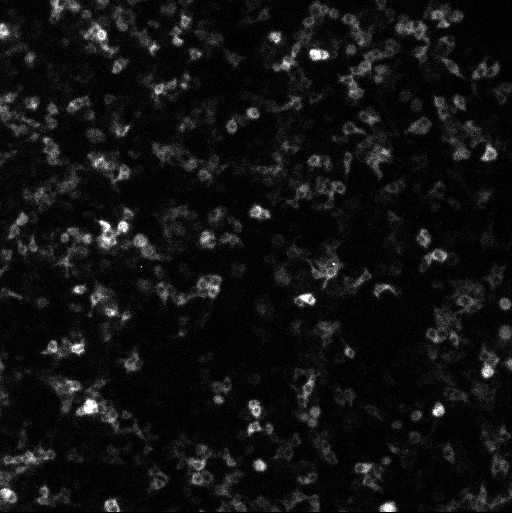

Supplement: Supplementary file 11 — Source Data [file 41467_2024_54263_MOESM11_ESM.zip › Source Data/Supplementary Fig. 8/lumi_10x_90s.tif]

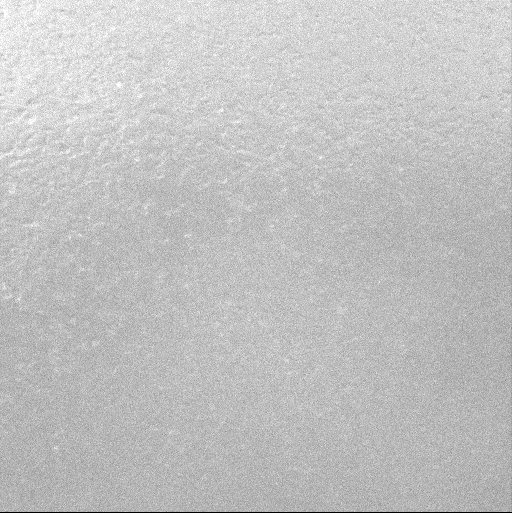

Supplement: Supplementary file 11 — Source Data [file 41467_2024_54263_MOESM11_ESM.zip › Source Data/Supplementary Fig. 8/lumi_4x_90s_5.tif]

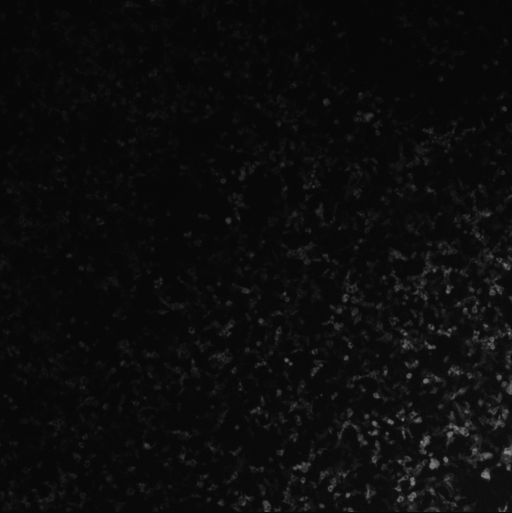

Supplement: Supplementary file 11 — Source Data [file 41467_2024_54263_MOESM11_ESM.zip › Source Data/Supplementary Fig. 8/GFP_4x_1s.tif]

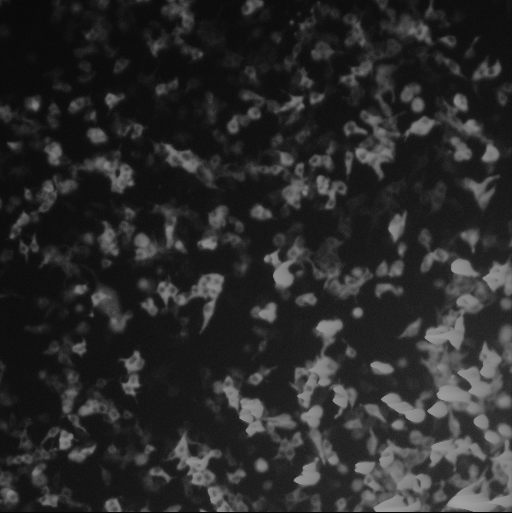

Supplement: Supplementary file 11 — Source Data [file 41467_2024_54263_MOESM11_ESM.zip › Source Data/Supplementary Fig. 8/GFP_10x_1s.tif]

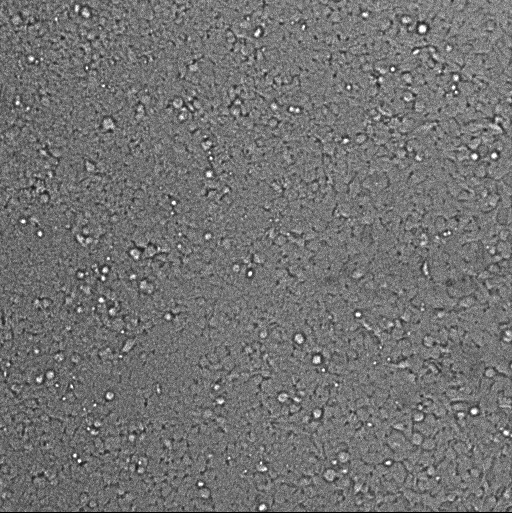

Supplement: Supplementary file 11 — Source Data [file 41467_2024_54263_MOESM11_ESM.zip › Source Data/Supplementary Fig. 8/BF_10x_point05s.tif]

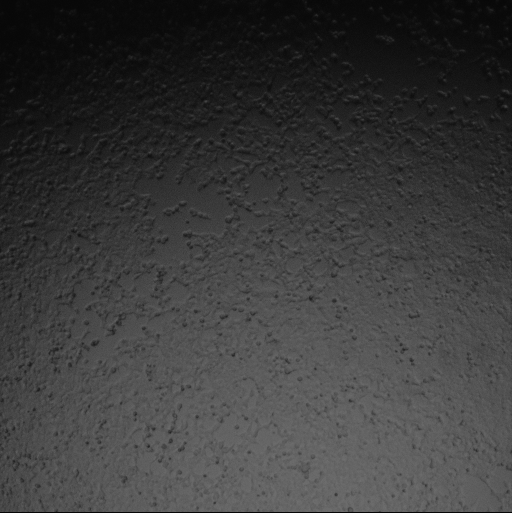

Supplement: Supplementary file 11 — Source Data [file 41467_2024_54263_MOESM11_ESM.zip › Source Data/Supplementary Fig. 8/BF_4x_point5s.tif]

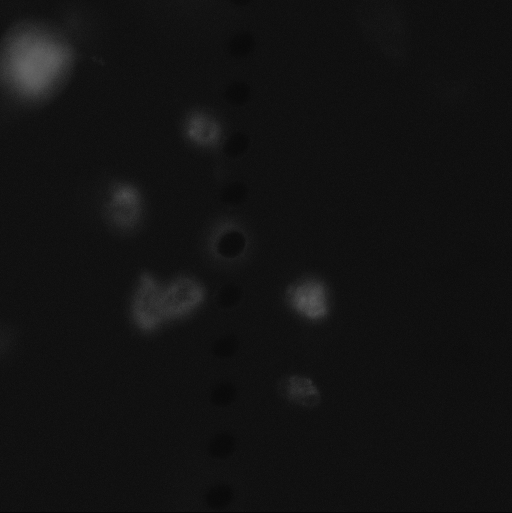

Supplement: Supplementary file 11 — Source Data [file 41467_2024_54263_MOESM11_ESM.zip › Source Data/Supplementary Fig. 13/gfp_after_0.tif]

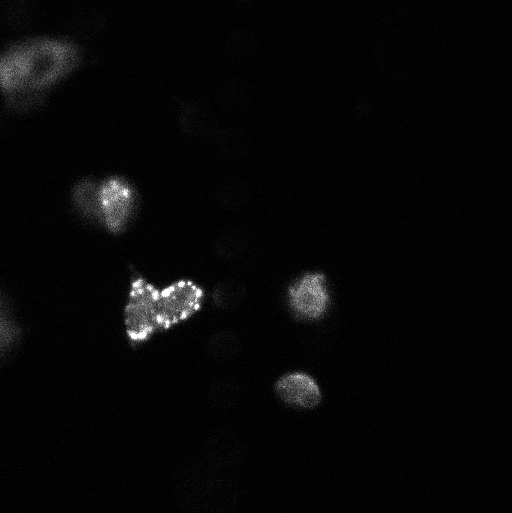

Supplement: Supplementary file 11 — Source Data [file 41467_2024_54263_MOESM11_ESM.zip › Source Data/Supplementary Fig. 13/_lumi__25mMaresinite_40files_90sacq.tif]

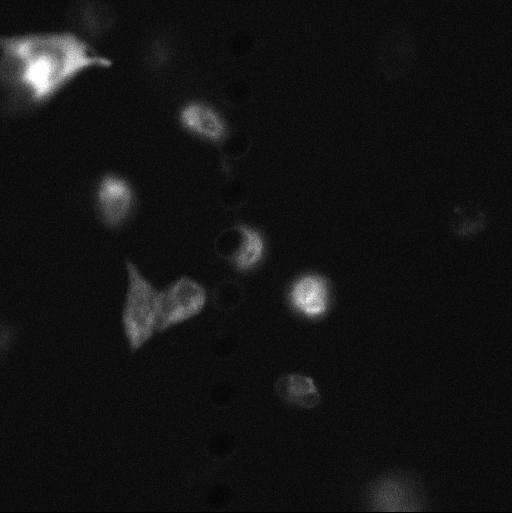

Supplement: Supplementary file 11 — Source Data [file 41467_2024_54263_MOESM11_ESM.zip › Source Data/Supplementary Fig. 13/GFP.tif]

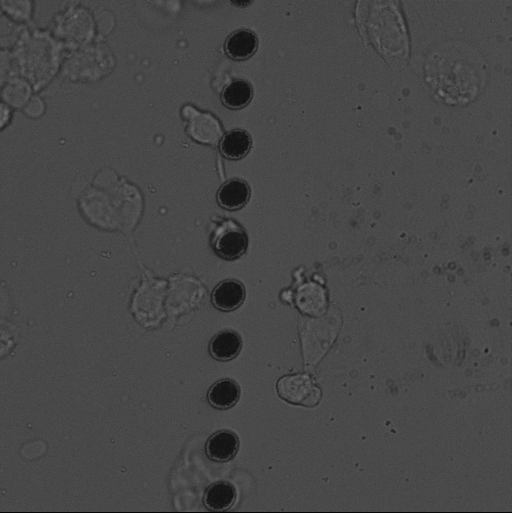

Supplement: Supplementary file 11 — Source Data [file 41467_2024_54263_MOESM11_ESM.zip › Source Data/Supplementary Fig. 13/bf_after.tif]

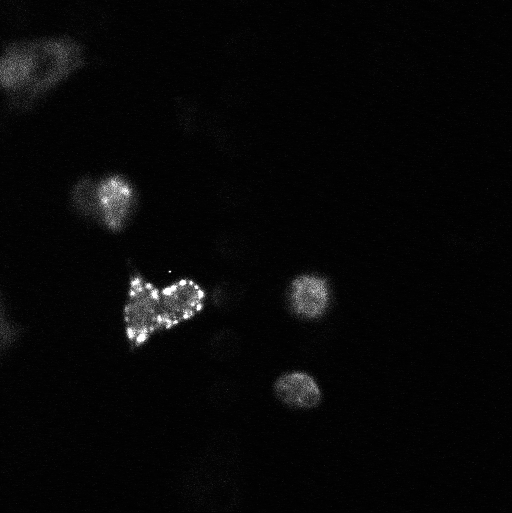

Supplement: Supplementary file 11 — Source Data [file 41467_2024_54263_MOESM11_ESM.zip › Source Data/Supplementary Fig. 13/lumi.tif]

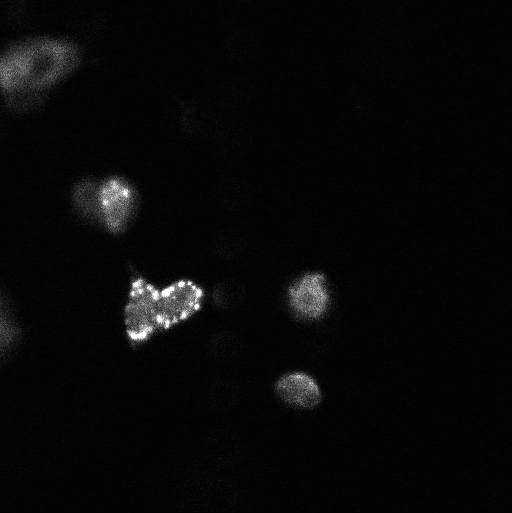

Supplement: Supplementary file 11 — Source Data [file 41467_2024_54263_MOESM11_ESM.zip › Source Data/Supplementary Fig. 13/lumi__25mMaresinite_40files_90sacq.tif]

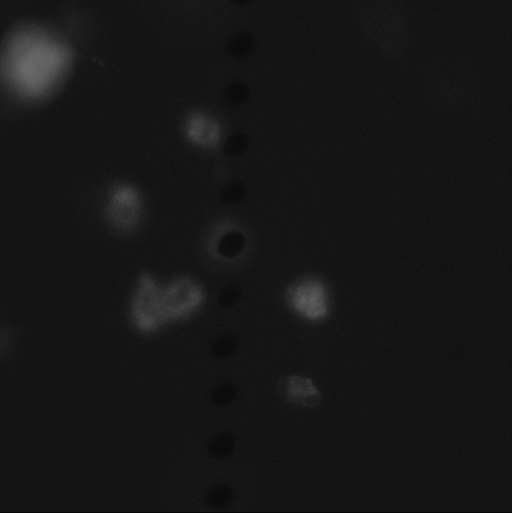

Supplement: Supplementary file 11 — Source Data [file 41467_2024_54263_MOESM11_ESM.zip › Source Data/Supplementary Fig. 13/gfp_after.tif]

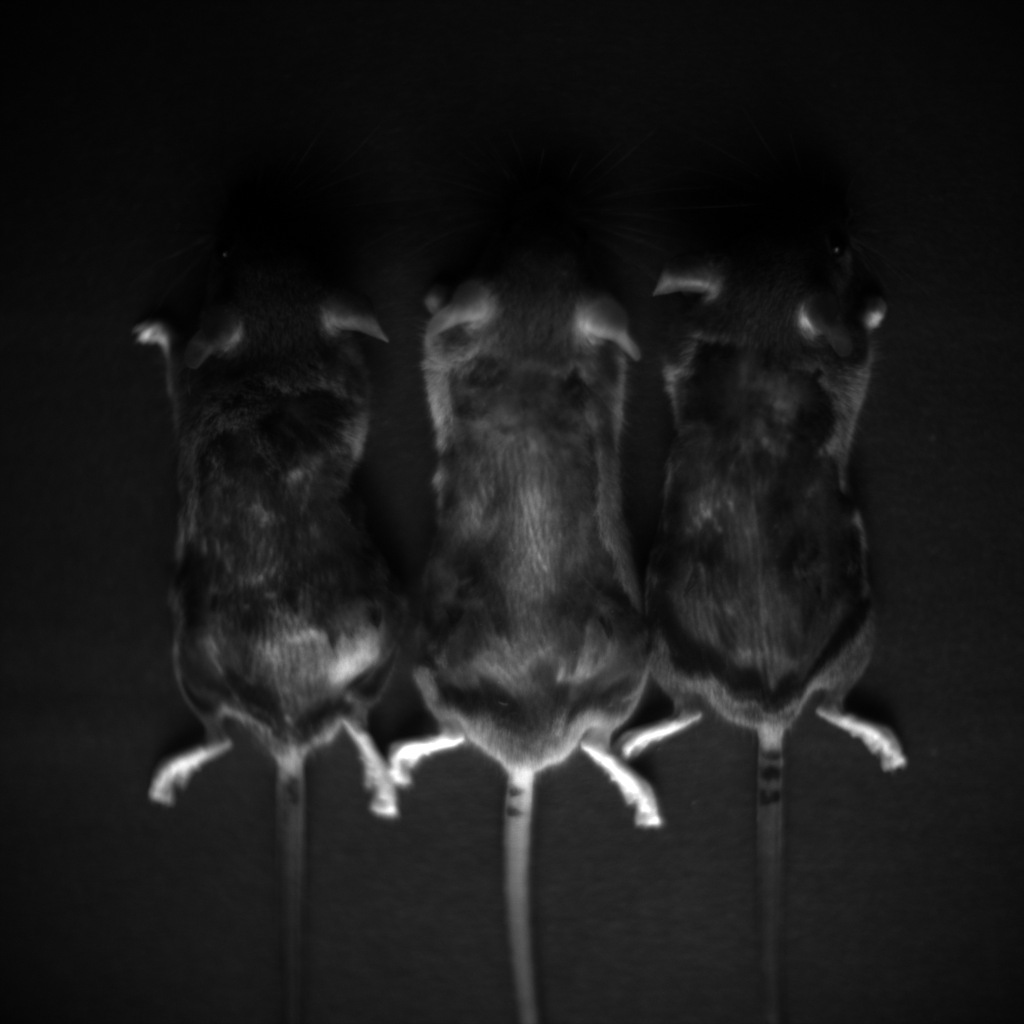

Supplement: Supplementary file 11 — Source Data [file 41467_2024_54263_MOESM11_ESM.zip › Source Data/Fig. 6/Fig. 6C BFP/brightfield-BFP.tif]

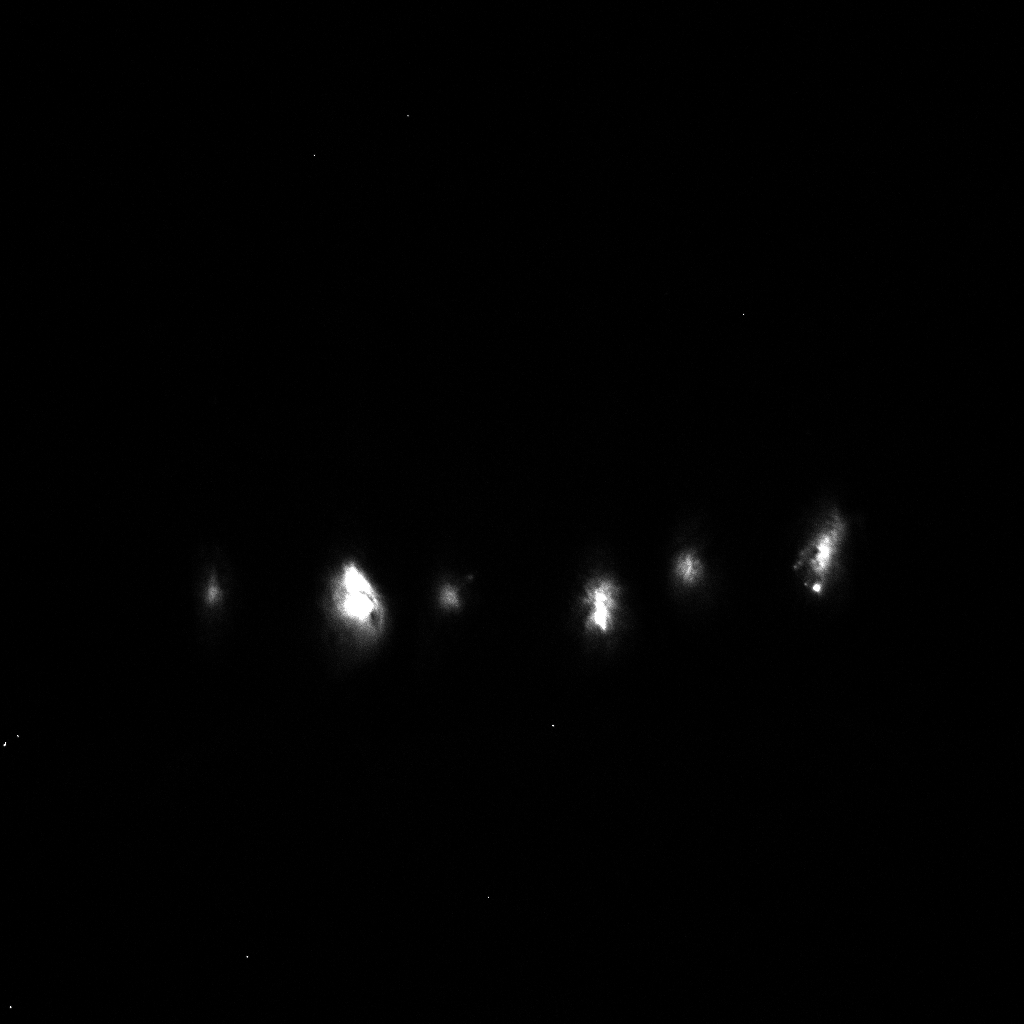

Supplement: Supplementary file 11 — Source Data [file 41467_2024_54263_MOESM11_ESM.zip › Source Data/Fig. 6/Fig. 6C BFP/300s-1000EM-BFP.tif]

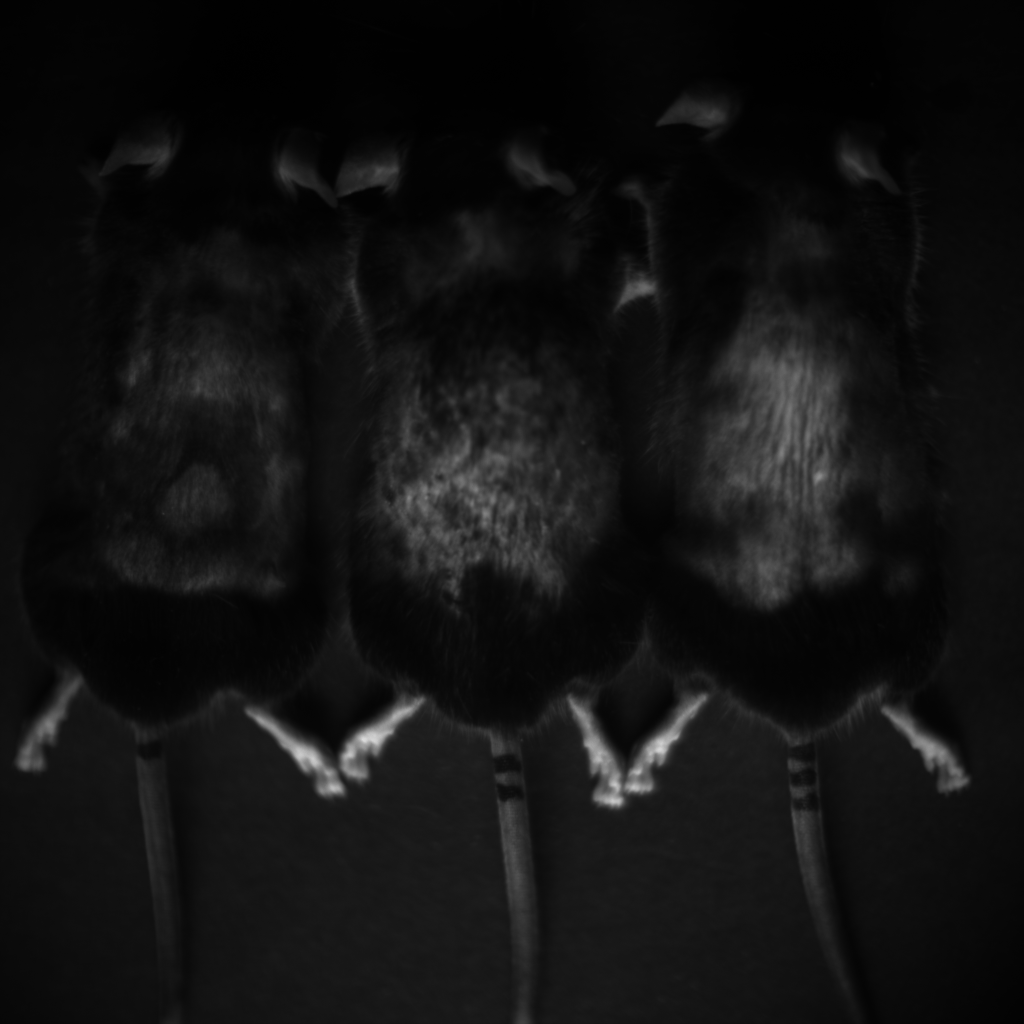

Supplement: Supplementary file 11 — Source Data [file 41467_2024_54263_MOESM11_ESM.zip › Source Data/Fig. 6/Fig. 6A GFP/GFP_brightfield.tif]

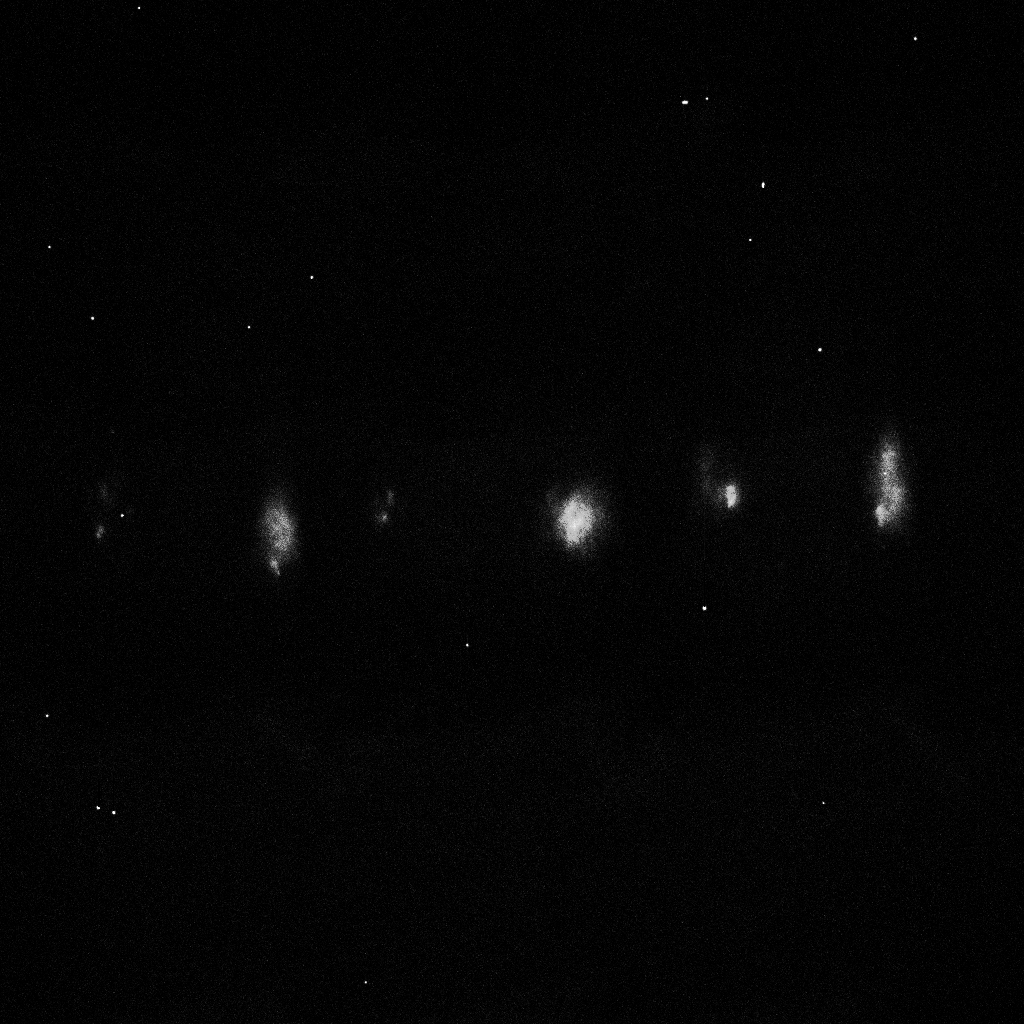

Supplement: Supplementary file 11 — Source Data [file 41467_2024_54263_MOESM11_ESM.zip › Source Data/Fig. 6/Fig. 6A GFP/300s-1000EM-GFP.tif]

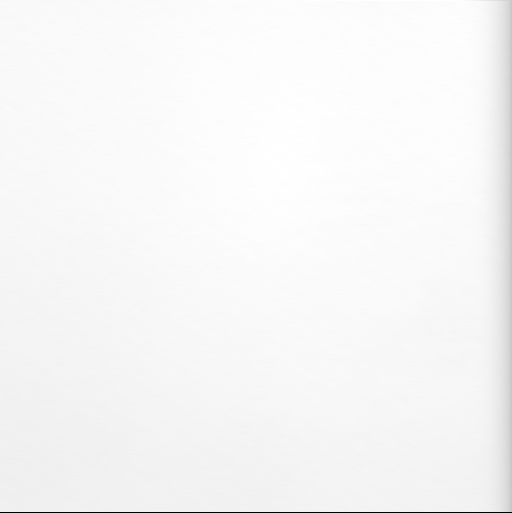

Supplement: Supplementary file 11 — Source Data [file 41467_2024_54263_MOESM11_ESM.zip › Source Data/Supplementary Fig. 11/Luminescence/lumi_15frames_90s.tif]

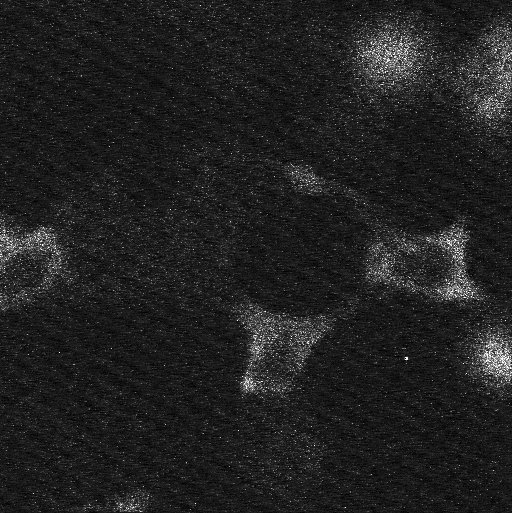

Supplement: Supplementary file 11 — Source Data [file 41467_2024_54263_MOESM11_ESM.zip › Source Data/Supplementary Fig. 11/Luminescence/lumi_15frames_90s_0.tif]

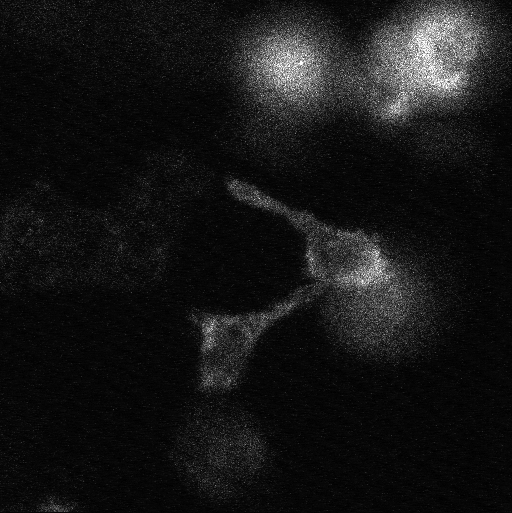

Supplement: Supplementary file 11 — Source Data [file 41467_2024_54263_MOESM11_ESM.zip › Source Data/Supplementary Fig. 11/Luminescence/lumi_2.tif]

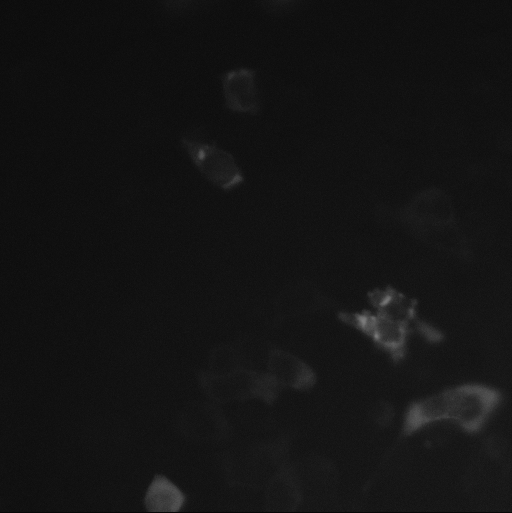

Supplement: Supplementary file 11 — Source Data [file 41467_2024_54263_MOESM11_ESM.zip › Source Data/Fig. 5/Fig. 5 D/GFP_after_point2_40x_point5s_0.tif]

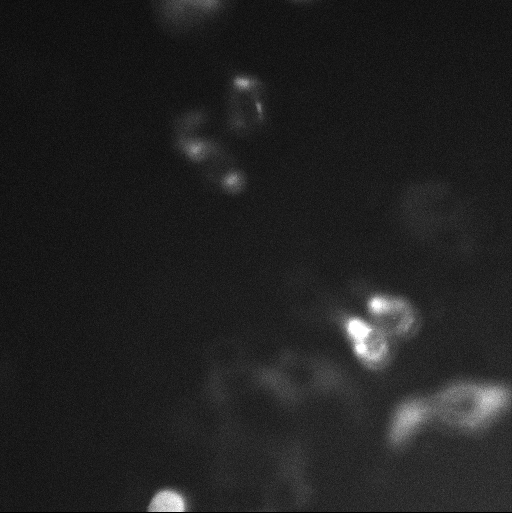

Supplement: Supplementary file 11 — Source Data [file 41467_2024_54263_MOESM11_ESM.zip › Source Data/Fig. 5/Fig. 5 D/GFP_before_40x_point5s_1000EM_2.tif]

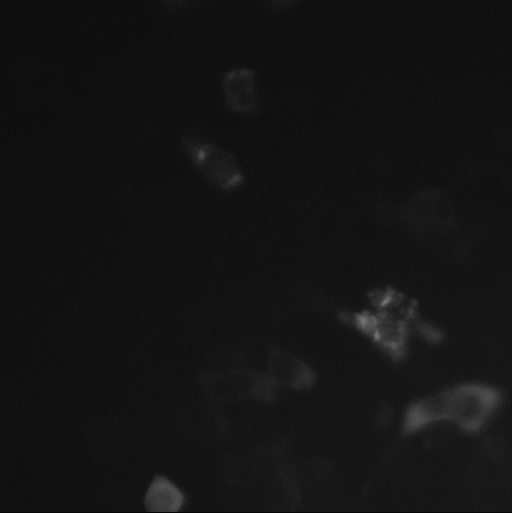

Supplement: Supplementary file 11 — Source Data [file 41467_2024_54263_MOESM11_ESM.zip › Source Data/Fig. 5/Fig. 5 D/GFP_after_point2_40x_point5s_1.tif]

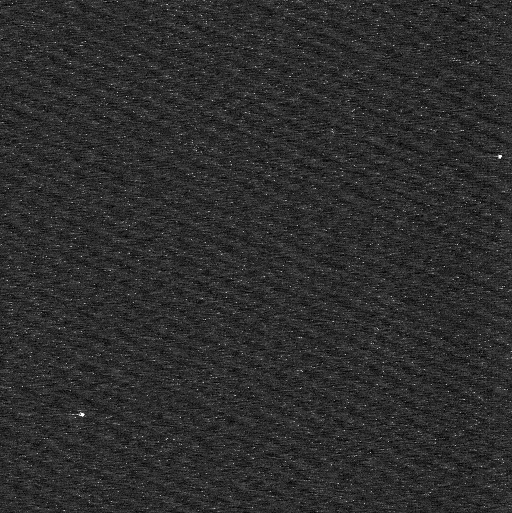

Supplement: Supplementary file 11 — Source Data [file 41467_2024_54263_MOESM11_ESM.zip › Source Data/Fig. 5/Fig. 5 D/lumi_90s__1000EM_40frames.tif]

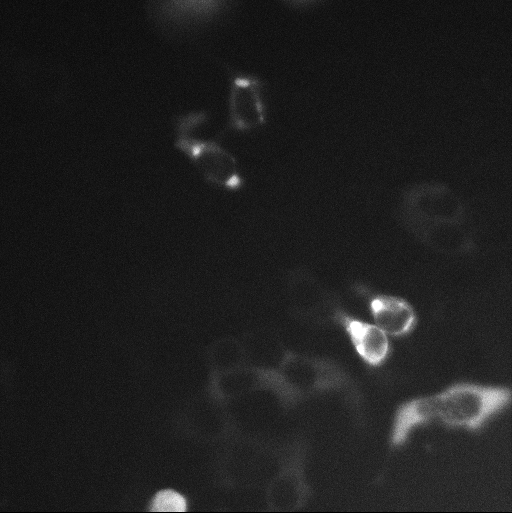

Supplement: Supplementary file 11 — Source Data [file 41467_2024_54263_MOESM11_ESM.zip › Source Data/Fig. 5/Fig. 5 D/GFP_before_40x_point5s_1000EM_0.tif]

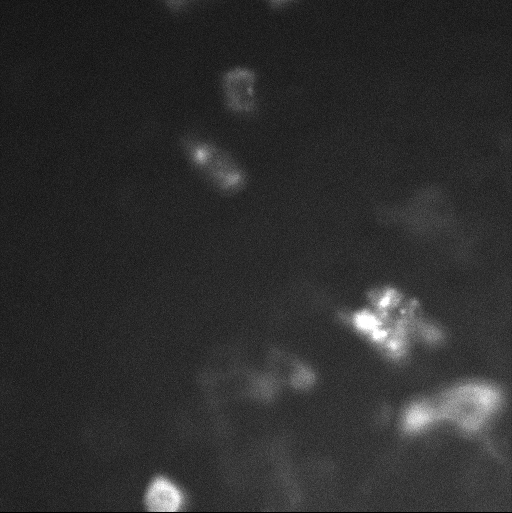

Supplement: Supplementary file 11 — Source Data [file 41467_2024_54263_MOESM11_ESM.zip › Source Data/Fig. 5/Fig. 5 D/GFP_after_point2_40x_point5s_3.tif]

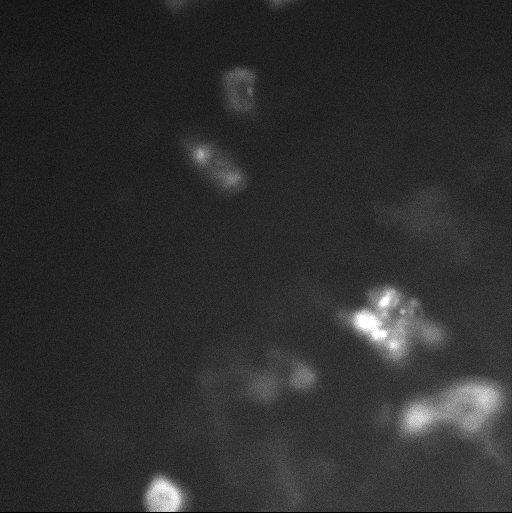

Supplement: Supplementary file 11 — Source Data [file 41467_2024_54263_MOESM11_ESM.zip › Source Data/Fig. 5/Fig. 5 D/GFP_after_point2_40x_point5s_2.tif]

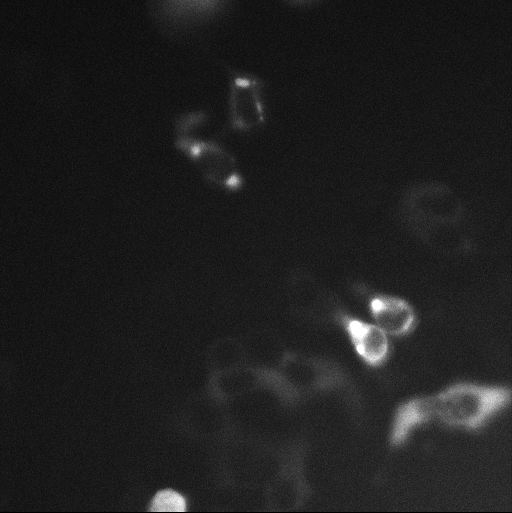

Supplement: Supplementary file 11 — Source Data [file 41467_2024_54263_MOESM11_ESM.zip › Source Data/Fig. 5/Fig. 5 D/GFP_before_40x_point5s_1000EM_1.tif]

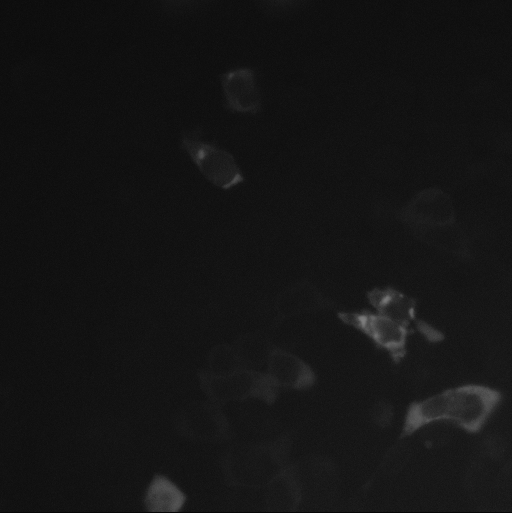

Supplement: Supplementary file 11 — Source Data [file 41467_2024_54263_MOESM11_ESM.zip › Source Data/Fig. 5/Fig. 5 D/GFP_after_point2_40x_point5s.tif]

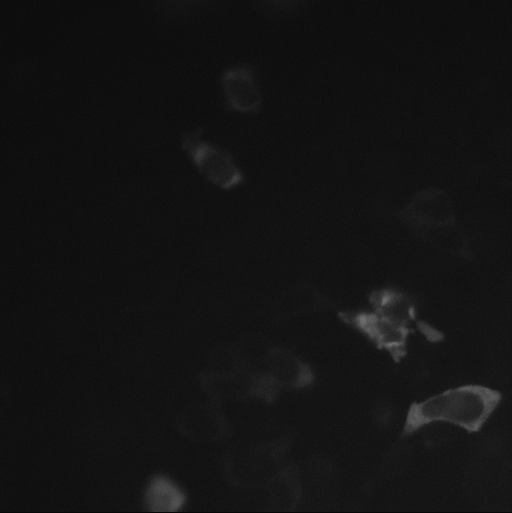

Supplement: Supplementary file 11 — Source Data [file 41467_2024_54263_MOESM11_ESM.zip › Source Data/Fig. 5/Fig. 5 D/GFP_after_point2_40x_point5s_5.tif]

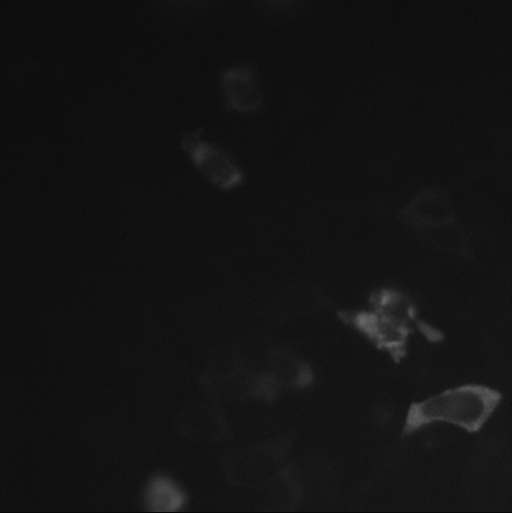

Supplement: Supplementary file 11 — Source Data [file 41467_2024_54263_MOESM11_ESM.zip › Source Data/Fig. 5/Fig. 5 D/GFP_after_point2_40x_point5s_4.tif]

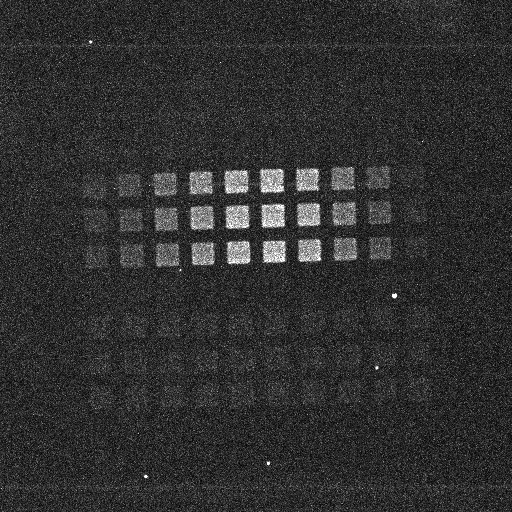

Supplement: Supplementary file 11 — Source Data [file 41467_2024_54263_MOESM11_ESM.zip › Source Data/Fig. 2/Fig. 2B/Luminescence.tif]

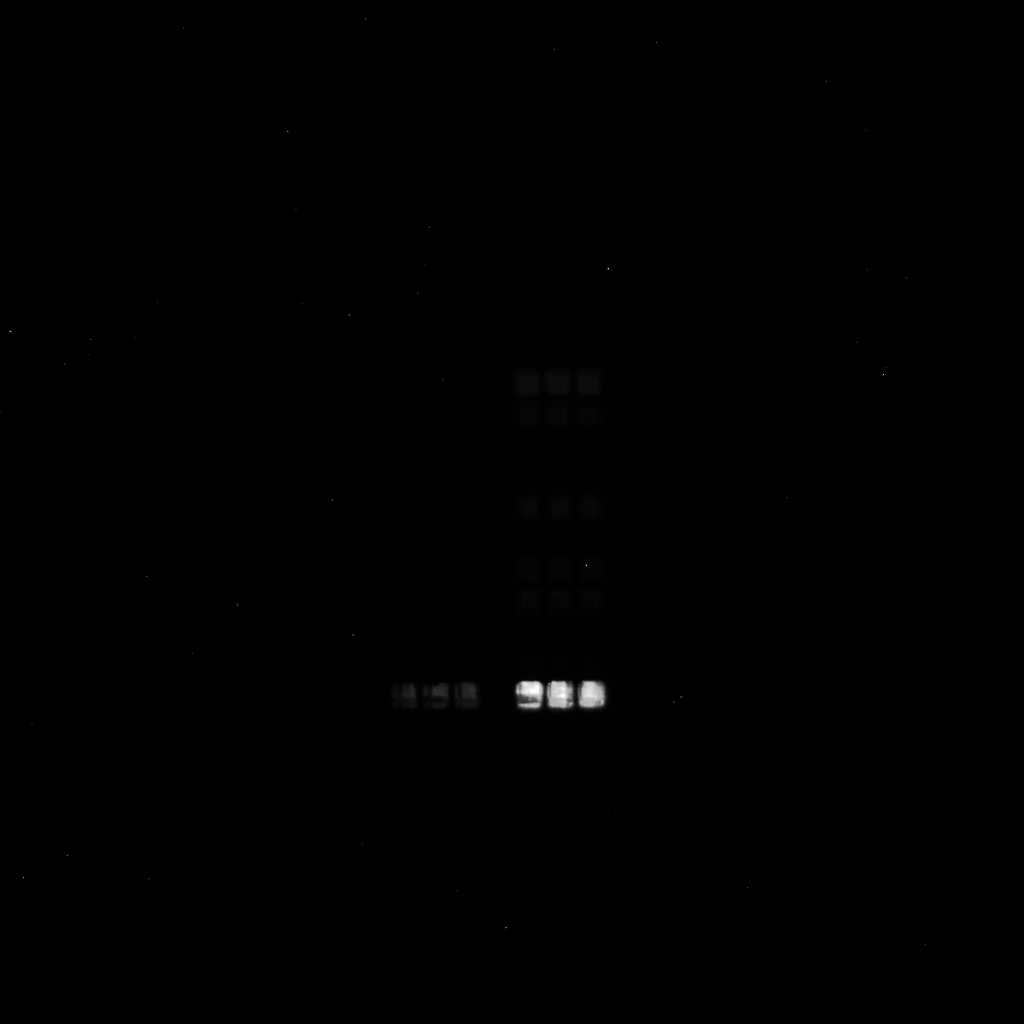

Supplement: Supplementary file 11 — Source Data [file 41467_2024_54263_MOESM11_ESM.zip › Source Data/Fig. 2/Fig. 2E/FLAG beads.tif]

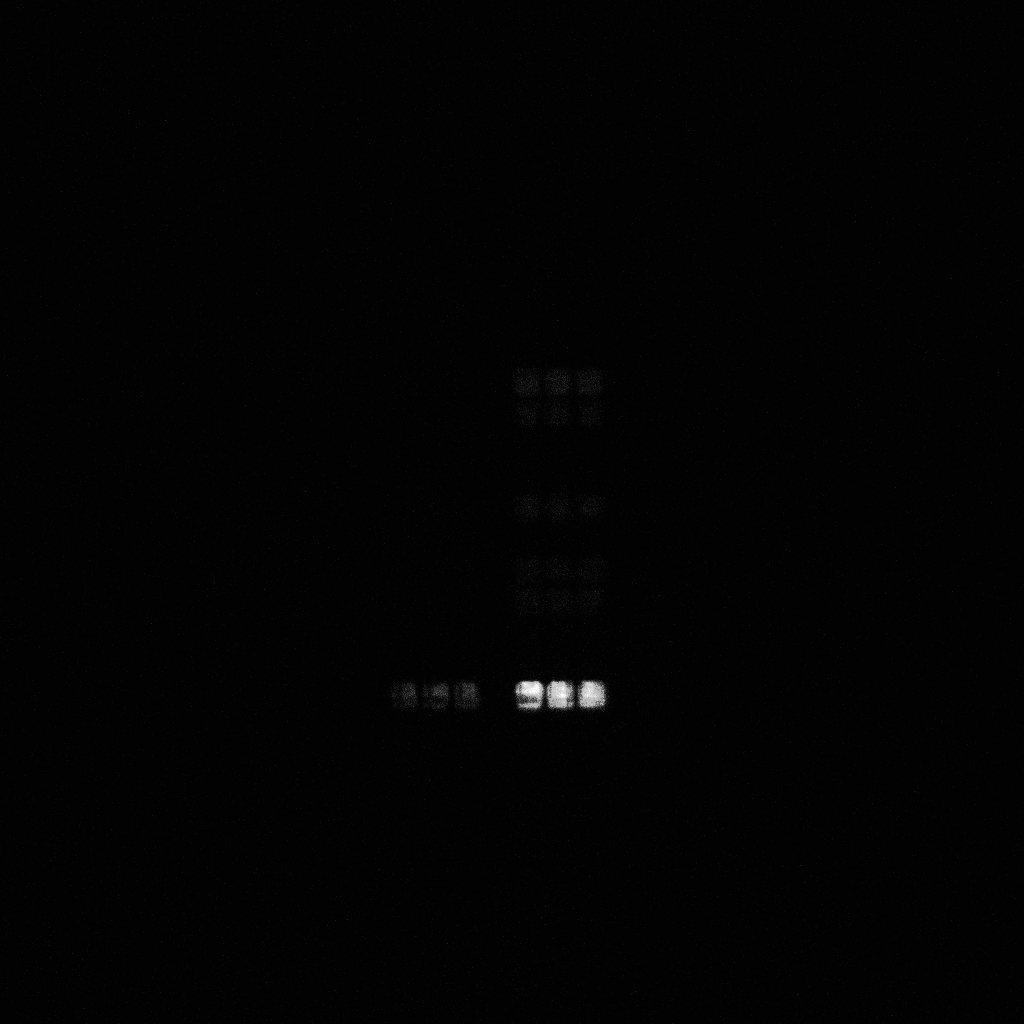

Supplement: Supplementary file 11 — Source Data [file 41467_2024_54263_MOESM11_ESM.zip › Source Data/Fig. 2/Fig. 2E/HA beads.tif]

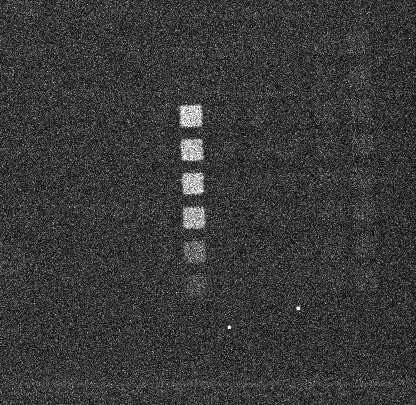

Supplement: Supplementary file 11 — Source Data [file 41467_2024_54263_MOESM11_ESM.zip › Source Data/Fig. 2/Fig. 2D/Luminescence.tif]

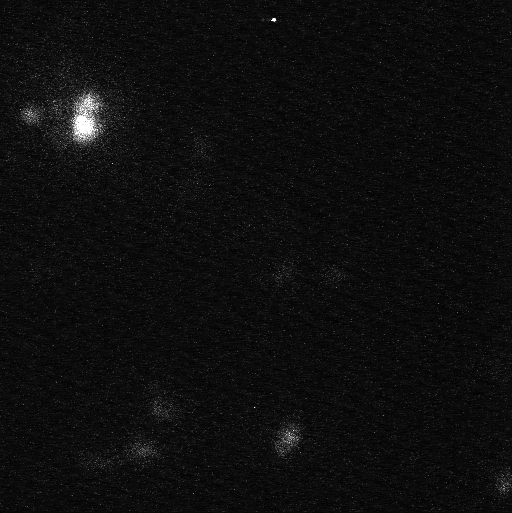

Supplement: Supplementary file 11 — Source Data [file 41467_2024_54263_MOESM11_ESM.zip › Source Data/Supplementary Fig. 10/Luminescence/lumi_90s_85f_1_0.tif]

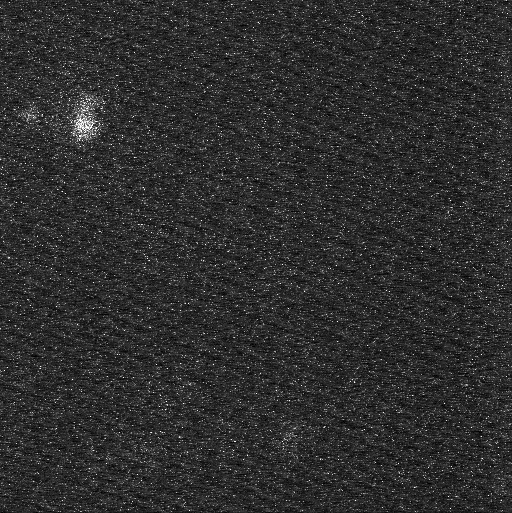

Supplement: Supplementary file 11 — Source Data [file 41467_2024_54263_MOESM11_ESM.zip › Source Data/Supplementary Fig. 10/Luminescence/lumi_90s_85f_1.tif]

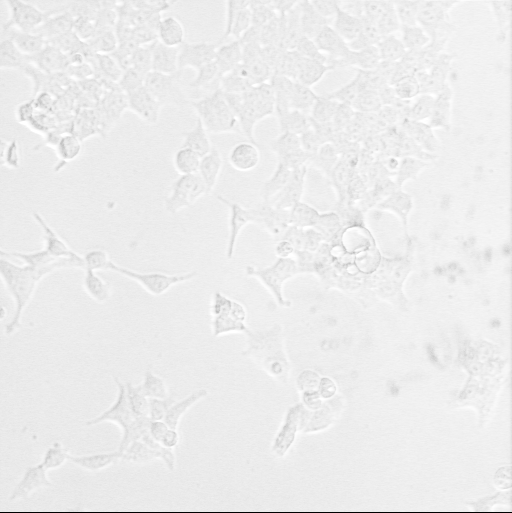

Supplement: Supplementary file 11 — Source Data [file 41467_2024_54263_MOESM11_ESM.zip › Source Data/Supplementary Fig. 10/BF/BF_20X_before.tif]

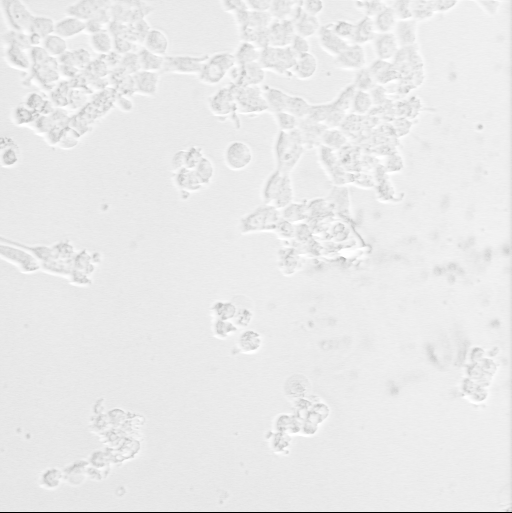

Supplement: Supplementary file 11 — Source Data [file 41467_2024_54263_MOESM11_ESM.zip › Source Data/Supplementary Fig. 10/BF/bf_after.tif]

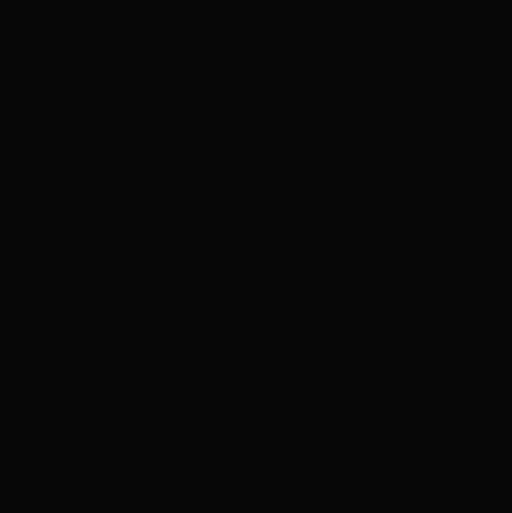

Supplement: Supplementary file 11 — Source Data [file 41467_2024_54263_MOESM11_ESM.zip › Source Data/Supplementary Fig. 10/Fluorescence /RFP_time0.tif]

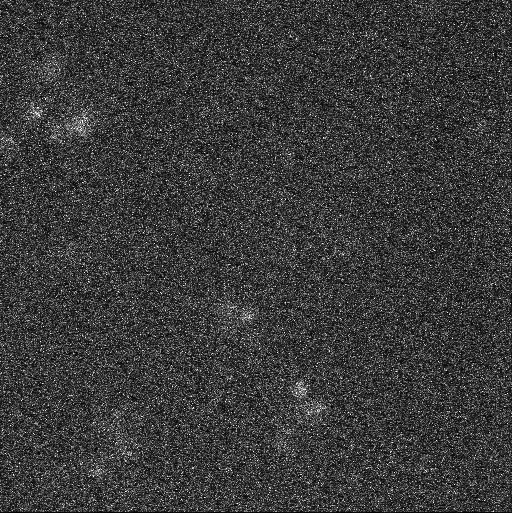

Supplement: Supplementary file 11 — Source Data [file 41467_2024_54263_MOESM11_ESM.zip › Source Data/Supplementary Fig. 10/Fluorescence /rfp_after71frames.tif]

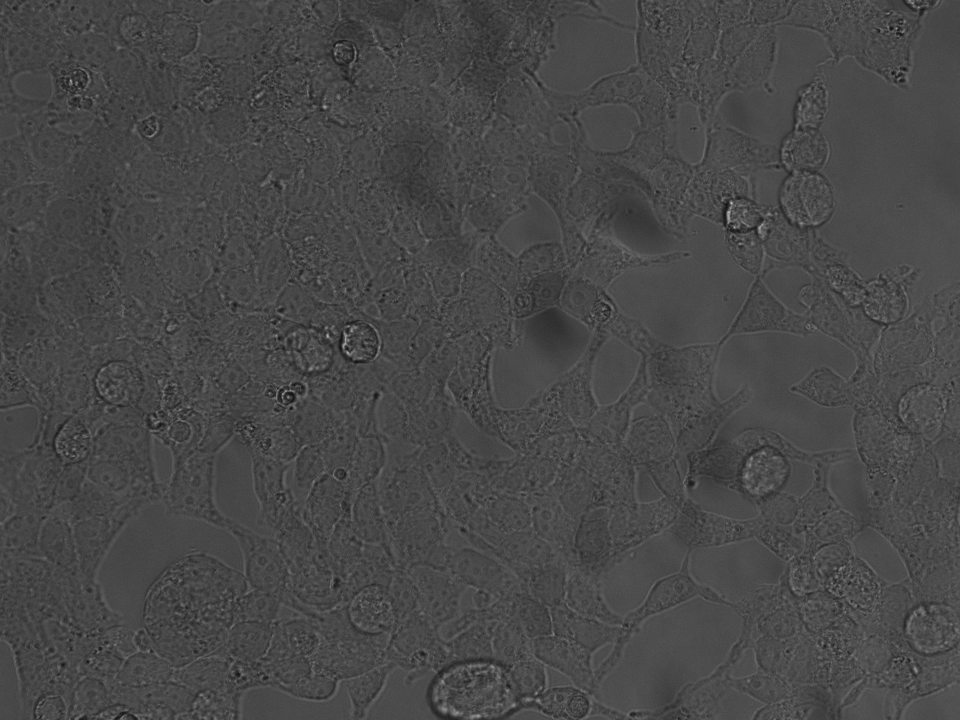

Supplement: Supplementary file 11 — Source Data [file 41467_2024_54263_MOESM11_ESM.zip › Source Data/Supplementary Fig. 12/- aresnite/BF/Image_CH4.tif]

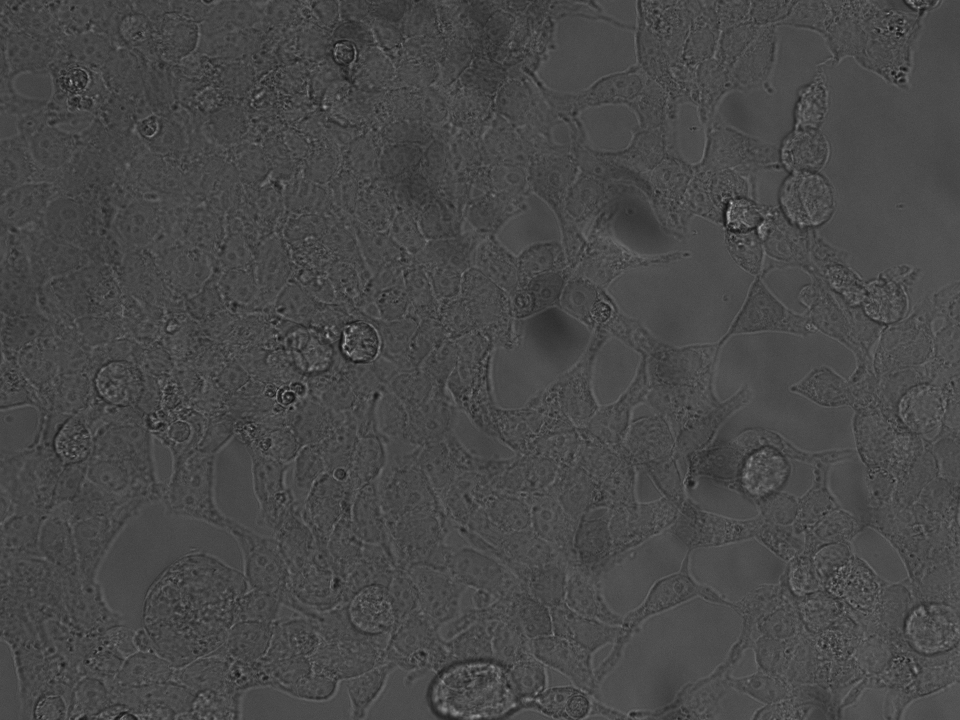

Supplement: Supplementary file 11 — Source Data [file 41467_2024_54263_MOESM11_ESM.zip › Source Data/Supplementary Fig. 12/- aresnite/BF/Image_Overlay.tif]

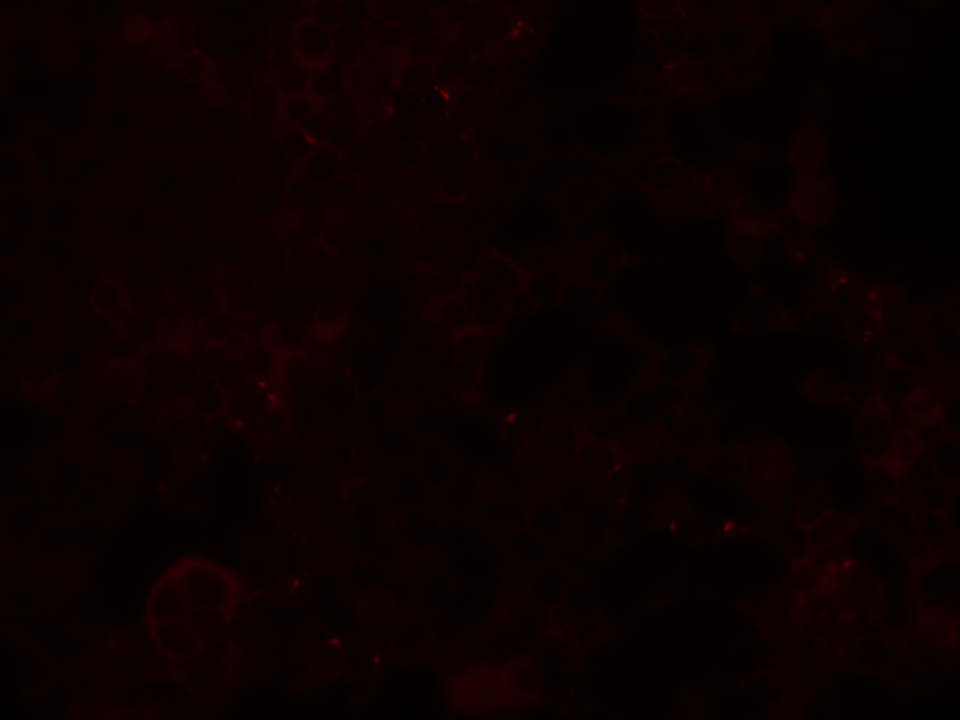

Supplement: Supplementary file 11 — Source Data [file 41467_2024_54263_MOESM11_ESM.zip › Source Data/Supplementary Fig. 12/- aresnite/Cy5 (G3BP1)/Image_CH3.tif]

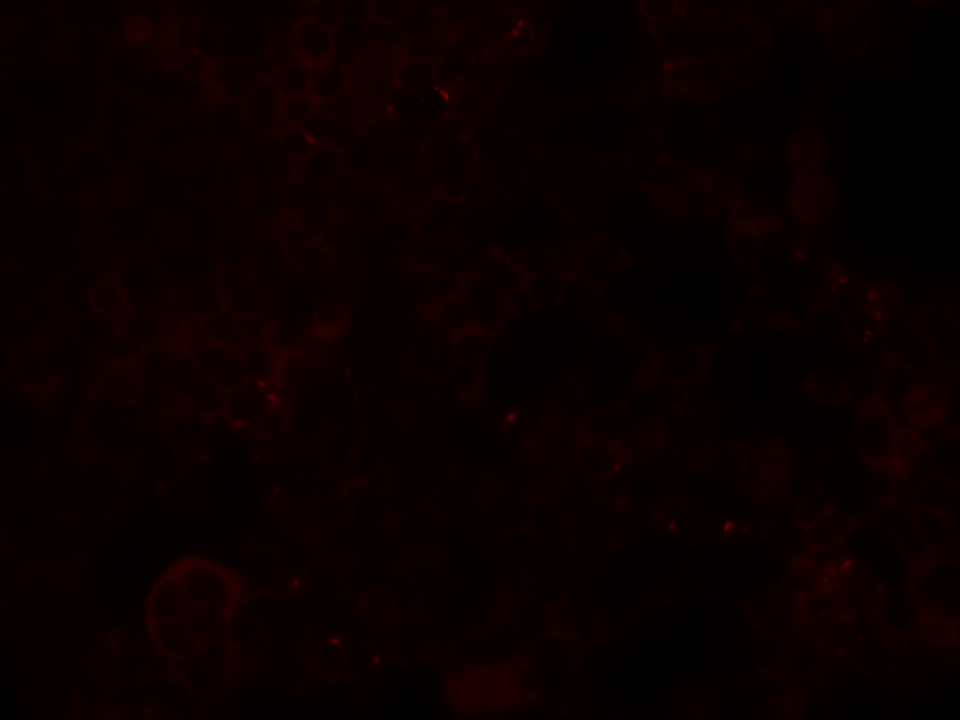

Supplement: Supplementary file 11 — Source Data [file 41467_2024_54263_MOESM11_ESM.zip › Source Data/Supplementary Fig. 12/- aresnite/Cy5 (G3BP1)/Image_Overlay.tif]

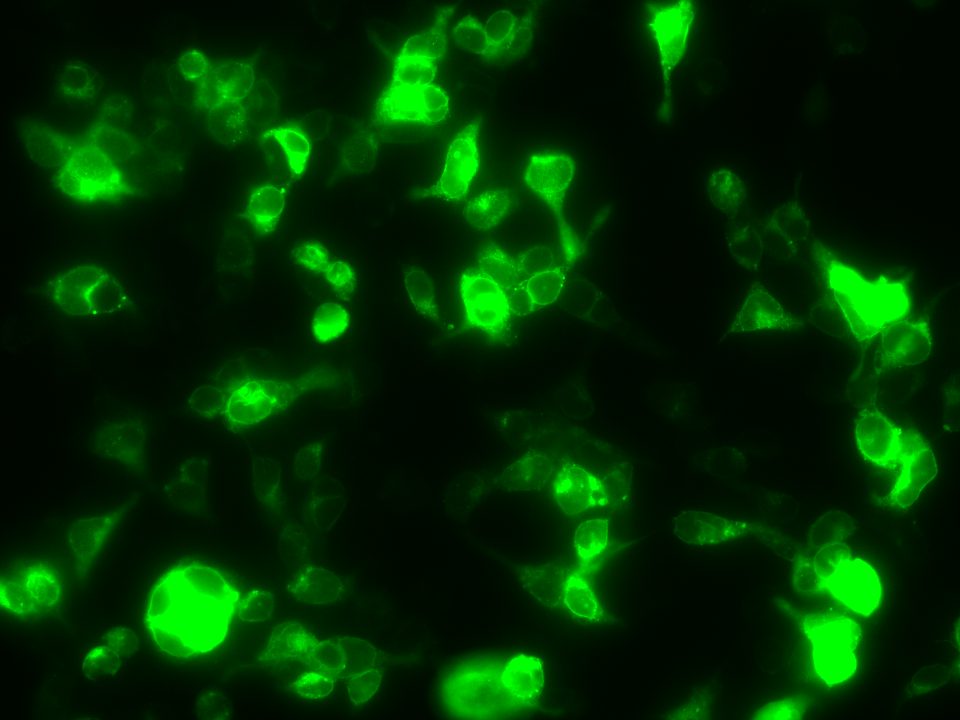

Supplement: Supplementary file 11 — Source Data [file 41467_2024_54263_MOESM11_ESM.zip › Source Data/Supplementary Fig. 12/- aresnite/GFP/Image_CH1.tif]

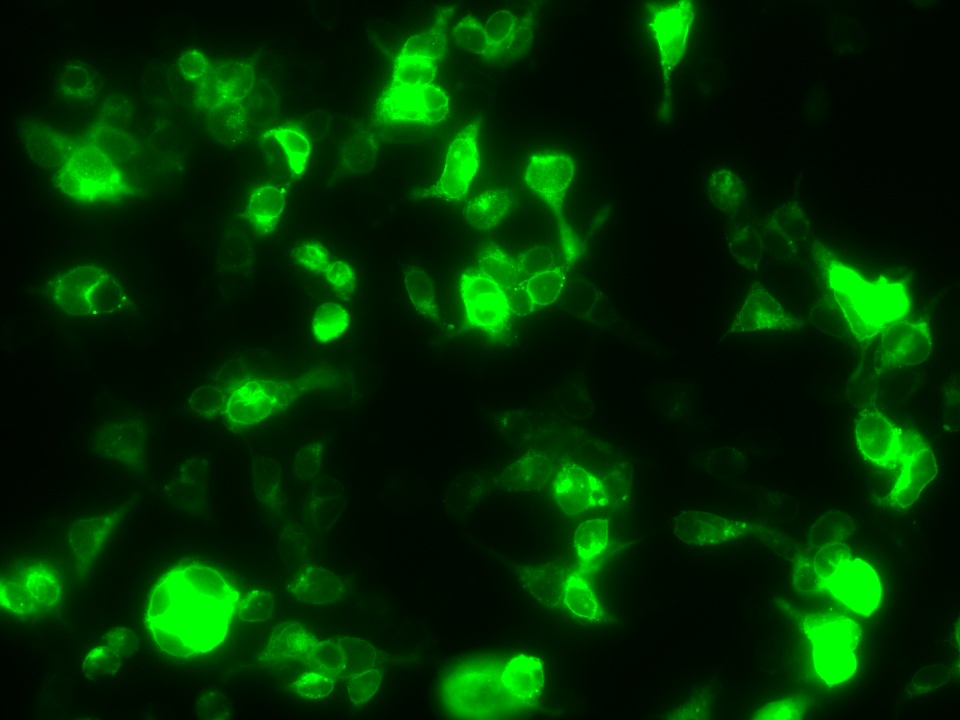

Supplement: Supplementary file 11 — Source Data [file 41467_2024_54263_MOESM11_ESM.zip › Source Data/Supplementary Fig. 12/- aresnite/GFP/Image_Overlay.tif]

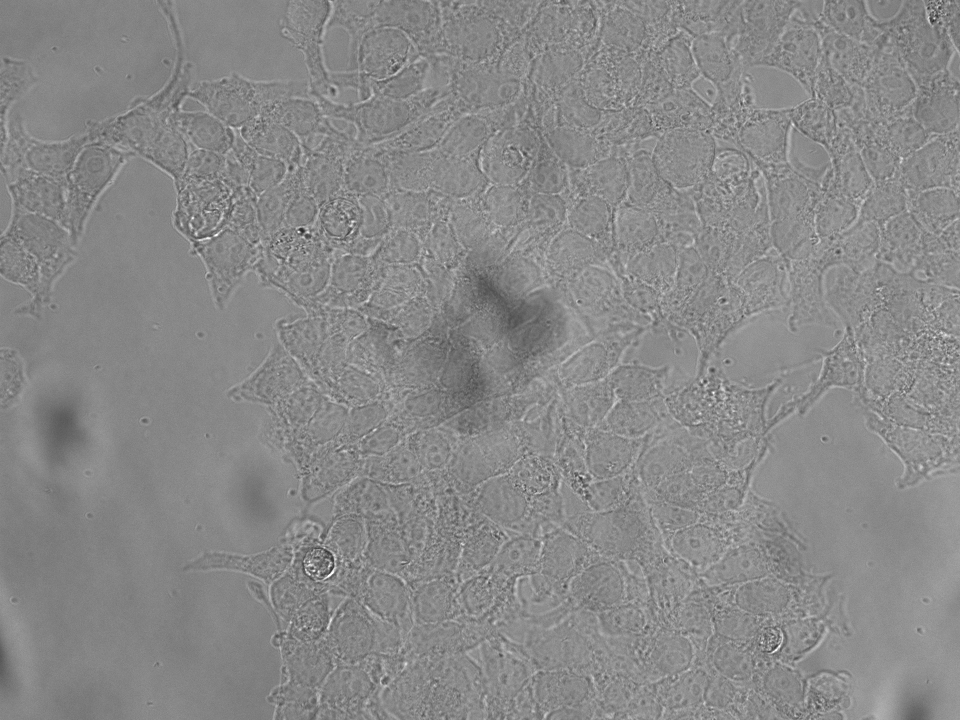

Supplement: Supplementary file 11 — Source Data [file 41467_2024_54263_MOESM11_ESM.zip › Source Data/Supplementary Fig. 12/secondary Ab only/BF/Image_CH4.tif]

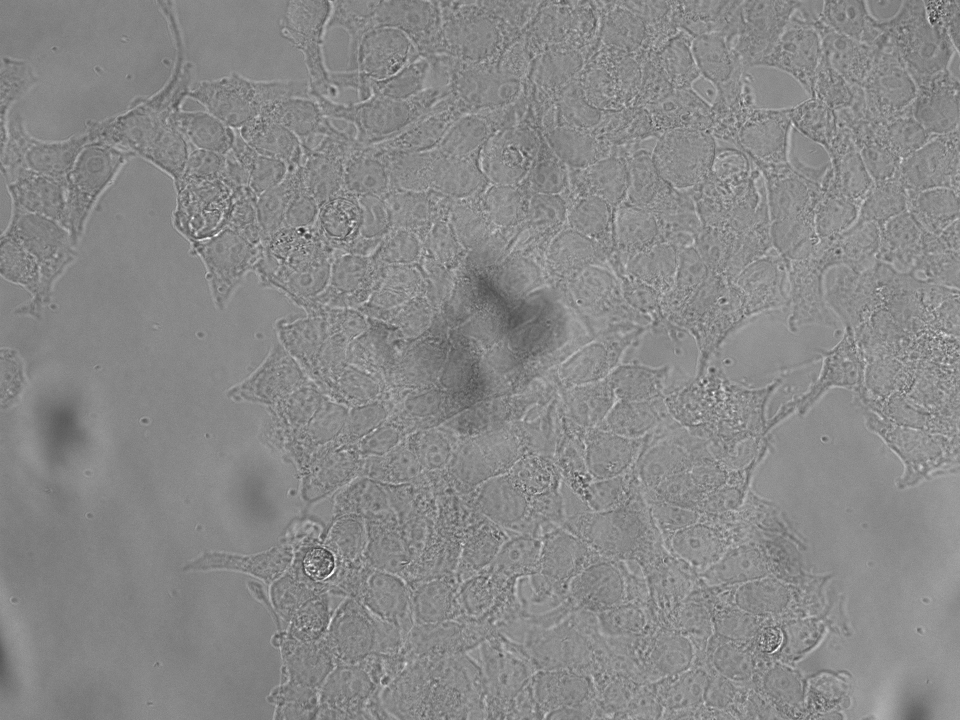

Supplement: Supplementary file 11 — Source Data [file 41467_2024_54263_MOESM11_ESM.zip › Source Data/Supplementary Fig. 12/secondary Ab only/BF/Image_Overlay.tif]

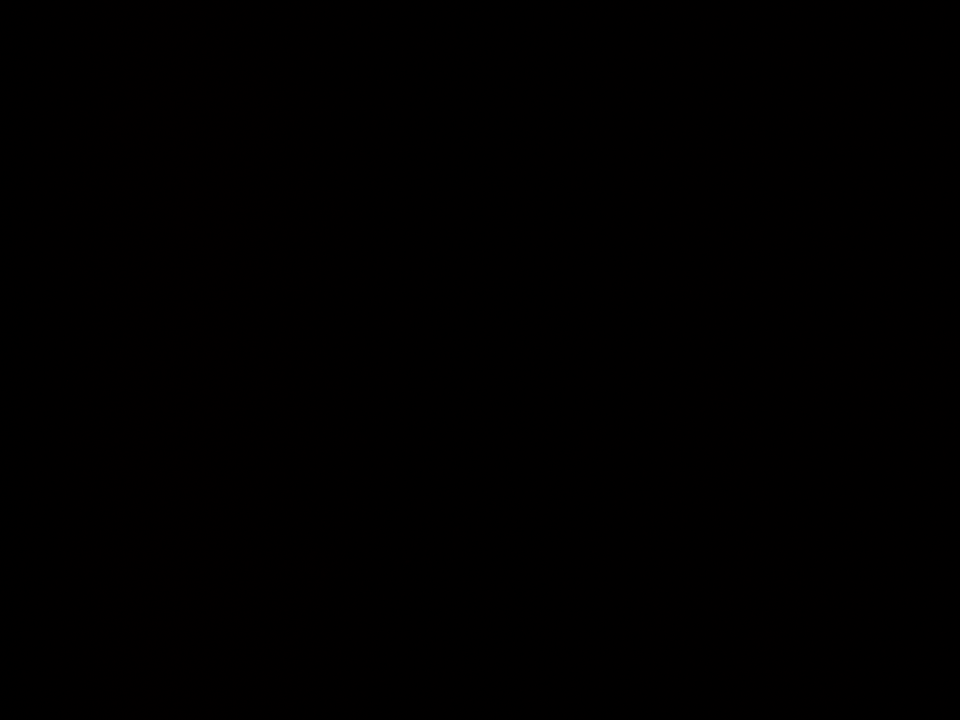

Supplement: Supplementary file 11 — Source Data [file 41467_2024_54263_MOESM11_ESM.zip › Source Data/Supplementary Fig. 12/secondary Ab only/Cy5 (G3BP1)/Image_CH3.tif]

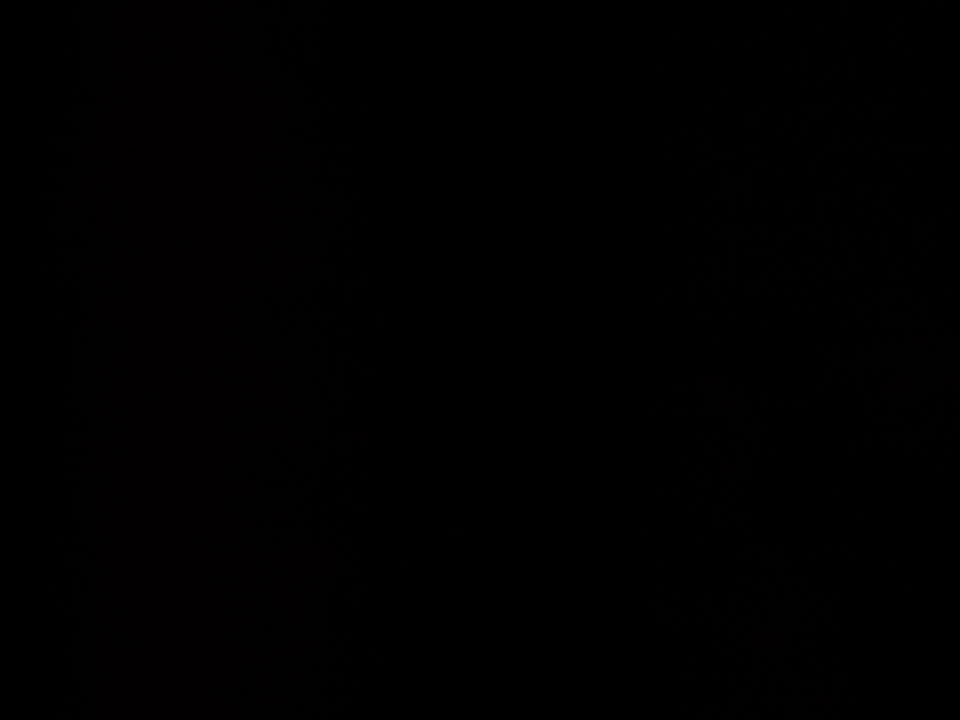

Supplement: Supplementary file 11 — Source Data [file 41467_2024_54263_MOESM11_ESM.zip › Source Data/Supplementary Fig. 12/secondary Ab only/Cy5 (G3BP1)/Image_Overlay.tif]

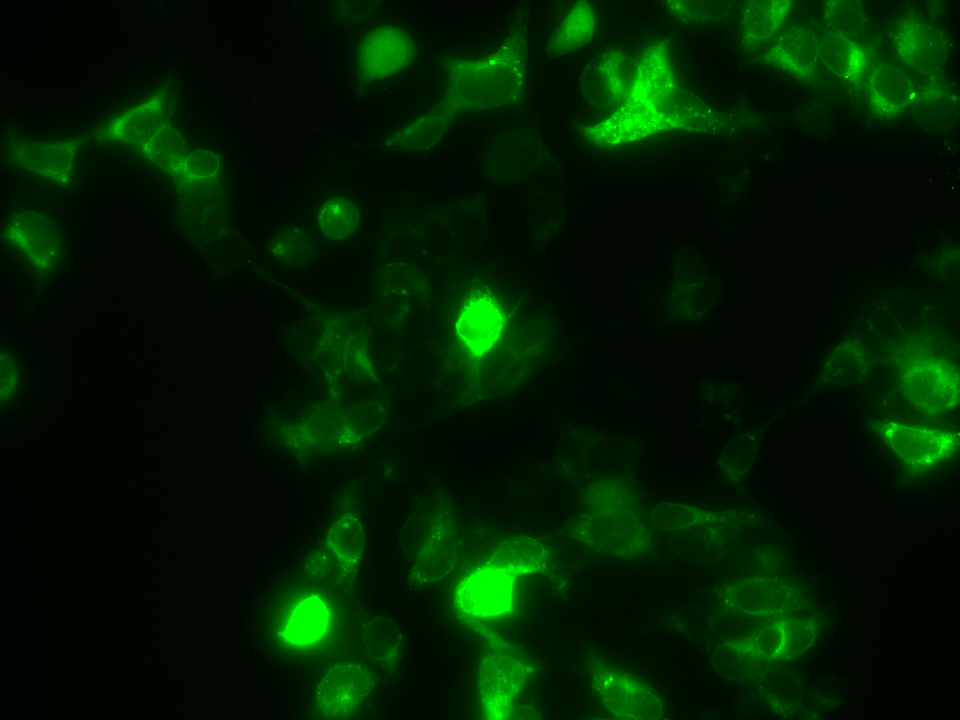

Supplement: Supplementary file 11 — Source Data [file 41467_2024_54263_MOESM11_ESM.zip › Source Data/Supplementary Fig. 12/secondary Ab only/GFP/Image_CH1.tif]

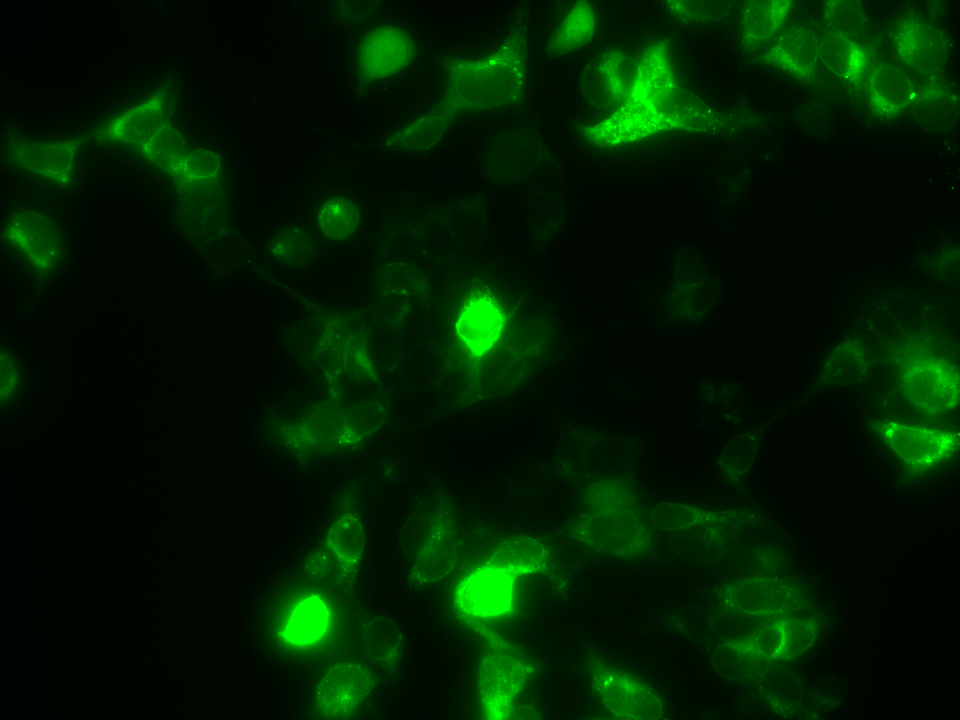

Supplement: Supplementary file 11 — Source Data [file 41467_2024_54263_MOESM11_ESM.zip › Source Data/Supplementary Fig. 12/secondary Ab only/GFP/Image_Overlay.tif]

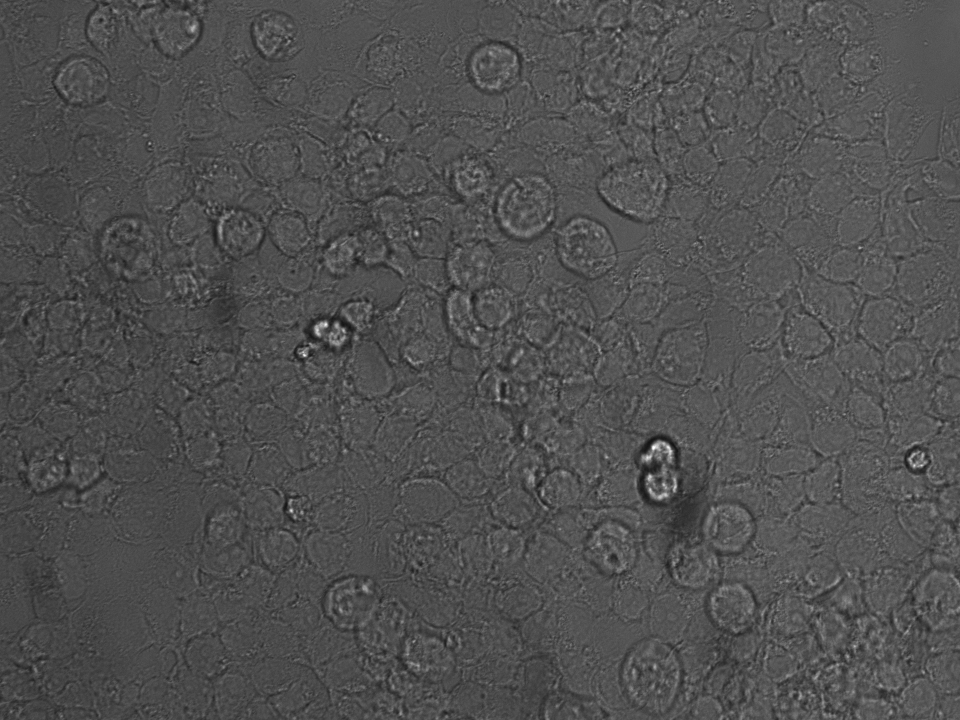

Supplement: Supplementary file 11 — Source Data [file 41467_2024_54263_MOESM11_ESM.zip › Source Data/Supplementary Fig. 12/+ arsenite/BF/Image_CH4.tif]

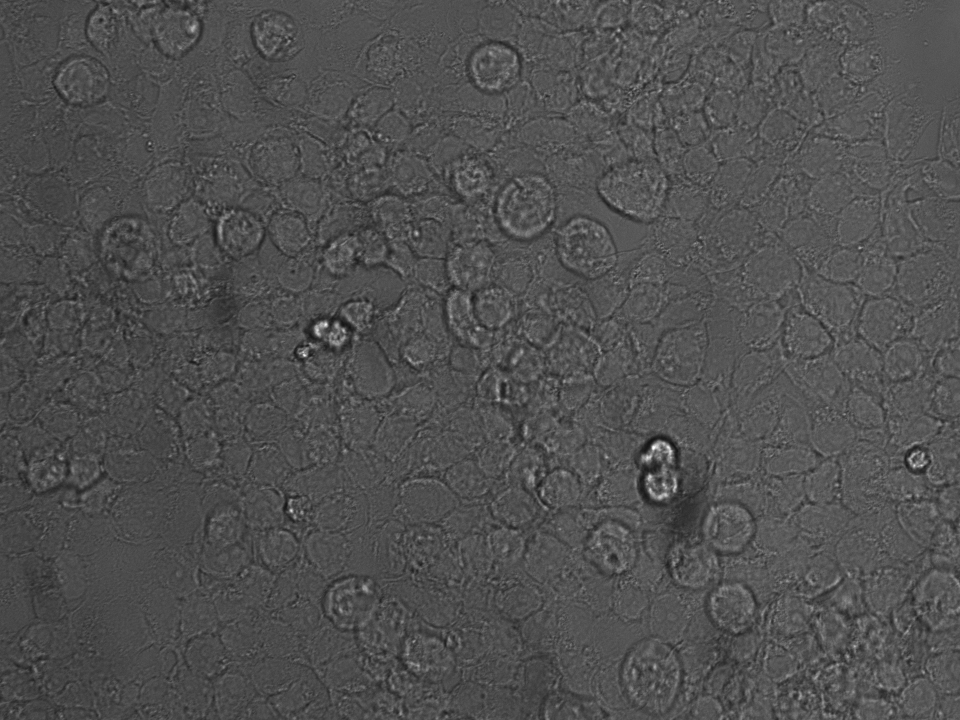

Supplement: Supplementary file 11 — Source Data [file 41467_2024_54263_MOESM11_ESM.zip › Source Data/Supplementary Fig. 12/+ arsenite/BF/Image_Overlay.tif]

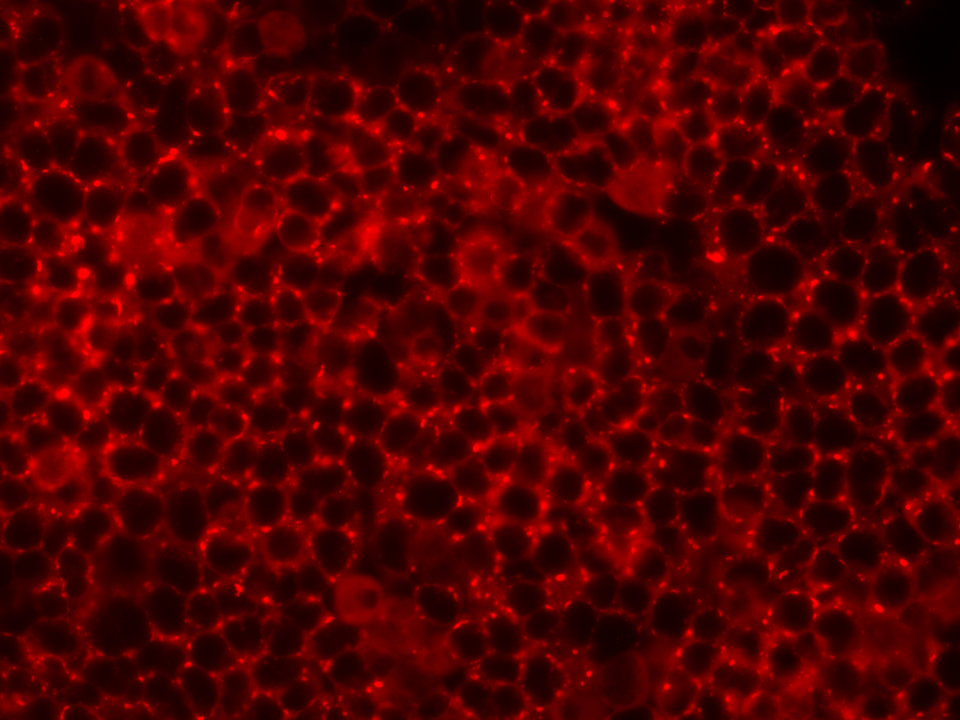

Supplement: Supplementary file 11 — Source Data [file 41467_2024_54263_MOESM11_ESM.zip › Source Data/Supplementary Fig. 12/+ arsenite/Cy5 (G3BP1)/Image_CH3.tif]

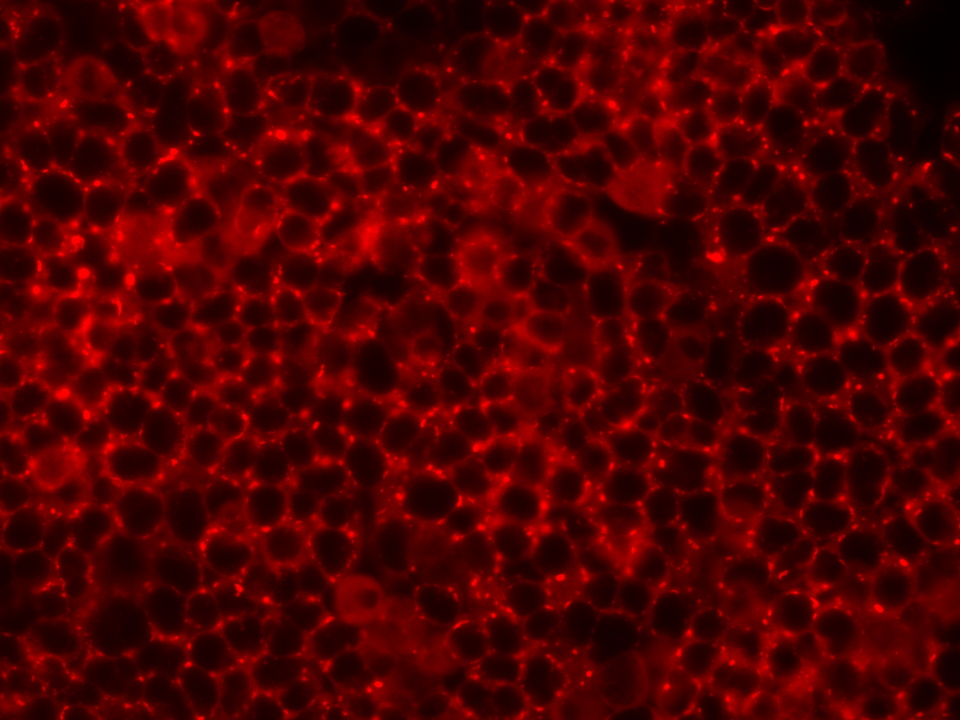

Supplement: Supplementary file 11 — Source Data [file 41467_2024_54263_MOESM11_ESM.zip › Source Data/Supplementary Fig. 12/+ arsenite/Cy5 (G3BP1)/Image_Overlay.tif]

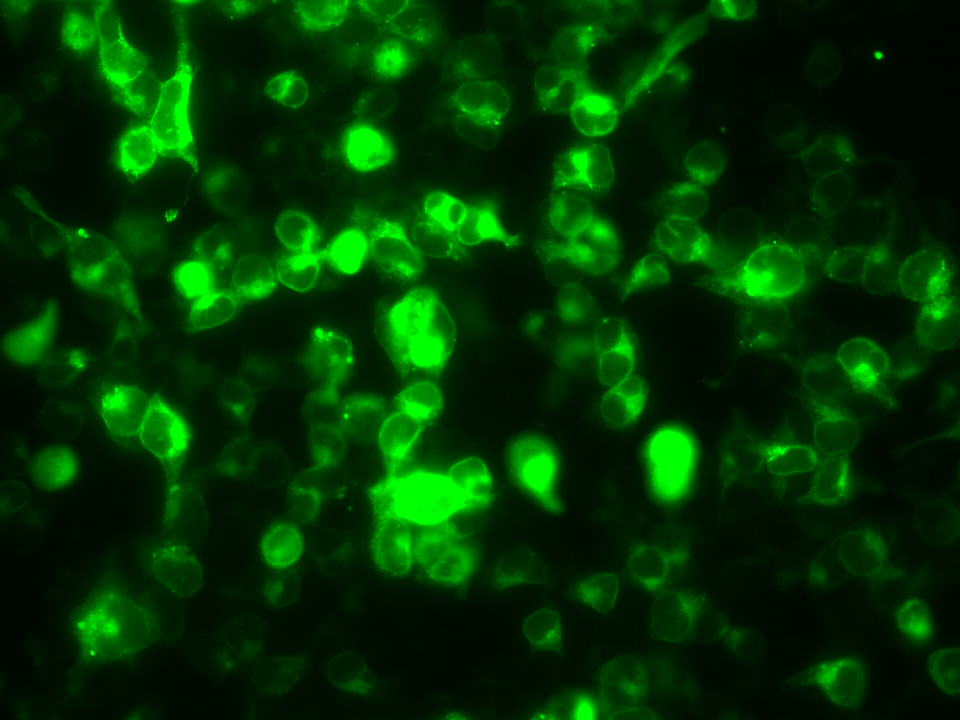

Supplement: Supplementary file 11 — Source Data [file 41467_2024_54263_MOESM11_ESM.zip › Source Data/Supplementary Fig. 12/+ arsenite/GFP/Image_CH1.tif]

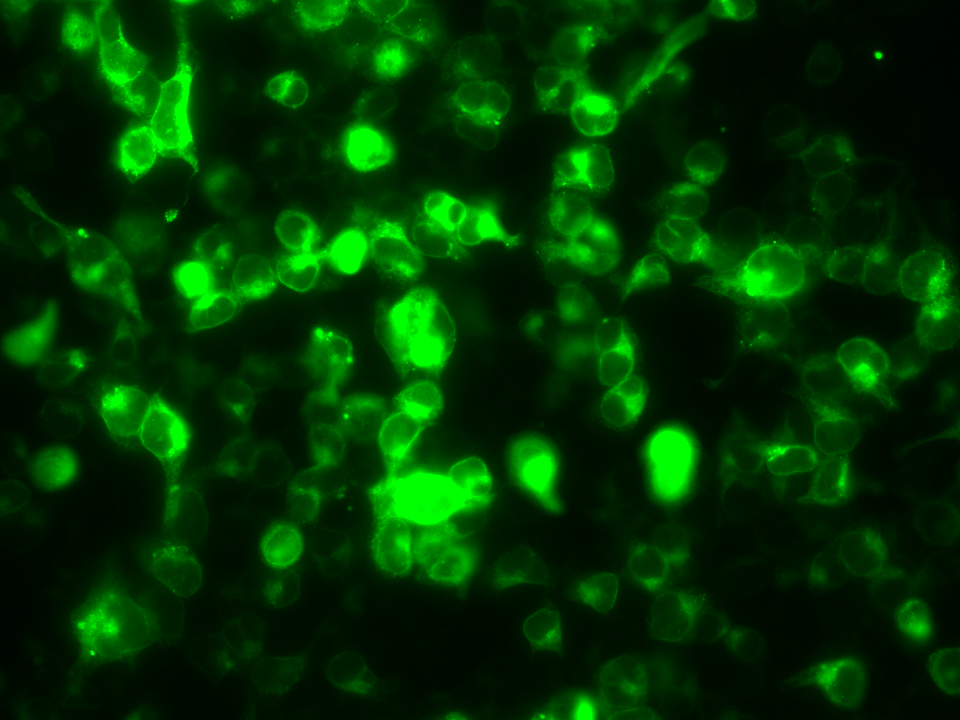

Supplement: Supplementary file 11 — Source Data [file 41467_2024_54263_MOESM11_ESM.zip › Source Data/Supplementary Fig. 12/+ arsenite/GFP/Image_Overlay.tif]

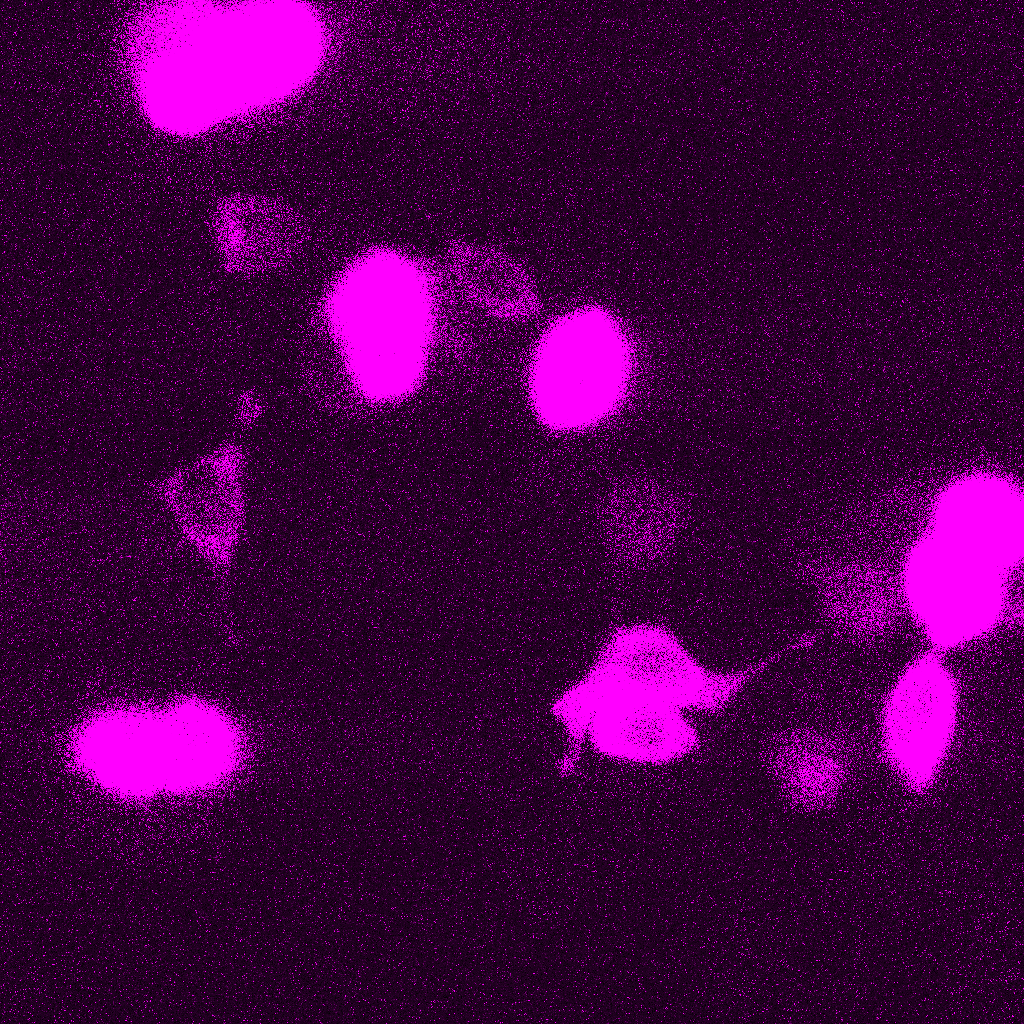

Supplement: Supplementary file 11 — Source Data [file 41467_2024_54263_MOESM11_ESM.zip › Source Data/Supplementary Fig. 7/Fig. 7A and B/Experimental/thresholded lumi.gif]

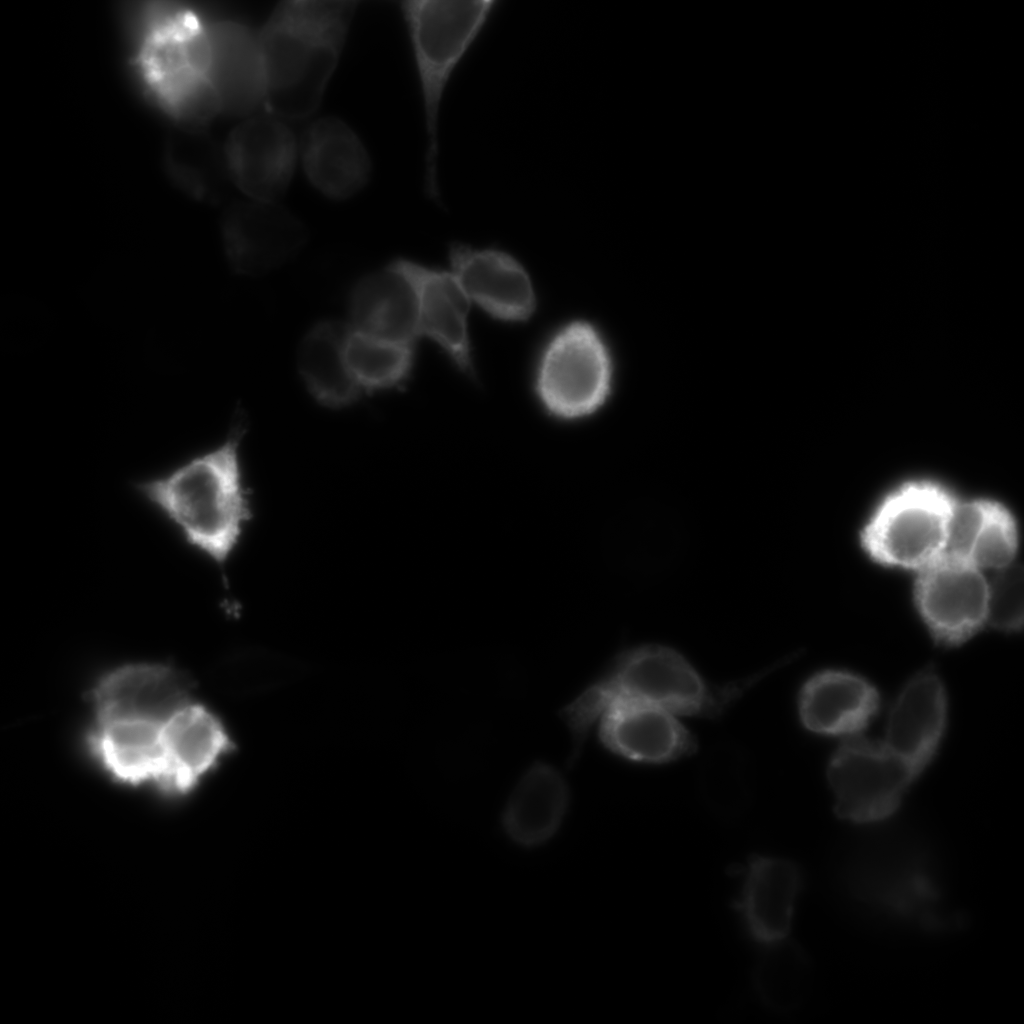

Supplement: Supplementary file 11 — Source Data [file 41467_2024_54263_MOESM11_ESM.zip › Source Data/Supplementary Fig. 7/Fig. 7A and B/Experimental/AVG_Stack-StayGold-normalized.tif]

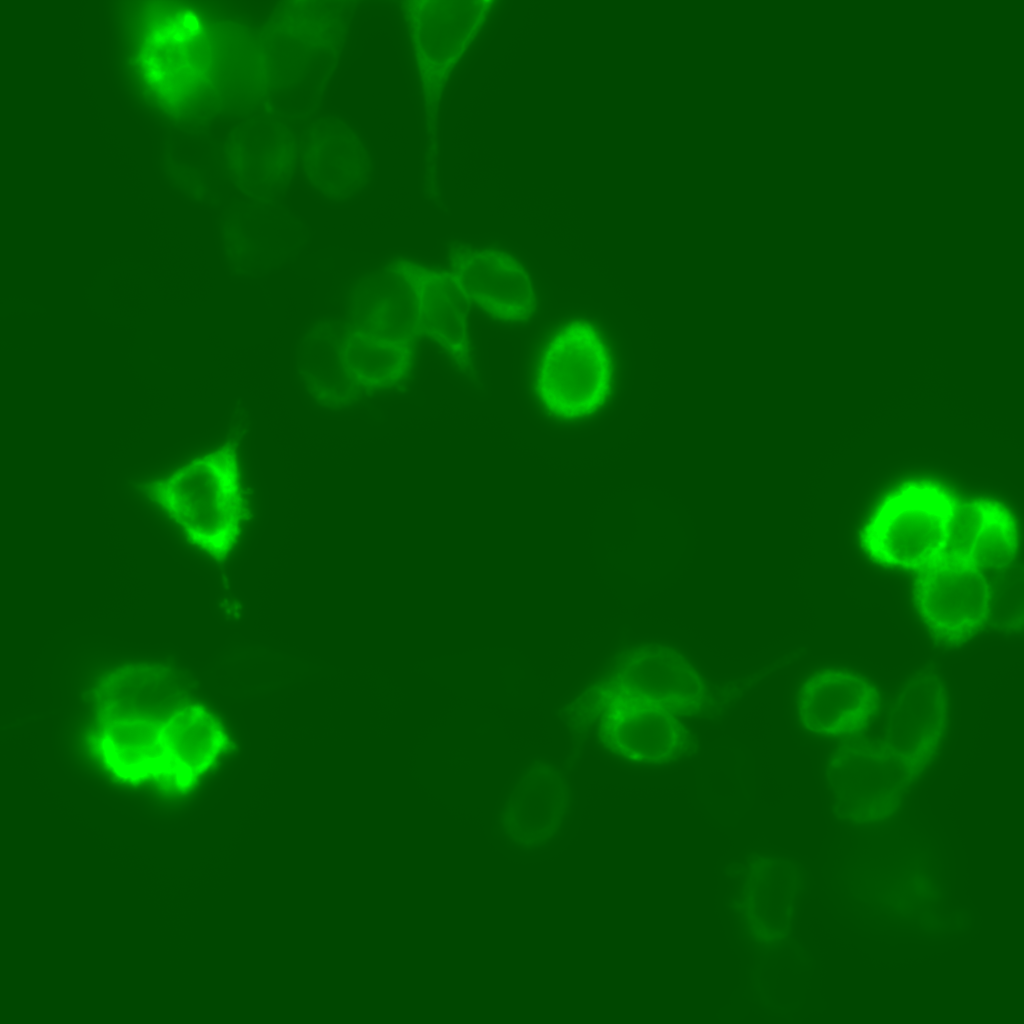

Supplement: Supplementary file 11 — Source Data [file 41467_2024_54263_MOESM11_ESM.zip › Source Data/Supplementary Fig. 7/Fig. 7A and B/Experimental/thresholdgfp.gif]

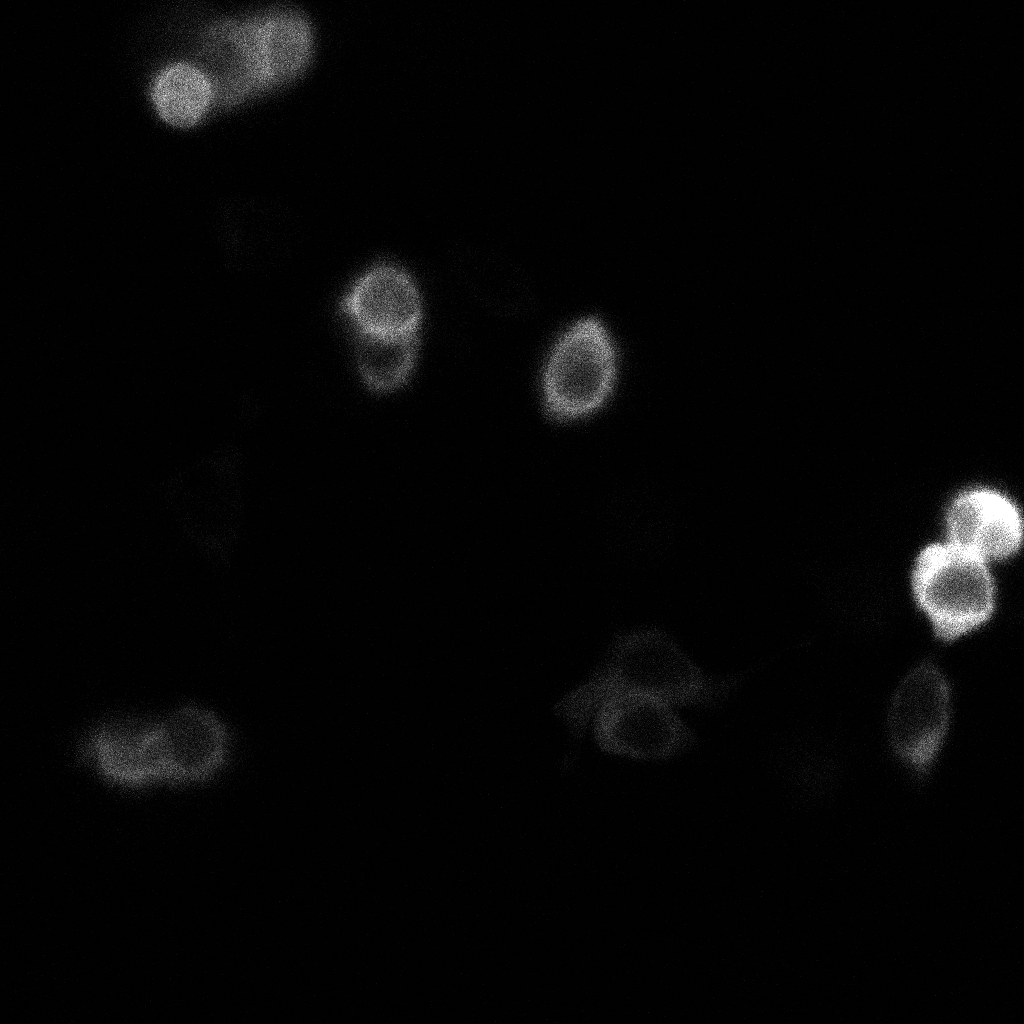

Supplement: Supplementary file 11 — Source Data [file 41467_2024_54263_MOESM11_ESM.zip › Source Data/Supplementary Fig. 7/Fig. 7A and B/Experimental/Lumi_180s_2_X3-normalized.tif]

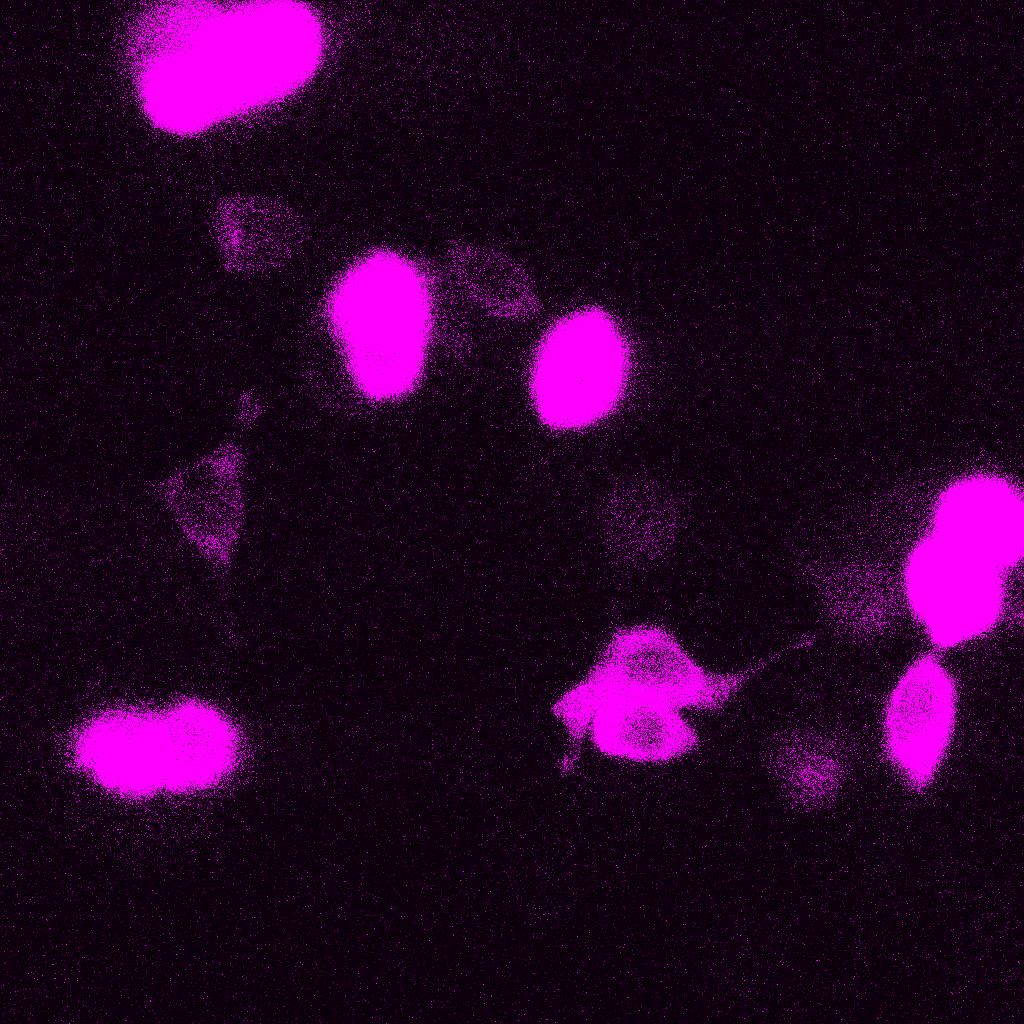

Supplement: Supplementary file 11 — Source Data [file 41467_2024_54263_MOESM11_ESM.zip › Source Data/Supplementary Fig. 7/Fig. 7A and B/Experimental/thresholded lumi_2.gif]

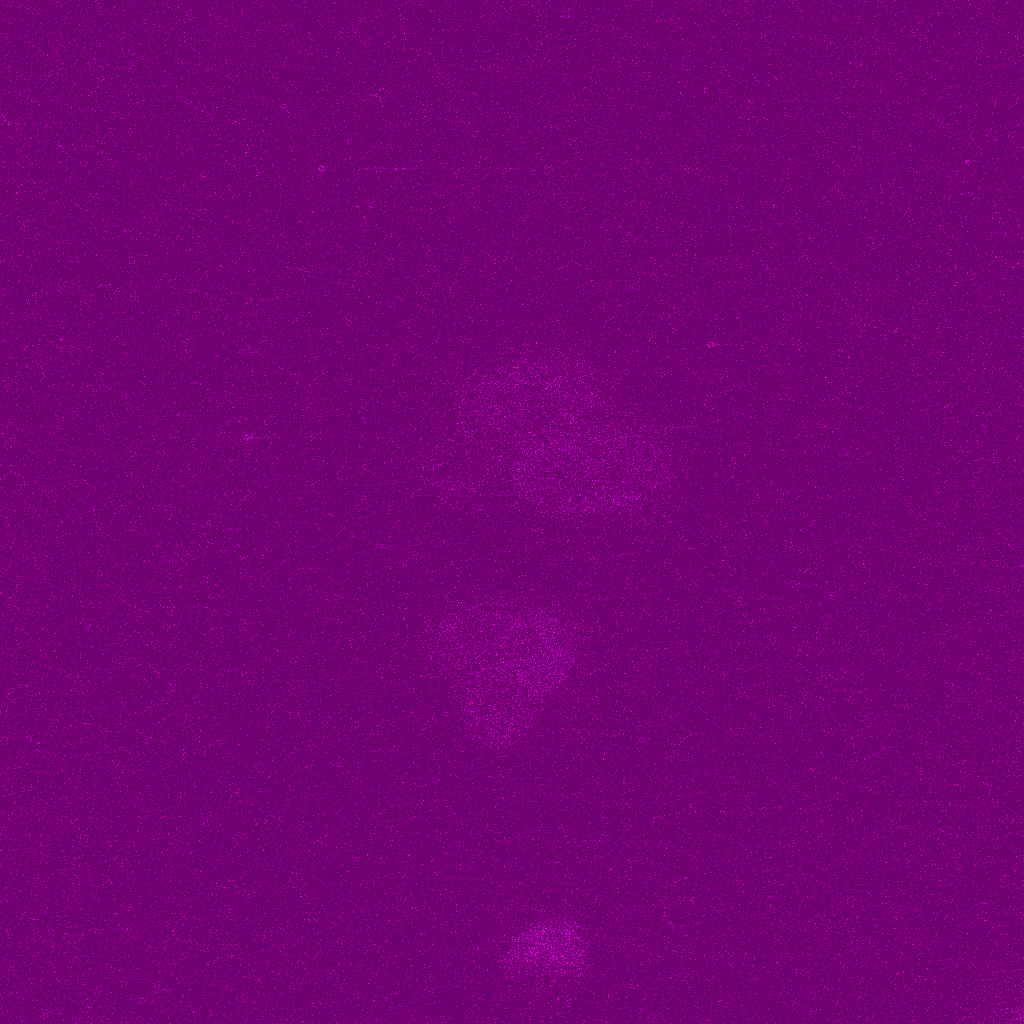

Supplement: Supplementary file 11 — Source Data [file 41467_2024_54263_MOESM11_ESM.zip › Source Data/Supplementary Fig. 7/Fig. 7A and B/Control/thresholded lumi.gif]

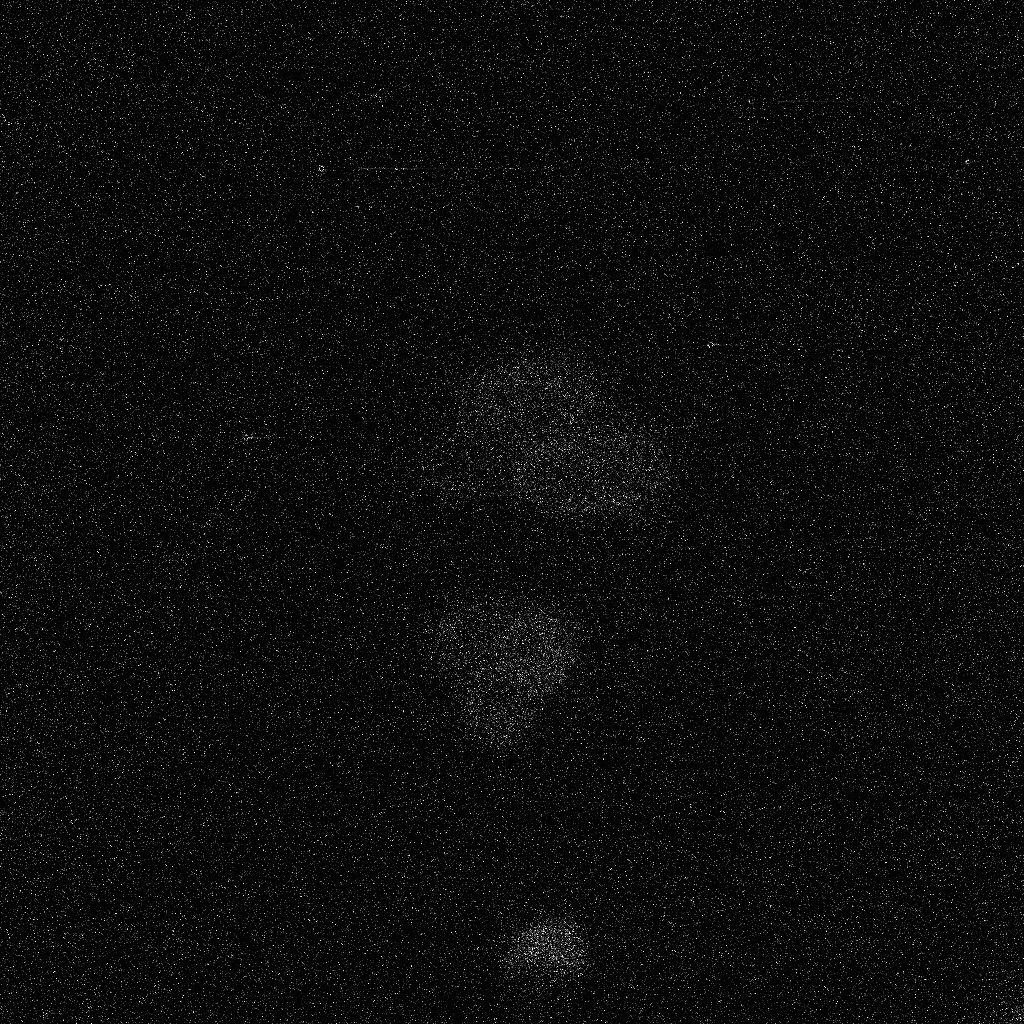

Supplement: Supplementary file 11 — Source Data [file 41467_2024_54263_MOESM11_ESM.zip › Source Data/Supplementary Fig. 7/Fig. 7A and B/Control/AVG_Stack_Lumi-outliersremoved.tif]

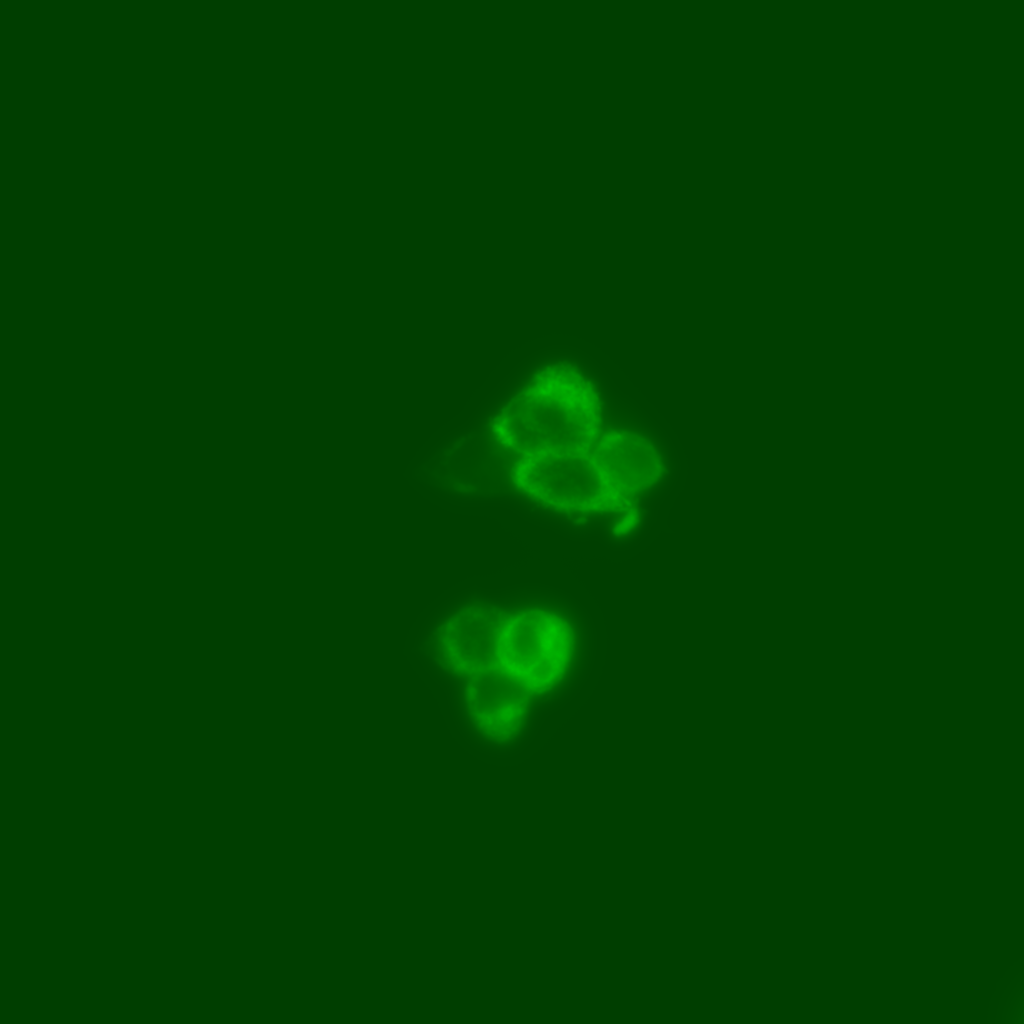

Supplement: Supplementary file 11 — Source Data [file 41467_2024_54263_MOESM11_ESM.zip › Source Data/Supplementary Fig. 7/Fig. 7A and B/Control/thresholdgfp.gif]

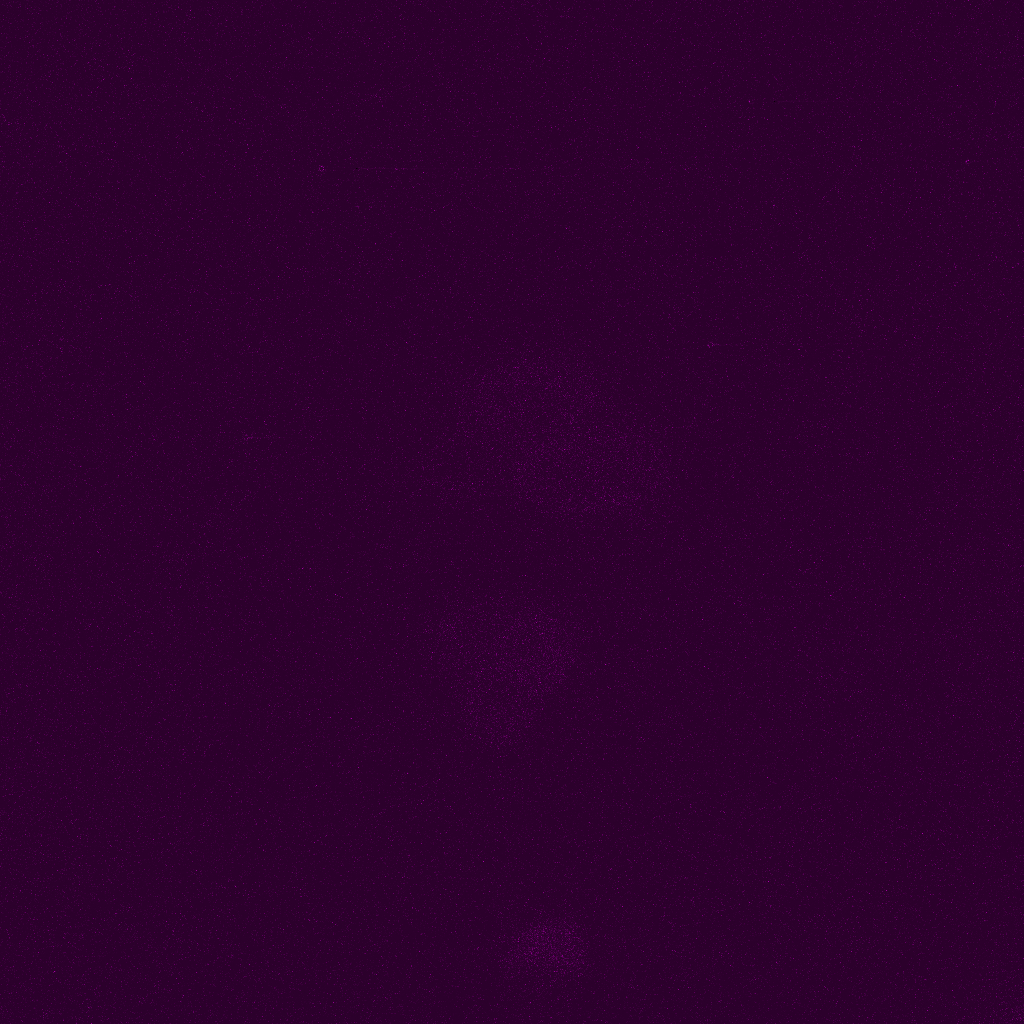

Supplement: Supplementary file 11 — Source Data [file 41467_2024_54263_MOESM11_ESM.zip › Source Data/Supplementary Fig. 7/Fig. 7A and B/Control/thresholded lumi_2.gif]

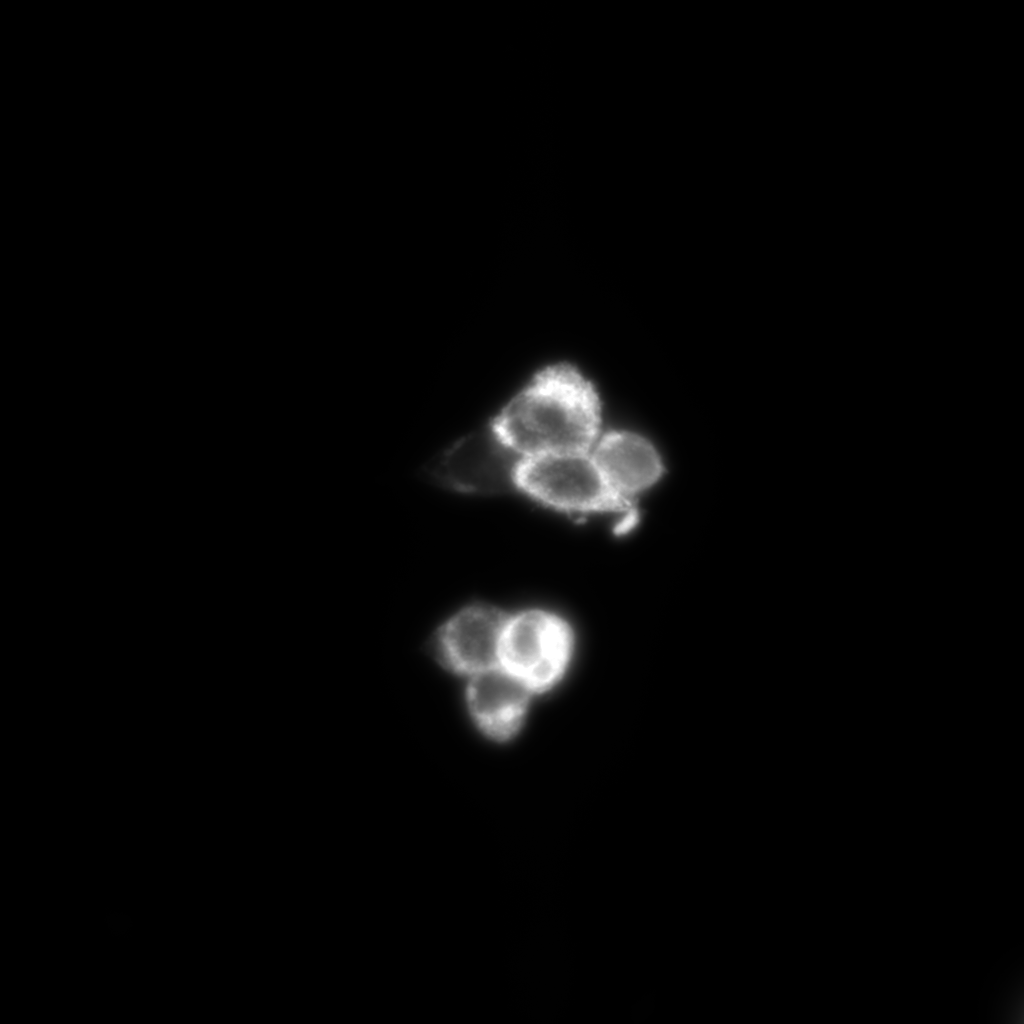

Supplement: Supplementary file 11 — Source Data [file 41467_2024_54263_MOESM11_ESM.zip › Source Data/Supplementary Fig. 7/Fig. 7A and B/Control/AVG_Stack_StayGold.tif]

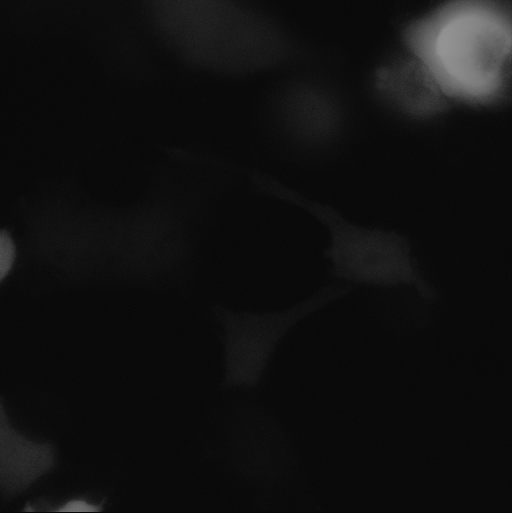

Supplement: Supplementary file 11 — Source Data [file 41467_2024_54263_MOESM11_ESM.zip › Source Data/Supplementary Fig. 11/Fluorescence /mCherry_after stress/mcherry_after_0.tif]

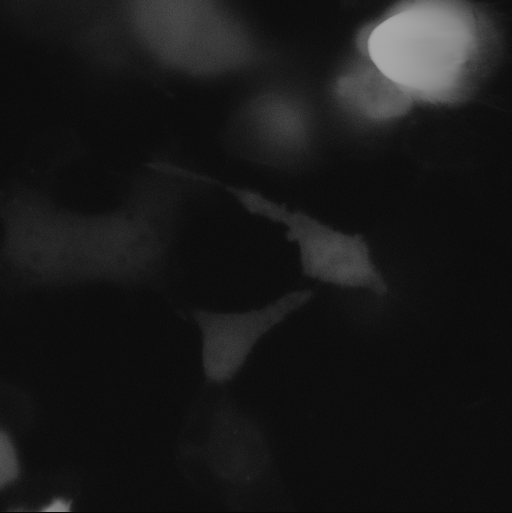

Supplement: Supplementary file 11 — Source Data [file 41467_2024_54263_MOESM11_ESM.zip › Source Data/Supplementary Fig. 11/Fluorescence /mCherry_after stress/mcherryafter_2.tif]

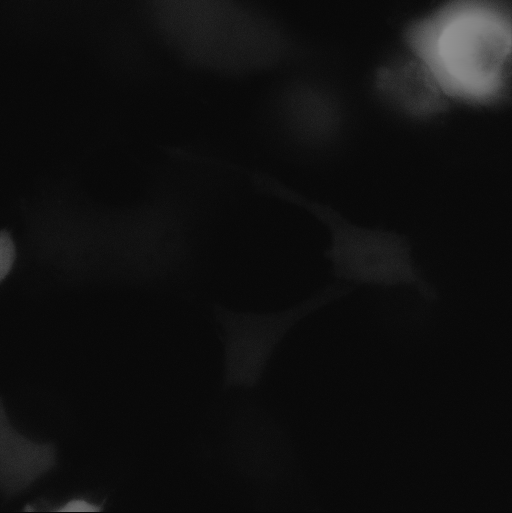

Supplement: Supplementary file 11 — Source Data [file 41467_2024_54263_MOESM11_ESM.zip › Source Data/Supplementary Fig. 11/Fluorescence /mCherry_after stress/mcherry_after.tif]

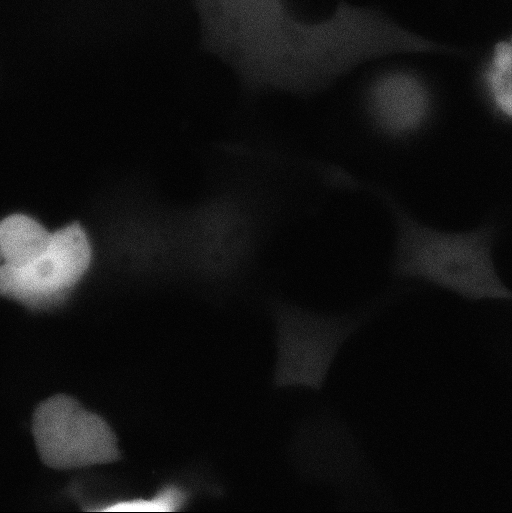

Supplement: Supplementary file 11 — Source Data [file 41467_2024_54263_MOESM11_ESM.zip › Source Data/Supplementary Fig. 11/Fluorescence /mCherry_before stress/mCherry_before.tif]

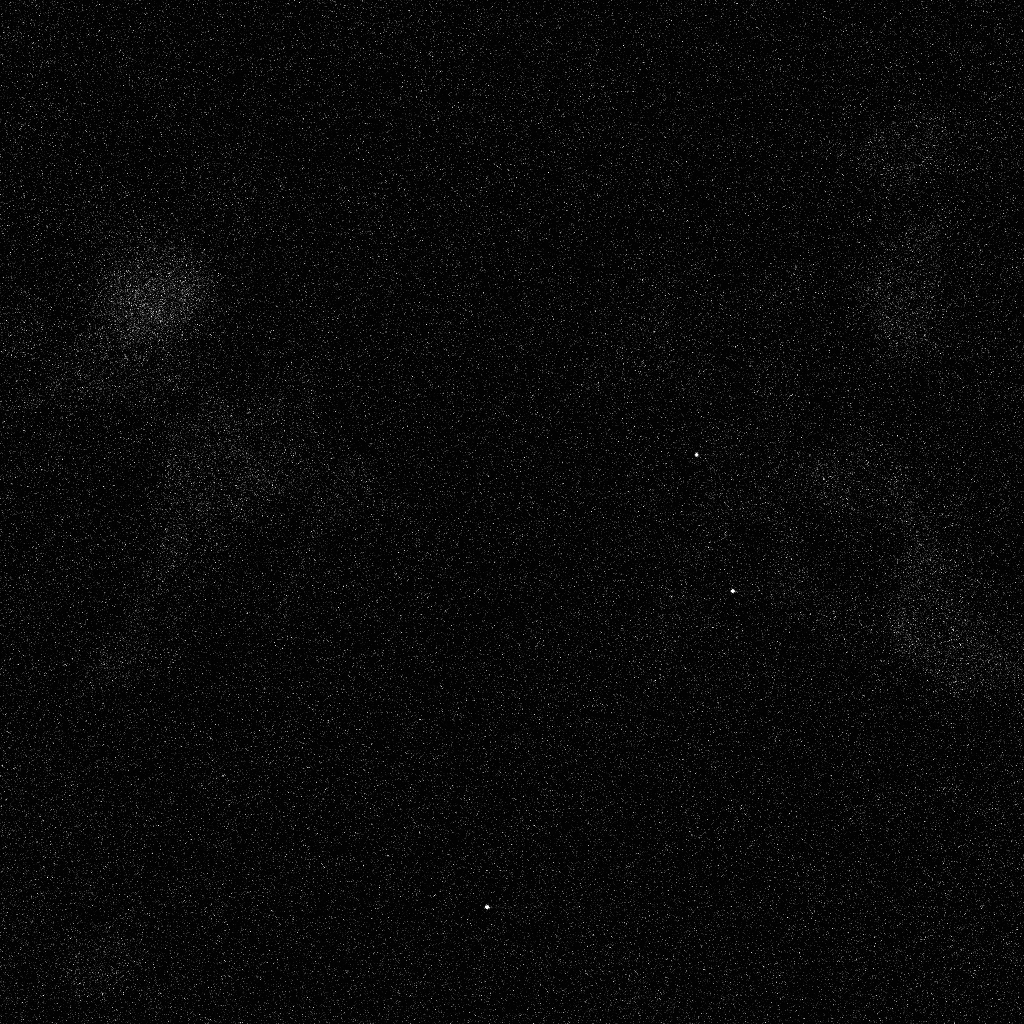

Supplement: Supplementary file 11 — Source Data [file 41467_2024_54263_MOESM11_ESM.zip › Source Data/Fig. 5/Fig. 5 B/Stable probe HEK + 300 probe + 100 staygoldm3p/lumi/Lumi180s_1_X1.tif]

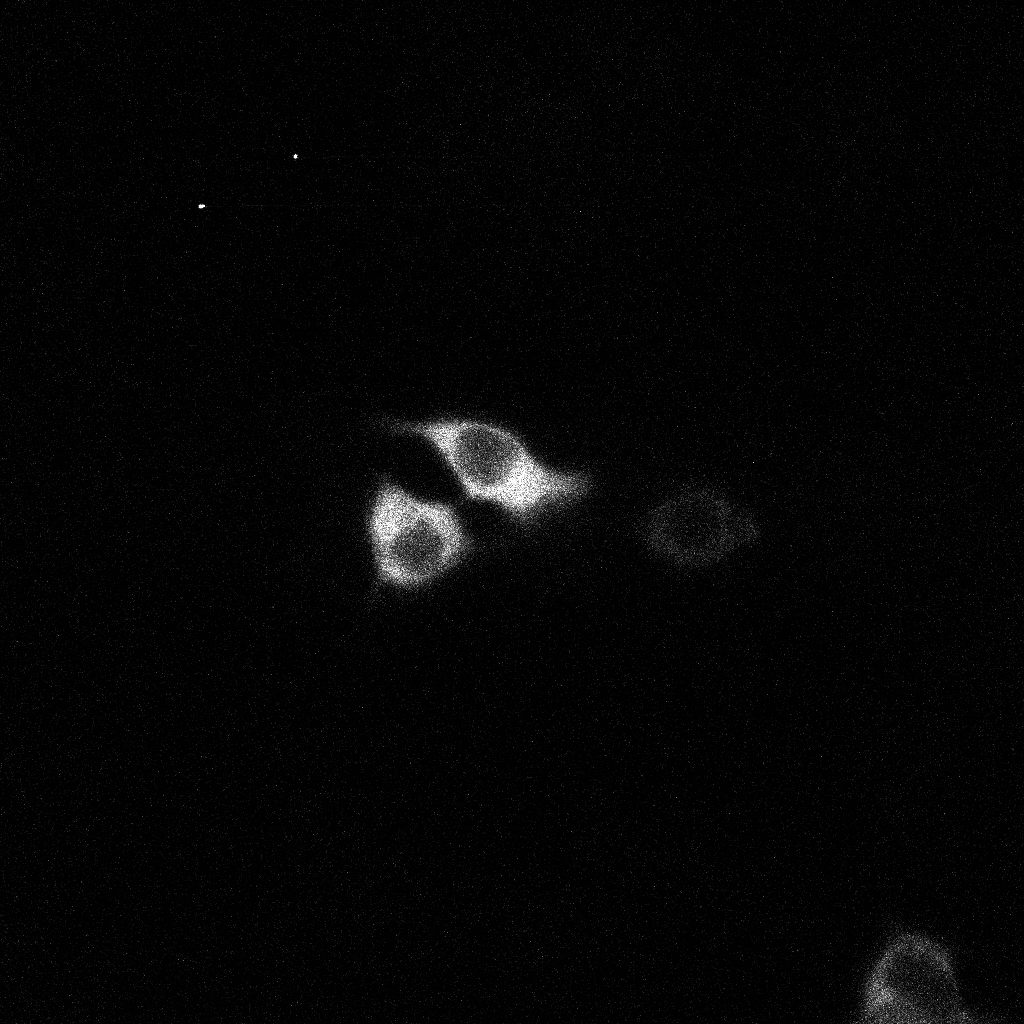

Supplement: Supplementary file 11 — Source Data [file 41467_2024_54263_MOESM11_ESM.zip › Source Data/Fig. 5/Fig. 5 B/Stable probe HEK + 300 probe + 100 staygoldm3p/lumi/Lumi60s_1_X1.tif]

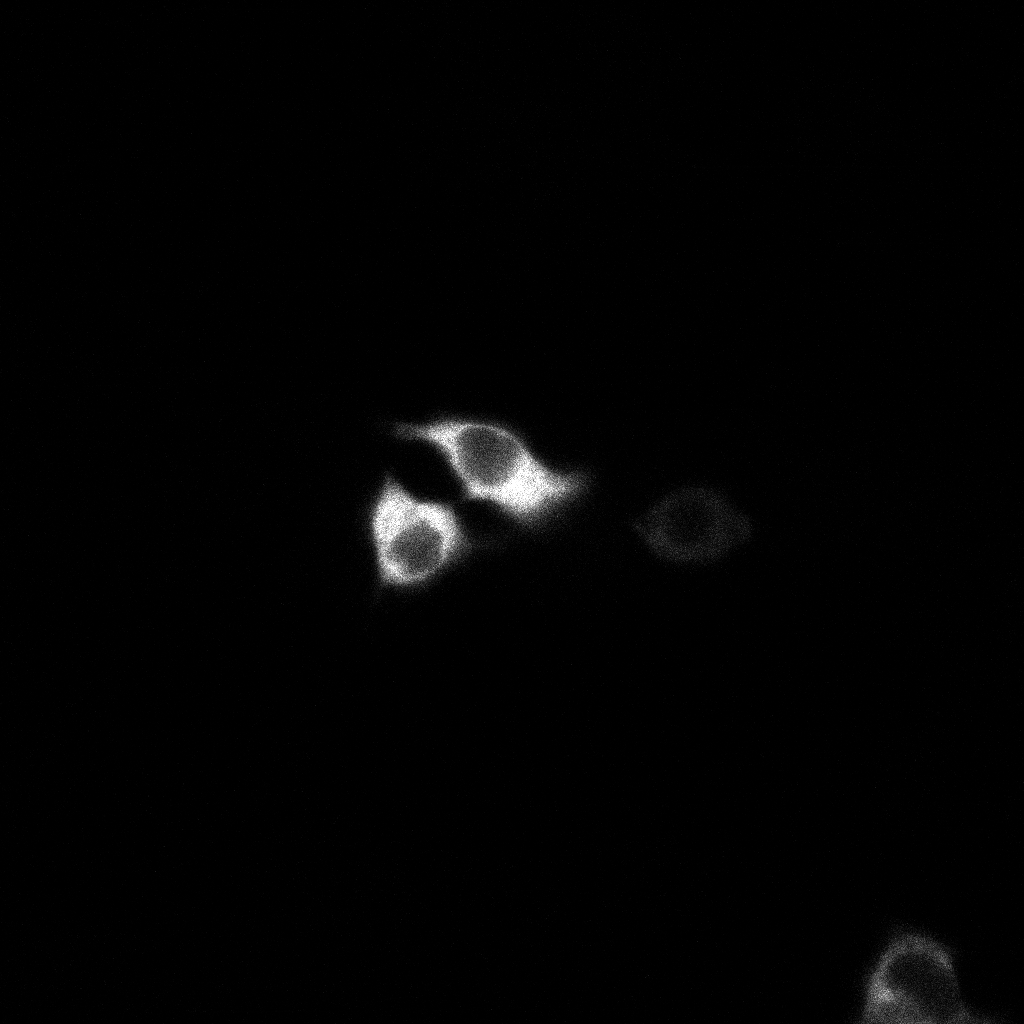

Supplement: Supplementary file 11 — Source Data [file 41467_2024_54263_MOESM11_ESM.zip › Source Data/Fig. 5/Fig. 5 B/Stable probe HEK + 300 probe + 100 staygoldm3p/lumi/Lumi180_2_X2.tif]

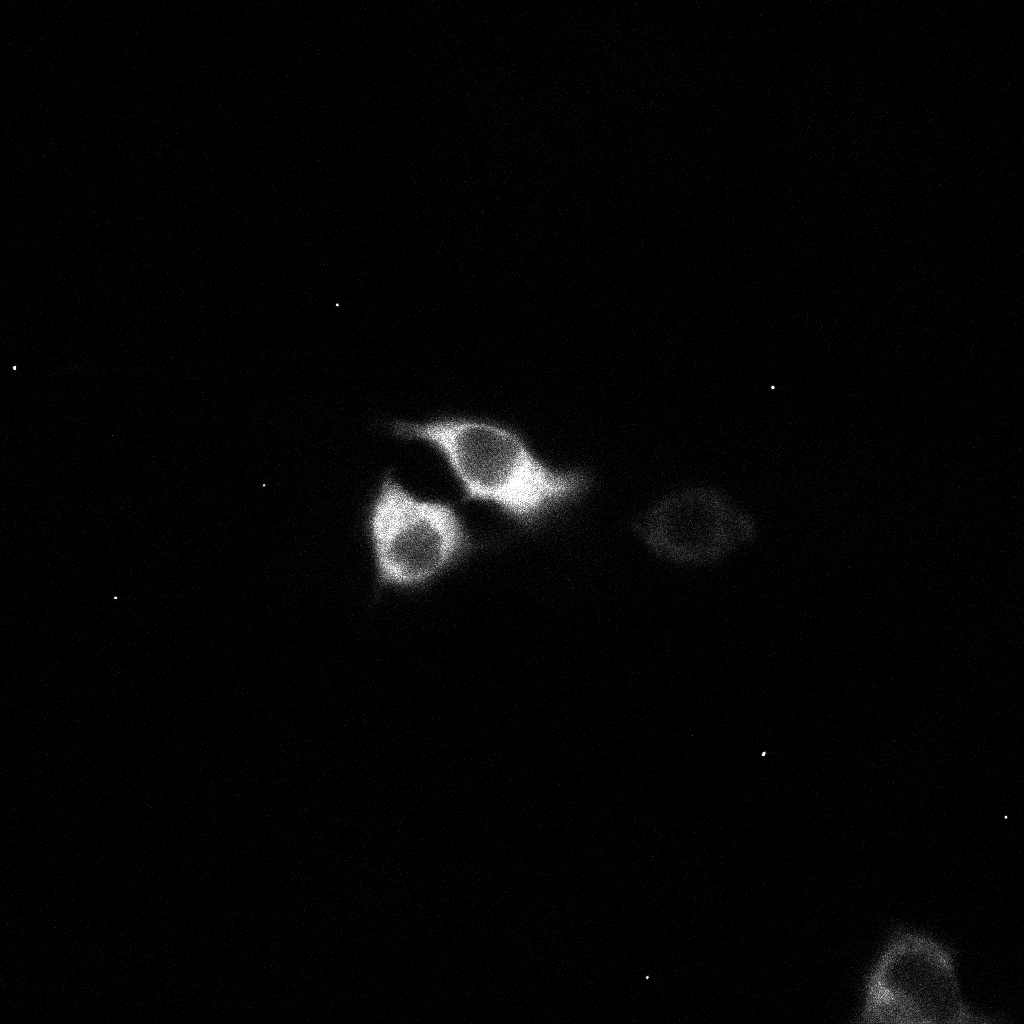

Supplement: Supplementary file 11 — Source Data [file 41467_2024_54263_MOESM11_ESM.zip › Source Data/Fig. 5/Fig. 5 B/Stable probe HEK + 300 probe + 100 staygoldm3p/lumi/Lumi180_2_X1.tif]

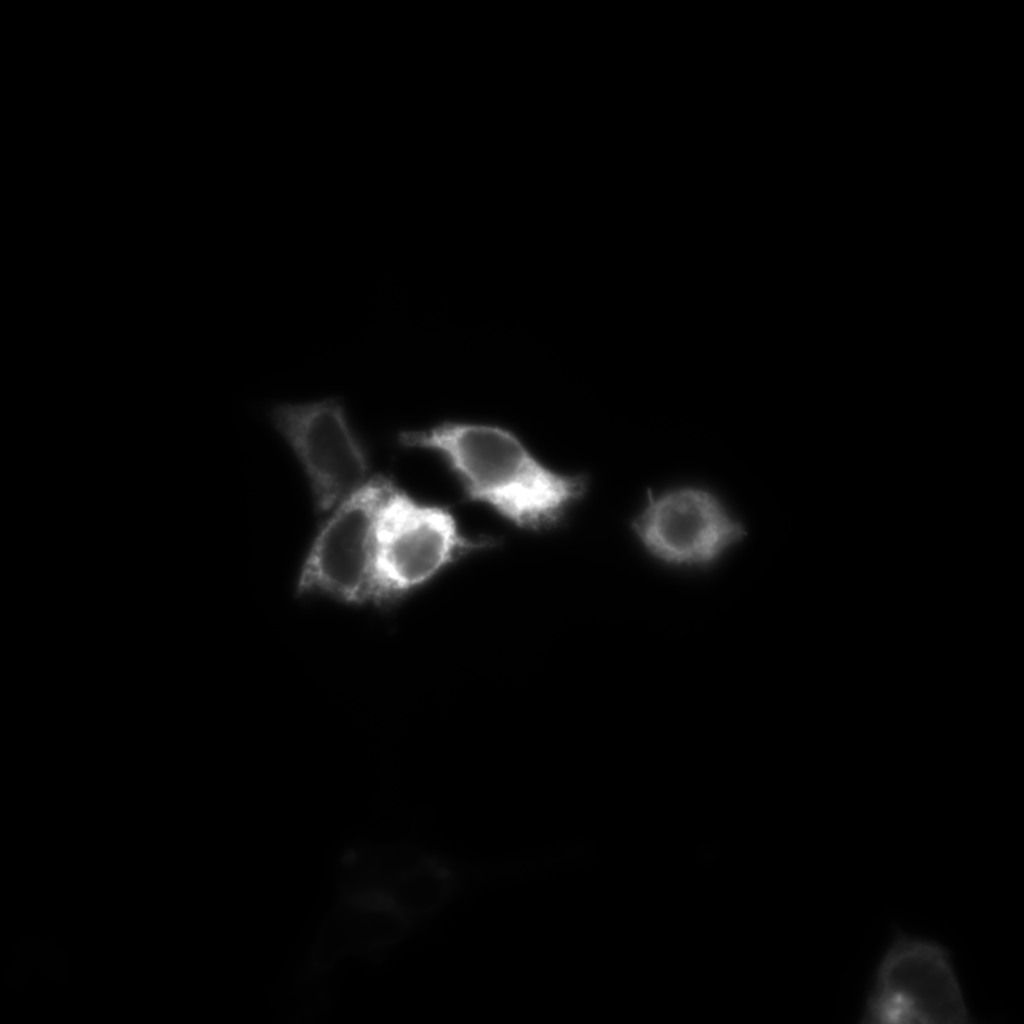

Supplement: Supplementary file 11 — Source Data [file 41467_2024_54263_MOESM11_ESM.zip › Source Data/Fig. 5/Fig. 5 B/Stable probe HEK + 300 probe + 100 staygoldm3p/edited/AVG_Stack-StayGold.tif]

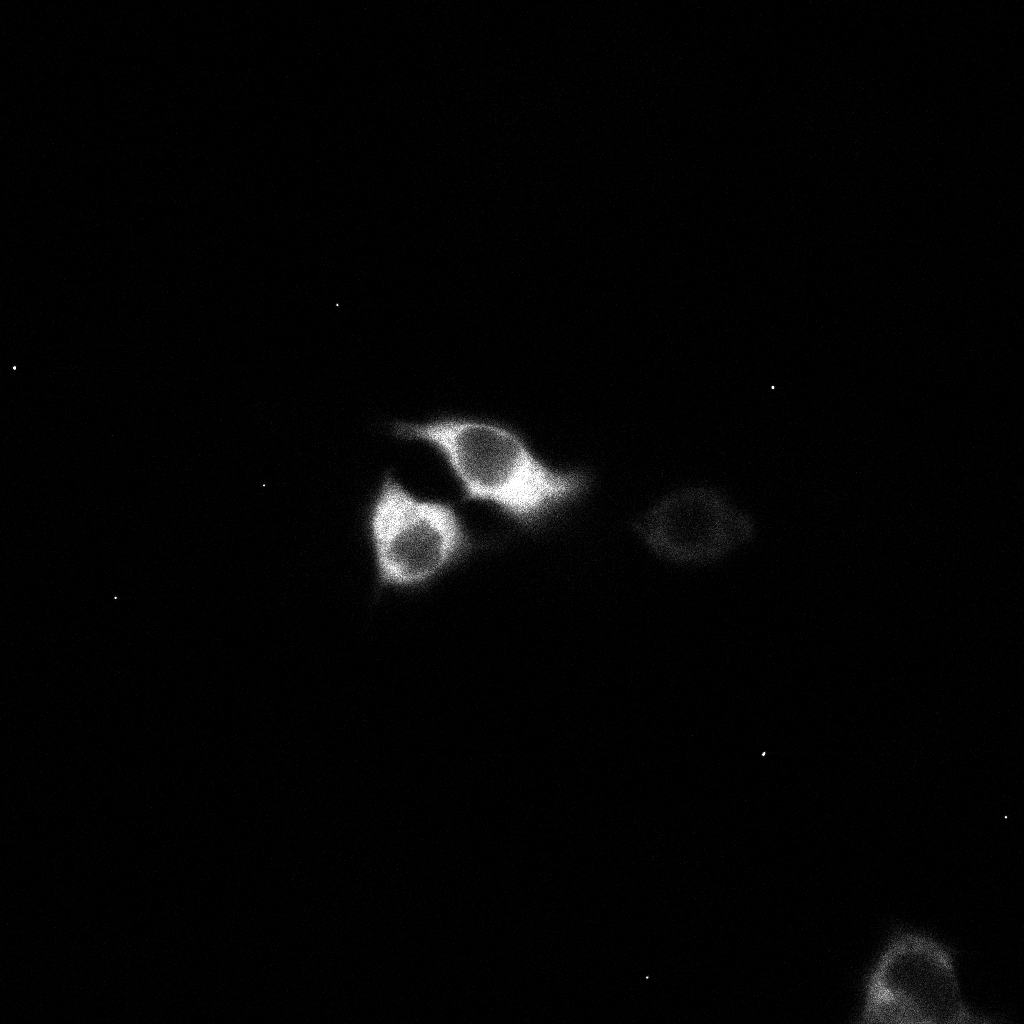

Supplement: Supplementary file 11 — Source Data [file 41467_2024_54263_MOESM11_ESM.zip › Source Data/Fig. 5/Fig. 5 B/Stable probe HEK + 300 probe + 100 staygoldm3p/edited/AVG_Stack-lumi.tif]

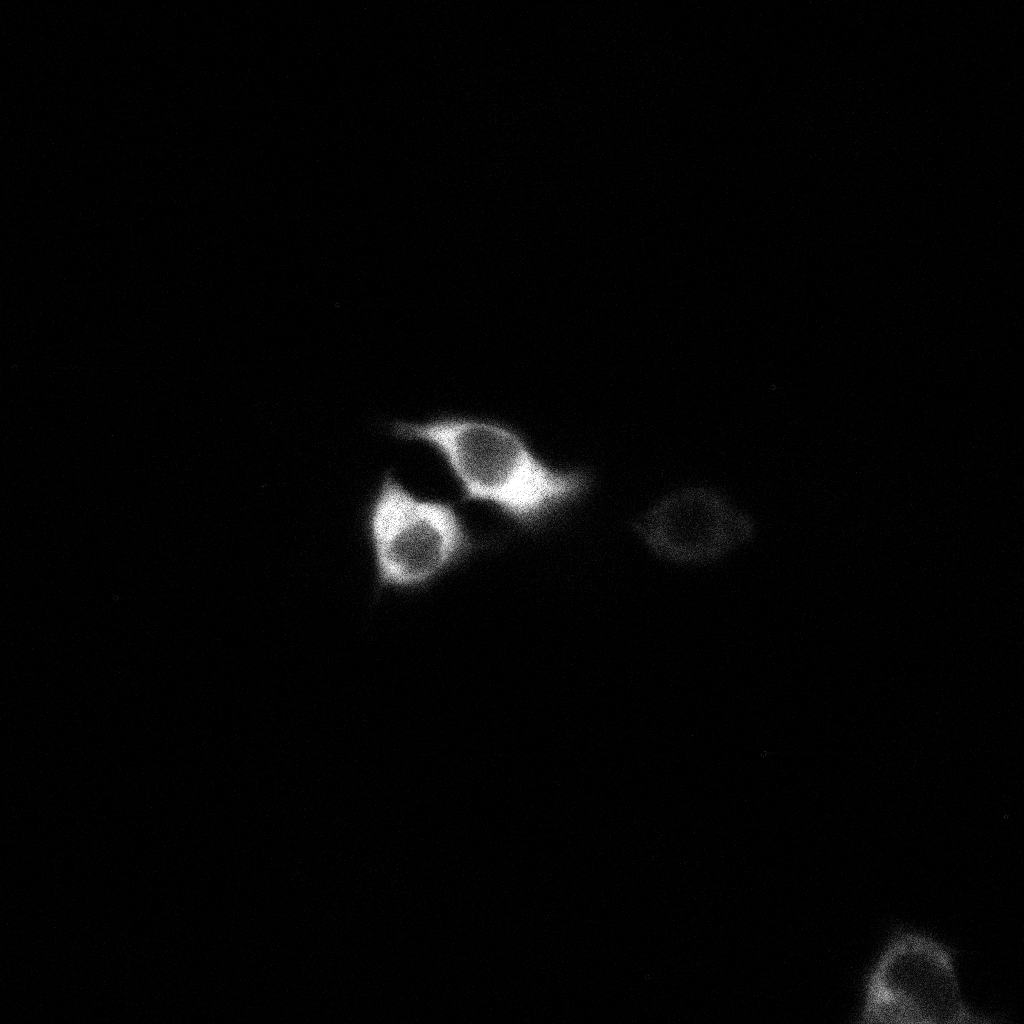

Supplement: Supplementary file 11 — Source Data [file 41467_2024_54263_MOESM11_ESM.zip › Source Data/Fig. 5/Fig. 5 B/Stable probe HEK + 300 probe + 100 staygoldm3p/edited/AVG_Stack-lumi-removeoutliers.tif]

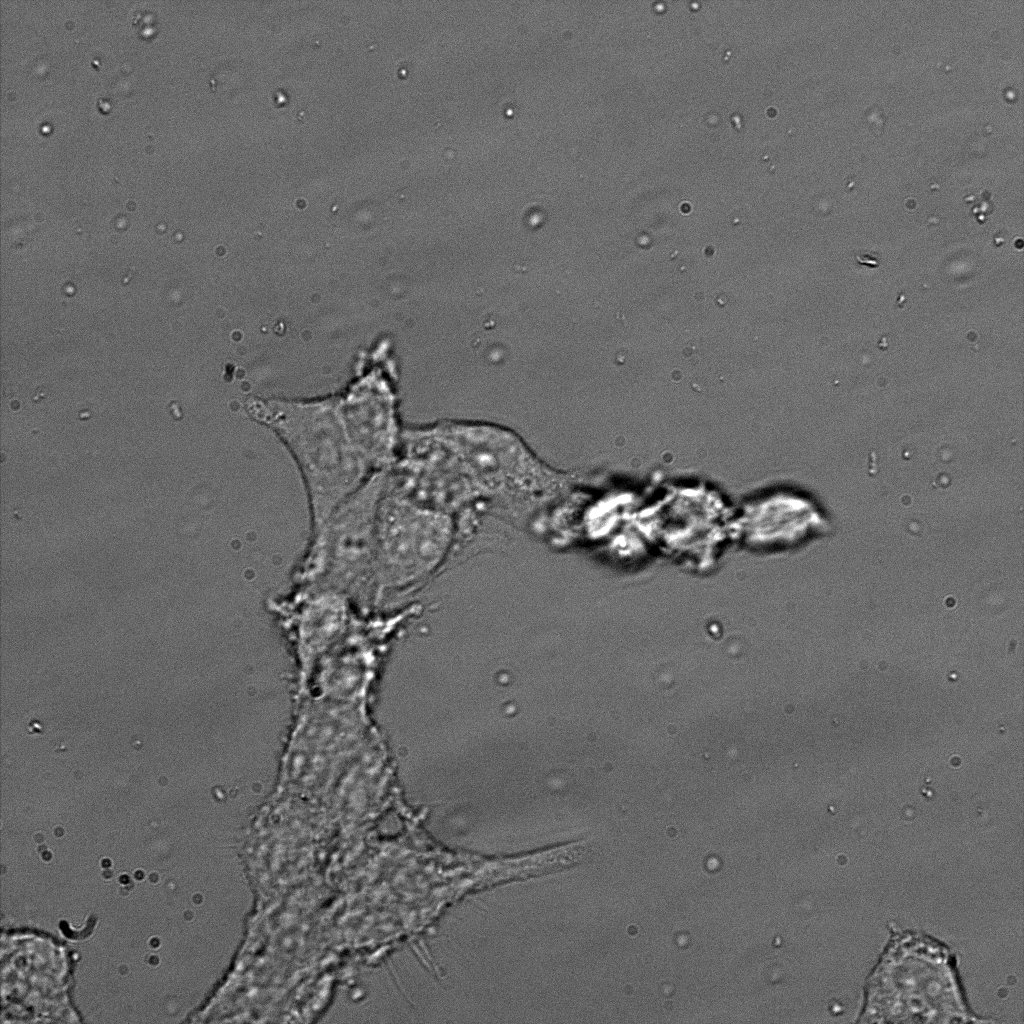

Supplement: Supplementary file 11 — Source Data [file 41467_2024_54263_MOESM11_ESM.zip › Source Data/Fig. 5/Fig. 5 B/Stable probe HEK + 300 probe + 100 staygoldm3p/BF/40X-brightfield_1_X1.tif]

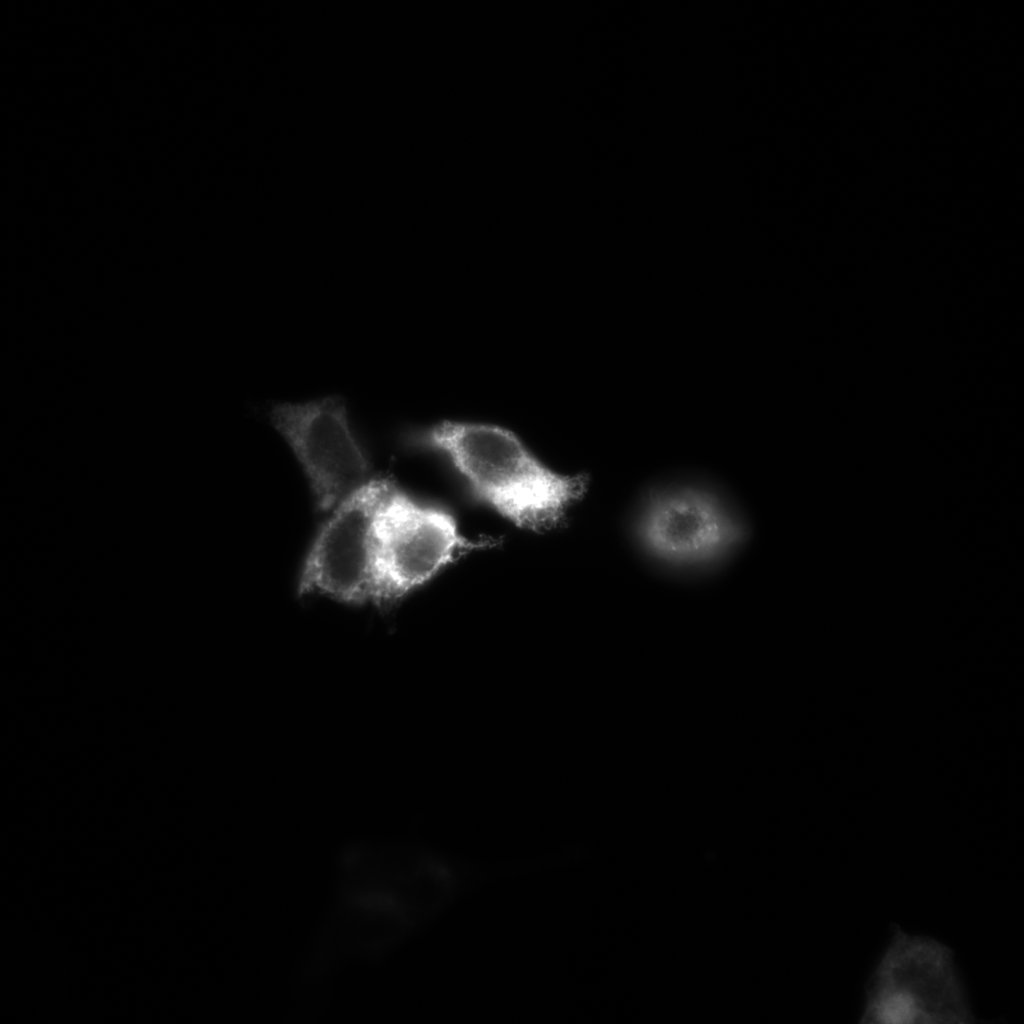

Supplement: Supplementary file 11 — Source Data [file 41467_2024_54263_MOESM11_ESM.zip › Source Data/Fig. 5/Fig. 5 B/Stable probe HEK + 300 probe + 100 staygoldm3p/fluorescence/StayGoldm3p100ng_7_X1.tif]

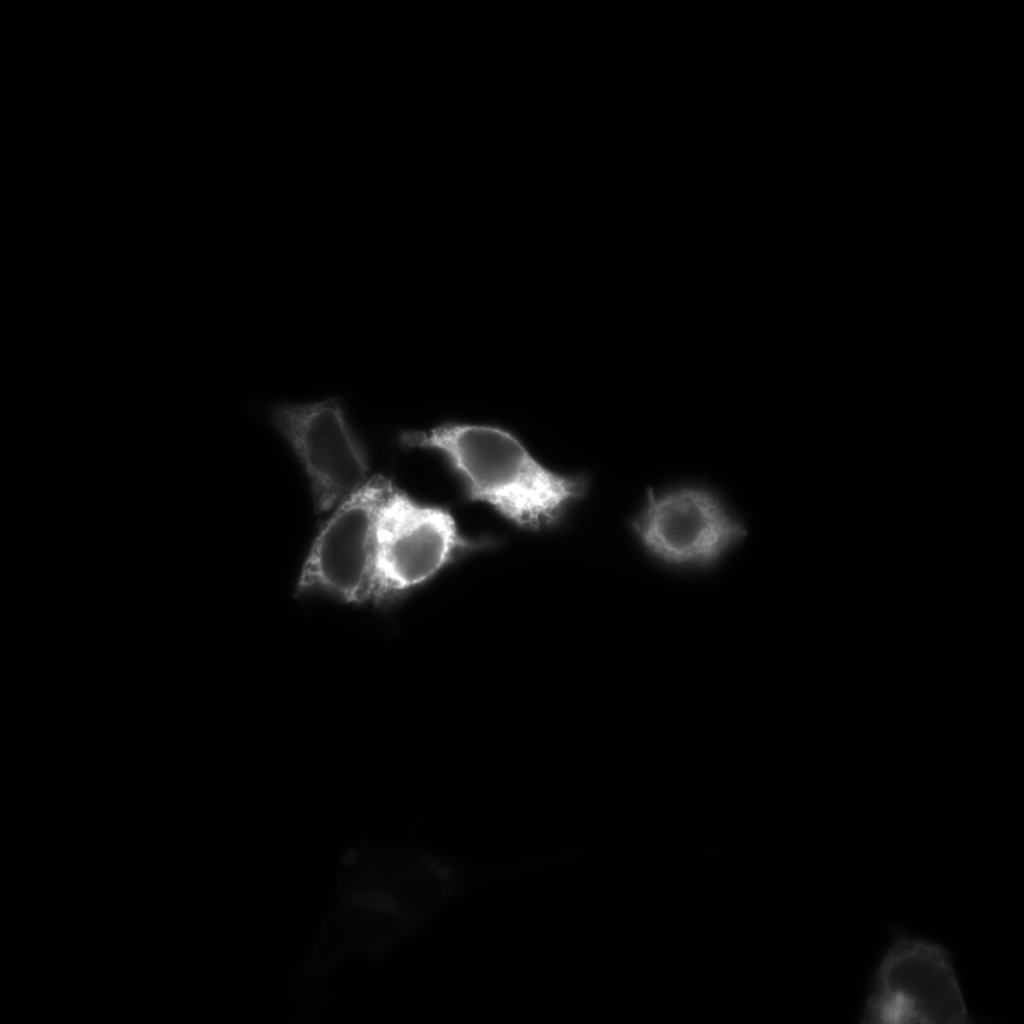

Supplement: Supplementary file 11 — Source Data [file 41467_2024_54263_MOESM11_ESM.zip › Source Data/Fig. 5/Fig. 5 B/Stable probe HEK + 300 probe + 100 staygoldm3p/fluorescence/StayGoldm3p100ng_5_X1.tif]

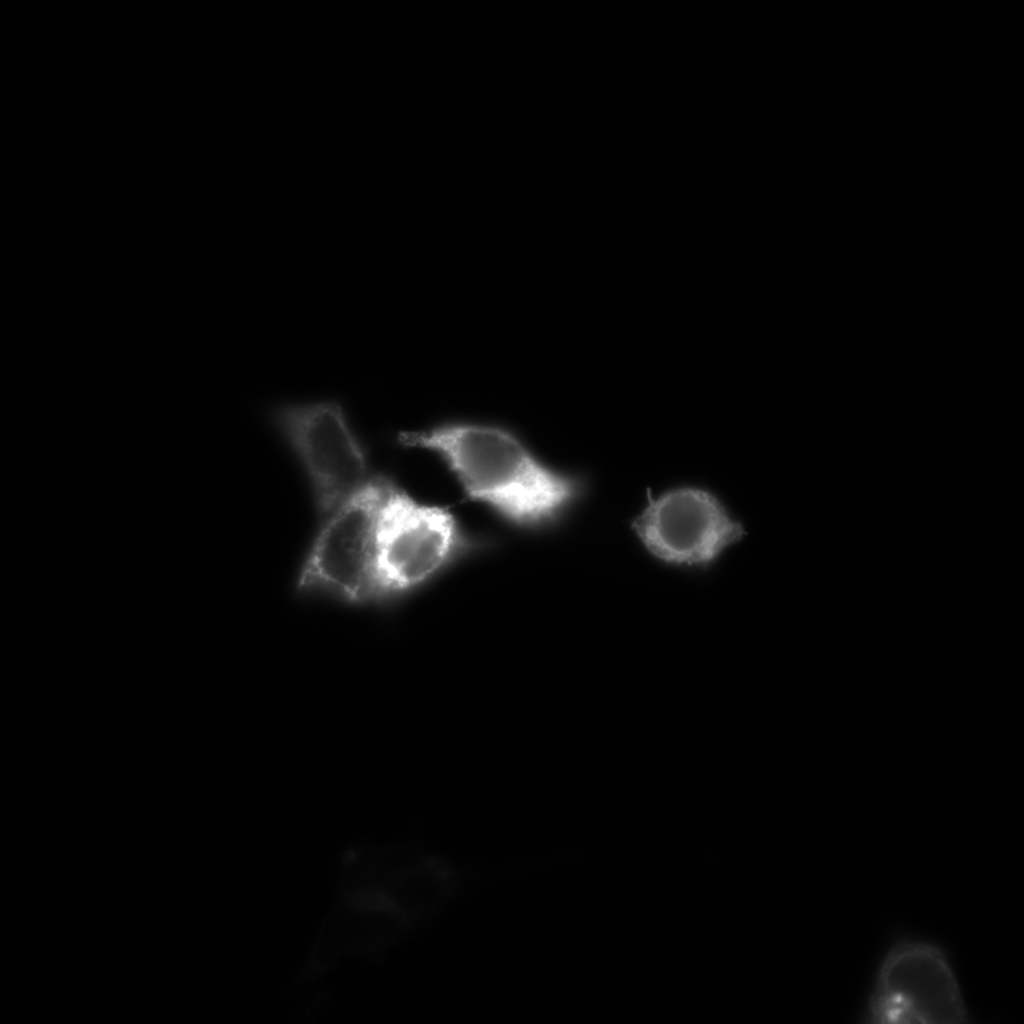

Supplement: Supplementary file 11 — Source Data [file 41467_2024_54263_MOESM11_ESM.zip › Source Data/Fig. 5/Fig. 5 B/Stable probe HEK + 300 probe + 100 staygoldm3p/fluorescence/StayGoldm3p100ng_1_X1.tif]

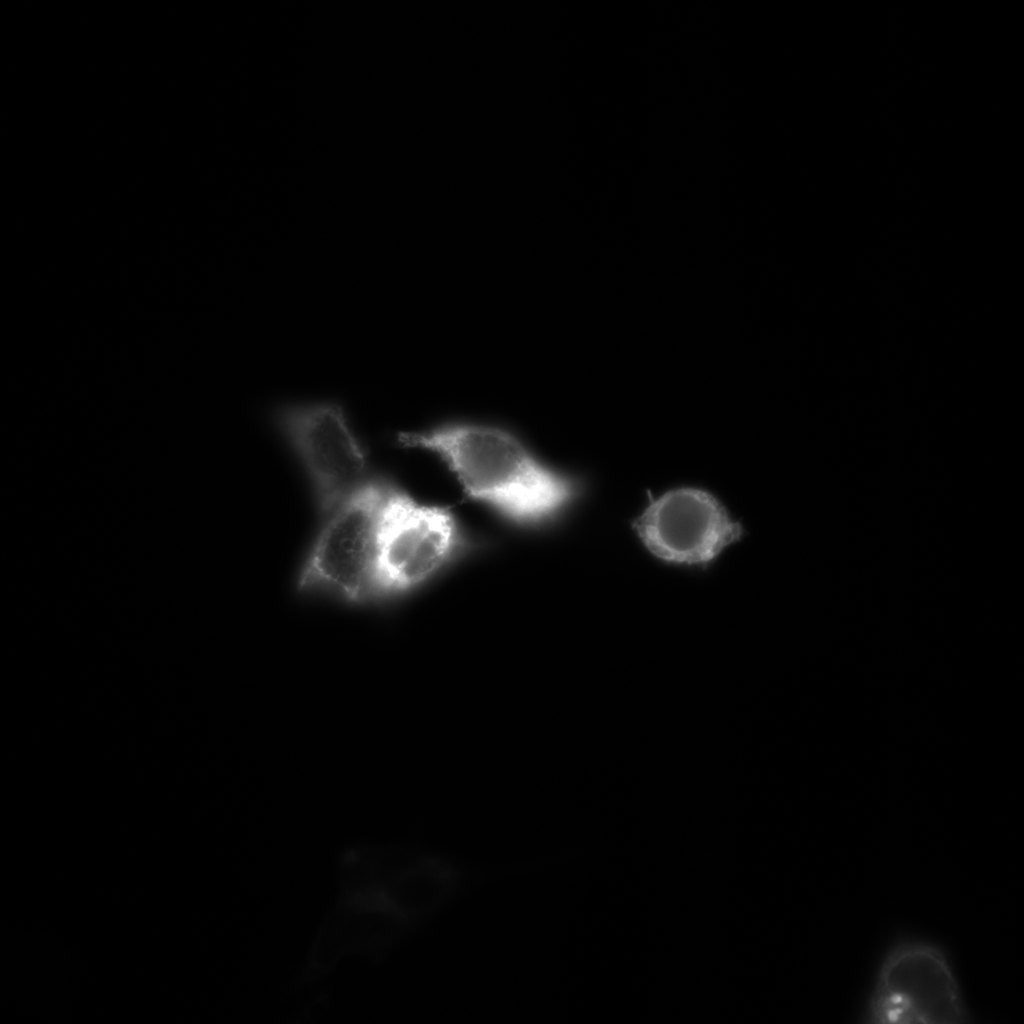

Supplement: Supplementary file 11 — Source Data [file 41467_2024_54263_MOESM11_ESM.zip › Source Data/Fig. 5/Fig. 5 B/Stable probe HEK + 300 probe + 100 staygoldm3p/fluorescence/StayGoldm3p100ng_3_X1.tif]

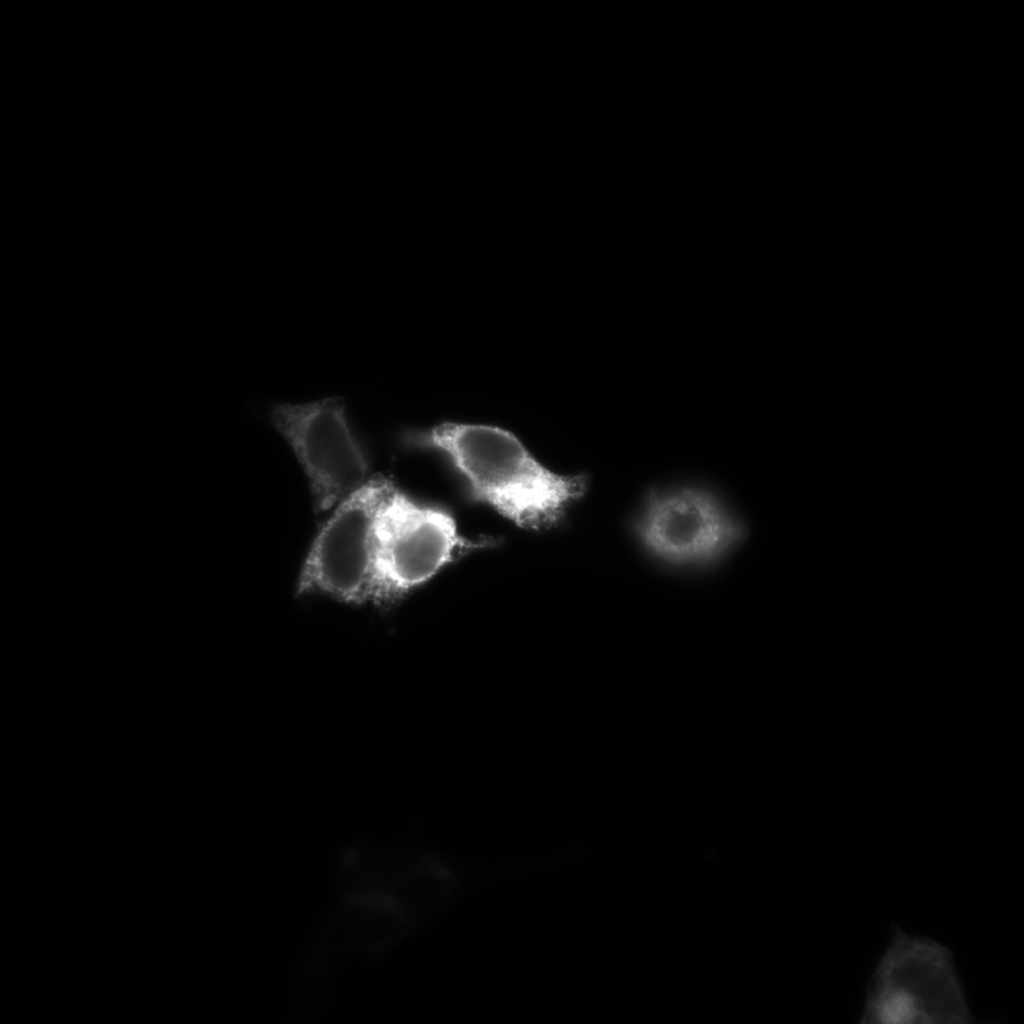

Supplement: Supplementary file 11 — Source Data [file 41467_2024_54263_MOESM11_ESM.zip › Source Data/Fig. 5/Fig. 5 B/Stable probe HEK + 300 probe + 100 staygoldm3p/fluorescence/StayGoldm3p100ng_6_X1.tif]

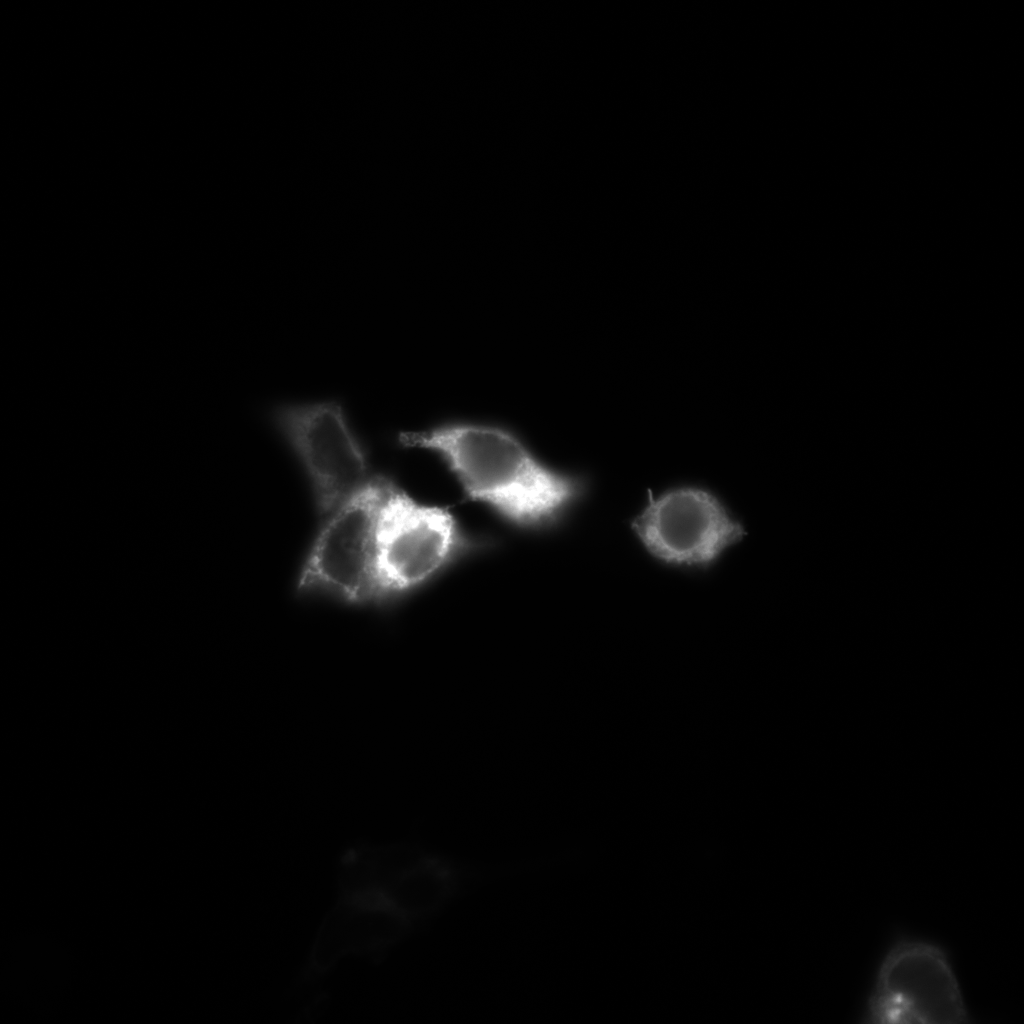

Supplement: Supplementary file 11 — Source Data [file 41467_2024_54263_MOESM11_ESM.zip › Source Data/Fig. 5/Fig. 5 B/Stable probe HEK + 300 probe + 100 staygoldm3p/fluorescence/StayGoldm3p100ng_4_X1.tif]

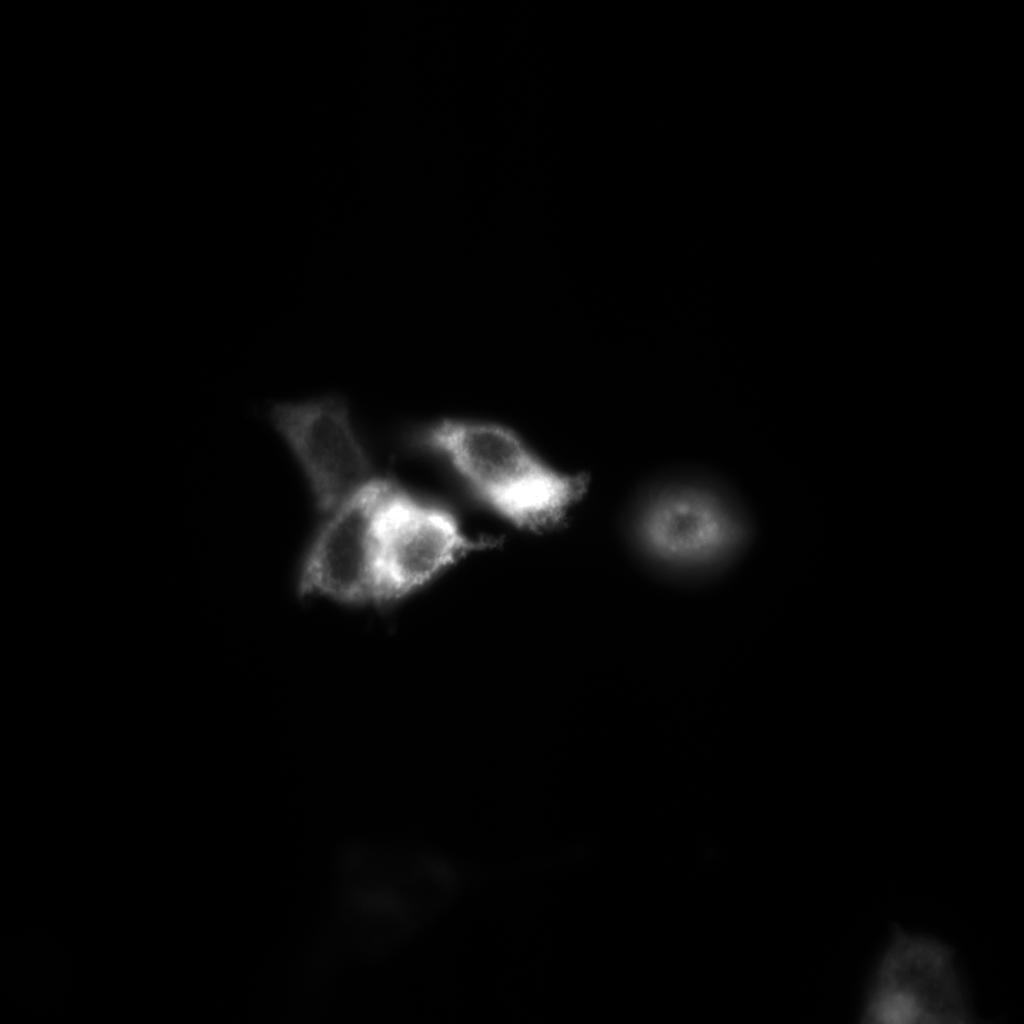

Supplement: Supplementary file 11 — Source Data [file 41467_2024_54263_MOESM11_ESM.zip › Source Data/Fig. 5/Fig. 5 B/Stable probe HEK + 300 probe + 100 staygoldm3p/fluorescence/StayGoldm3p100ng_8_X1.tif]

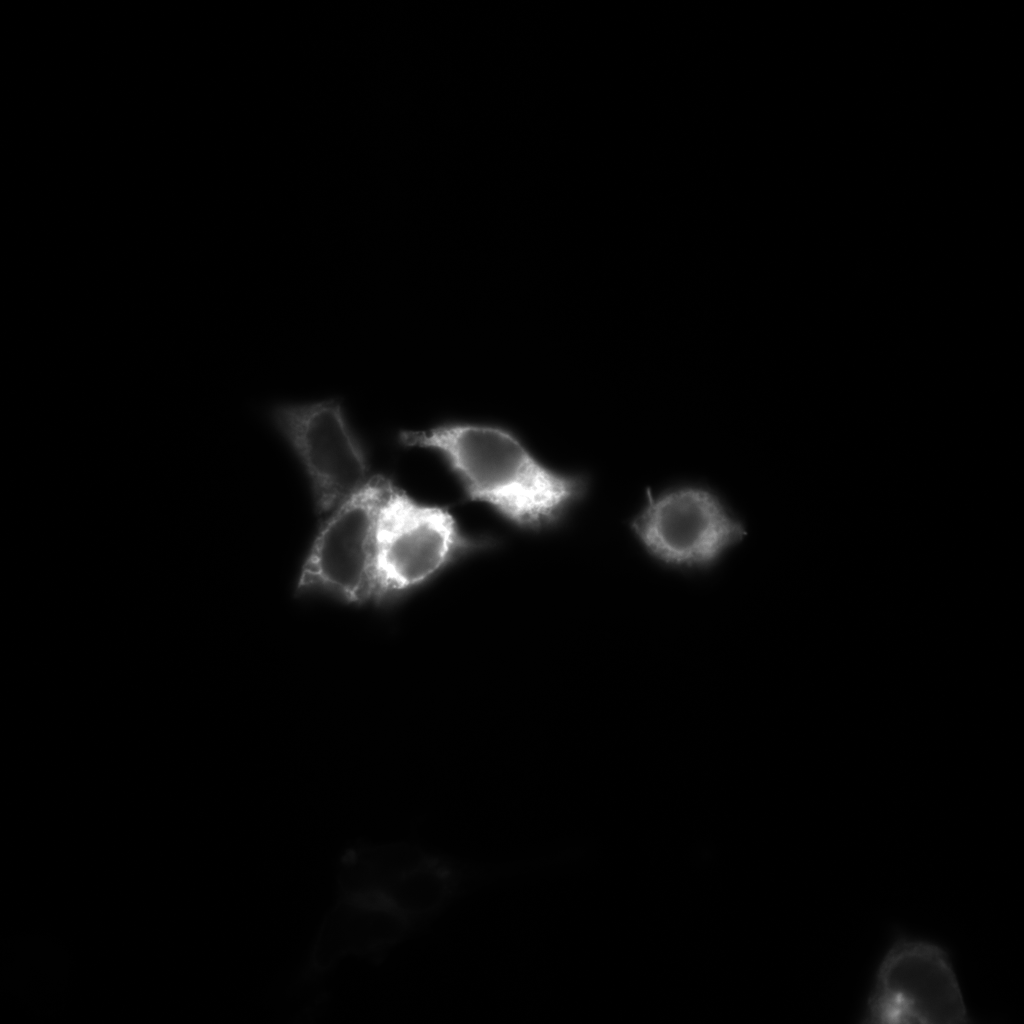

Supplement: Supplementary file 11 — Source Data [file 41467_2024_54263_MOESM11_ESM.zip › Source Data/Fig. 5/Fig. 5 B/Stable probe HEK + 300 probe + 100 staygoldm3p/fluorescence/StayGoldm3p100ng_0_X1.tif]

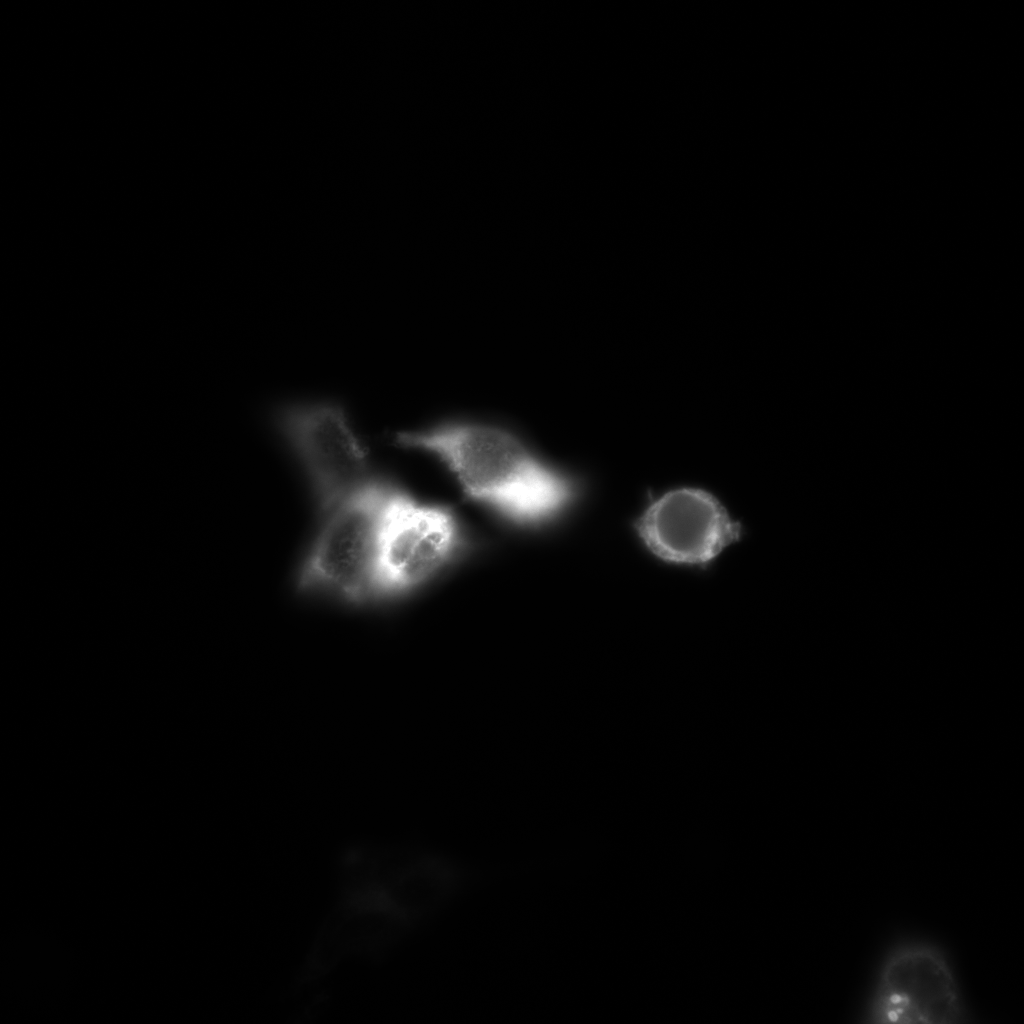

Supplement: Supplementary file 11 — Source Data [file 41467_2024_54263_MOESM11_ESM.zip › Source Data/Fig. 5/Fig. 5 B/Stable probe HEK + 300 probe + 100 staygoldm3p/fluorescence/StayGoldm3p100ng_2_X1.tif]

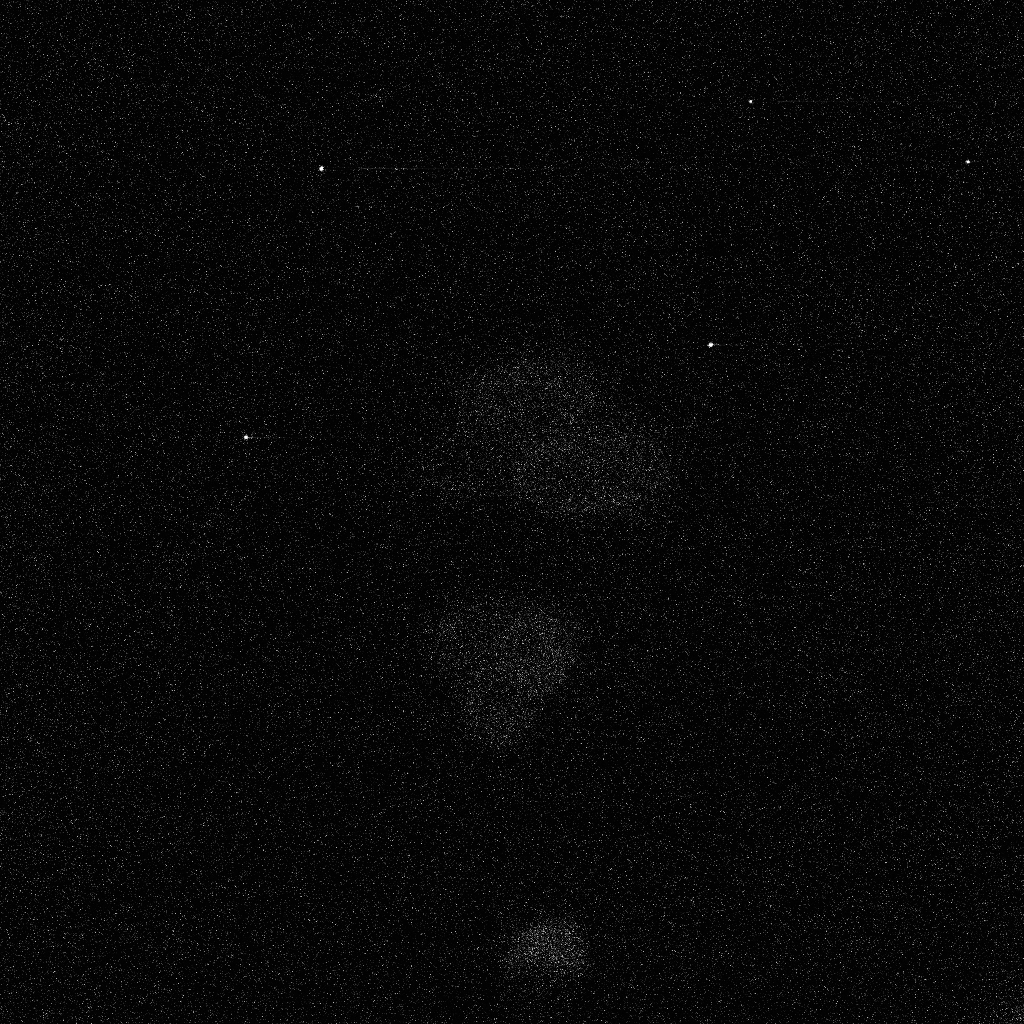

Supplement: Supplementary file 11 — Source Data [file 41467_2024_54263_MOESM11_ESM.zip › Source Data/Fig. 5/Fig. 5 B/Stable probe HEK + 300 probe + 100staygoldcontrol/lumi/Lumi180_1_X1.tif]

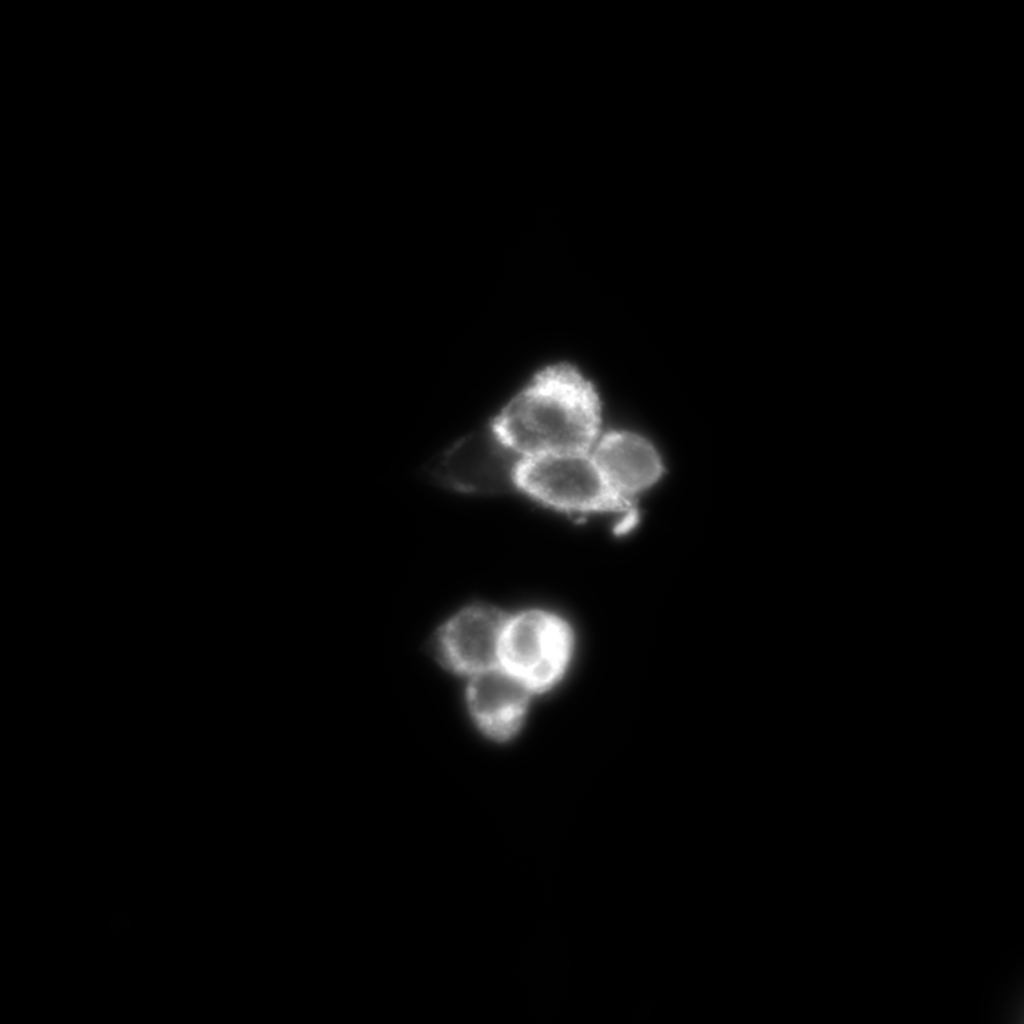

Supplement: Supplementary file 11 — Source Data [file 41467_2024_54263_MOESM11_ESM.zip › Source Data/Fig. 5/Fig. 5 B/Stable probe HEK + 300 probe + 100staygoldcontrol/edited/Composite.tif]

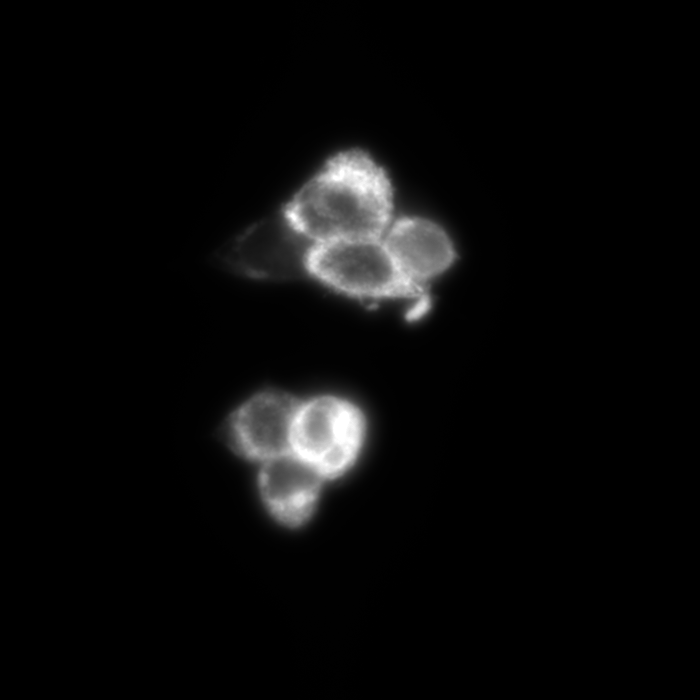

Supplement: Supplementary file 11 — Source Data [file 41467_2024_54263_MOESM11_ESM.zip › Source Data/Fig. 5/Fig. 5 B/Stable probe HEK + 300 probe + 100staygoldcontrol/edited/Composite-ROI-700.tif]

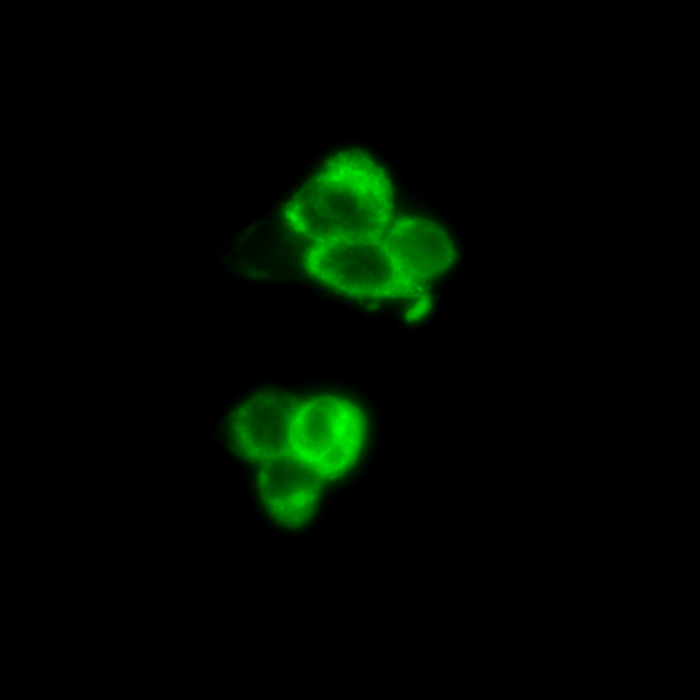

Supplement: Supplementary file 11 — Source Data [file 41467_2024_54263_MOESM11_ESM.zip › Source Data/Fig. 5/Fig. 5 B/Stable probe HEK + 300 probe + 100staygoldcontrol/edited/Composite-ROI-700-StayGold.tif (RGB).tif]

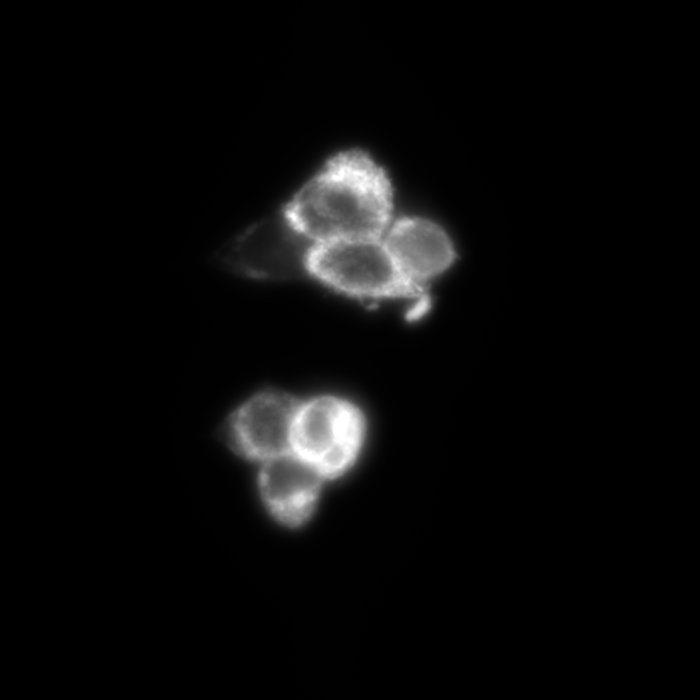

Supplement: Supplementary file 11 — Source Data [file 41467_2024_54263_MOESM11_ESM.zip › Source Data/Fig. 5/Fig. 5 B/Stable probe HEK + 300 probe + 100staygoldcontrol/edited/Composite-ROI-700-20umscalebar.tif]

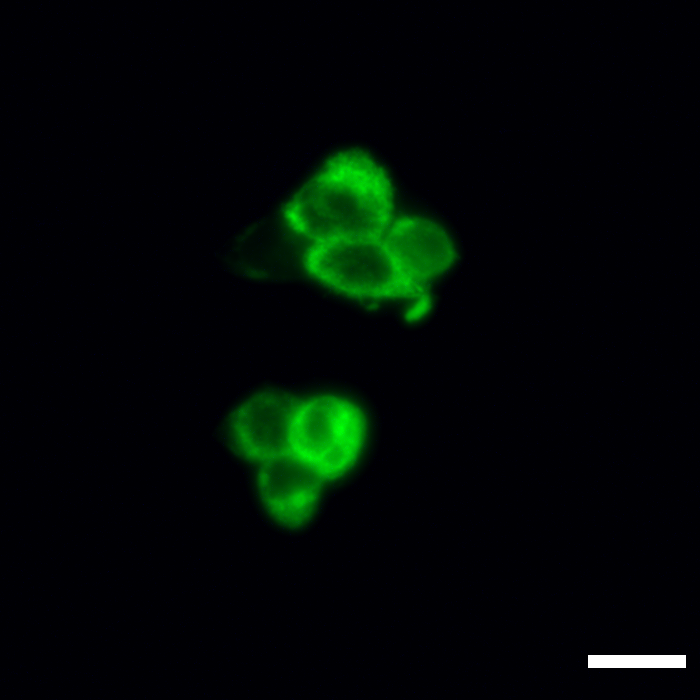

Supplement: Supplementary file 11 — Source Data [file 41467_2024_54263_MOESM11_ESM.zip › Source Data/Fig. 5/Fig. 5 B/Stable probe HEK + 300 probe + 100staygoldcontrol/edited/Composite-ROI-700-20umscalebar.tif (RGB).tif]

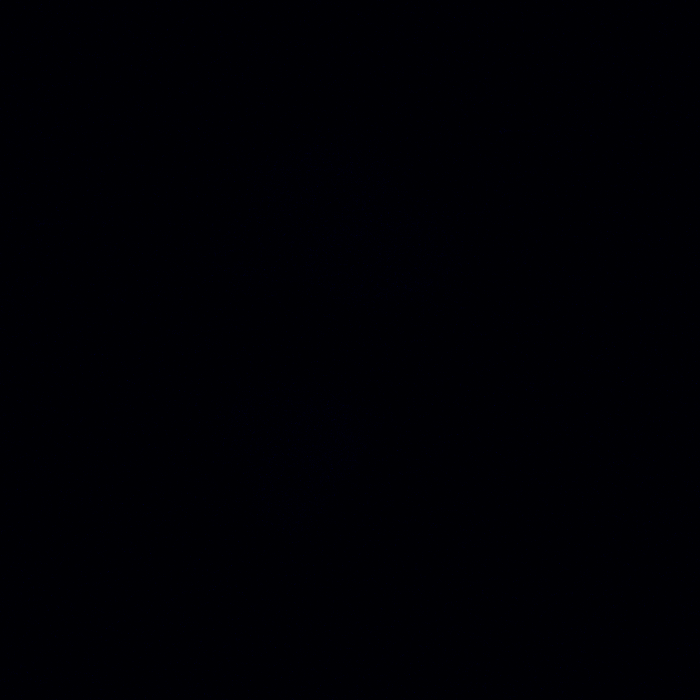

Supplement: Supplementary file 11 — Source Data [file 41467_2024_54263_MOESM11_ESM.zip › Source Data/Fig. 5/Fig. 5 B/Stable probe HEK + 300 probe + 100staygoldcontrol/edited/Composite-ROI-700-Lumi.tif (RGB).tif]

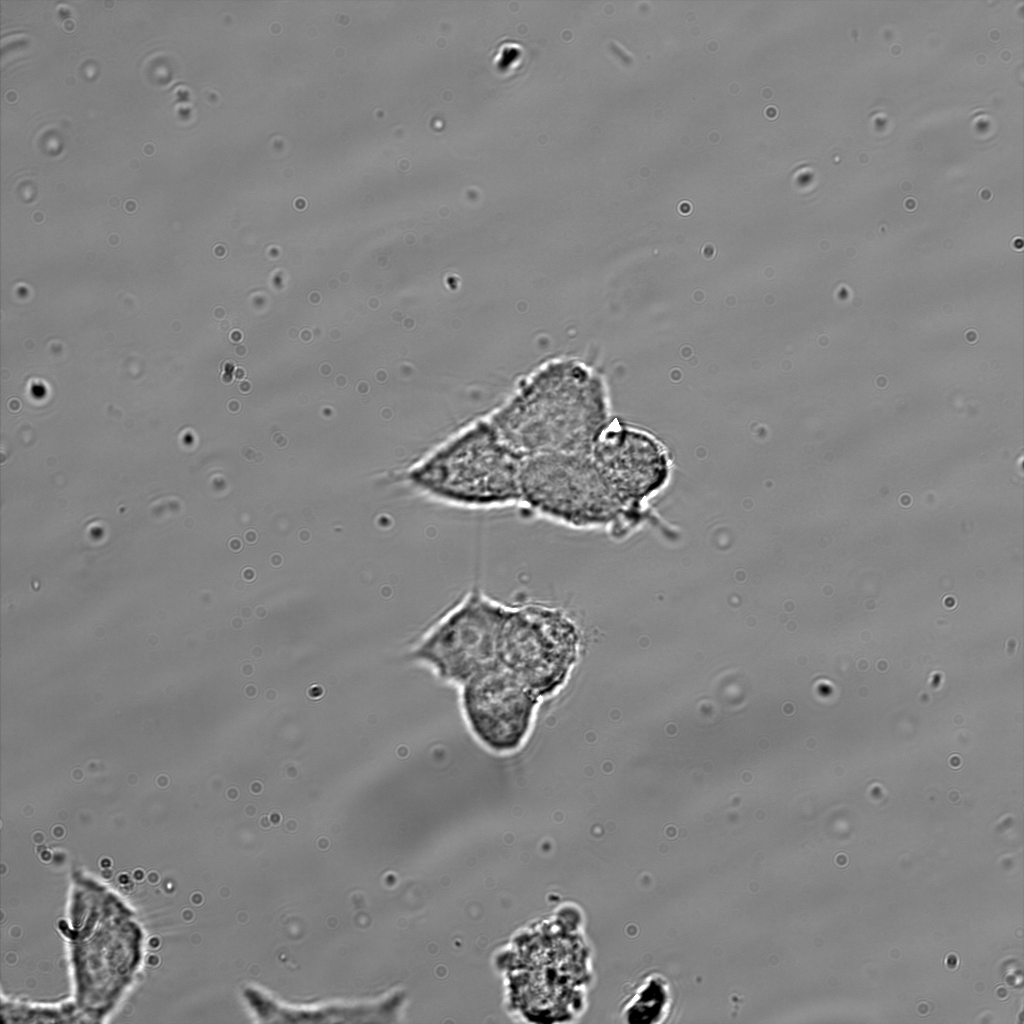

Supplement: Supplementary file 11 — Source Data [file 41467_2024_54263_MOESM11_ESM.zip › Source Data/Fig. 5/Fig. 5 B/Stable probe HEK + 300 probe + 100staygoldcontrol/BF/40X-brightfield_2_X1.tif]

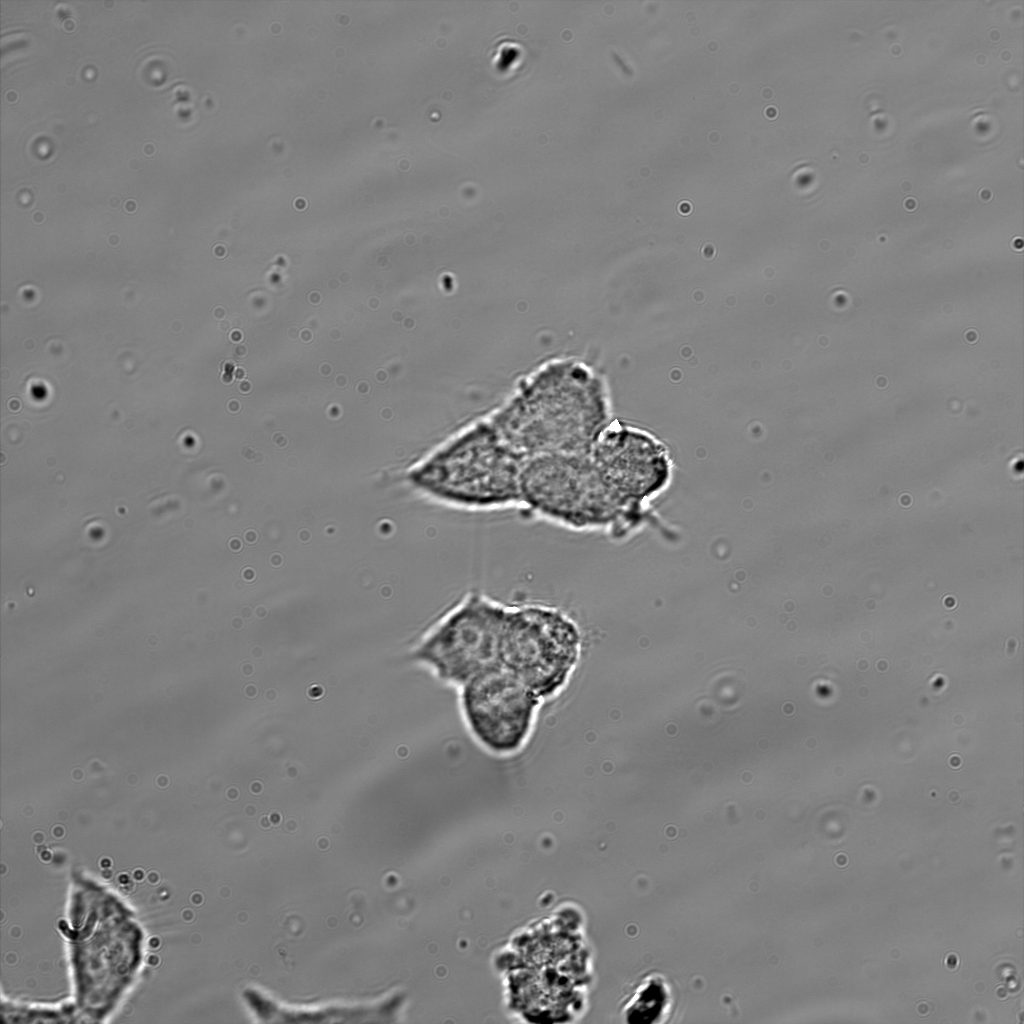

Supplement: Supplementary file 11 — Source Data [file 41467_2024_54263_MOESM11_ESM.zip › Source Data/Fig. 5/Fig. 5 B/Stable probe HEK + 300 probe + 100staygoldcontrol/BF/40X-brightfield_3_X1.tif]

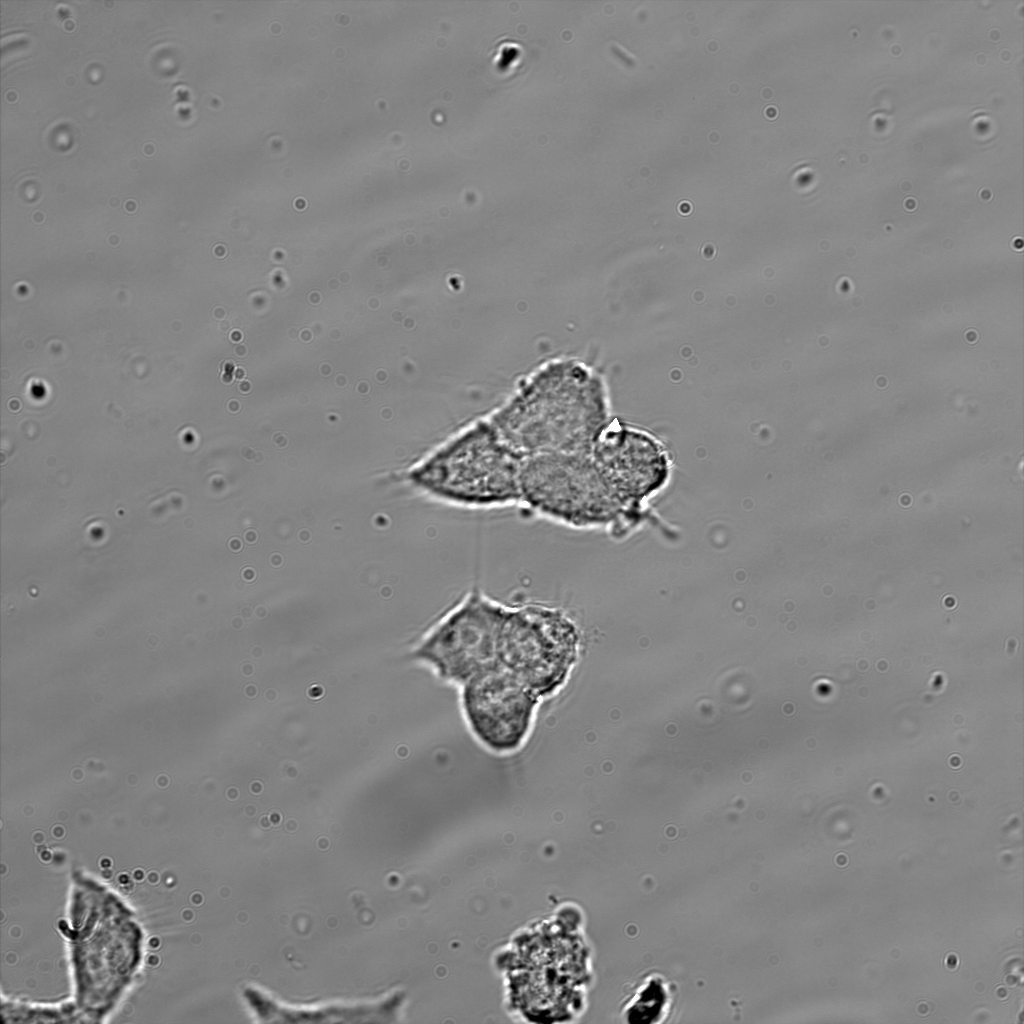

Supplement: Supplementary file 11 — Source Data [file 41467_2024_54263_MOESM11_ESM.zip › Source Data/Fig. 5/Fig. 5 B/Stable probe HEK + 300 probe + 100staygoldcontrol/BF/40X-brightfield_1_X1.tif]
